# Heterogeneous contributions of change in population distribution of body mass index to change in obesity and underweight

**DOI:** 10.7554/eLife.60060

**Published:** 2021-03-09

**Authors:** NCD Risk Factor Collaboration (NCD-RisC), Maria LC Iurilli, Bin Zhou, James E Bennett, Rodrigo M Carrillo-Larco, Marisa K Sophiea, Andrea Rodriguez-Martinez, Honor Bixby, Bethlehem D Solomon, Cristina Taddei, Goodarz Danaei, Mariachiara Di Cesare, Gretchen A Stevens, Leanne M Riley, Stefan Savin, Melanie J Cowan, Pascal Bovet, Albertino Damasceno, Adela Chirita-Emandi, Alison J Hayes, Nayu Ikeda, Rod T Jackson, Young-Ho Khang, Avula Laxmaiah, Jing Liu, J Jaime Miranda, Olfa Saidi, Sylvain Sebert, Maroje Sorić, Gregor Starc, Edward W Gregg, Leandra Abarca-Gómez, Ziad A Abdeen, Shynar Abdrakhmanova, Suhaila Abdul Ghaffar, Hanan F Abdul Rahim, Niveen M Abu-Rmeileh, Jamila Abubakar Garba, Benjamin Acosta-Cazares, Robert J Adams, Wichai Aekplakorn, Kaosar Afsana, Shoaib Afzal, Imelda A Agdeppa, Javad Aghazadeh-Attari, Carlos A Aguilar-Salinas, Charles Agyemang, Mohamad Hasnan Ahmad, Noor Ani Ahmad, Ali Ahmadi, Naser Ahmadi, Soheir H Ahmed, Wolfgang Ahrens, Gulmira Aitmurzaeva, Kamel Ajlouni, Hazzaa M Al-Hazzaa, Badreya Al-Lahou, Rajaa Al-Raddadi, Monira Alarouj, Fadia AlBuhairan, Shahla AlDhukair, Mohamed M Ali, Abdullah Alkandari, Ala'a Alkerwi, Kristine Allin, Mar Alvarez-Pedrerol, Eman Aly, Deepak N Amarapurkar, Parisa Amiri, Norbert Amougou, Philippe Amouyel, Lars Bo Andersen, Sigmund A Anderssen, Lars Ängquist, Ranjit Mohan Anjana, Alireza Ansari-Moghaddam, Hajer Aounallah-Skhiri, Joana Araújo, Inger Ariansen, Tahir Aris, Raphael E Arku, Nimmathota Arlappa, Krishna K Aryal, Thor Aspelund, Felix K Assah, Maria Cecília F Assunção, May Soe Aung, Juha Auvinen, Mária Avdicová, Shina Avi, Ana Azevedo, Mohsen Azimi-Nezhad, Fereidoun Azizi, Mehrdad Azmin, Bontha V Babu, Maja Bæksgaard Jørgensen, Azli Baharudin, Suhad Bahijri, Jennifer L Baker, Nagalla Balakrishna, Mohamed Bamoshmoosh, Maciej Banach, Piotr Bandosz, José R Banegas, Joanna Baran, Carlo M Barbagallo, Alberto Barceló, Amina Barkat, Aluisio JD Barros, Mauro Virgílio Gomes Barros, Abdul Basit, Joao Luiz D Bastos, Iqbal Bata, Anwar M Batieha, Rosangela L Batista, Zhamilya Battakova, Assembekov Batyrbek, Louise A Baur, Robert Beaglehole, Silvia Bel-Serrat, Antonisamy Belavendra, Habiba Ben Romdhane, Judith Benedics, Mikhail Benet, Ingunn Holden Bergh, Salim Berkinbayev, Antonio Bernabe-Ortiz, Gailute Bernotiene, Heloísa Bettiol, Jorge Bezerra, Aroor Bhagyalaxmi, Sumit Bharadwaj, Santosh K Bhargava, Zulfiqar A Bhutta, Hongsheng Bi, Yufang Bi, Daniel Bia, Elysée Claude Bika Lele, Mukharram M Bikbov, Bihungum Bista, Dusko J Bjelica, Peter Bjerregaard, Espen Bjertness, Marius B Bjertness, Cecilia Björkelund, Katia V Bloch, Anneke Blokstra, Simona Bo, Martin Bobak, Lynne M Boddy, Bernhard O Boehm, Heiner Boeing, Jose G Boggia, Elena Bogova, Carlos P Boissonnet, Stig E Bojesen, Marialaura Bonaccio, Vanina Bongard, Alice Bonilla-Vargas, Matthias Bopp, Herman Borghs, Lien Braeckevelt, Lutgart Braeckman, Marjolijn CE Bragt, Imperia Brajkovich, Francesco Branca, Juergen Breckenkamp, João Breda, Hermann Brenner, Lizzy M Brewster, Garry R Brian, Lacramioara Brinduse, Sinead Brophy, Graziella Bruno, H Bas Bueno-de-Mesquita, Anna Bugge, Marta Buoncristiano, Genc Burazeri, Con Burns, Antonio Cabrera de León, Joseph Cacciottolo, Hui Cai, Tilema Cama, Christine Cameron, José Camolas, Günay Can, Ana Paula C Cândido, Felicia Cañete, Mario V Capanzana, Nadežda Capková, Eduardo Capuano, Vincenzo Capuano, Marloes Cardol, Viviane C Cardoso, Axel C Carlsson, Esteban Carmuega, Joana Carvalho, José A Casajús, Felipe F Casanueva, Ertugrul Celikcan, Laura Censi, Marvin Cervantes-Loaiza, Juraci A Cesar, Snehalatha Chamukuttan, Angelique W Chan, Queenie Chan, Himanshu K Chaturvedi, Nish Chaturvedi, Norsyamlina Che Abdul Rahim, Miao Li Chee, Chien-Jen Chen, Fangfang Chen, Huashuai Chen, Shuohua Chen, Zhengming Chen, Ching-Yu Cheng, Bahman Cheraghian, Angela Chetrit, Ekaterina Chikova-Iscener, Arnaud Chiolero, Shu-Ti Chiou, María-Dolores Chirlaque, Belong Cho, Kaare Christensen, Diego G Christofaro, Jerzy Chudek, Renata Cifkova, Michelle Cilia, Eliza Cinteza, Frank Claessens, Janine Clarke, Els Clays, Emmanuel Cohen, Hans Concin, Susana C Confortin, Cyrus Cooper, Tara C Coppinger, Eva Corpeleijn, Simona Costanzo, Dominique Cottel, Chris Cowell, Cora L Craig, Amelia C Crampin, Ana B Crujeiras, Semánová Csilla, Alexandra M Cucu, Liufu Cui, Felipe V Cureau, Ewelina Czenczek-Lewandowska, Graziella D'Arrigo, Eleonora d'Orsi, Liliana Dacica, María Ángeles Dal Re Saavedra, Jean Dallongeville, Camilla T Damsgaard, Rachel Dankner, Thomas M Dantoft, Parasmani Dasgupta, Saeed Dastgiri, Luc Dauchet, Kairat Davletov, Guy De Backer, Dirk De Bacquer, Giovanni de Gaetano, Stefaan De Henauw, Paula Duarte de Oliveira, David De Ridder, Karin De Ridder, Susanne R de Rooij, Delphine De Smedt, Mohan Deepa, Alexander D Deev, Vincent Jr DeGennaro, Abbas Dehghan, Hélène Delisle, Francis Delpeuch, Stefaan Demarest, Elaine Dennison, Katarzyna Dereń, Valérie Deschamps, Meghnath Dhimal, Augusto F Di Castelnuovo, Juvenal Soares Dias-da-Costa, María Elena Díaz-Sánchez, Alejandro Diaz, Zivka Dika, Shirin Djalalinia, Visnja Djordjic, Ha TP Do, Annette J Dobson, Maria Benedetta Donati, Chiara Donfrancesco, Silvana P Donoso, Angela Döring, Maria Dorobantu, Ahmad Reza Dorosty, Kouamelan Doua, Nico Dragano, Wojciech Drygas, Jia Li Duan, Charmaine A Duante, Priscilla Duboz, Rosemary B Duda, Vesselka Duleva, Virginija Dulskiene, Samuel C Dumith, Anar Dushpanova, Vilnis Dzerve, Elzbieta Dziankowska-Zaborszczyk, Ricky Eddie, Ebrahim Eftekhar, Eruke E Egbagbe, Robert Eggertsen, Sareh Eghtesad, Gabriele Eiben, Ulf Ekelund, Mohammad El-Khateeb, Jalila El Ati, Denise Eldemire-Shearer, Marie Eliasen, Paul Elliott, Reina Engle-Stone, Macia Enguerran, Rajiv T Erasmus, Raimund Erbel, Cihangir Erem, Louise Eriksen, Johan G Eriksson, Jorge Escobedo-de la Peña, Saeid Eslami, Ali Esmaeili, Alun Evans, David Faeh, Albina A Fakhretdinova, Caroline H Fall, Elnaz Faramarzi, Mojtaba Farjam, Victoria Farrugia Sant'Angelo, Farshad Farzadfar, Mohammad Reza Fattahi, Asher Fawwad, Francisco J Felix-Redondo, Trevor S Ferguson, Romulo A Fernandes, Daniel Fernández-Bergés, Daniel Ferrante, Thomas Ferrao, Marika Ferrari, Marco M Ferrario, Catterina Ferreccio, Eldridge Ferrer, Jean Ferrieres, Thamara Hubler Figueiró, Anna Fijalkowska, Günther Fink, Krista Fischer, Leng Huat Foo, Maria Forsner, Heba M Fouad, Damian K Francis, Maria do Carmo Franco, Ruth Frikke-Schmidt, Guillermo Frontera, Flavio D Fuchs, Sandra C Fuchs, Isti I Fujiati, Yuki Fujita, Matsuda Fumihiko, Takuro Furusawa, Zbigniew Gaciong, Mihai Gafencu, Andrzej Galbarczyk, Henrike Galenkamp, Daniela Galeone, Myriam Galfo, Fabio Galvano, Jingli Gao, Manoli Garcia-de-la-Hera, Marta García-Solano, Dickman Gareta, Sarah P Garnett, Jean-Michel Gaspoz, Magda Gasull, Adroaldo Cesar Araujo Gaya, Anelise Reis Gaya, Andrea Gazzinelli, Ulrike Gehring, Harald Geiger, Johanna M Geleijnse, Ali Ghanbari, Erfan Ghasemi, Oana-Florentina Gheorghe-Fronea, Simona Giampaoli, Francesco Gianfagna, Tiffany K Gill, Jonathan Giovannelli, Glen Gironella, Aleksander Giwercman, Konstantinos Gkiouras, Justyna Godos, Sibel Gogen, Marcel Goldberg, Rebecca A Goldsmith, David Goltzman, Santiago F Gómez, Aleksandra Gomula, Bruna Goncalves Cordeiro da Silva, Helen Gonçalves, David A Gonzalez-Chica, Marcela Gonzalez-Gross, Margot González-Leon, Juan P González-Rivas, Clicerio González-Villalpando, María-Elena González-Villalpando, Angel R Gonzalez, Frederic Gottrand, Antonio Pedro Graça, Sidsel Graff-Iversen, Dušan Grafnetter, Aneta Grajda, Maria G Grammatikopoulou, Ronald D Gregor, Tomasz Grodzicki, Else Karin Grøholt, Anders Grøntved, Giuseppe Grosso, Gabriella Gruden, Dongfeng Gu, Emanuela Gualdi-Russo, Pilar Guallar-Castillón, Andrea Gualtieri, Elias F Gudmundsson, Vilmundur Gudnason, Ramiro Guerrero, Idris Guessous, Andre L Guimaraes, Martin C Gulliford, Johanna Gunnlaugsdottir, Marc J Gunter, Xiu-Hua Guo, Yin Guo, Prakash C Gupta, Rajeev Gupta, Oye Gureje, Beata Gurzkowska, Enrique Gutiérrez-González, Laura Gutierrez, Felix Gutzwiller, Seongjun Ha, Farzad Hadaegh, Charalambos A Hadjigeorgiou, Rosa Haghshenas, Hamid Hakimi, Jytte Halkjær, Ian R Hambleton, Behrooz Hamzeh, Dominique Hange, Abu AM Hanif, Sari Hantunen, Jie Hao, Rachakulla Hari Kumar, Seyed Mohammad Hashemi-Shahri, Maria Hassapidou, Jun Hata, Teresa Haugsgjerd, Jiang He, Yuan He, Yuna He, Regina Heidinger-Felso, Mirjam Heinen, Tatjana Hejgaard, Marleen Elisabeth Hendriks, Rafael dos Santos Henrique, Ana Henriques, Leticia Hernandez Cadena, Sauli Herrala, Victor M Herrera, Isabelle Herter-Aeberli, Ramin Heshmat, Allan G Hill, Sai Yin Ho, Suzanne C Ho, Michael Hobbs, Michelle Holdsworth, Reza Homayounfar, Clara Homs, Wilma M Hopman, Andrea RVR Horimoto, Claudia M Hormiga, Bernardo L Horta, Leila Houti, Christina Howitt, Thein Thein Htay, Aung Soe Htet, Maung Maung Than Htike, Yonghua Hu, José María Huerta, Ilpo Tapani Huhtaniemi, Laetitia Huiart, Constanta Huidumac Petrescu, Martijn Huisman, Abdullatif Husseini, Chinh Nguyen Huu, Inge Huybrechts, Nahla Hwalla, Jolanda Hyska, Licia Iacoviello, Jesús M Ibarluzea, Mohsen M Ibrahim, Norazizah Ibrahim Wong, M Arfan Ikram, Violeta Iotova, Vilma E Irazola, Takafumi Ishida, Muhammad Islam, Sheikh Mohammed Shariful Islam, Masanori Iwasaki, Jeremy M Jacobs, Hashem Y Jaddou, Tazeen Jafar, Kenneth James, Kazi M Jamil, Konrad Jamrozik, Imre Janszky, Edward Janus, Juel Jarani, Marjo-Riitta Jarvelin, Grazyna Jasienska, Ana Jelakovic, Bojan Jelakovic, Garry Jennings, Anjani Kumar Jha, Chao Qiang Jiang, Ramon O Jimenez, Karl-Heinz Jöckel, Michel Joffres, Mattias Johansson, Jari J Jokelainen, Jost B Jonas, Jitendra Jonnagaddala, Torben Jørgensen, Pradeep Joshi, Farahnaz Joukar, Dragana P Jovic, Jacek J Jóźwiak, Anne Juolevi, Gregor Jurak, Iulia Jurca Simina, Vesna Juresa, Rudolf Kaaks, Felix O Kaducu, Anthony Kafatos, Eero O Kajantie, Zhanna Kalmatayeva, Ofra Kalter-Leibovici, Yves Kameli, Freja B Kampmann, Kodanda R Kanala, Srinivasan Kannan, Efthymios Kapantais, Argyro Karakosta, Line L Kårhus, Khem B Karki, Marzieh Katibeh, Joanne Katz, Peter T Katzmarzyk, Jussi Kauhanen, Prabhdeep Kaur, Maryam Kavousi, Gyulli M Kazakbaeva, Ulrich Keil, Lital Keinan Boker, Sirkka Keinänen-Kiukaanniemi, Roya Kelishadi, Cecily Kelleher, Han CG Kemper, Andre P Kengne, Maryam Keramati, Alina Kerimkulova, Mathilde Kersting, Timothy Key, Yousef Saleh Khader, Davood Khalili, Kay-Tee Khaw, Bahareh Kheiri, Motahareh Kheradmand, Alireza Khosravi, Ilse MSL Khouw, Ursula Kiechl-Kohlendorfer, Stefan Kiechl, Japhet Killewo, Dong Wook Kim, Hyeon Chang Kim, Jeongseon Kim, Jenny M Kindblom, Heidi Klakk, Magdalena Klimek, Jeannette Klimont, Jurate Klumbiene, Michael Knoflach, Bhawesh Koirala, Elin Kolle, Patrick Kolsteren, Jürgen König, Raija Korpelainen, Paul Korrovits, Magdalena Korzycka, Jelena Kos, Seppo Koskinen, Katsuyasu Kouda, Viktoria A Kovacs, Sudhir Kowlessur, Slawomir Koziel, Jana Kratenova, Wolfgang Kratzer, Susi Kriemler, Peter Lund Kristensen, Steinar Krokstad, Daan Kromhout, Herculina S Kruger, Ruzena Kubinova, Renata Kuciene, Urho M Kujala, Enisa Kujundzic, Zbigniew Kulaga, R Krishna Kumar, Marie Kunešová, Pawel Kurjata, Yadlapalli S Kusuma, Kari Kuulasmaa, Catherine Kyobutungi, Quang Ngoc La, Fatima Zahra Laamiri, Tiina Laatikainen, Carl Lachat, Youcef Laid, Tai Hing Lam, Christina-Paulina Lambrinou, Edwige Landais, Vera Lanska, Georg Lappas, Bagher Larijani, Tint Swe Latt, Laura Lauria, Maria Lazo-Porras, Gwenaëlle Le Coroller, Khanh Le Nguyen Bao, Agnès Le Port, Tuyen D Le, Jeannette Lee, Jeonghee Lee, Paul H Lee, Nils Lehmann, Terho Lehtimäki, Daniel Lemogoum, Naomi S Levitt, Yanping Li, Merike Liivak, Christa L Lilly, Wei-Yen Lim, M Fernanda Lima-Costa, Hsien-Ho Lin, Xu Lin, Yi-Ting Lin, Lars Lind, Allan Linneberg, Lauren Lissner, Mieczyslaw Litwin, Lijuan Liu, Wei-Cheng Lo, Helle-Mai Loit, Khuong Quynh Long, Luis Lopes, Oscar Lopes, Esther Lopez-Garcia, Tania Lopez, Paulo A Lotufo, José Eugenio Lozano, Janice L Lukrafka, Dalia Luksiene, Annamari Lundqvist, Robert Lundqvist, Nuno Lunet, Charles Lunogelo, Michala Lustigová, Edyta Łuszczki, Guansheng Ma, Jun Ma, Xu Ma, George LL Machado-Coelho, Aristides M Machado-Rodrigues, Luisa M Macieira, Ahmed A Madar, Stefania Maggi, Dianna J Magliano, Sara Magnacca, Emmanuella Magriplis, Gowri Mahasampath, Bernard Maire, Marjeta Majer, Marcia Makdisse, Päivi Mäki, Fatemeh Malekzadeh, Reza Malekzadeh, Rahul Malhotra, Kodavanti Mallikharjuna Rao, Sofia K Malyutina, Lynell V Maniego, Yannis Manios, Jim I Mann, Fariborz Mansour-Ghanaei, Enzo Manzato, Paula Margozzini, Anastasia Markaki, Oonagh Markey, Eliza Markidou Ioannidou, Pedro Marques-Vidal, Larissa Pruner Marques, Jaume Marrugat, Yves Martin-Prevel, Rosemarie Martin, Reynaldo Martorell, Eva Martos, Katharina Maruszczak, Stefano Marventano, Luis P Mascarenhas, Shariq R Masoodi, Ellisiv B Mathiesen, Prashant Mathur, Alicia Matijasevich, Tandi E Matsha, Christina Mavrogianni, Artur Mazur, Jean Claude N Mbanya, Shelly R McFarlane, Stephen T McGarvey, Martin McKee, Stela McLachlan, Rachael M McLean, Scott B McLean, Breige A McNulty, Sounnia Mediene Benchekor, Jurate Medzioniene, Parinaz Mehdipour, Kirsten Mehlig, Amir Houshang Mehrparvar, Aline Meirhaeghe, Jørgen Meisfjord, Christa Meisinger, Ana Maria B Menezes, Geetha R Menon, Gert BM Mensink, Maria Teresa Menzano, Alibek Mereke, Indrapal I Meshram, Andres Metspalu, Haakon E Meyer, Jie Mi, Kim F Michaelsen, Nathalie Michels, Kairit Mikkel, Karolina Milkowska, Jody C Miller, Cláudia S Minderico, GK Mini, Juan Francisco Miquel, Mohammad Reza Mirjalili, Daphne Mirkopoulou, Erkin Mirrakhimov, Marjeta Mišigoj-Durakovic, Antonio Mistretta, Veronica Mocanu, Pietro A Modesti, Sahar Saeedi Moghaddam, Bahram Mohajer, Mostafa K Mohamed, Shukri F Mohamed, Kazem Mohammad, Zahra Mohammadi, Noushin Mohammadifard, Reza Mohammadpourhodki, Viswanathan Mohan, Salim Mohanna, Muhammad Fadhli Mohd Yusoff, Iraj Mohebbi, Farnam Mohebi, Marie Moitry, Drude Molbo, Line T Møllehave, Niels C Møller, Dénes Molnár, Amirabbas Momenan, Charles K Mondo, Michele Monroy-Valle, Eric Monterrubio-Flores, Kotsedi Daniel K Monyeki, Jin Soo Moon, Mahmood Moosazadeh, Leila B Moreira, Alain Morejon, Luis A Moreno, Karen Morgan, Suzanne N Morin, Erik Lykke Mortensen, George Moschonis, Malgorzata Mossakowska, Aya Mostafa, Anabela Mota-Pinto, Jorge Mota, Mohammad Esmaeel Motlagh, Jorge Motta, Marcos André Moura-dos-Santos, Malay K Mridha, Kelias P Msyamboza, Thet Thet Mu, Magdalena Muc, Boban Mugoša, Maria L Muiesan, Parvina Mukhtorova, Martina Müller-Nurasyid, Neil Murphy, Jaakko Mursu, Elaine M Murtagh, Kamarul Imran Musa, Sanja Music Milanovic, Vera Musil, Norlaila Mustafa, Iraj Nabipour, Shohreh Naderimagham, Gabriele Nagel, Balkish M Naidu, Farid Najafi, Harunobu Nakamura, Jana Námešná, Ei Ei K Nang, Vinay B Nangia, Martin Nankap, Sameer Narake, Paola Nardone, Matthias Nauck, William A Neal, Azim Nejatizadeh, Chandini Nekkantti, Keiu Nelis, Liis Nelis, Ilona Nenko, Martin Neovius, Flavio Nervi, Chung T Nguyen, Nguyen D Nguyen, Quang Ngoc Nguyen, Ramfis E Nieto-Martínez, Yury P Nikitin, Guang Ning, Toshiharu Ninomiya, Sania Nishtar, Marianna Noale, Oscar A Noboa, Helena Nogueira, Teresa Norat, Maria Nordendahl, Børge G Nordestgaard, Davide Noto, Natalia Nowak-Szczepanska, Mohannad Al Nsour, Irfan Nuhoglu, Eha Nurk, Terence W O'Neill, Dermot O'Reilly, Galina Obreja, Caleb Ochimana, Angélica M Ochoa-Avilés, Eiji Oda, Kyungwon Oh, Kumiko Ohara, Claes Ohlsson, Ryutaro Ohtsuka, Örn Olafsson, Maria Teresa A Olinto, Isabel O Oliveira, Mohd Azahadi Omar, Altan Onat, Sok King Ong, Lariane M Ono, Pedro Ordunez, Rui Ornelas, Ana P Ortiz, Pedro J Ortiz, Merete Osler, Clive Osmond, Sergej M Ostojic, Afshin Ostovar, Johanna A Otero, Kim Overvad, Ellis Owusu-Dabo, Fred Michel Paccaud, Cristina Padez, Ioannis Pagkalos, Elena Pahomova, Karina Mary de Paiva, Andrzej Pajak, Domenico Palli, Alberto Palloni, Luigi Palmieri, Wen-Harn Pan, Songhomitra Panda-Jonas, Arvind Pandey, Francesco Panza, Dimitrios Papandreou, Soon-Woo Park, Suyeon Park, Winsome R Parnell, Mahboubeh Parsaeian, Ionela M Pascanu, Patrick Pasquet, Nikhil D Patel, Ivan Pecin, Mangesh S Pednekar, Nasheeta Peer, Gao Pei, Sergio Viana Peixoto, Markku Peltonen, Alexandre C Pereira, Marco A Peres, Napoleón Pérez-Farinós, Cynthia M Pérez, Valentina Peterkova, Annette Peters, Astrid Petersmann, Janina Petkeviciene, Ausra Petrauskiene, Emanuela Pettenuzzo, Niloofar Peykari, Son Thai Pham, Rafael N Pichardo, Daniela Pierannunzio, Iris Pigeot, Hynek Pikhart, Aida Pilav, Lorenza Pilotto, Francesco Pistelli, Freda Pitakaka, Aleksandra Piwonska, Andreia N Pizarro, Pedro Plans-Rubió, Bee Koon Poh, Hermann Pohlabeln, Raluca M Pop, Stevo R Popovic, Miquel Porta, Georg Posch, Anil Poudyal, Dimitrios Poulimeneas, Hamed Pouraram, Farhad Pourfarzi, Akram Pourshams, Hossein Poustchi, Rajendra Pradeepa, Alison J Price, Jacqueline F Price, Rui Providencia, Jardena J Puder, Iveta Pudule, Soile E Puhakka, Maria Puiu, Margus Punab, Radwan F Qasrawi, Mostafa Qorbani, Tran Quoc Bao, Ivana Radic, Ricardas Radisauskas, Salar Rahimikazerooni, Mahfuzar Rahman, Mahmudur Rahman, Olli Raitakari, Manu Raj, Ellina Rakhimova, Sherali Rakhmatulloev, Ivo Rakovac, Sudha Ramachandra Rao, Ambady Ramachandran, Jacqueline Ramke, Elisabete Ramos, Rafel Ramos, Lekhraj Rampal, Sanjay Rampal, Vayia Rarra, Ramon A Rascon-Pacheco, Mette Rasmussen, Cassiano Ricardo Rech, Josep Redon, Paul Ferdinand M Reganit, Valéria Regecová, Luis Revilla, Abbas Rezaianzadeh, Lourdes Ribas-Barba, Robespierre Ribeiro, Elio Riboli, Adrian Richter, Fernando Rigo, Natascia Rinaldo, Tobias F Rinke de Wit, Ana Rito, Raphael M Ritti-Dias, Juan A Rivera, Cynthia Robitaille, Romana Roccaldo, Daniela Rodrigues, Fernando Rodríguez-Artalejo, María del Cristo Rodriguez-Perez, Laura A Rodríguez-Villamizar, Ulla Roggenbuck, Rosalba Rojas-Martinez, Nipa Rojroongwasinkul, Dora Romaguera, Elisabetta L Romeo, Rafaela V Rosario, Annika Rosengren, Ian Rouse, Joel GR Roy, Adolfo Rubinstein, Frank J Rühli, Jean-Bernard Ruidavets, Blanca Sandra Ruiz-Betancourt, Maria Ruiz-Castell, Emma Ruiz Moreno, Iuliia A Rusakova, Kenisha Russell Jonsson, Paola Russo, Petra Rust, Marcin Rutkowski, Charumathi Sabanayagam, Elena Sacchini, Harshpal S Sachdev, Alireza Sadjadi, Ali Reza Safarpour, Saeid Safiri, Nader Saki, Benoit Salanave, Eduardo Salazar Martinez, Diego Salmerón, Veikko Salomaa, Jukka T Salonen, Massimo Salvetti, Margarita Samoutian, Jose Sánchez-Abanto, Sandjaja, Susana Sans, Loreto Santa Marina, Diana A Santos, Ina S Santos, Lèlita C Santos, Maria Paula Santos, Osvaldo Santos, Rute Santos, Sara Santos Sanz, Jouko L Saramies, Luis B Sardinha, Nizal Sarrafzadegan, Thirunavukkarasu Sathish, Kai-Uwe Saum, Savvas Savva, Mathilde Savy, Norie Sawada, Mariana Sbaraini, Marcia Scazufca, Beatriz D Schaan, Angelika Schaffrath Rosario, Herman Schargrodsky, Anja Schienkiewitz, Sabine Schipf, Carsten O Schmidt, Ida Maria Schmidt, Peter Schnohr, Ben Schöttker, Sara Schramm, Stine Schramm, Helmut Schröder, Constance Schultsz, Aletta E Schutte, Aye Aye Sein, Rusidah Selamat, Vedrana Sember, Abhijit Sen, Idowu O Senbanjo, Sadaf G Sepanlou, Victor Sequera, Luis Serra-Majem, Jennifer Servais, Ludmila Ševcíková, Svetlana A Shalnova, Teresa Shamah-Levy, Morteza Shamshirgaran, Coimbatore Subramaniam Shanthirani, Maryam Sharafkhah, Sanjib K Sharma, Jonathan E Shaw, Amaneh Shayanrad, Ali Akbar Shayesteh, Lela Shengelia, Zumin Shi, Kenji Shibuya, Hana Shimizu-Furusawa, Dong Wook Shin, Majid Shirani, Rahman Shiri, Namuna Shrestha, Khairil Si-Ramlee, Alfonso Siani, Rosalynn Siantar, Abla M Sibai, Antonio M Silva, Diego Augusto Santos Silva, Mary Simon, Judith Simons, Leon A Simons, Agneta Sjöberg, Michael Sjöström, Gry Skodje, Jolanta Slowikowska-Hilczer, Przemyslaw Slusarczyk, Liam Smeeth, Hung-Kwan So, Fernanda Cunha Soares, Grzegorz Sobek, Eugène Sobngwi, Morten Sodemann, Stefan Söderberg, Moesijanti YE Soekatri, Agustinus Soemantri, Reecha Sofat, Vincenzo Solfrizzi, Mohammad Hossein Somi, Emily Sonestedt, Yi Song, Thorkild IA Sørensen, Elin P Sørgjerd, Charles Sossa Jérome, Victoria E Soto-Rojas, Aïcha Soumaré, Slavica Sovic, Bente Sparboe-Nilsen, Karen Sparrenberger, Angela Spinelli, Igor Spiroski, Jan A Staessen, Hanspeter Stamm, Maria G Stathopoulou, Kaspar Staub, Bill Stavreski, Jostein Steene-Johannessen, Peter Stehle, Aryeh D Stein, George S Stergiou, Jochanan Stessman, Ranko Stevanovic, Jutta Stieber, Doris Stöckl, Tanja Stocks, Jakub Stokwiszewski, Ekaterina Stoyanova, Gareth Stratton, Karien Stronks, Maria Wany Strufaldi, Lela Sturua, Ramón Suárez-Medina, Machi Suka, Chien-An Sun, Johan Sundström, Yn-Tz Sung, Jordi Sunyer, Paibul Suriyawongpaisal, Boyd A Swinburn, Rody G Sy, Holly E Syddall, René Charles Sylva, Moyses Szklo, Lucjan Szponar, E Shyong Tai, Mari-Liis Tammesoo, Abdonas Tamosiunas, Eng Joo Tan, Xun Tang, Maya Tanrygulyyeva, Frank Tanser, Yong Tao, Mohammed Rasoul Tarawneh, Jakob Tarp, Carolina B Tarqui-Mamani, Radka Taxová Braunerová, Anne Taylor, Julie Taylor, Félicité Tchibindat, William R Tebar, Grethe S Tell, Tania Tello, Yih Chung Tham, KR Thankappan, Holger Theobald, Xenophon Theodoridis, Lutgarde Thijs, Nihal Thomas, Betina H Thuesen, Lubica Tichá, Erik J Timmermans, Anne Tjonneland, Hanna K Tolonen, Janne S Tolstrup, Murat Topbas, Roman Topór-Madry, Liv Elin Torheim, María José Tormo, Michael J Tornaritis, Maties Torrent, Laura Torres-Collado, Stefania Toselli, Giota Touloumi, Pierre Traissac, Thi Tuyet-Hanh Tran, Dimitrios Trichopoulos, Antonia Trichopoulou, Oanh TH Trinh, Atul Trivedi, Lechaba Tshepo, Maria Tsigga, Shoichiro Tsugane, Azaliia M Tuliakova, Marshall K Tulloch-Reid, Fikru Tullu, Tomi-Pekka Tuomainen, Jaakko Tuomilehto, Maria L Turley, Gilad Twig, Per Tynelius, Themistoklis Tzotzas, Christophe Tzourio, Peter Ueda, Eunice Ugel, Flora AM Ukoli, Hanno Ulmer, Belgin Unal, Zhamyila Usupova, Hannu MT Uusitalo, Nalan Uysal, Justina Vaitkeviciute, Gonzalo Valdivia, Susana Vale, Damaskini Valvi, Rob M van Dam, Johan Van der Heyden, Yvonne T van der Schouw, Koen Van Herck, Hoang Van Minh, Natasja M Van Schoor, Irene GM van Valkengoed, Dirk Vanderschueren, Diego Vanuzzo, Anette Varbo, Gregorio Varela-Moreiras, Patricia Varona-Pérez, Senthil K Vasan, Tomas Vega, Toomas Veidebaum, Gustavo Velasquez-Melendez, Biruta Velika, Giovanni Veronesi, WM Monique Verschuren, Cesar G Victora, Giovanni Viegi, Lucie Viet, Salvador Villalpando, Paolo Vineis, Jesus Vioque, Jyrki K Virtanen, Marjolein Visser, Sophie Visvikis-Siest, Bharathi Viswanathan, Mihaela Vladulescu, Tiina Vlasoff, Dorja Vocanec, Peter Vollenweider, Henry Völzke, Ari Voutilainen, Sari Voutilainen, Martine Vrijheid, Tanja GM Vrijkotte, Alisha N Wade, Aline Wagner, Thomas Waldhör, Janette Walton, Elvis OA Wambiya, Wan Mohamad Wan Bebakar, Wan Nazaimoon Wan Mohamud, Rildo de Souza Wanderley Júnior, Ming-Dong Wang, Ningli Wang, Qian Wang, Xiangjun Wang, Ya Xing Wang, Ying-Wei Wang, S Goya Wannamethee, Nicholas Wareham, Adelheid Weber, Niels Wedderkopp, Deepa Weerasekera, Daniel Weghuber, Wenbin Wei, Aneta Weres, Bo Werner, Peter H Whincup, Kurt Widhalm, Indah S Widyahening, Andrzej Wiecek, Rainford J Wilks, Johann Willeit, Peter Willeit, Julianne Williams, Tom Wilsgaard, Bogdan Wojtyniak, Roy A Wong-McClure, Andrew Wong, Jyh Eiin Wong, Tien Yin Wong, Jean Woo, Mark Woodward, Frederick C Wu, Jianfeng Wu, Li Juan Wu, Shouling Wu, Haiquan Xu, Liang Xu, Nor Azwany Yaacob, Uruwan Yamborisut, Weili Yan, Ling Yang, Xiaoguang Yang, Yang Yang, Nazan Yardim, Mehdi Yaseri, Tabara Yasuharu, Xingwang Ye, Panayiotis K Yiallouros, Moein Yoosefi, Akihiro Yoshihara, Qi Sheng You, San-Lin You, Novie O Younger-Coleman, Safiah Md Yusof, Ahmad Faudzi Yusoff, Luciana Zaccagni, Vassilis Zafiropulos, Ahmad A Zainuddin, Seyed Rasoul Zakavi, Farhad Zamani, Sabina Zambon, Antonis Zampelas, Hana Zamrazilová, Maria Elisa Zapata, Abdul Hamid Zargar, Ko Ko Zaw, Tomasz Zdrojewski, Kristyna Zejglicova, Tajana Zeljkovic Vrkic, Yi Zeng, Luxia Zhang, Zhen-Yu Zhang, Dong Zhao, Ming-Hui Zhao, Wenhua Zhao, Shiqi Zhen, Wei Zheng, Yingfeng Zheng, Bekbolat Zholdin, Maigeng Zhou, Dan Zhu, Marie Zins, Emanuel Zitt, Yanina Zocalo, Julio Zuñiga Cisneros, Monika Zuziak, Majid Ezzati, Sarah Filippi

**Affiliations:** Imperial College LondonLondonUnited Kingdom; Imperial College LondonLondonUnited Kingdom; Imperial College LondonLondonUnited Kingdom; Imperial College LondonLondonUnited Kingdom; Imperial College LondonLondonUnited Kingdom; Imperial College LondonLondonUnited Kingdom; Imperial College LondonLondonUnited Kingdom; Imperial College LondonLondonUnited Kingdom; Imperial College LondonLondonUnited Kingdom; Harvard TH Chan School of Public HealthBostonUnited States; Middlesex UniversityLondonUnited Kingdom; Independent researcherLos AngelesUnited States; Imperial College LondonLondonUnited Kingdom; World Health OrganizationGenevaSwitzerland; World Health OrganizationGenevaSwitzerland; World Health OrganizationGenevaSwitzerland; Ministry of HealthVictoriaSeychelles; University of LausanneLausanneSwitzerland; Eduardo Mondlane UniversityMaputoMozambique; Victor Babes University of Medicine and Pharmacy TimisoaraTimisoaraRomania; University of SydneySydneyAustralia; National Institutes of Biomedical Innovation, Health and NutritionTokyoJapan; University of AucklandAucklandNew Zealand; Seoul National UniversitySeoulRepublic of Korea; ICMR - National Institute of NutritionHyderabadIndia; Capital Medical University Beijing An Zhen HospitalBeijingChina; Universidad Peruana Cayetano HerediaLimaPeru; University Tunis El ManarTunisTunisia; University of OuluOuluFinland; University of ZagrebZagrebCroatia; University of LjubljanaLjubljanaSlovenia; Imperial College LondonLondonUnited Kingdom; Caja Costarricense de Seguro SocialSan JoséCosta Rica; Al-Quds UniversityEast JerusalemState of Palestine; National Center of Public HealthcareNur-SultanKazakhstan; Ministry of HealthKuala LumpurMalaysia; Qatar UniversityDohaQatar; Birzeit UniversityBirzeitState of Palestine; Usmanu Danfodiyo University Teaching HospitalSokotoNigeria; Instituto Mexicano del Seguro SocialMexico CityMexico; Flinders UniversityAdelaideAustralia; Mahidol UniversityNakhon PathomThailand; BRAC James P Grant School of Public HealthDhakaBangladesh; University of CopenhagenCopenhagenDenmark; Copenhagen University HospitalCopenhagenDenmark; Food and Nutrition Research InstituteTaguigPhilippines; Urmia University of Medical SciencesUrmiaIslamic Republic of Iran; Instituto Nacional de Ciencias Médicas y NutriciónMexico CityMexico; University of AmsterdamAmsterdamNetherlands; Ministry of HealthKuala LumpurMalaysia; Ministry of HealthKuala LumpurMalaysia; Shahrekord University of Medical SciencesShahrekordIslamic Republic of Iran; Non-Communicable Diseases Research CenterTehranIslamic Republic of Iran; University of OsloOsloNorway; University of BremenBremenGermany; Republican Center for Health PromotionBishkekKyrgyzstan; National Center for Diabetes, Endocrinology and GeneticsAmmanJordan; Princess Nourah bint Abdulrahman UniversityRiyadhSaudi Arabia; Kuwait Institute for Scientific ResearchKuwait CityKuwait; King Abdulaziz UniversityJeddahSaudi Arabia; Dasman Diabetes InstituteKuwait CityKuwait; Aldara Hospital and Medical CenterRiyadhSaudi Arabia; King Abdullah International Medical Research CenterRiyadhSaudi Arabia; World Health OrganizationGenevaSwitzerland; Dasman Diabetes InstituteKuwait CityKuwait; Luxembourg Institute of HealthStrassenLuxembourg; Bispebjerg and Frederiksberg HospitalCopenhagenDenmark; Barcelona Institute for Global Health CIBERESPBarcelonaSpain; World Health Organization Regional Office for the Eastern MediterraneanCairoEgypt; Bombay Hospital and Medical Research CentreMumbaiIndia; Research Center for Social Determinants of HealthTehranIslamic Republic of Iran; UMR CNRS-MNHN 7206 Eco-anthropologieParisFrance; University of LilleFrance; Lille University HospitalLilleFrance; Western Norway University of Applied SciencesSogndalNorway; Norwegian School of Sport SciencesOsloNorway; University of CopenhagenCopenhagenDenmark; Madras Diabetes Research FoundationChennaiIndia; Zahedan University of Medical SciencesZahedanIslamic Republic of Iran; National Institute of Public HealthTunisTunisia; Institute of Public Health of the University of PortoPortoPortugal; Norwegian Institute of Public HealthOsloNorway; Ministry of HealthKuala LumpurMalaysia; University of Massachusetts AmherstAmherstUnited States; ICMR - National Institute of NutritionHyderabadIndia; Abt AssociatesKathmanduNepal; University of IcelandReykjavikIceland; University of Yaoundé 1YaoundéCameroon; Federal University of PelotasPelotasBrazil; University of Medicine 1YangonMyanmar; University of OuluOuluFinland; Oulu University HospitalOuluFinland; Banska Bystrica Regional Authority of Public HealthBanska BystricaSlovakia; Tel-Aviv UniversityTel-AvivIsrael; Hebrew University of JerusalemJerusalemIsrael; University of Porto Medical SchoolPortoPortugal; Neyshabur University of Medical SciencesNeyshaburIslamic Republic of Iran; Research Institute for Endocrine SciencesTehranIslamic Republic of Iran; Non-Communicable Diseases Research CenterTehranIslamic Republic of Iran; Indian Council of Medical ResearchNew DelhiIndia; National Institute of Public HealthCopenhagenDenmark; Ministry of HealthKuala LumpurMalaysia; King Abdulaziz UniversityJeddahSaudi Arabia; Bispebjerg and Frederiksberg HospitalCopenhagenDenmark; ICMR - National Institute of NutritionHyderabadIndia; University of Science and TechnologySana'aYemen; Medical University of LodzLodzPoland; Medical University of GdanskGdanskPoland; Universidad Autónoma de Madrid CIBERESPMadridSpain; University of RzeszówRzeszówPoland; University of PalermoPalermoItaly; Pan American Health OrganizationWashington DCUnited States; Mohammed V University de RabatRabatMorocco; Federal University of PelotasPelotasBrazil; University of PernambucoRecifeBrazil; Baqai Institute of Diabetology and EndocrinologyKarachiPakistan; Federal University of Santa CatarinaFlorianópolisBrazil; Dalhousie UniversityHalifaxCanada; Jordan University of Science and TechnologyIrbidJordan; Federal University of MaranhãoSão LuísBrazil; National Center of Public HealthcareNur-SultanKazakhstan; Al-Farabi Kazakh National UniversityAlmatyKazakhstan; University of SydneySydneyAustralia; University of AucklandAucklandNew Zealand; University College DublinDublinIreland; Christian Medical CollegeVelloreIndia; University Tunis El ManarTunisTunisia; Federal Ministry of Social Affairs, Health, Care and Consumer ProtectionViennaAustria; Cafam University FoundationBogotaColombia; Norwegian Institute of Public HealthOsloNorway; Kazakh National Medical UniversityAlmatyKazakhstan; Universidad Peruana Cayetano HerediaLimaPeru; Lithuanian University of Health SciencesKaunasLithuania; University of São PauloSão PauloBrazil; University of PernambucoRecifeBrazil; B J Medical CollegeAhmedabadIndia; Chirayu Medical CollegeNew DelhiIndia; Sunder Lal Jain HospitalDelhiIndia; The Hospital for Sick ChildrenTorontoCanada; Aga Khan UniversityKarachiPakistan; Shandong University of Traditional Chinese MedicineJinanChina; Shanghai Jiao-Tong University School of MedicineShanghaiChina; Universidad de la RepúblicaMontevideoUruguay; Institute of Medical Research and Medicinal Plant StudiesYaoundéCameroon; Ufa Eye Research InstituteUfaRussian Federation; Nepal Health Research CouncilKathmanduNepal; University of MontenegroNiksicMontenegro; University of Southern DenmarkCopenhagenDenmark; University of OsloOsloNorway; University of OsloOsloNorway; University of GothenburgGothenburgSweden; Universidade Federal do Rio de JaneiroRio de JaneiroBrazil; National Institute for Public Health and the EnvironmentBilthovenNetherlands; University of TurinTurinItaly; University College LondonLondonUnited Kingdom; Liverpool John Moores UniversityLiverpoolUnited Kingdom; Nanyang Technological University SingaporeSingaporeSingapore; German Institute of Human NutritionPotsdamGermany; Universidad de la RepúblicaMontevideoUruguay; Endocrinology Research CentreMoscowRussian Federation; Centro de Educación Médica e Investigaciones ClínicasBuenos AiresArgentina; Copenhagen University HospitalCopenhagenDenmark; University of CopenhagenCopenhagenDenmark; IRCCS NeuromedPozzilliItaly; Toulouse University School of MedicineToulouseFrance; Caja Costarricense de Seguro SocialSan JoséCosta Rica; University of ZurichZurichSwitzerland; University Hospital KU LeuvenLeuvenBelgium; Flemish Agency for Care and HealthBrusselsBelgium; Ghent UniversityGhentBelgium; FrieslandCampinaAmersfoortNetherlands; Universidad Central de VenezuelaCaracasVenezuela; World Health OrganizationGenevaSwitzerland; Bielefeld UniversityBielefeldGermany; World Health Organization Regional Office for EuropeMoscowRussian Federation; German Cancer Research CenterHeidelbergGermany; University of AmsterdamAmsterdamNetherlands; The Fred Hollows FoundationAucklandNew Zealand; University of Medicine and Pharmacy BucharestBucharestRomania; Swansea UniversitySwanseaUnited Kingdom; University of TurinTurinItaly; National Institute for Public Health and the EnvironmentBilthovenNetherlands; University College CopenhagenCopenhagenDenmark; World Health Organization Regional Office for EuropeMoscowRussian Federation; Institute of Public HealthTiranaAlbania; Munster Technological UniversityCorkIreland; Universidad de La LagunaTenerifeSpain; University of MaltaMsidaMalta; Vanderbilt UniversityNashvilleUnited States; Ministry of HealthTongatapuTonga; Canadian Fitness and Lifestyle Research InstituteOttawaCanada; Hospital Santa MariaLisbonPortugal; Istanbul University - CerrahpasaIstanbulTurkey; Universidade Federal de Juiz de ForaJuiz de ForaBrazil; Ministry of Public HealthAsunciónParaguay; Food and Nutrition Research InstituteTaguigPhilippines; National Institute of Public HealthPragueCzech Republic; Gaetano Fucito HospitalMercato San SeverinoItaly; Gaetano Fucito HospitalMercato San SeverinoItaly; University of GroningenGroningenNetherlands; University of São PauloSão PauloBrazil; Karolinska InstitutetHuddingeSweden; Centro de Estudios sobre Nutrición InfantilBuenos AiresArgentina; University of PortoPortoPortugal; University of ZaragozaZaragozaSpain; Santiago de Compostela UniversitySantiago de CompostelaSpain; Ministry of HealthAnkaraTurkey; Council for Agricultural Research and EconomicsRomeItaly; Caja Costarricense de Seguro SocialSan JoséCosta Rica; Federal University of Rio GrandeRio GrandeBrazil; India Diabetes Research FoundationChennaiIndia; Duke-NUS Medical SchoolSingaporeSingapore; Imperial College LondonLondonUnited Kingdom; ICMR - National Institute of Medical StatisticsNew DelhiIndia; University College LondonLondonUnited Kingdom; Ministry of HealthKuala LumpurMalaysia; Singapore Eye Research InstituteSingaporeSingapore; Academia SinicaTaipeiTaiwan; Capital Institute of PediatricsBeijingChina; Duke UniversityDurhamUnited States; Kailuan General HospitalTangshanChina; University of OxfordOxfordUnited Kingdom; Duke-NUS Medical SchoolSingaporeSingapore; Ahvaz Jundishapur University of Medical SciencesAhvazIslamic Republic of Iran; The Gertner Institute for Epidemiology and Health Policy ResearchRamat GanIsrael; National Centre of Public Health and AnalysesSofiaBulgaria; University of FribourgFribourgSwitzerland; Ministry of Health and WelfareTaipeiTaiwan; CIBER Epidemiología y Salud PúblicaMurciaSpain; Seoul National UniversitySeoulRepublic of Korea; University of Southern DenmarkOdenseDenmark; Universidade Estadual PaulistaPresidente PrudenteBrazil; Medical University of SilesiaKatowicePoland; Charles UniversityPragueCzech Republic; Thomayer HospitalPragueCzech Republic; Primary Health CareFlorianaMalta; Carol Davila University of Medicine and PharmacyBucharestRomania; Katholieke Universiteit LeuvenLeuvenBelgium; Statistics CanadaOttawaCanada; Ghent UniversityGhentBelgium; UMR CNRS-MNHN 7206 Eco-anthropologieMarseilleFrance; Agency for Preventive and Social MedicineBregenzAustria; Federal University of MaranhãoSão LuísBrazil; University of SouthamptonSouthamptonUnited Kingdom; Munster Technological UniversityCorkIreland; University of GroningenGroningenNetherlands; IRCCS NeuromedPozzilliItaly; Institut Pasteur de LilleLilleFrance; University of SydneySydneyAustralia; Canadian Fitness and Lifestyle Research InstituteOttawaCanada; Malawi Epidemiology and Intervention Research UnitLilongweMalawi; CIBEROBNMadridSpain; University of DebrecenDebrecenHungary; University of Medicine and Pharmacy Carol DavilaBucharestRomania; Kailuan General HospitalTangshanChina; Universidade Federal do Rio Grande do SulPorto AlegreBrazil; University of RzeszówRzeszówPoland; National Research CouncilReggio CalabriaItaly; Federal University of Santa CatarinaFlorianópolisBrazil; Eftimie Murgu University ResitaResitaRomania; Spanish Agency for Food Safety and NutritionMadridSpain; Institut Pasteur de LilleLilleFrance; University of CopenhagenCopenhagenDenmark; The Gertner Institute for Epidemiology and Health Policy ResearchRamat GanIsrael; Bispebjerg and Frederiksberg HospitalCopenhagenDenmark; Indian Statistical InstituteKolkataIndia; Tabriz Health Services Management Research CenterTabrizIslamic Republic of Iran; University of LilleLilleFrance; Lille University HospitalLilleFrance; Al-Farabi Kazakh National UniversityAlmatyKazakhstan; Ghent UniversityGhentBelgium; Ghent UniversityGhentBelgium; IRCCS NeuromedPozzilliItaly; Ghent UniversityGhentBelgium; Federal University of PelotasPelotasBrazil; Geneva University HospitalsGenevaSwitzerland; SciensanoBrusselsBelgium; University Medical CentersGroningenNetherlands; University of AmsterdamAmsterdamNetherlands; Ghent UniversityGhentBelgium; Madras Diabetes Research FoundationChennaiIndia; National Research Centre for Preventive MedicineMoscowRussian Federation; Innovating Health InternationalPort-au-PrinceHaiti; Imperial College LondonLondonUnited Kingdom; University of MontrealMontrealCanada; French National Research Institute for Sustainable DevelopmentMontpellierFrance; SciensanoBrusselsBelgium; University of SouthamptonSouthamptonUnited Kingdom; University of RzeszówRzeszówPoland; French Public Health AgencySt MauriceFrance; Nepal Health Research CouncilKathmanduNepal; Mediterranea CardiocentroNaplesItaly; Universidade do Vale do Rio dos SinosSão LeopoldoBrazil; National Institute of Hygiene, Epidemiology and MicrobiologyHavanaCuba; National Council of Scientific and Technical ResearchBuenos AiresArgentina; University of ZagrebZagrebCroatia; Ministry of Health and Medical EducationTehranIslamic Republic of Iran; University of Novi SadNovi SadSerbia; National Institute of NutritionHanoiViet Nam; University of QueenslandBrisbaneAustralia; IRCCS NeuromedPozzilliItaly; Istituto Superiore di SanitàRomeItaly; Universidad de CuencaCuencaEcuador; Helmholtz Zentrum MünchenMunichGermany; Carol Davila University of Medicine and PharmacyBucharestRomania; Tehran University of Medical SciencesTehranIslamic Republic of Iran; Ministère de la Santé et de l'Hygiène PubliqueAbidjanCôte d'Ivoire; University Hospital DüsseldorfDüsseldorfGermany; National Institute of CardiologyWarsawPoland; Medical University of LodzLodzPoland; Beijing Center for Disease Prevention and ControlBeijingChina; Food and Nutrition Research InstituteTaguigPhilippines; UMI 3189 ESSMarseilleFrance; Beth Israel Deaconess Medical CenterBostonUnited States; Harvard Medical SchoolBostonUnited States; National Centre of Public Health and AnalysesSofiaBulgaria; Lithuanian University of Health SciencesKaunasLithuania; Federal University of Rio GrandeRio GrandeBrazil; Scuola Superiore Sant'AnnaPisaItaly; Al-Farabi Kazakh National UniversityAlmatyKazakhstan; University of LatviaRigaLatvia; Medical University of LodzLodzPoland; Ministry of Health and Medical ServicesGizoSolomon Islands; Hormozgan University of Medical SciencesBandar AbbasIslamic Republic of Iran; University of BeninBenin CityNigeria; University of GothenburgGothenburgSweden; Tehran University of Medical SciencesTehranIslamic Republic of Iran; University of SkövdeSkövdeSweden; Norwegian School of Sport SciencesOsloNorway; National Center for Diabetes, Endocrinology and GeneticsAmmanJordan; National Institute of Nutrition and Food TechnologyTunisTunisia; The University of the West IndiesKingstonJamaica; Bispebjerg and Frederiksberg HospitalCopenhagenDenmark; Imperial College LondonLondonUnited Kingdom; University of California DavisDavisUnited States; UMI 3189 ESSMarseilleFrance; University of StellenboschCape TownSouth Africa; University of Duisburg-EssenDuisburgGermany; Karadeniz Technical UniversityTrabzonTurkey; University of Southern DenmarkCopenhagenDenmark; University of HelsinkiHelsinkiFinland; Instituto Mexicano del Seguro SocialMexico CityMexico; Mashhad University of Medical SciencesMashhadIslamic Republic of Iran; Rafsanjan University of Medical SciencesRafsanjanIslamic Republic of Iran; Queen's University of BelfastBelfastUnited Kingdom; University of ZurichZurichSwitzerland; Ufa Eye Research InstituteUfaRussian Federation; University of SouthamptonSouthamptonUnited Kingdom; Tabriz University of Medical SciencesTabrizIslamic Republic of Iran; Fasa University of Medical SciencesFasaIslamic Republic of Iran; Primary Health CareFlorianaMalta; Non-Communicable Diseases Research CenterTehranIslamic Republic of Iran; Shiraz University of Medical SciencesShirazIslamic Republic of Iran; Baqai Medical UniversityKarachiPakistan; Centro de Salud Villanueva NorteBadajozSpain; The University of the West IndiesKingstonJamaica; Universidade Estadual PaulistaPresidente PrudenteBrazil; Hospital Don Benito-Villanueva de la SerenaBadajozSpain; Ministry of HealthBuenos AiresArgentina; Statistics CanadaOttawaCanada; Council for Agricultural Research and EconomicsRomeItaly; University of InsubriaVareseItaly; Pontificia Universidad Católica de ChileSantiagoChile; Food and Nutrition Research InstituteTaguigPhilippines; Toulouse University School of MedicineToulouseFrance; Federal University of Santa CatarinaFlorianópolisBrazil; Institute of Mother and ChildWarsawPoland; Swiss Tropical and Public Health InstituteBaselSwitzerland; University of BaselBaselSwitzerland; University of TartuTartuEstonia; Universiti Sains MalaysiaKelantanMalaysia; Umeå UniversityUmeåSweden; World Health Organization Regional Office for the Eastern MediterraneanCairoEgypt; The University of the West IndiesKingstonJamaica; Federal University of São PauloSão PauloBrazil; University of CopenhagenCopenhagenDenmark; Copenhagen University HospitalCopenhagenDenmark; Hospital Universitario Son EspasesPalmaSpain; Hospital de Clinicas de Porto AlegrePorto AlegreBrazil; Universidade Federal do Rio Grande do SulPorto AlegreBrazil; Universitas Sumatera UtaraMedanIndonesia; Kindai UniversityOsaka-SayamaJapan; Kyoto UniversityKyotoJapan; Kyoto UniversityKyotoJapan; Medical University of WarsawWarsawPoland; Victor Babes University of Medicine and Pharmacy TimisoaraTimisoaraRomania; Jagiellonian University Medical CollegeKrakówPoland; University of AmsterdamAmsterdamNetherlands; Ministero della Salute DG Prevenzione SanitariaRomeItaly; Council for Agricultural Research and EconomicsRomeItaly; University of CataniaCataniaItaly; Kailuan General HospitalTangshanChina; CIBER en Epidemiología y Salud PúblicaAlicanteSpain; Spanish Agency for Food Safety and NutritionMadridSpain; Africa Health Research InstituteMtubatubaSouth Africa; University of SydneySydneyAustralia; Geneva University Medical SchoolGenevaSwitzerland; CIBER en Epidemiología y Salud PúblicaBarcelonaSpain; Universidade Federal do Rio Grande do SulPorto AlegreBrazil; Universidade Federal do Rio Grande do SulPorto AlegreBrazil; Universidade Federal de Minas GeraisBelo HorizonteBrazil; Utrecht UniversityUtrechtNetherlands; Agency for Preventive and Social MedicineBregenzAustria; Wageningen UniversityWageningenNetherlands; Non-Communicable Diseases Research CenterTehranIslamic Republic of Iran; Non-Communicable Diseases Research CenterTehranIslamic Republic of Iran; Carol Davila University of Medicine and PharmacyBucharestRomania; Istituto Superiore di SanitàRomeItaly; University of InsubriaVareseItaly; Mediterranea CardiocentroNaplesItaly; University of AdelaideAdelaideAustralia; University of LilleLilleFrance; Lille University HospitalLilleFrance; Food and Nutrition Research InstituteTaguigPhilippines; Lund UniversityLundSweden; Aristotle University of ThessalonikiThessalonikiGreece; University of CataniaCataniaItaly; Ministry of HealthAnkaraTurkey; Institut National de la Santé et de la Recherche MédicaleVillejuifFrance; Paris UniversityParisFrance; Ministry of HealthJerusalemIsrael; McGill UniversityMontrealCanada; Gasol FoundationBarcelonaSpain; University of LleidaSant Boi de LlobregatSpain; PASs Hirszfeld Institute of Immunology and Experimental TherapyWroclawPoland; Federal University of PelotasPelotasBrazil; Federal University of PelotasPelotasBrazil; University of AdelaideAdelaideAustralia; Universidad Politécnica de MadridMadridSpain; Instituto Mexicano del Seguro SocialMexico CityMexico; St Anne's University HospitalBrnoCzech Republic; National Institute of Public HealthCuernavacaMexico; Centro de Estudios en Diabetes A.C.Mexico CityMexico; Universidad Autónoma de Santo DomingoSanto DomingoDominican Republic; University of LilleLilleFrance; Ministry of HealthLisbonPortugal; Norwegian Institute of Public HealthOsloNorway; Institute for Clinical and Experimental MedicinePragueCzech Republic; Children's Memorial Health InstituteWarsawPoland; Aristotle University of ThessalonikiThessalonikiGreece; Dalhousie UniversityHalifaxCanada; Jagiellonian University Medical CollegeKrakówPoland; Norwegian Institute of Public HealthOsloNorway; University of Southern DenmarkOdenseDenmark; University of CataniaCataniaItaly; University of TurinTurinItaly; National Center of Cardiovascular DiseasesBeijingChina; University of FerraraFerraraItaly; Universidad Autónoma de Madrid CIBERESPMadridSpain; Authority Sanitaria San MarinoSan MarinoSan Marino; Icelandic Heart AssociationKopavogurIceland; University of IcelandReykjavikIceland; Universidad IcesiCaliColombia; Geneva University HospitalsGenevaSwitzerland; State University of Montes ClarosMontes ClarosBrazil; King's College LondonLondonUnited Kingdom; Icelandic Heart AssociationKopavogurIceland; International Agency for Research on CancerLyonFrance; Capital Medical UniversityBeijingChina; Capital Medical University Beijing Tongren HospitalBeijingChina; Healis-Sekhsaria Institute for Public HealthNavi MumbaiIndia; Eternal Heart Care Centre and Research InstituteJaipurIndia; University of IbadanIbadanNigeria; Children's Memorial Health InstituteWarsawPoland; Spanish Agency for Food Safety and NutritionMadridSpain; Institute for Clinical Effectiveness and Health PolicyBuenos AiresArgentina; University of ZurichZurichSwitzerland; National Health Insurance ServiceWonjuRepublic of Korea; Prevention of Metabolic Disorders Research CenterTehranIslamic Republic of Iran; Research and Education Institute of Child HealthNicosiaCyprus; Non-Communicable Diseases Research CenterTehranIslamic Republic of Iran; Rafsanjan University of Medical SciencesRafsanjanIslamic Republic of Iran; Danish Cancer Society Research CenterCopenhagenDenmark; The University of the West IndiesCave HillBarbados; Kermanshah University of Medical SciencesKermanshahIslamic Republic of Iran; University of GothenburgGothenburgSweden; BRAC James P Grant School of Public HealthDhakaBangladesh; University of Eastern FinlandKuopioFinland; Beijing Institute of OphthalmologyBeijingChina; ICMR - National Institute of NutritionHyderabadIndia; Zahedan University of Medical SciencesZahedanIslamic Republic of Iran; International Hellenic UniversityThessalonikiGreece; Kyushu UniversityFukuokaJapan; University of BergenBergenNorway; Tulane UniversityNew OrleansUnited States; National Research Institute for Health and Family PlanningBeijingChina; Chinese Center for Disease Control and PreventionBeijingChina; University of PécsPécsHungary; University College DublinDublinIreland; Danish Health AuthorityCopenhagenDenmark; Joep Lange InstituteAmsterdamNetherlands; Federal University of PernambucoRecifeBrazil; Institute of Public Health of the University of PortoPortoPortugal; National Institute of Public HealthCuernavacaMexico; Oulu University HospitalOuluFinland; Universidad Autónoma de BucaramangaBucaramangaColombia; ETH ZurichZurichSwitzerland; Chronic Diseases Research CenterTehranIslamic Republic of Iran; University of SouthamptonSouthamptonUnited Kingdom; University of Hong KongHong KongChina; The Chinese University of Hong KongHong KongChina; University of Western AustraliaPerthAustralia; French National Research Institute for Sustainable DevelopmentMontpellierFrance; Fasa University of Medical SciencesFasaIslamic Republic of Iran; Gasol FoundationSpain; University Ramon LlullSant Boi de LlobregatSpain; Kingston Health Sciences CentreKingstonCanada; University of São PauloSão PauloBrazil; Fundación Oftalmológica de SantanderBucaramangaColombia; Federal University of PelotasPelotasBrazil; University Oran 1OranAlgeria; The University of the West IndiesCave HillBarbados; Independent Public Health SpecialistNay Pyi TawMyanmar; Ministry of Health and SportsNay Pyi TawMyanmar; Ministry of Health and SportsNay Pyi TawMyanmar; Peking UniversityBeijingChina; CIBER en Epidemiología y Salud PúblicaMurciaSpain; Imperial College LondonLondonUnited Kingdom; Luxembourg Institute of HealthLuxembourgLuxembourg; National Institute of Public HealthBucharestRomania; VU University Medical CenterAmsterdamNetherlands; Birzeit UniversityBirzeitState of Palestine; National Institute of NutritionHanoiViet Nam; International Agency for Research on CancerLyonFrance; American University of BeirutBeirutLebanon; Institute of Public HealthTiranaAlbania; IRCCS NeuromedPozzilliItaly; University of InsubriaVareseItaly; CIBER en Epidemiología y Salud PúblicaSan SebastianSpain; Cairo UniversityCairoEgypt; Ministry of HealthKuala LumpurMalaysia; Erasmus Medical Center RotterdamRotterdamNetherlands; Medical University VarnaVarnaBulgaria; Institute for Clinical Effectiveness and Health PolicyBuenos AiresArgentina; The University of TokyoTokyoJapan; The Hospital for Sick ChildrenTorontoCanada; Deakin UniversityGeelongAustralia; Tokyo Metropolitan Institute of GerontologyTokyoJapan; Hadassah University Medical CenterJerusalemIsrael; Jordan University of Science and TechnologyIrbidJordan; Duke-NUS Medical SchoolSingaporeSingapore; The University of the West IndiesKingstonJamaica; Kuwait Institute for Scientific ResearchSafatKuwait; University of AdelaideAdelaideAustralia; Norwegian University of Science and TechnologyTrondheimNorway; University of MelbourneMelbourneAustralia; Sports University of TiranaTiranaAlbania; Imperial College LondonLondonUnited Kingdom; University of OuluOuluFinland; Jagiellonian University Medical CollegeKrakówPoland; University Hospital Center ZagrebZagrebCroatia; University of Zagreb School of MedicineZagrebCroatia; Heart FoundationMelbourneAustralia; Nepal Health Research CouncilKathmanduNepal; Guangzhou 12th HospitalGuangzhouChina; Universidad Eugenio Maria de HostosSanto DomingoDominican Republic; University of Duisburg-EssenDuisburgGermany; Simon Fraser UniversityBurnabyCanada; International Agency for Research on CancerLyonFrance; Oulu University HospitalOuluFinland; Institute of Molecular and Clinical Ophthalmology BaselBaselSwitzerland; University of New South WalesSydneyAustralia; Bispebjerg and Frederiksberg HospitalCopenhagenDenmark; World Health Organization Country OfficeDelhiIndia; Guilan University of Medical SciencesRashtIslamic Republic of Iran; Institute of Public HealthBelgradeSerbia; University of OpoleOpolePoland; Finnish Institute for Health and WelfareHelsinkiFinland; University of LjubljanaLjubljanaSlovenia; Victor Babes University of Medicine and Pharmacy TimisoaraTimisoaraRomania; University of ZagrebZagrebCroatia; German Cancer Research CenterHeidelbergGermany; Gulu UniversityGuluUganda; University of CreteHeraklionGreece; Finnish Institute for Health and WelfareHelsinkiFinland; Al-Farabi Kazakh National UniversityAlmatyKazakhstan; The Gertner Institute for Epidemiology and Health Policy ResearchRamat GanIsrael; French National Research Institute for Sustainable DevelopmentMontpellierFrance; Bispebjerg and Frederiksberg HospitalCopenhagenDenmark; Sri Venkateswara UniversityTirupatiIndia; Sree Chitra Tirunal Institute for Medical Sciences and TechnologyTrivandrumIndia; Hellenic Medical Association for ObesityAthensGreece; National and Kapodistrian University of AthensAthensGreece; Bispebjerg and Frederiksberg HospitalCopenhagenDenmark; Maharajgunj Medical CampusKathmanduNepal; Aarhus UniversityAarhusDenmark; Johns Hopkins Bloomberg School of Public HealthBaltimoreUnited States; Pennington Biomedical Research CenterBaton RougeUnited States; University of Eastern FinlandKuopioFinland; National Institute of EpidemiologyChennaiIndia; Erasmus Medical Center RotterdamRotterdamNetherlands; Ufa Eye Research InstituteUfaRussian Federation; University of MünsterMünsterGermany; Israel Center for Disease ControlRamat GanIsrael; Oulu University HospitalOuluFinland; Research Institute for Primordial Prevention of Non-communicable DiseaseIsfahanIslamic Republic of Iran; University College DublinDublinIreland; Amsterdam UMC Public Health Research InstituteAmsterdamNetherlands; South African Medical Research CouncilCape TownSouth Africa; Mashhad University of Medical SciencesMashhadIslamic Republic of Iran; Kyrgyz State Medical AcademyBishkekKyrgyzstan; Research Institute of Child NutritionDortmundGermany; University of OxfordOxfordUnited Kingdom; Jordan University of Science and TechnologyIrbidJordan; Shahid Beheshti University of Medical SciencesTehranIslamic Republic of Iran; University of CambridgeCambridgeUnited Kingdom; Shahid Beheshti University of Medical SciencesTehranIslamic Republic of Iran; Mazandaran University of Medical SciencesSariIslamic Republic of Iran; Hypertension Research CenterIsfahanIslamic Republic of Iran; FrieslandCampinaAmersfoortNetherlands; Medical University of InnsbruckInnsbruckAustria; Medical University of InnsbruckInnsbruckAustria; VASCageInnsbruckAustria; Muhimbili University of Health and Allied SciencesDar es SalaamUnited Republic of Tanzania; National Health Insurance ServiceWonjuRepublic of Korea; Yonsei University College of MedicineSeoulRepublic of Korea; National Cancer CenterGoyang-siRepublic of Korea; University of GothenburgGothenburgSweden; Sahlgrenska University HospitalGothenburgSweden; University College South DenmarkHaderslevDenmark; Jagiellonian University Medical CollegeKrakówPoland; Statistics AustriaViennaAustria; Lithuanian University of Health SciencesKaunasLithuania; Medical University of InnsbruckInnsbruckAustria; B P Koirala Institute of Health SciencesDharanNepal; Norwegian School of Sport SciencesOsloNorway; Ghent UniversityGhentBelgium; University of ViennaViennaAustria; University of OuluOuluFinland; Oulu Deaconess Institute FoundationOuluFinland; Tartu University ClinicsTartuEstonia; Institute of Mother and ChildWarsawPoland; University Hospital Center ZagrebZagrebCroatia; Finnish Institute for Health and WelfareHelsinkiFinland; Kansai Medical UniversityHirakataJapan; Hungarian School Sport FederationBudapestHungary; Ministry of Health and Quality of LifePort LouisMauritius; PASs Hirszfeld Institute of Immunology and Experimental TherapyWroclawPoland; National Institute of Public HealthPragueCzech Republic; University Hospital UlmUlmGermany; University of ZurichZurichSwitzerland; University of Southern DenmarkOdenseDenmark; Norwegian University of Science and TechnologyTrondheimNorway; University of GroningenGroningenNetherlands; North-West UniversityPotchefstroomSouth Africa; National Institute of Public HealthPragueCzech Republic; Lithuanian University of Health SciencesKaunasLithuania; University of JyväskyläJyväskyläFinland; Institute of Public HealthPodgoricaMontenegro; Children's Memorial Health InstituteWarsawPoland; Amrita Institute of Medical SciencesCochinIndia; Institute of EndocrinologyPragueCzech Republic; National Institute of CardiologyWarsawPoland; All India Institute of Medical SciencesNew DelhiIndia; Finnish Institute for Health and WelfareHelsinkiFinland; African Population and Health Research CenterNairobiKenya; Hanoi University of Public HealthHanoiViet Nam; Hassan First University of SettatSettatMorocco; Finnish Institute for Health and WelfareHelsinkiFinland; Ghent UniversityGhentBelgium; Ministry of HealthAlgiersAlgeria; University of Hong KongHong KongChina; Harokopio UniversityAthensGreece; French National Research Institute for Sustainable DevelopmentMontpellierFrance; Institute for Clinical and Experimental MedicinePragueCzech Republic; Sahlgrenska AcademyGothenburgSweden; Endocrinology and Metabolism Research CenterTehranIslamic Republic of Iran; University of Public HealthYangonMyanmar; Istituto Superiore di SanitàRomeItaly; Universidad Peruana Cayetano HerediaLimaPeru; Luxembourg Institute of HealthStrassenLuxembourg; National Institute of NutritionHanoiViet Nam; International Food Policy Research InstituteDakarSenegal; National Institute of NutritionHanoiViet Nam; National University of SingaporeSingaporeSingapore; National Cancer CenterGoyang-siRepublic of Korea; Hong Kong Polytechnic UniversityHong KongChina; University of Duisburg-EssenDuisburgGermany; Tampere University HospitalTampereFinland; Tampere UniversityTampereFinland; University of DoualaDoualaCameroon; University of Cape TownCape TownSouth Africa; Harvard TH Chan School of Public HealthBostonUnited States; National Institute for Health DevelopmentTallinnEstonia; West Virginia UniversityMorgantownUnited States; National University of SingaporeSingaporeSingapore; Oswaldo Cruz Foundation Rene Rachou Research InstituteBelo HorizonteBrazil; National Taiwan UniversityTaipeiTaiwan; University of Chinese Academy of SciencesShanghaiChina; Uppsala UniversityUppsalaSweden; Uppsala UniversityUppsalaSweden; Bispebjerg and Frederiksberg HospitalCopenhagenDenmark; University of GothenburgGothenburgSweden; Children's Memorial Health InstituteWarsawPoland; Capital Medical University Beijing Tongren HospitalBeijingChina; Taipei Medical UniversityTaipeiTaiwan; National Institute for Health DevelopmentTallinnEstonia; Hanoi University of Public HealthHanoiViet Nam; University of PortoPortoPortugal; Sports Medical Center of MinhoBragaPortugal; Universidad Autónoma de Madrid CIBERESPMadridSpain; Universidad San Martín de PorresLimaPeru; University of São PauloSão PauloBrazil; Consejería de Sanidad Junta de Castilla y LeónValladolidSpain; Universidade Federal de Ciências da Saúde de Porto AlegrePorto AlegreBrazil; Lithuanian University of Health SciencesKaunasLithuania; Finnish Institute for Health and WelfareHelsinkiFinland; Norrbotten County CouncilLuleåSweden; University of PortoPortoPortugal; Ilembula Lutheran HospitalIlembulaUnited Republic of Tanzania; Charles UniversityPragueCzech Republic; National Institute of Public HealthPragueCzech Republic; University of RzeszówRzeszówPoland; Peking UniversityBeijingChina; Peking UniversityBeijingChina; National Research Institute for Health and Family PlanningBeijingChina; Universidade Federal de Ouro PretoOuro PretoBrazil; University of CoimbraCoimbraPortugal; Coimbra University Hospital CenterCoimbraPortugal; University of OsloOsloNorway; Institute of Neuroscience of the National Research CouncilPaduaItaly; Baker Heart and Diabetes InstituteMelbourneAustralia; Mediterranea CardiocentroNaplesItaly; Agricultural University of AthensAthensGreece; Christian Medical CollegeVelloreIndia; French National Research Institute for Sustainable DevelopmentMontpellierFrance; University of ZagrebZagrebCroatia; Hospital Israelita Albert EinsteinSão PauloBrazil; Finnish Institute for Health and WelfareHelsinkiFinland; Tehran University of Medical SciencesTehranIslamic Republic of Iran; Tehran University of Medical SciencesTehranIslamic Republic of Iran; Duke-NUS Medical SchoolSingaporeSingapore; ICMR - National Institute of NutritionHyderabadIndia; SB RAS Federal Research Center Institute of Cytology and GeneticsNovosibirskRussian Federation; Food and Nutrition Research InstituteTaguigPhilippines; Harokopio UniversityAthensGreece; University of OtagoDunedinNew Zealand; Guilan University of Medical SciencesRashtIslamic Republic of Iran; University of PaduaPaduaItaly; Pontificia Universidad Católica de ChileSantiagoChile; Hellenic Mediterranean UniversitySiteiaGreece; Loughborough UniversityLoughboroughUnited Kingdom; Ministry of HealthNicosiaCyprus; Lausanne University HospitalLausanneSwitzerland; Escola Nacional de Saúde Pública Sergio AroucaRio de JaneiroBrazil; CIBERCVMadridSpain; Institut Hospital del Mar d'Investigacions MèdiquesBarcelonaSpain; French National Research Institute for Sustainable DevelopmentMontpellierFrance; Mary Immaculate CollegeLimerickIreland; Emory UniversityAtlantaUnited States; Hungarian Society of Sports MedicineBudapestHungary; Paracelsus Medical UniversitySalzburgAustria; University of CataniaCataniaItaly; Universidade Estadual do Centro-OesteGuarapuavaBrazil; Sher-i-Kashmir Institute of Medical SciencesSrinagarIndia; UiT The Arctic University of NorwayTromsøNorway; ICMR - National Centre for Disease Informatics and ResearchBengaluruIndia; University of São PauloSão PauloBrazil; Cape Peninsula University of TechnologyCape TownSouth Africa; Harokopio UniversityAthensGreece; University of RzeszówRzeszówPoland; University of Yaoundé 1YaoundéCameroon; The University of the West IndiesKingstonJamaica; Brown UniversityProvidenceUnited States; London School of Hygiene & Tropical MedicineLondonUnited Kingdom; University of EdinburghEdinburghUnited Kingdom; University of OtagoDunedinNew Zealand; Statistics CanadaOttawaCanada; University College DublinDublinIreland; University Oran 1OranAlgeria; Lithuanian University of Health SciencesKaunasLithuania; Non-Communicable Diseases Research CenterTehranIslamic Republic of Iran; University of GothenburgGothenburgSweden; Shahid Sadoughi University of Medical SciencesTehranIslamic Republic of Iran; Institut National de la Santé et de la Recherche MédicaleLilleFrance; Norwegian Institute of Public HealthOsloNorway; Helmholtz Zentrum MünchenMunichGermany; Federal University of PelotasPelotasBrazil; ICMR - National Institute of Medical StatisticsNew DelhiIndia; Robert Koch InstituteBerlinGermany; Ministero della Salute DG Prevenzione SanitariaRomeItaly; Al-Farabi Kazakh National UniversityAlmatyKazakhstan; ICMR - National Institute of NutritionHyderabadIndia; University of TartuTartuEstonia; University of OsloOsloNorway; Capital Institute of PediatricsBeijingChina; University of CopenhagenCopenhagenDenmark; Ghent UniversityGhentBelgium; University of TartuTartuEstonia; Jagiellonian University Medical CollegeKrakówPoland; University of OtagoDunedinNew Zealand; Universidade de LisboaLisbonPortugal; Women’s Social and Health Studies FoundationTrivandrumIndia; Pontificia Universidad Católica de ChileSantiagoChile; Shahid Sadoughi University of Medical SciencesTehranIslamic Republic of Iran; Democritus UniversityAlexandroupolisGreece; Kyrgyz State Medical AcademyBishkekKyrgyzstan; University of ZagrebZagrebCroatia; University of CataniaCataniaItaly; Grigore T Popa University of Medicine and PharmacyIasiRomania; Università degli Studi di FirenzeFlorenceItaly; Non-Communicable Diseases Research CenterTehranIslamic Republic of Iran; Non-Communicable Diseases Research CenterTehranIslamic Republic of Iran; Ain Shams UniversityCairoEgypt; African Population and Health Research CenterNairobiKenya; Tehran University of Medical SciencesTehranIslamic Republic of Iran; Tehran University of Medical SciencesTehranIslamic Republic of Iran; Isfahan Cardiovascular Research CenterIsfahanIslamic Republic of Iran; Mashhad University of Medical SciencesMashhadIslamic Republic of Iran; Madras Diabetes Research FoundationChennaiIndia; Universidad Peruana Cayetano HerediaLimaPeru; Ministry of HealthKuala LumpurMalaysia; Urmia University of Medical SciencesUrmiaIslamic Republic of Iran; Non-Communicable Diseases Research CenterTehranIslamic Republic of Iran; University of StrasbourgStrasbourgFrance; Strasbourg University HospitalStrasbourgFrance; University of CopenhagenCopenhagenDenmark; Bispebjerg and Frederiksberg HospitalCopenhagenDenmark; University of Southern DenmarkOdenseDenmark; University of PécsPécsHungary; Shahid Beheshti University of Medical SciencesTehranIslamic Republic of Iran; Mulago HospitalKampalaUganda; University of San Carlos of GuatemalaGuatemala CityGuatemala; National Institute of Public HealthCuernavacaMexico; University of LimpopoSovengaSouth Africa; Seoul National UniversitySeoulRepublic of Korea; Mazandaran University of Medical SciencesSariIslamic Republic of Iran; Universidade Federal do Rio Grande do SulPorto AlegreBrazil; University of Medical Sciences of CienfuegosCienfuegosCuba; University of ZaragozaZaragozaSpain; CIBEROBNZaragozaSpain; Royal College of Surgeons in IrelandDublinIreland; McGill UniversityMontrealCanada; University of CopenhagenCopenhagenDenmark; La Trobe UniversityMelbourneAustralia; International Institute of Molecular and Cell BiologyWarsawPoland; Ain Shams UniversityCairoEgypt; University of CoimbraCoimbraPortugal; University of PortoPortoPortugal; Ahvaz Jundishapur University of Medical SciencesAhvazIslamic Republic of Iran; Instituto Conmemorativo Gorgas de Estudios de la SaludPanama CityPanama; University of PernambucoRecifeBrazil; BRAC James P Grant School of Public HealthDhakaBangladesh; World Health Organization Country OfficeLilongweMalawi; Department of Public HealthNay Pyi TawMyanmar; University of CoimbraCoimbraPortugal; Institute of Public HealthPodgoricaMontenegro; University of BresciaBresciaItaly; Ministry of Health and Social ProtectionDushanbeTajikistan; Helmholtz Zentrum MünchenMunichGermany; International Agency for Research on CancerLyonFrance; University of Eastern FinlandKuopioFinland; University of LimerickLimerickIreland; Universiti Sains MalaysiaKelantanMalaysia; Croatian Institute of Public HealthZagrebCroatia; University of ZagrebZagrebCroatia; University of ZagrebZagrebCroatia; Universiti Kebangsaan MalaysiaKuala LumpurMalaysia; Bushehr University of Medical SciencesBushehrIslamic Republic of Iran; Non-Communicable Diseases Research CenterTehranIslamic Republic of Iran; Ulm UniversityUlmGermany; Department of StatisticsKuala LumpurMalaysia; Kermanshah University of Medical SciencesKermanshahIslamic Republic of Iran; Kobe UniversityKobeJapan; Banska Bystrica Regional Authority of Public HealthBanska BystricaSlovakia; National University of SingaporeSingaporeSingapore; Suraj Eye InstituteNagpurIndia; UNICEFYaoundéCameroon; Healis-Sekhsaria Institute for Public HealthNavi MumbaiIndia; Istituto Superiore di SanitàRomeItaly; University Medicine GreifswaldGreifswaldGermany; West Virginia UniversityMorgantownUnited States; Hormozgan University of Medical SciencesBandar AbbasIslamic Republic of Iran; University of New South WalesSydneyAustralia; National Institute for Health DevelopmentTallinnEstonia; National Institute for Health DevelopmentTallinnEstonia; Jagiellonian University Medical CollegeKrakówPoland; Karolinska InstitutetStockholmSweden; Pontificia Universidad Católica de ChileSantiagoChile; National Institute of Hygiene and EpidemiologyHanoiViet Nam; University of Medicine and Pharmacy at Ho Chi Minh CityHo Chi Minh CityViet Nam; Hanoi Medical UniversityHanoiViet Nam; LifeDoc HealthMemphisUnited States; SB RAS Federal Research Center Institute of Cytology and GeneticsNovosibirskRussian Federation; Shanghai Jiao-Tong University School of MedicineShanghaiChina; Kyushu UniversityFukuokaJapan; HeartfileIslamabadPakistan; Institute of Neuroscience of the National Research CouncilPaduaItaly; Universidad de la RepúblicaMontevideoUruguay; University of CoimbraCoimbraPortugal; Imperial College LondonLondonUnited Kingdom; Umeå UniversityUmeåSweden; Copenhagen University HospitalCopenhagenDenmark; University of CopenhagenCopenhagenDenmark; University of PalermoPalermoItaly; PASs Hirszfeld Institute of Immunology and Experimental TherapyWroclawPoland; Eastern Mediterranean Public Health NetworkAmmanJordan; Karadeniz Technical UniversityTrabzonTurkey; National Institute for Health DevelopmentTallinnEstonia; University of ManchesterManchesterUnited Kingdom; Queen's University of BelfastBelfastUnited Kingdom; State University of Medicine and PharmacyChisinauMoldova; Harvard TH Chan School of Public HealthBostonUnited States; Universidad de CuencaCuencaEcuador; Tachikawa General HospitalNagaokaJapan; Korea Centers for Disease Control and PreventionCheongju-siRepublic of Korea; Kindai UniversityOsaka-SayamaJapan; University of GothenburgGothenburgSweden; Sahlgrenska University HospitalGothenburgSweden; Japan Wildlife Research CenterTokyoJapan; Icelandic Heart AssociationKopavogurIceland; Universidade do Vale do Rio dos SinosSão LeopoldoBrazil; Federal University of PelotasPelotasBrazil; Ministry of HealthKuala LumpurMalaysia; Istanbul UniversityIstanbulTurkey; Ministry of HealthBandar Seri BegawanBrunei Darussalam; Universidade Federal do ParanáCuritibaBrazil; Pan American Health OrganizationWashington DCUnited States; University of MadeiraFunchalPortugal; University of Puerto RicoSan JuanPuerto Rico; Universidad Peruana Cayetano HerediaLimaPeru; Center for Clinical Research and PreventionGlostrupDenmark; MRC Lifecourse Epidemiology UnitSouthamptonUnited Kingdom; University of Novi SadNovi SadSerbia; Tehran University of Medical SciencesTehranIslamic Republic of Iran; Fundación Oftalmológica de SantanderBucaramangaColombia; Aarhus UniversityAarhusDenmark; Kwame Nkrumah University of Science and TechnologyKumasiGhana; UniSantéLausanneSwitzerland; University of CoimbraCoimbraPortugal; International Hellenic UniversityThessalonikiGreece; University of LatviaRigaLatvia; Federal University of Santa CatarinaFlorianópolisBrazil; Jagiellonian University Medical CollegeKrakówPoland; Institute for Cancer Research, Prevention and Clinical NetworkFlorenceItaly; University of Wisconsin-MadisonMadisonUnited States; Istituto Superiore di SanitàRomeItaly; Academia SinicaTaipeiTaiwan; Ruprecht-Karls-University of HeidelbergHeidelbergGermany; ICMR - National Institute of Medical StatisticsNew DelhiIndia; IRCCS Ente Ospedaliero Specializzato in Gastroenterologia S. de BellisBariItaly; Zayed UniversityAbu DhabiUnited Arab Emirates; Catholic University of DaeguDaeguRepublic of Korea; Korea Centers for Disease Control and PreventionCheongju-siRepublic of Korea; University of OtagoDunedinNew Zealand; Tehran University of Medical SciencesTehranIslamic Republic of Iran; University of Medicine, Pharmacy, Science and Technology of Târgu MuresTârgu MuresRomania; UMR CNRS-MNHN 7206 Eco-anthropologieParisFrance; Jivandeep HospitalAnandIndia; University Hospital Center ZagrebZagrebCroatia; Healis-Sekhsaria Institute for Public HealthNavi MumbaiIndia; South African Medical Research CouncilDurbanSouth Africa; Peking UniversityBeijingChina; Oswaldo Cruz Foundation Rene Rachou Research InstituteBelo HorizonteBrazil; Finnish Institute for Health and WelfareHelsinkiFinland; University of São PauloSão PauloBrazil; National Dental Care Centre SingaporeSingaporeSingapore; Universidad de MálagaMalagaSpain; University of Puerto RicoSan JuanPuerto Rico; Endocrinology Research CentreMoscowRussian Federation; Helmholtz Zentrum MünchenMunichGermany; University Medicine GreifswaldGreifswaldGermany; Lithuanian University of Health SciencesKaunasLithuania; Lithuanian University of Health SciencesKaunasLithuania; University Hospital of VareseVareseItaly; Ministry of Health and Medical EducationTehranIslamic Republic of Iran; Vietnam National Heart InstituteHanoiViet Nam; Clínica de Medicina Avanzada Dr. Abel GonzálezSanto DomingoDominican Republic; Istituto Superiore di SanitàRomeItaly; Leibniz Institute for Prevention Research and Epidemiology - BIPSBremenGermany; University College LondonLondonUnited Kingdom; University of SarajevoSarajevoBosnia and Herzegovina; Cardiovascular Prevention Centre UdineUdineItaly; Pisa University HospitalPisaItaly; Ministry of Health and Medical ServicesHoniaraSolomon Islands; National Institute of CardiologyWarsawPoland; University of PortoPortoPortugal; Public Health Agency of CataloniaBarcelonaSpain; Universiti Kebangsaan MalaysiaKuala LumpurMalaysia; Leibniz Institute for Prevention Research and Epidemiology - BIPSBremenGermany; University of Medicine, Pharmacy, Science and Technology of Târgu MuresTârgu MuresRomania; University of MontenegroNiksicMontenegro; Institut Hospital del Mar d'Investigacions MèdiquesBarcelonaSpain; Agency for Preventive and Social MedicineBregenzAustria; Nepal Health Research CouncilKathmanduNepal; International Hellenic UniversityThessalonikiGreece; Tehran University of Medical SciencesTehranIslamic Republic of Iran; Ardabil University of Medical SciencesArdabilIslamic Republic of Iran; Tehran University of Medical SciencesTehranIslamic Republic of Iran; Tehran University of Medical SciencesTehranIslamic Republic of Iran; Madras Diabetes Research FoundationChennaiIndia; London School of Hygiene & Tropical MedicineLondonUnited Kingdom; University of EdinburghEdinburghUnited Kingdom; University College LondonLondonUnited Kingdom; Lausanne University HospitalLausanneSwitzerland; Centre for Disease Prevention and ControlRigaLatvia; University of OuluOuluFinland; Oulu Deaconess Institute FoundationOuluFinland; Victor Babes University of Medicine and Pharmacy TimisoaraTimisoaraRomania; Tartu University ClinicsTartuEstonia; Al-Quds UniversityEast JerusalemState of Palestine; Alborz University of Medical SciencesKarajIslamic Republic of Iran; Ministry of HealthHanoiViet Nam; University of Novi SadNovi SadSerbia; Lithuanian University of Health SciencesKaunasLithuania; Shiraz University of Medical SciencesShirazIslamic Republic of Iran; Pure EarthDhakaBangladesh; Institute of Epidemiology Disease Control and ResearchDhakaBangladesh; University of TurkuTurkuFinland; Amrita Institute of Medical SciencesCochinIndia; Ufa Eye Research InstituteUfaRussian Federation; Ministry of Health and Social ProtectionDushanbeTajikistan; World Health Organization Regional Office for EuropeMoscowRussian Federation; National Institute of EpidemiologyChennaiIndia; India Diabetes Research FoundationChennaiIndia; University of AucklandAucklandNew Zealand; University of Porto Medical SchoolPortoPortugal; Institut Universitari d'Investigació en Atenció Primària Jordi GolGironaSpain; Universiti Putra MalaysiaSerdangMalaysia; University of MalayaKuala LumpurMalaysia; Sotiria HospitalAthensGreece; Instituto Mexicano del Seguro SocialMexico CityMexico; University of Southern DenmarkOdenseDenmark; Federal University of Santa CatarinaFlorianópolisBrazil; University of ValenciaValenciaSpain; University of the PhilippinesManilaPhilippines; Slovak Academy of SciencesBratislavaSlovakia; Universidad San Martín de PorresLimaPeru; Shiraz University of Medical SciencesShirazIslamic Republic of Iran; Nutrition Research FoundationBarcelonaSpain; Minas Gerais State Secretariat for HealthBelo HorizonteBrazil; Imperial College LondonLondonUnited Kingdom; University Medicine GreifswaldGreifswaldGermany; CS S. Agustín IbsalutPalmaSpain; University of FerraraFerraraItaly; Amsterdam Institute for Global Health and DevelopmentAmsterdamNetherlands; National Institute of Health Doutor Ricardo JorgeLisbonPortugal; Universidade Nove de JulhoSão PauloBrazil; National Institute of Public HealthCuernavacaMexico; Public Health Agency of CanadaOttawaCanada; Council for Agricultural Research and EconomicsRomeItaly; University of CoimbraCoimbraPortugal; Universidad Autónoma de Madrid CIBERESPMadridSpain; Canarian Health ServiceTenerifeSpain; Universidad Industrial de SantanderBucaramangaColombia; University of Duisburg-EssenDuisburgGermany; National Institute of Public HealthCuernavacaMexico; Mahidol UniversityNakhon PathomThailand; CIBEROBNMadridSpain; Associazione Calabrese di EpatologiaReggio CalabriaItaly; University of MinhoBragaPortugal; University of GothenburgGothenburgSweden; Sahlgrenska University HospitalGothenburgSweden; Fiji National UniversitySuvaFiji; Statistics CanadaOttawaCanada; Institute for Clinical Effectiveness and Health PolicyBuenos AiresArgentina; University of ZurichZurichSwitzerland; Toulouse University School of MedicineToulouseFrance; Instituto Mexicano del Seguro SocialMexico CityMexico; Luxembourg Institute of HealthStrassenLuxembourg; Spanish Nutrition FoundationMadridSpain; Ufa Eye Research InstituteUfaRussian Federation; Public Health Agency of SwedenStockholmSweden; Institute of Food Sciences of the National Research CouncilAvellinoItaly; University of ViennaViennaAustria; Medical University of GdanskGdanskPoland; Singapore Eye Research InstituteSingaporeSingapore; Authority Sanitaria San MarinoSan MarinoSan Marino; Sitaram Bhartia Institute of Science and ResearchNew DelhiIndia; Tehran University of Medical SciencesTehranIslamic Republic of Iran; Shiraz University of Medical SciencesShirazIslamic Republic of Iran; Tabriz University of Medical SciencesTabrizIslamic Republic of Iran; Ahvaz Jundishapur University of Medical SciencesAhvazIslamic Republic of Iran; French Public Health AgencySt MauriceFrance; National Institute of Public HealthCuernavacaMexico; CIBER en Epidemiología y Salud PúblicaMurciaSpain; Finnish Institute for Health and WelfareHelsinkiFinland; University of HelsinkiHelsinkiFinland; University of BresciaBresciaItaly; Kindergarten of AvlonariEviaGreece; National Institute of HealthLimaPeru; Ministry of HealthJakartaIndonesia; Catalan Department of HealthBarcelonaSpain; Biodonostia Health Research InstituteSan SebastianSpain; Universidade de LisboaLisbonPortugal; Federal University of PelotasPelotasBrazil; Coimbra University Hospital CenterCoimbraPortugal; University of PortoPortoPortugal; Instituto de Saúde AmbientalLisbonPortugal; University of PortoPortoPortugal; Spanish Agency for Food Safety and NutritionMadridSpain; South Karelia Social and Health Care DistrictLappeenrantaFinland; Universidade de LisboaLisbonPortugal; Isfahan Cardiovascular Research CenterIsfahanIslamic Republic of Iran; McMaster UniversityHamiltonCanada; German Cancer Research CenterHeidelbergGermany; Research and Education Institute of Child HealthNicosiaCyprus; French National Research Institute for Sustainable DevelopmentMontpellierFrance; National Cancer CenterTokyoJapan; Universidade Federal do Rio Grande do SulPorto AlegreBrazil; University of São Paulo Clinics HospitalSão PauloBrazil; Universidade Federal do Rio Grande do SulPorto AlegreBrazil; Robert Koch InstituteBerlinGermany; Hospital Italiano de Buenos AiresBuenos AiresArgentina; Robert Koch InstituteBerlinGermany; University Medicine GreifswaldGreifswaldGermany; University Medicine GreifswaldGreifswaldGermany; RigshospitaletCopenhagenDenmark; Copenhagen University HospitalCopenhagenDenmark; German Cancer Research CenterHeidelbergGermany; University of Duisburg-EssenDuisburgGermany; University of Southern DenmarkOdenseDenmark; CIBER en Epidemiología y Salud PúblicaBarcelonaSpain; Academic Medical Center of University of AmsterdamAmsterdamNetherlands; University of New South WalesSydneyAustralia; The George Institute for Global HealthSydneyAustralia; Ministry of Health and SportsNay Pyi TawMyanmar; Ministry of HealthKuala LumpurMalaysia; University of LjubljanaLjubljanaSlovenia; Center for Oral Health Services and Research Mid-NorwayTrondheimNorway; Lagos State University College of MedicineLagosNigeria; Tehran University of Medical SciencesTehranIslamic Republic of Iran; Ministry of Public HealthAsunciónParaguay; University of Las Palmas de Gran CanariaLas Palmas de Gran CanariaSpain; Statistics CanadaOttawaCanada; Comenius UniversityBratislavaSlovakia; National Research Centre for Preventive MedicineMoscowRussian Federation; National Institute of Public HealthCuernavacaMexico; Neyshabur University of Medical SciencesNeyshaburIslamic Republic of Iran; Madras Diabetes Research FoundationChennaiIndia; Tehran University of Medical SciencesTehranIslamic Republic of Iran; B P Koirala Institute of Health SciencesDharanNepal; Baker Heart and Diabetes InstituteMelbourneAustralia; Tehran University of Medical SciencesTehranIslamic Republic of Iran; Ahvaz Jundishapur University of Medical SciencesAhvazIslamic Republic of Iran; National Center for Disease Control and Public HealthTbilisiGeorgia; Qatar UniversityDohaQatar; King's College LondonLondonUnited Kingdom; Nippon Medical SchoolTokyoJapan; Sungkyunkwan UniversitySeoulRepublic of Korea; Shahrekord University of Medical SciencesShahrekordIslamic Republic of Iran; Finnish Institute of Occupational HealthHelsinkiFinland; Public Health Promotion and Development OrganizationKathmanduNepal; Ministry of HealthBandar Seri BegawanBrunei Darussalam; Institute of Food Sciences of the National Research CouncilAvellinoItaly; Singapore Eye Research InstituteSingaporeSingapore; American University of BeirutBeirutLebanon; Federal University of MaranhãoSão LuísBrazil; Federal University of Santa CatarinaFlorianópolisBrazil; India Diabetes Research FoundationChennaiIndia; St Vincent's HospitalSydneyAustralia; University of New South WalesSydneyAustralia; University of GothenburgGothenburgSweden; Karolinska InstitutetStockholmSweden; Nes MunicipalityAarnesNorway; Medical University of LodzLodzPoland; International Institute of Molecular and Cell BiologyWarsawPoland; London School of Hygiene & Tropical MedicineLondonUnited Kingdom; University of Hong KongHong KongChina; University of PernambucoRecifeBrazil; University of RzeszówRzeszówPoland; University of Yaoundé 1YaoundéCameroon; University of Southern DenmarkOdenseDenmark; Umeå UniversityUmeåSweden; Health Polytechnic Jakarta II InstituteJakartaIndonesia; Diponegoro UniversitySemarangIndonesia; University College LondonLondonUnited Kingdom; University of BariBariItaly; Tabriz University of Medical SciencesTabrizIslamic Republic of Iran; Lund UniversityLundSweden; Peking UniversityBeijingChina; University of CopenhagenCopenhagenDenmark; Norwegian University of Science and TechnologyTrondheimNorway; Institut Régional de Santé PubliqueOuidahBenin; Universidad IcesiCaliColombia; University of BordeauxBordeauxFrance; University of Zagreb School of MedicineZagrebCroatia; Oslo Metropolitan UniversityOsloNorway; Universidade Federal do Rio Grande do SulPorto AlegreBrazil; Istituto Superiore di SanitàRomeItaly; Institute of Public HealthSkopjeNorth Macedonia; Ss. Cyril and Methodius UniversitySkopjeNorth Macedonia; University of LeuvenLeuvenBelgium; Lamprecht und Stamm Sozialforschung und Beratung AGZurichSwitzerland; Institut National de la Santé et de la Recherche MédicaleNancyFrance; University of ZurichZurichSwitzerland; Heart FoundationMelbourneAustralia; Norwegian School of Sport SciencesOsloNorway; Bonn UniversityBonnGermany; Emory UniversityAtlantaUnited States; Sotiria HospitalSotiriaGreece; Hadassah University Medical CenterJerusalemIsrael; Croatian Institute of Public HealthZagrebCroatia; Helmholtz Zentrum MünchenMunichGermany; Helmholtz Zentrum MünchenMunichGermany; Lund UniversityLundSweden; National Institute of Public Health - National Institute of HygieneWarsawPoland; Kalina Malina KindergartenPazardjikBulgaria; Swansea UniversitySwanseaUnited Kingdom; University of AmsterdamAmsterdamNetherlands; Federal University of São PauloSão PauloBrazil; National Center for Disease Control and Public HealthTbilisiGeorgia; National Institute of Hygiene, Epidemiology and MicrobiologyHavanaCuba; The Jikei University School of MedicineTokyoJapan; Fu Jen Catholic UniversityTaipeiTaiwan; Uppsala UniversityUppsalaSweden; The Chinese University of Hong KongHong KongChina; Barcelona Institute for Global Health CIBERESPBarcelonaSpain; Mahidol UniversityNakhon PathomThailand; University of AucklandAucklandNew Zealand; University of the PhilippinesManilaPhilippines; University of SouthamptonSouthamptonUnited Kingdom; National Statistical OfficePraiaCabo Verde; Johns Hopkins Bloomberg School of Public HealthBaltimoreUnited States; National Institute of Public Health – National Institute of HygieneWarsawPoland; National University of SingaporeSingaporeSingapore; University of TartuTartuEstonia; Lithuanian University of Health SciencesKaunasLithuania; Deakin UniversitySydneyAustralia; Peking UniversityBeijingChina; Scientific Research Institute of Maternal and Child HealthAshgabatTurkmenistan; University of LincolnLincolnUnited Kingdom; Peking UniversityBeijingChina; Ministry of HealthAmmanJordan; Norwegian School of Sport SciencesOsloNorway; National Institute of HealthLimaPeru; Institute of EndocrinologyPragueCzech Republic; University of AdelaideAdelaideAustralia; University College LondonLondonUnited Kingdom; UNICEFNiameyNiger; Universidade Estadual PaulistaPresidente PrudenteBrazil; University of BergenBergenNorway; Universidad Peruana Cayetano HerediaLimaPeru; Singapore Eye Research InstituteSingaporeSingapore; Central University of KeralaKasaragodIndia; Karolinska InstitutetHuddingeSweden; Aristotle University of ThessalonikiThessalonikiGreece; University of LeuvenLeuvenBelgium; Christian Medical CollegeVelloreIndia; Bispebjerg and Frederiksberg HospitalCopenhagenDenmark; Comenius UniversityBratislavaSlovakia; Amsterdam Public Health Research InstituteAmsterdamNetherlands; Danish Cancer Society Research CenterCopenhagenDenmark; Finnish Institute for Health and WelfareHelsinkiFinland; University of Southern DenmarkCopenhagenDenmark; Karadeniz Technical UniversityTrabzonTurkey; Jagiellonian University Medical CollegeKrakówPoland; Oslo Metropolitan UniversityOsloNorway; Health Service of MurciaMurciaSpain; Research and Education Institute of Child HealthNicosiaCyprus; Institut d'Investigacio Sanitaria Illes BalearsMenorcaSpain; CIBER en Epidemiología y Salud PúblicaAlicanteSpain; University of BolognaBolognaItaly; National and Kapodistrian University of AthensAthensGreece; French National Research Institute for Sustainable DevelopmentMontpellierFrance; Hanoi University of Public HealthHanoiViet Nam; Harvard TH Chan School of Public HealthBostonUnited States; Hellenic Health FoundationAthensGreece; University of Medicine and Pharmacy at Ho Chi Minh CityHo Chi Minh CityViet Nam; Government Medical CollegeBhavnagarIndia; Sefako Makgatho Health Science UniversityGa-RankuwaSouth Africa; International Hellenic UniversityThessalonikiGreece; National Cancer CenterTokyoJapan; Ufa Eye Research InstituteUfaRussian Federation; The University of the West IndiesKingstonJamaica; Addis Ababa UniversityAddis AbabaEthiopia; University of Eastern FinlandKuopioFinland; Finnish Institute for Health and WelfareHelsinkiFinland; Ministry of HealthWellingtonNew Zealand; Tel-Aviv UniversityTel-AvivIsrael; Hebrew University of JerusalemJerusalemIsrael; Karolinska InstitutetStockholmSweden; Hellenic Medical Association for ObesityAthensGreece; University of BordeauxBordeauxFrance; Karolinska InstitutetStockholmSweden; Universidad Centro-Occidental Lisandro AlvaradoBarquisimetoVenezuela; Meharry Medical CollegeNashvilleUnited States; Medical University of InnsbruckInnsbruckAustria; Dokuz Eylul UniversityIzmirTurkey; Republican Center for Health PromotionBishkekKyrgyzstan; University of Tampere Tays Eye CenterTampereFinland; Sabiha Gokcen IlkokuluAnkaraTurkey; Lithuanian University of Health SciencesKaunasLithuania; Pontificia Universidad Católica de ChileSantiagoChile; Polytechnic Institute of PortoPortoPortugal; Icahn School of Medicine at Mount SinaiNew York CityUnited States; National University of SingaporeSingaporeSingapore; SciensanoBrusselsBelgium; Utrecht UniversityUtrechtNetherlands; Ghent UniversityGhentBelgium; Hanoi University of Public HealthHanoiViet Nam; VU University Medical CenterAmsterdamNetherlands; University of AmsterdamAmsterdamNetherlands; Katholieke Universiteit LeuvenLeuvenBelgium; Cardiovascular Prevention Centre UdineUdineItaly; Copenhagen University HospitalCopenhagenDenmark; University of CopenhagenCopenhagenDenmark; Universidad CEU San PabloMadridSpain; National Institute of Hygiene, Epidemiology and MicrobiologyHavanaCuba; University of SouthamptonSouthamptonUnited Kingdom; Consejería de Sanidad Junta de Castilla y LeónValladolidSpain; National Institute for Health DevelopmentTallinnEstonia; Universidade Federal de Minas GeraisBelo HorizonteBrazil; Centre for Disease Prevention and ControlRigaLatvia; University of InsubriaVareseItaly; National Institute for Public Health and the EnvironmentBilthovenNetherlands; Federal University of PelotasPelotasBrazil; National Research CouncilPisaItaly; National Institute for Public Health and the EnvironmentBilthovenNetherlands; National Institute of Public HealthCuernavacaMexico; Imperial College LondonLondonUnited Kingdom; University Miguel HernandezAlicanteSpain; University of Eastern FinlandKuopioFinland; Vrije Universiteit AmsterdamAmsterdamNetherlands; Institut National de la Santé et de la Recherche MédicaleNancyFrance; Ministry of HealthVictoriaSeychelles; Sunflower Nursery SchoolCraiovaRomania; North Karelia Center for Public HealthJoensuuFinland; University of Zagreb School of MedicineZagrebCroatia; Lausanne University HospitalLausanneSwitzerland; University Medicine GreifswaldGreifswaldGermany; University of Eastern FinlandKuopioFinland; University of Eastern FinlandKuopioFinland; Barcelona Institute for Global Health CIBERESPBarcelonaSpain; University Medical CentersGroningenNetherlands; University of AmsterdamAmsterdamNetherlands; University of the WitwatersrandJohannesburgSouth Africa; University of StrasbourgStrasbourgFrance; Medical University of ViennaViennaAustria; Cork Institute of TechnologyCorkIreland; African Population and Health Research CenterNairobiKenya; Universiti Sains MalaysiaKelantanMalaysia; Institute for Medical ResearchKuala LumpurMalaysia; University of PernambucoRecifeBrazil; Public Health Agency of CanadaOttawaCanada; Capital Medical University Beijing Tongren HospitalBeijingChina; Xinjiang Medical UniversityUrumqiChina; Shanghai Educational Development Co. Ltd.ShanghaiChina; Capital Medical UniversityBeijingChina; Ministry of Health and WelfareTaipeiTaiwan; University College LondonLondonUnited Kingdom; University of CambridgeCambridgeUnited Kingdom; Federal Ministry of Social Affairs, Health, Care and Consumer ProtectionViennaAustria; University of Southern DenmarkOdenseDenmark; Ministry of HealthAucklandNew Zealand; Paracelsus Medical UniversitySalzburgAustria; Capital Medical UniversityBeijingChina; University of RzeszówRzeszówPoland; Örebro UniversityÖrebroSweden; St George's, University of LondonLondonUnited Kingdom; Medical University of ViennaViennaAustria; Universitas IndonesiaJakartaIndonesia; Medical University of SilesiaKatowicePoland; The University of the West IndiesKingstonJamaica; Medical University of InnsbruckInnsbruckAustria; Medical University of InnsbruckInnsbruckAustria; World Health Organization Regional Office for EuropeMoscowRussian Federation; UiT The Arctic University of NorwayTromsøNorway; National Institute of Public Health - National Institute of HygieneWarsawPoland; Caja Costarricense de Seguro SocialSan JoséCosta Rica; University College LondonLondonUnited Kingdom; Universiti Kebangsaan MalaysiaKuala LumpurMalaysia; Duke-NUS Medical SchoolSingaporeSingapore; The Chinese University of Hong KongHong KongChina; University of New South WalesSydneyAustralia; University of OxfordOxfordUnited Kingdom; University of ManchesterManchesterUnited Kingdom; Shandong University of Traditional Chinese MedicineJinanChina; Capital Medical UniversityBeijingChina; Kailuan General HospitalTangshanChina; Institute of Food and Nutrition Development of Ministry of Agriculture and Rural AffairsBeijingChina; Beijing Institute of OphthalmologyBeijingChina; Universiti Sains MalaysiaKelantanMalaysia; Mahidol UniversityNakhon PathomThailand; Children's Hospital of Fudan UniversityShanghaiChina; University of OxfordOxfordUnited Kingdom; Chinese Center for Disease Control and PreventionBeijingChina; Shanghai Educational Development Co. LtdShanghaiChina; Ministry of HealthAnkaraTurkey; Shahid Beheshti University of Medical SciencesTehranIslamic Republic of Iran; Kyoto UniversityKyotoJapan; University of Chinese Academy of SciencesShanghaiChina; University of CyprusNicosiaCyprus; Non-Communicable Diseases Research CenterTehranIslamic Republic of Iran; Niigata UniversityNiigataJapan; Capital Medical UniversityBeijingChina; Fu Jen Catholic UniversityTaipeiTaiwan; The University of the West IndiesKingstonJamaica; International Medical UniversityShah AlamMalaysia; Ministry of HealthKuala LumpurMalaysia; University of FerraraFerraraItaly; Hellenic Mediterranean UniversityHeraklionGreece; Ministry of HealthKuala LumpurMalaysia; Mashhad University of Medical SciencesMashhadIslamic Republic of Iran; Iran University of Medical SciencesTehranIslamic Republic of Iran; University of PaduaPaduaItaly; Agricultural University of AthensAthensGreece; Institute of EndocrinologyPragueCzech Republic; Centro de Estudios sobre Nutrición InfantilBuenos AiresArgentina; Center for Diabetes and Endocrine CareSrinagarIndia; University of Public HealthYangonMyanmar; Medical University of GdanskGdanskPoland; National Institute of Public HealthPragueCzech Republic; University Hospital Center ZagrebZagrebCroatia; Peking UniversityBeijingChina; Duke UniversityDurhamUnited States; Peking University First HospitalBeijingChina; University of LeuvenLeuvenBelgium; Capital Medical University Beijing An Zhen HospitalBeijingChina; Peking University First HospitalBeijingChina; Chinese Center for Disease Control and PreventionBeijingChina; Jiangsu Provincial Center for Disease Control and PreventionNanjingChina; Vanderbilt UniversityNashvilleUnited States; Sun Yat-sen UniversityGuangzhouChina; West Kazakhstan Medical UniversityAktobeKazakhstan; Chinese Center for Disease Control and PreventionBeijingChina; Inner Mongolia Medical UniversityHohhotChina; Institut National de la Santé et de la Recherche MédicaleVillejuifFrance; Paris UniversityParisFrance; Agency for Preventive and Social MedicineBregenzAustria; Universidad de la RepúblicaMontevideoUruguay; Instituto Conmemorativo Gorgas de Estudios de la SaludPanama CityPanama; Przedszkole No. 81WarsawPoland; Imperial College LondonLondonUnited Kingdom; University of GhanaAccraGhana; Imperial College LondonLondonUnited Kingdom; University of Calgary Canada; McGill University Canada

**Keywords:** underweight, obesity, BMI, None

## Abstract

From 1985 to 2016, the prevalence of underweight decreased, and that of obesity and severe obesity increased, in most regions, with significant variation in the magnitude of these changes across regions. We investigated how much change in mean body mass index (BMI) explains changes in the prevalence of underweight, obesity, and severe obesity in different regions using data from 2896 population-based studies with 187 million participants. Changes in the prevalence of underweight and total obesity, and to a lesser extent severe obesity, are largely driven by shifts in the distribution of BMI, with smaller contributions from changes in the shape of the distribution. In East and Southeast Asia and sub-Saharan Africa, the underweight tail of the BMI distribution was left behind as the distribution shifted. There is a need for policies that address all forms of malnutrition by making healthy foods accessible and affordable, while restricting unhealthy foods through fiscal and regulatory restrictions.

## Introduction

Underweight as well as obesity can lead to adverse health outcomes (Prospective Studies Collaboration et al., 2009; Global BMI Mortality Collaboration, 2016; Emerging Risk Factors Collaboration et al., 2011). For at least four decades, the prevalence of underweight has decreased, and that of obesity has increased, in most countries with significant variation in the magnitude of these changes across regions of the world (NCD Risk Factor Collaboration (NCD-RisC), 2017a; NCD Risk Factor Collaboration (NCD-RisC), 2019).

**Figure 1.**
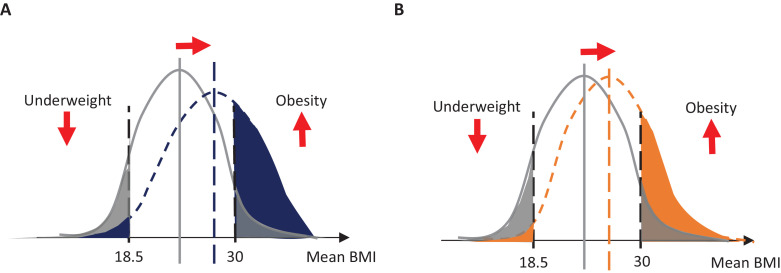
Schematic diagram of contribution of change in mean body mass index (BMI) to change in total prevalence of underweight or obesity. (**A**) Change in the prevalence of underweight and obesity if the distribution shifts, represented by a change in its mean and its shape. In this example, the change (shown as the difference between blue and gray) results in a small decrease of underweight and a large increase in obesity. (**B**) Change in the prevalence of underweight and obesity when only mean BMI changes (shown as the difference between orange and gray), without a change in the shape of the distribution.

A shift in the whole distribution of body mass index (BMI) would simultaneously affect mean BMI as well as the prevalence of underweight and obesity (Razak et al., 2018; Rose and Day, 1990). In contrast, changes in the shape of BMI distribution – for example, widening or narrowing of the BMI distribution, becoming more or less skewed, or having a thinner or thicker tail – would affect the prevalence of underweight and obesity with only small impacts on the population mean, as shown schematically in Figure 1. Understanding these two mechanisms is essential as they may require different public health and clinical responses (Penman and Johnson, 2006). But it is unclear how much the two mechanisms have contributed to the observed decline in underweight and rise in obesity in different world regions.

Some studies have investigated whether the rise in obesity or the decrease of underweight over time, or differences across countries, were due to a shift in BMI distribution versus changes in the low- or high-BMI tails of the distribution (Razak et al., 2018; Wang et al., 2007; Wagner et al., 2019; Vaezghasemi et al., 2016; Razak et al., 2013; Popkin and Slining, 2013; Popkin, 2010; Peeters et al., 2015; Ouyang et al., 2015; Monteiro et al., 2002; Midthjell et al., 2013; Lebel et al., 2018; Khang and Yun, 2010; Helmchen and Henderson, 2004; Hayes et al., 2015; Green et al., 2016; Flegal and Troiano, 2000; Stenholm et al., 2015; Hayes et al., 2017; Flegal et al., 2012; Bovet et al., 2008). Most of these studies focused on a single or small number of countries over relatively short durations or covered only one sex, a narrow age group, or specific social groups. To understand whether weight gain occurs across all BMI levels or disproportionately affects the underweight or obese segments of the distribution, and how this phenomenon varies geographically, there is a need for a population-based study that simultaneously investigates both underweight and obesity in relation to mean BMI in different regions of the world. We used a comprehensive global database to investigate how much change in mean BMI can explain the corresponding changes in prevalence of adults with underweight (defined as BMI <18.5 kg/m^2^), total obesity (BMI ≥30 kg/m^2^), and severe obesity (BMI ≥35 kg/m^2^) over three decades from 1985 to 2016 in different regions of the world.

## Results

### Data sources

**Figure 2.**
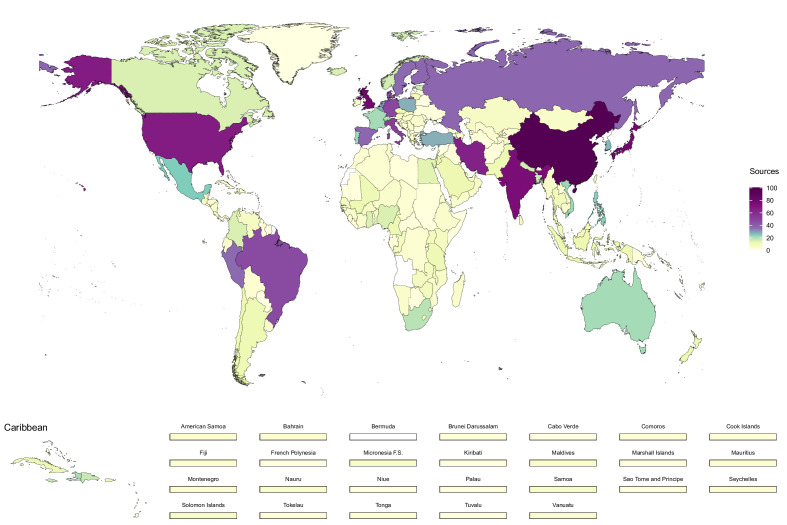
Number of data sources with participants aged 20-79 years.

The Non-Communicable Disease Risk Factor Collaboration (NCD-RisC) database contains 2896 population-based studies conducted from 1985 to 2019 with height and weight measurements of 187 million participants. Of these, 2033 studies had measurements of height and weight on 132.6 million participants aged 20–79 years. The number of studies with participants aged 20–79 years in different regions ranged from 53 in Oceania to 637 in the high-income western region. The number of data sources by country is shown in Figure 2. The list of data sources and their characteristics is provided in Supplementary file 4.

### Change in mean BMI and prevalence of underweight, obesity, and severe obesity by region

In 2016, the age-standardised prevalence of underweight was highest (>16% in different sex-age groups) in South Asia; it was <2.5% in Central and Eastern Europe; the high-income western region; Latin America and the Caribbean; Oceania; and Central Asia, the Middle East, and North Africa for most age and sex groups. The age-standardised prevalence of obesity was highest (>24%) in these same regions for most age and sex groups. It was lowest (<7%) in men and women from South Asia; the high-income Asia Pacific region; and men from sub-Saharan Africa. The age-standardised prevalence of severe obesity was highest (12–18%) in women aged 50–79 years from Central Asia, the Middle East, and North Africa; the high-income western region; Central and Eastern Europe; and Latin America and the Caribbean. It was lowest (<2%) in South Asia; East and Southeast Asia; the high-income Asia Pacific region; and men in sub-Saharan Africa.

**Figure 3.**
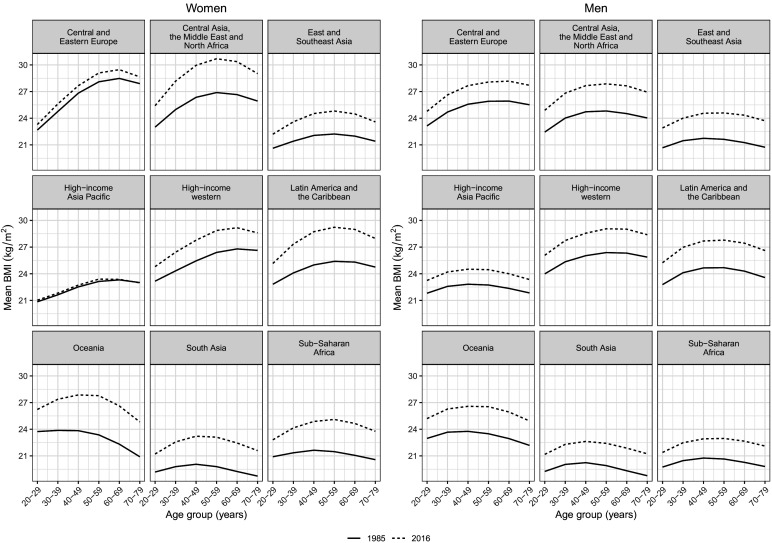
Change in mean body mass index (BMI) from 1985 to 2016 by region, sex, and age group.

**Figure 4.**
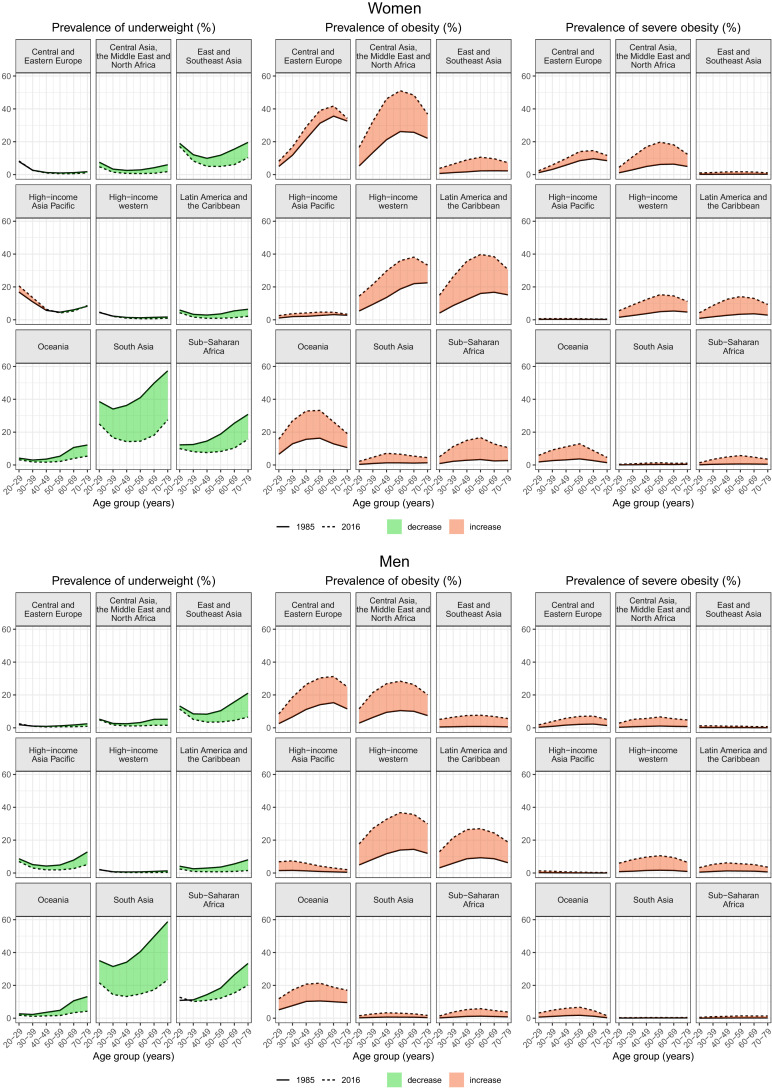
Change in prevalence of underweight, obesity, and severe obesity from 1985 to 2016 by region, sex, and age group.

From 1985 to 2016, age-standardised mean BMI increased by 1–4 kg/m^2^ in all regions, with the exception of women in the high-income Asia Pacific region and Central and Eastern Europe whose mean BMI changed by less than 1 kg/m^2^ (Figure 3). The prevalence of underweight decreased or stayed unchanged and that of obesity and severe obesity increased from 1985 to 2016 in all regions, with the exception of an increase in the prevalence of underweight in younger women in the high-income Asia Pacific region. The largest absolute decrease in underweight prevalence from 1985 to 2016 was seen in South Asia; East and Southeast Asia; and sub-Saharan Africa, where it declined by 14–35 percentage points in different age–sex groups (Figure 4). Nonetheless, underweight prevalence remained higher in these three regions than elsewhere in 2016. Prevalence of underweight changed only marginally in regions such as Central and Eastern Europe and the high-income western region, where prevalence was already low in 1985.

The largest absolute increase in obesity prevalence from 1985 to 2016 occurred in Central Asia, the Middle East, and North Africa; the high-income western region; Latin America and the Caribbean; Oceania (women); and Central and Eastern Europe (men) (Figure 4). Women in these regions also experienced the largest increase in severe obesity prevalence, along with men in the high-income western region. In these regions and sexes, obesity prevalence increased by 16–24 percentage points in different age groups, and severe obesity increased by 5–13 percentage points. The increase in obesity was less than five percentage points in the high-income Asia Pacific region; South Asia; and in men in sub-Saharan Africa; in the same regions, along with East and Southeast Asia, the increase in severe obesity was less than two percentage points.

### Associations of underweight, obesity, and severe obesity prevalence with mean BMI

There was a strong association between the prevalence of underweight, obesity, and severe obesity with mean BMI as measured by R-squared of the regressions of prevalence on mean (Supplementary files 1 and 2). These indicate that 93% (men) and 96% (women) of variation in obesity, and between 83% and 92% of variation in underweight and severe obesity, were explained by mean BMI and other variables (year, region, and age group) in the regression models. The coefficients of the mean BMI terms represent the changes in (probit-transformed) prevalence associated with a unit change in mean BMI, and their interactions with region represent variations in this association across regions. For all three outcomes, the association of prevalence with mean BMI varied across regions.

The inter-regional variation in the prevalence–mean association was stronger for obesity and severe obesity than underweight, as seen in larger inter-regional range of the interaction terms. The extent to which prevalence changes with any variation in mean BMI in each region is an outcome of the main BMI term and its interaction with region; to be epidemiologically interpretable, this will have to be converted from probit-transformed to original prevalence scale. For example, in the year 2016, for women aged 50–59 years, at a mean BMI of 25 kg/m^2^ (which was approximately the global age-standardised mean level of BMI) (NCD Risk Factor Collaboration (NCD-RisC), 2017a), prevalence of underweight would have varied by seven percentage points across regions, being lowest in Central and Eastern Europe and highest in sub-Saharan Africa; a unit increase in mean BMI would have been associated with a relative change in prevalence ranging from −49% in the high-income Asia Pacific region to −14% in Oceania. Also for women aged 50–59 years and a mean BMI of 25 kg/m^2^, the prevalence of obesity and severe obesity would both have been the highest in Oceania and the lowest in the high-income Asia Pacific region, with a difference of 12 and 6 percentage points, respectively, for the two outcomes; a unit increase in mean BMI would have been associated with a relative change ranging from 21% to 46% for obesity and from 30% to 59% for severe obesity, the smallest change for both being in Oceania and the largest in East and Southeast Asia. There was similar inter-regional variation in the other year–age–sex strata.

### Contribution of mean BMI to changes in underweight and obesity prevalence

**Figure 5.**
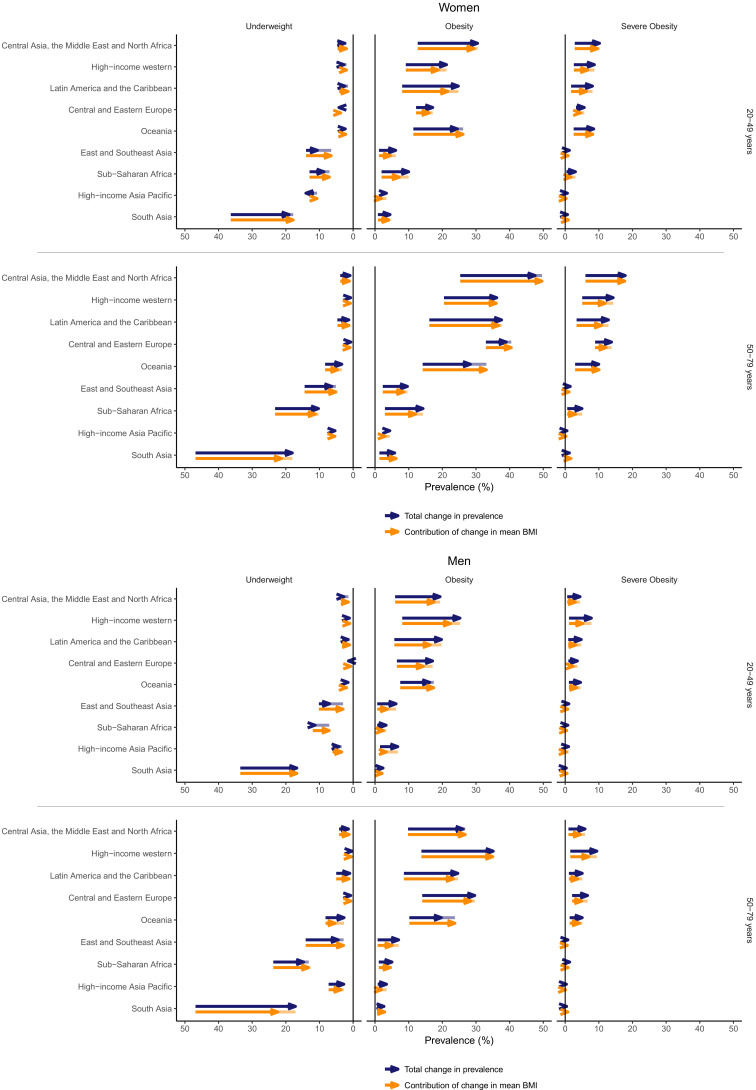
Contribution of change in mean body mass index (BMI) to total change from 1985 to 2016 in prevalence of underweight, obesity, or severe obesity by region, sex, and age group. Blue arrows show the total change in prevalence of underweight, obesity, or severe obesity. Orange arrows show the contribution of change in mean BMI to the change in prevalence. The difference between these two arrows is shown with a line, whose colour follows the shorter arrow.

The rise in mean BMI accounted for >82% of the decline in underweight in different age–sex groups in South Asia, where underweight prevalence declined by over 16 percentage points for all age–sex groups (Figure 5). The remainder of the decline was due to change in the shape of the BMI distribution which reduced underweight prevalence beyond the effects of the population mean. In contrast, in sub-Saharan Africa and East and Southeast Asia, the total change in prevalence of underweight (3–12 percentage points) was 20–80% less than what was expected based on the increase in mean BMI (Figure 5). In other words, in these regions the underweight tail of the BMI distribution was left behind as the distribution shifted.

Where obesity increased the most – Central Asia, the Middle East, and North Africa; Latin America and the Caribbean; and the high-income western region – the rise in mean BMI accounted for over three quarters of the increase in different age–sex groups (Figure 5). In Oceania, the actual rise in prevalence of obesity (8–14 percentage points for all age–sex groups) was about two-thirds to one-half of what would have been expected by the observed increase in mean BMI in men and women (Figure 5). Change in mean BMI consistently accounted for a smaller share of the change in severe obesity than it did for change in total obesity. Specifically, in regions where prevalence of severe obesity changed by more than one percentage point, the contribution of change in mean BMI to change in severe obesity in different regions was 53–90% of the corresponding contribution for total obesity (Figure 5).

In other regions, the change in the prevalence of underweight, obesity, or severe obesity was too small for the contribution of change in mean BMI to be epidemiologically relevant (Figure 5).

## Discussion

We found that the trends in the prevalence of underweight, total obesity, and, to a lesser extent, severe obesity are largely driven by shifts in the distribution of BMI, with smaller contributions from changes in the shape of the distribution. The notable exceptions to this pattern were the decline in the prevalence of underweight in East and Southeast Asia and sub-Saharan Africa and the rise of obesity in Oceania, which were both smaller than expected based on change in mean BMI.

Our results are consistent with a recent cross-sectional study (Razak et al., 2018) using data from women in low- and middle-income countries that found a strong association between mean BMI and prevalence of obesity, and a moderate association between mean BMI and prevalence of underweight. Being cross-sectional, this study did not consider changes over time, as we have. Our results are also consistent with another study which found that changes in median BMI contributed more than 75% to the increase in obesity in the USA from 1980 to 2000 (Helmchen and Henderson, 2004).

Previous studies used one or more approaches to investigate changes in population BMI distribution: some analysed percentiles of the BMI distribution (Wagner et al., 2019; Vaezghasemi et al., 2016; Razak et al., 2013; Popkin and Slining, 2013; Popkin, 2010; Peeters et al., 2015; Ouyang et al., 2015; Lebel et al., 2018; Hayes et al., 2015), others focused on the change in prevalence above or below pre-specified BMI thresholds (Wang et al., 2007; Razak et al., 2013; Popkin, 2010; Peeters et al., 2015; Ouyang et al., 2015; Khang and Yun, 2010; Flegal and Troiano, 2000), or evaluated how the shape of the BMI distribution has changed via examining metrics such as standard deviation and skewness (Peeters et al., 2015; Ouyang et al., 2015; Lebel et al., 2018; Khang and Yun, 2010; Hayes et al., 2015; Flegal and Troiano, 2000). Most of these studies reached the same conclusion as our study that, as the BMI distribution shifts upwards, the prevalence of underweight declines somewhat more slowly than the prevalence of obesity rises.

Our study has strengths in scope, data, and methods: the strengths of our study include presenting the first global analysis of how much the rise in mean BMI versus changes in the shape of its distribution influenced changes in both underweight and obesity prevalence. We used an unprecedented amount of data from different regions covering three decades and used only measured data on height and weight to avoid biases in self-reported data.

As with all global analyses, our study has some limitations. Despite using the most comprehensive global collection of population-based studies to date, some regions, especially Oceania and sub-Saharan Africa, had less data, especially early in our analysis period. Further, given the large number of age, sex, and region subgroups of population in our analysis, and its long duration, it was not possible to visually explore how the shape of BMI distribution has changed in the underweight and obesity ranges where changes in the mean did not fully explain change in prevalence. Finally, there are variations in characteristics such as response rate and measurement protocol across studies. Some of these, such as exclusion of studies with self-reported height and weight, were a part of our inclusion and exclusion criteria. Others may affect population mean or prevalence.

The finding that the majority of the rise in the prevalence of obesity from 1985 to 2016 is mostly the result of a distributional shift points towards an important role for societal drivers, including lower availability and higher price of healthy and fresh foods compared to caloric-dense and nutrient-deficient foods (Swinburn et al., 2011), and mechanisation of work and motorisation of transport throughout the world that have reduced energy expenditure in populations around the world (NCD Risk Factor Collaboration (NCD-RisC), 2019; Ng and Popkin, 2012). First, although there is a genetic component to BMI at the individual level (Silventoinen et al., 2017; Silventoinen et al., 2016; Locke et al., 2015; Brandkvist et al., 2019), genetics explain only a small part of changes over time, especially when people have access to healthy food and living environment. When the environment becomes more obesogenic, some people or population subgroups may gain more weight than others, implying that the environment remains the main contributor (Brandkvist et al., 2019). This interplay of genetic predisposition and changes in the environment might account for some of the excess rise in obesity and severe obesity beyond the effect of distributional shift alone (Brandkvist et al., 2019). The exception observed in Oceania is possibly because in 1985 obesity prevalence in this region was already so high (NCD Risk Factor Collaboration (NCD-RisC), 2017a) that the rise in BMI did not change overall obesity status (but there was a substantial increase in those with severe obesity, mostly accounted for by the change in mean BMI). The smaller decline in underweight than expected in sub-Saharan Africa and East and Southeast Asia may be because underweight is associated with lower socioeconomic status, food insecurity, and for sub-Saharan Africa widening difference between rural and urban BMI levels which is different from other regions (NCD Risk Factor Collaboration (NCD-RisC), 2019; Brandkvist et al., 2019; Di Cesare et al., 2015; Subramanian and Smith, 2006; Di Cesare et al., 2013). If the benefits of economic development do not sufficiently reach the poor, they remain nutritionally vulnerable, as has been seen for height and weight during childhood and adolescence (NCD Risk Factor Collaboration (NCD-RisC), 2020; Subramanyam et al., 2011; Sanchez and Swaminathan, 2005; Pongou et al., 2006; Haddad, 2003; Stevens et al., 2012). Together with the rise in mean BMI and obesity (and short stature which is not a topic of this paper but addressed in other studies) (NCD Risk Factor Collaboration (NCD-RisC), 2020; Stevens et al., 2012; NCD Risk Factor Collaboration (NCD-RisC), 2016a), this creates a double burden of malnutrition (Popkin et al., 2020).

In summary, we found that the worldwide rise in obesity and the decline in underweight are primarily driven by the shift in the population distribution of BMI. At the same time, there is an evidence of both excess obesity, and especially severe obesity, and persistent underweight beyond the distributional shift in some regions, which may be related to growing social inequalities that restrict access to healthy foods in those at highest risk of undernutrition (Popkin et al., 2020; Wells et al., 2020; Darmon and Drewnowski, 2015). The response to these trends must motivate ‘double-duty actions’ that prevent and tackle all forms of malnutrition through both fiscal and regulatory restrictions on unhealthy foods, and making healthy foods available, accessible, and affordable especially to those at high risks of underweight and obesity (Powell et al., 2013; Hawkes et al., 2020; Bleich et al., 2017).

## Materials and methods

### Study design

Our aim was to quantify, for all regions of the world, how much of the change in prevalence of underweight (defined as BMI <18.5 kg/m^2^), (total) obesity (BMI ≥30 kg/m^2^), and severe obesity (BMI ≥35 kg/m^2^) among men and women aged 20–79 years from 1985 to 2016 could be accounted for by change in mean BMI. In the first step, we used data from a global database of human anthropometry to estimate the associations of the prevalence of underweight, prevalence of obesity, or prevalence of severe obesity with population mean BMI, including how the association varies in relation to age group and region. We then used the fitted association to estimate the contribution of change in the population mean BMI to change in the prevalence of underweight, obesity, or severe obesity in different regions.

### Data sources

In the first step of the analysis, we estimated the prevalence-mean associations, using data from a comprehensive database on cardiometabolic risk factors collated by NCD-RisC as described below. In the second step, we needed consistent estimates of mean BMI for all regions. For this purpose, we used data from a recent comprehensive analysis of worldwide trends in mean BMI from 1985 to 2016 (NCD Risk Factor Collaboration (NCD-RisC), 2017a) which had fitted a Bayesian hierarchical model to the NCD-RisC data.

Data in the NCD-RisC database were obtained from publicly available multi-country and national measurement surveys (e.g., Demographic and Health Surveys, WHO-STEPwise approach to Surveillance [STEPS] surveys, and those identified via the Inter-University Consortium for Political and Social Research and European Health Interview and Health Examination Surveys Database). With the help of the World Health Organization (WHO) and its regional and country offices as well as the World Heart Federation, we identified and accessed population-based survey data from national health and statistical agencies. We searched and reviewed published studies as detailed previously (NCD Risk Factor Collaboration (NCD-RisC), 2017a) and invited eligible studies to join NCD-RisC, as we did with data holders from earlier pooled analysis of cardiometabolic risk factors (Finucane et al., 2011; Farzadfar et al., 2011; Danaei et al., 2011a; Danaei et al., 2011b).

### Data inclusion and exclusion

We carefully checked that each data source met our inclusion criteria as listed below:

- measurement data on height and weight were available;
- study participants were 5 years of age and older (as described earlier data used here were for those 20–79 years);
- data were collected using a probabilistic sampling method with a defined sampling frame;
- data were from population samples at the national, sub-national (i.e., covering one or more sub-national regions, with more than three urban or five rural communities), or community level; and
- data were from the countries and territories listed in Supplementary file 3.

We excluded all data sources that only used self-reported weight and height without a measurement component because these data are subject to biases that vary with geography, time, age, sex, and socioeconomic characteristics (Tolonen et al., 2014; Hayes et al., 2011; Ezzati et al., 2006). We also excluded data on population subgroups whose anthropometric status may differ systematically from the general population, including

- studies that had included or excluded people based on their health status or cardiovascular risk;
- studies whose participants were only ethnic minorities;
- specific educational, occupational, or socioeconomic subgroups, with the exception noted below; and
- those recruited through health facilities, with the exception noted below.

We included school-based data in countries and age–sex groups with enrolment of 70% or higher. We also included data whose sampling frame was health insurance schemes in countries where at least 80% of the population were insured. Finally, we included data collected through general practice and primary care systems in high-income and Central European countries with universal insurance because contact with the primary care systems tends to be as good as or better than the response rates for population-based surveys. The list of data sources with participants aged 20–79 years and their characteristics is provided in Supplementary file 4, with additional information in Source data 1.

Duplicate data were identified by comparing studies from the same country and year, and then discarded. All NCD-RisC members are also periodically asked to review the list of sources from their country, to verify that the included data meet the inclusion criteria and are not duplicates, and to suggest additional sources. The NCD-RisC database is continuously updated through all the above routes. For each data source, we recorded the study population, sampling approach, years of measurement, and measurement methods. Only population-representative data were included, and these were assessed in terms of whether they covered the whole country, multiple sub-national regions, or one or a small number of communities, and whether rural, urban, or both participants were included. All submitted data were checked independently by at least two persons. Questions and clarifications were discussed with NCD-RisC members and resolved before data were incorporated in the database.

We calculated mean BMI and the associated standard errors by sex and age. All analyses incorporated sample weights and complex survey design, when applicable, in calculating summary statistics, with computer code provided to NCD-RisC members who requested assistance.

Additionally, summary statistics for nationally representative data from sources that were identified but not accessed via the above routes were extracted from published reports. Data were also extracted for nine STEPS surveys that were not publicly available, one Countrywide Integrated Non-communicable Diseases Intervention survey, and five sites of the WHO Multinational MONItoring of trends and determinants in CArdiovascular disease (MONICA) project that were not deposited in the MONICA Data Centre. We also included data from a previous global data pooling study (Finucane et al., 2011) when they had not been accessed as described above.

Here, to estimate the association of underweight, obesity, and severe obesity prevalence with mean BMI as described below, we used data collected from 1985 to 2019 with measured height and weight among men and women aged 20–79 years, in 10-year age groups. Data that did not cover the complete 10-year age groups, for example, 25–29 or 60–64 years, were excluded. We included data from study–age–sex strata with a prevalence between 0 and 1 to allow probit transformation and with at least 25 participants in each stratum. These data were summarised into 11,652 study–age–sex-specific pairs of mean and prevalence of adults with underweight, obesity, or severe obesity.

### Statistical methods

Anonymised data from studies in the NCD-RisC database were reanalysed according to a common protocol. We calculated mean BMI and prevalence of underweight, obesity, and severe obesity by sex and age group in each study in the NCD-RisC database from 1985 to 2019. We used data through 2019 so that the prevalence–mean association is informed by as much data as possible. All calculations took into account complex survey design where relevant. We excluded study–age–sex groups with less than 25 participants because their means and prevalence have larger uncertainty.

We then estimated the relationship between probit-transformed prevalence of underweight, obesity, and severe obesity and mean BMI in a regression model, separately for each of these prevalences. The correlation coefficient between mean BMI and median BMI was ≥0.98 in different age–sex groups, indicating a strong correlation between the two. In our statistical model, described below, the prevalence of underweight, obesity, or severe obesity depends on population mean BMI as well as on age group, region, and year.

All analyses were done separately for men and women. We chose a probit-transformed prevalence because it changes in an approximately linear manner as the mean changes, thus providing a better fit to the data. The regressions also included age group in 10-year bands, region and the year when the data were collected. The regions, used in previous analyses of cardiometabolic risk factors (NCD Risk Factor Collaboration (NCD-RisC), 2017a; NCD Risk Factor Collaboration (NCD-RisC), 2019; NCD Risk Factor Collaboration (NCD-RisC), 2020; NCD Risk Factor Collaboration (NCD-RisC), 2016a; NCD Risk Factor Collaboration (NCD-RisC), 2018; NCD Risk Factor Collaboration (NCD-RisC), 2017b; NCD Risk Factor Collaboration (NCD-RisC), 2016b; NCD Risk Factor Collaboration (NCD-RisC), 2016c), were Central and Eastern Europe; Central Asia, the Middle East, and North Africa; East and Southeast Asia; high-income Asia Pacific region; high-income western region; Latin America and the Caribbean; Oceania; South Asia; and sub-Saharan Africa. Countries in each region are listed in Supplementary file 3. The model also included interactions between mean BMI and age group, mean BMI and region, age group and region, age group and year, and year and region. These terms allowed the prevalence–mean association to vary by age group, region, and over time. The models were fitted in statistical software R (version 4.0.2) (Source code 1). The coefficients of the regression models are presented in Supplementary files 1 and 2.

We used the fitted regressions to quantify how much of the change over time in the prevalence of underweight, obesity, or severe obesity in each region and age group can be explained by the corresponding change in mean BMI. To do so, we first used the region- and age–sex-specific mean BMI in 1985 and 2016 in the fitted association and then estimated the total change in prevalence of underweight, obesity, or severe obesity by region. The mean BMI values were from a recent comprehensive analysis of worldwide trends in mean BMI (NCD Risk Factor Collaboration (NCD-RisC), 2017a) and are listed in Supplementary file 5. We then calculated the contribution of change in mean BMI to the change in prevalence of underweight or obesity by allowing mean BMI for each age group and region to change over time, while keeping year fixed at 1985. Results were calculated by 10-year age group and then aggregated into two age bands, 20–49 and 50–79 years, by taking weighted average of age-specific results using weights from the WHO standard population (Ahmad et al., 2001).

## Data Availability

Names and characteristics of data sources included in this pooling analysis are listed in Supplementary file 4. Of these data, some are from public sources, for which we have provided the data in Source data 1. Others are from individual researchers and/or from government and international agencies; these should be requested from the data holders on a study to study basis using the information in Source data 1.

## Additional files

Supplementary file 1. Coefficients of the regression of probit-transformed prevalence of underweight, obesity, and severe obesity in women on mean body mass index.

Supplementary file 2. Coefficients of the regression of probit-transformed prevalence of underweight, obesity, and severe obesity in men on mean body mass index.

Supplementary file 3. List of analysis regions and countries in each region.

Supplementary file 4. List of data sources used in the analysis and their characteristics.

Supplementary file 5. Mean body mass index estimates (kg/m^2^) in 1985 and 2016 stratified by region, gender, and age group.

Source data 1. Data from studies in the Non-Communicable Disease Risk Factor Collaboration database conducted from 1985 to 2019 with participants aged 20–79 years old.

Source code 1. Liner regression model used for the analysis of the association of prevalence of underweight, total obesity, and severe obesity with mean body mass index.

Transparent reporting form
