# Supplementary material for: Heterogeneous contributions of change in population distribution of body mass index to change in obesity and underweight
Source: eLife. 2021 Mar 9;10:e60060. doi: 10.7554/eLife.60060 (PMC7943191; doi:10.7554/eLife.60060)
Supplement: Supplementary file 1. [file elife-60060-supp1.docx]

**Supplementary file 1.** Coefficients of the regression of probit-transformed prevalence of underweight, obesity and severe obesity in women on mean body mass index.

| **Variable** | **Coefficient for underweight** | **Coefficient for obesity** | **Coefficient for severe obesity** |
| --- | --- | --- | --- |
| Intercept^#^ | -1.6 (-1.7, -1.5)*** | -0.74 (-0.79, -0.68)*** | -1.4 (-1.4, -1.3)*** |
| Mean BMI (per one more unit kg/m^2^)^#^ | -0.089 (-0.12, -0.06)*** | 0.27 (0.26, 0.29)*** | 0.24 (0.22, 0.26)*** |
| Age group (years)^#^ |  |  |  |
| 20-29 | Reference | Reference | Reference |
| 30-39 | -0.34 (-0.45, -0.24)*** | -0.18 (-0.24, -0.13)*** | -0.15 (-0.23, -0.073)*** |
| 40-49 | -0.5 (-0.65, -0.36)*** | -0.23 (-0.31, -0.16)*** | -0.3 (-0.4, -0.2)*** |
| 50-59 | -0.54 (-0.72, -0.35)*** | -0.22 (-0.32, -0.13)*** | -0.34 (-0.46, -0.21)*** |
| 60-69 | -0.43 (-0.64, -0.22)*** | -0.22 (-0.32, -0.11)*** | -0.34 (-0.48, -0.2)*** |
| 70-79 | -0.28 (-0.47, -0.084)** | -0.21 (-0.3, -0.11)*** | -0.33 (-0.46, -0.2)*** |
| Year (per one more recent year since 1985)^#^ | 0.0025 (-0.0011, 0.006) | 0.0024 (0.00062, 0.0042)** | 0.0035 (0.001, 0.006)** |
| Region^#^ |  |  |  |
| Central and Eastern Europe | Reference | Reference | Reference |
| Central Asia, the Middle East and North Africa | -0.1 (-0.21, 0.0043) | -0.16 (-0.22, -0.1)*** | -0.2 (-0.28, -0.12)*** |
| East and Southeast Asia | -0.21 (-0.32, -0.091)*** | 0.0075 (-0.059, 0.074) | -0.03 (-0.13, 0.07) |
| High-income Asia Pacific | -0.45 (-0.63, -0.27)*** | -0.12 (-0.23, -0.014)* | -0.34 (-0.5, -0.18)*** |
| High-income western | -0.19 (-0.3, -0.091)*** | -0.11 (-0.17, -0.058)*** | 0.018 (-0.062, 0.098) |
| Latin America and the Caribbean | -0.24 (-0.34, -0.13)*** | -0.21 (-0.27, -0.15)*** | -0.23 (-0.31, -0.15)*** |
| Oceania | -0.21 (-0.4, -0.012)* | -0.34 (-0.43, -0.25)*** | -0.24 (-0.36, -0.11)*** |
| South Asia | -0.019 (-0.15, 0.11) | -0.013 (-0.086, 0.059) | -0.062 (-0.17, 0.043) |
| Sub-Saharan Africa | -0.077 (-0.18, 0.026) | -0.21 (-0.27, -0.16)*** | -0.22 (-0.3, -0.14)*** |
| Region × mean BMI (per one more unit kg/m^2^) |  |  |  |
| Central and Eastern Europe | Reference | Reference | Reference |
| Central Asia, the Middle East and North Africa | -0.05 (-0.079, -0.022)*** | -0.031 (-0.045, -0.018)*** | -0.018 (-0.036, 0.0003) |
| East and Southeast Asia | -0.14 (-0.17, -0.11)*** | 0.04 (0.026, 0.054)*** | 0.015 (-0.0051, 0.035) |
| High-income Asia Pacific | -0.17 (-0.21, -0.13)*** | -0.016 (-0.038, 0.0054) | -0.061 (-0.091, -0.031)*** |
| High-income western | 0.0062 (-0.022, 0.035) | -0.013 (-0.026, 0.00096) | 0.0057 (-0.012, 0.024) |
| Latin America and the Caribbean | -0.064 (-0.094, -0.035)*** | -0.034 (-0.048, -0.02)*** | -0.03 (-0.049, -0.011)** |
| Oceania | 0.023 (-0.0091, 0.054) | -0.063 (-0.077, -0.048)*** | -0.047 (-0.066, -0.028)*** |
| South Asia | -0.13 (-0.16, -0.1)*** | 0.0067 (-0.0079, 0.021) | -0.00092 (-0.021, 0.019) |
| Sub-Saharan Africa | -0.043 (-0.071, -0.016)** | -0.0087 (-0.022, 0.0045) | -0.0094 (-0.027, 0.0084) |
| Region × age group (years) |  |  |  |
| Central and Eastern Europe | Reference | Reference | Reference |
| Central Asia, the Middle East and North Africa |  |  |  |
| 20-29 | Reference | Reference | Reference |
| 30-39 | 0.22 (0.094, 0.34)*** | 0.15 (0.086, 0.22)*** | 0.066 (-0.022, 0.15) |
| 40-49 | 0.41 (0.25, 0.58)*** | 0.25 (0.17, 0.34)*** | 0.19 (0.076, 0.3)*** |
| 50-59 | 0.53 (0.33, 0.73)*** | 0.29 (0.19, 0.39)*** | 0.24 (0.11, 0.38)*** |
| 60-69 | 0.54 (0.32, 0.76)*** | 0.28 (0.17, 0.39)*** | 0.29 (0.14, 0.44)*** |
| 70-79 | 0.5 (0.29, 0.71)*** | 0.26 (0.15, 0.36)*** | 0.26 (0.12, 0.4)*** |
| East and Southeast Asia |  |  |  |
| 20-29 | Reference | Reference | Reference |
| 30-39 | 0.23 (0.11, 0.36)*** | -0.07 (-0.14, -0.0016)* | -0.2 (-0.3, -0.1)*** |
| 40-49 | 0.38 (0.22, 0.54)*** | -0.12 (-0.21, -0.041)** | -0.19 (-0.31, -0.077)** |
| 50-59 | 0.52 (0.32, 0.72)*** | -0.091 (-0.19, 0.0097) | -0.19 (-0.33, -0.053)** |
| 60-69 | 0.46 (0.23, 0.69)*** | -0.04 (-0.15, 0.074) | -0.15 (-0.31, 0.0041) |
| 70-79 | 0.34 (0.12, 0.56)** | 0.058 (-0.052, 0.17) | -0.11 (-0.26, 0.04) |
| High-income Asia Pacific |  |  |  |
| 20-29 | Reference | Reference | Reference |
| 30-39 | 0.28 (0.14, 0.42)*** | -0.00047 (-0.078, 0.077) | -0.091 (-0.2, 0.016) |
| 40-49 | 0.29 (0.11, 0.46)** | -0.15 (-0.25, -0.058)** | -0.12 (-0.25, 0.0044) |
| 50-59 | 0.34 (0.12, 0.56)** | -0.23 (-0.34, -0.12)*** | -0.23 (-0.38, -0.073)** |
| 60-69 | 0.36 (0.11, 0.6)** | -0.21 (-0.33, -0.08)** | -0.31 (-0.49, -0.14)*** |
| 70-79 | 0.3 (0.071, 0.54)* | -0.24 (-0.36, -0.12)*** | -0.32 (-0.49, -0.16)*** |
| High-income western |  |  |  |
| 20-29 | Reference | Reference | Reference |
| 30-39 | 0.13 (0.013, 0.24)* | 0.084 (0.025, 0.14)** | 0.019 (-0.063, 0.1) |
| 40-49 | 0.16 (0.0059, 0.31)* | 0.11 (0.034, 0.19)** | 0.07 (-0.033, 0.17) |
| 50-59 | 0.17 (-0.022, 0.36) | 0.098 (0.0037, 0.19)* | 0.047 (-0.081, 0.18) |
| 60-69 | 0.15 (-0.062, 0.37) | 0.1 (-0.0029, 0.21) | -0.0028 (-0.14, 0.14) |
| 70-79 | 0.072 (-0.13, 0.27) | 0.11 (0.015, 0.21)* | -0.063 (-0.19, 0.068) |
| Latin America and the Caribbean |  |  |  |
| 20-29 | Reference | Reference | Reference |
| 30-39 | 0.26 (0.14, 0.38)*** | 0.15 (0.089, 0.22)*** | 0.092 (0.0047, 0.18)* |
| 40-49 | 0.46 (0.3, 0.61)*** | 0.23 (0.15, 0.31)*** | 0.22 (0.11, 0.33)*** |
| 50-59 | 0.61 (0.41, 0.81)*** | 0.28 (0.18, 0.38)*** | 0.28 (0.15, 0.42)*** |
| 60-69 | 0.66 (0.44, 0.87)*** | 0.3 (0.19, 0.41)*** | 0.32 (0.17, 0.46)*** |
| 70-79 | 0.53 (0.32, 0.73)*** | 0.29 (0.19, 0.39)*** | 0.27 (0.13, 0.41)*** |
| Oceania |  |  |  |
| 20-29 | Reference | Reference | Reference |
| 30-39 | 0.2 (0.00064, 0.4)* | 0.42 (0.32, 0.52)*** | 0.2 (0.065, 0.34)** |
| 40-49 | 0.4 (0.18, 0.62)*** | 0.58 (0.47, 0.69)*** | 0.4 (0.25, 0.54)*** |
| 50-59 | 0.55 (0.31, 0.8)*** | 0.63 (0.51, 0.76)*** | 0.56 (0.39, 0.72)*** |
| 60-69 | 0.68 (0.35, 1)*** | 0.62 (0.47, 0.76)*** | 0.53 (0.34, 0.72)*** |
| 70-79 | 0.51 (0.15, 0.87)** | 0.68 (0.49, 0.87)*** | 0.45 (0.19, 0.71)*** |
| South Asia |  |  |  |
| 20-29 | Reference | Reference | Reference |
| 30-39 | 0.36 (0.23, 0.49)*** | 0.023 (-0.048, 0.094) | -0.078 (-0.18, 0.02) |
| 40-49 | 0.59 (0.43, 0.76)*** | 0.079 (-0.0065, 0.16) | 0.077 (-0.04, 0.19) |
| 50-59 | 0.66 (0.45, 0.87)*** | 0.059 (-0.045, 0.16) | 0.18 (0.041, 0.32)* |
| 60-69 | 0.56 (0.32, 0.79)*** | 0.13 (0.014, 0.25)* | 0.21 (0.047, 0.37)* |
| 70-79 | 0.47 (0.23, 0.71)*** | 0.26 (0.14, 0.38)*** | 0.4 (0.23, 0.57)*** |
| Sub-Saharan Africa |  |  |  |
| 20-29 | Reference | Reference | Reference |
| 30-39 | 0.41 (0.3, 0.53)*** | 0.21 (0.15, 0.27)*** | 0.18 (0.097, 0.26)*** |
| 40-49 | 0.67 (0.52, 0.82)*** | 0.25 (0.17, 0.33)*** | 0.32 (0.21, 0.42)*** |
| 50-59 | 0.82 (0.62, 1)*** | 0.28 (0.19, 0.38)*** | 0.4 (0.27, 0.53)*** |
| 60-69 | 0.8 (0.58, 1)*** | 0.23 (0.12, 0.34)*** | 0.4 (0.25, 0.55)*** |
| 70-79 | 0.75 (0.53, 0.96)*** | 0.32 (0.21, 0.43)*** | 0.42 (0.27, 0.57)*** |
| Age group (years) × mean BMI (per one more unit kg/m^2^) |  |  |  |
| 20-29 | Reference | Reference | Reference |
| 30-39 | 0.00087 (-0.014, 0.016) | -0.043 (-0.051, -0.035)*** | -0.031 (-0.042, -0.02)*** |
| 40-49 | -0.00078 (-0.015, 0.013) | -0.058 (-0.065, -0.05)*** | -0.043 (-0.053, -0.033)*** |
| 50-59 | 0.0012 (-0.014, 0.016) | -0.064 (-0.072, -0.057)*** | -0.052 (-0.062, -0.041)*** |
| 60-69 | -0.0088 (-0.025, 0.0071) | -0.061 (-0.069, -0.052)*** | -0.057 (-0.069, -0.045)*** |
| 70-79 | -0.015 (-0.033, 0.0038) | -0.06 (-0.07, -0.05)*** | -0.054 (-0.068, -0.04)*** |
| Age group (years) × year (per one more recent year since 1985) |  |  |  |
| 20-29 | Reference | Reference | Reference |
| 30-39 | -0.00032 (-0.0031, 0.0024) | -0.0016 (-0.0031, -0.00016)* | -0.000072 (-0.0022, 0.002) |
| 40-49 | -0.0026 (-0.0053, 0.000063) | -0.00088 (-0.0023, 0.00055) | -0.000028 (-0.0021, 0.002) |
| 50-59 | -0.0053 (-0.0081, -0.0025)*** | -0.0024 (-0.0039, -0.00098)** | -0.00024 (-0.0023, 0.0018) |
| 60-69 | -0.0074 (-0.011, -0.0043)*** | -0.0041 (-0.0057, -0.0024)*** | -0.0015 (-0.0039, 0.0008) |
| 70-79 | -0.0054 (-0.009, -0.0019)** | -0.0062 (-0.0081, -0.0043)*** | -0.0024 (-0.0051, 0.00026) |
| Region × year (per one more recent year since 1985) |  |  |  |
| Central and Eastern Europe | Reference | Reference | Reference |
| Central Asia, the Middle East and North Africa | 0.0012 (-0.0028, 0.0053) | -0.00044 (-0.0025, 0.0016) | -0.0024 (-0.0051, 0.00039) |
| East and Southeast Asia | 0.007 (0.0033, 0.011)*** | 0.0033 (0.0014, 0.0052)*** | 0.0012 (-0.0017, 0.004) |
| High-income Asia Pacific | 0.0035 (-0.00025, 0.0073) | 0.0066 (0.0047, 0.0086)*** | 0.0051 (0.0023, 0.0078)*** |
| High-income western | 0.0026 (-0.00064, 0.0058) | 0.0014 (-0.00016, 0.003) | 0.0011 (-0.0011, 0.0032) |
| Latin America and the Caribbean | 0.0044 (0.00042, 0.0083)* | 0.002 (-0.000032, 0.004) | 0.00086 (-0.0018, 0.0036) |
| Oceania | -0.00084 (-0.011, 0.0095) | -0.0033 (-0.0062, -0.00034)* | -0.0028 (-0.0068, 0.0011) |
| South Asia | -0.0002 (-0.0047, 0.0042) | -0.00026 (-0.0026, 0.0021) | -0.0074 (-0.011, -0.004)*** |
| Sub-Saharan Africa | 0.002 (-0.0018, 0.0058) | 0.0045 (0.0026, 0.0065)*** | 0.0047 (0.002, 0.0073)*** |

*p ≤ 0.05; **p ≤ 0.01; ***p ≤ 0.001

^#^ Coefficients are relative to the reference group 20-29, for the variable Age, and Central and Eastern Europe, for the variable Region.

The R^2^ of the regression for women was 0.83 for underweight, 0.96 for obesity and 0.91 for severe obesity.
