# Supplementary material for: Heterogeneous contributions of change in population distribution of body mass index to change in obesity and underweight
Source: eLife. 2021 Mar 9;10:e60060. doi: 10.7554/eLife.60060 (PMC7943191; doi:10.7554/eLife.60060)
Supplement: Supplementary file 2. [file elife-60060-supp2.docx]

**Supplementary file 2.** Coefficients of the regression of probit-transformed prevalence of underweight, obesity and severe obesity in men on mean body mass index.

| **Variable** | **Coefficient for underweight** | **Coefficient for obesity** | **Coefficient for severe obesity** |
| --- | --- | --- | --- |
| Intercept^#^ | -2.2 (-2.3, -2.1)*** | -1.2 (-1.2, -1.1)*** | -1.9 (-2, -1.8)*** |
| Mean BMI (per one more unit kg/m^2^)^#^ | -0.12 (-0.16, -0.076)*** | 0.26 (0.24, 0.28)*** | 0.21 (0.18, 0.24)*** |
| Age group (years)^#^ |  |  |  |
| 20-29 | Reference | Reference | Reference |
| 30-39 | -0.1 (-0.22, 0.022) | 0.013 (-0.05, 0.077) | -0.054 (-0.16, 0.049) |
| 40-49 | -0.098 (-0.24, 0.046) | 0.061 (-0.01, 0.13) | -0.018 (-0.13, 0.096) |
| 50-59 | 0.052 (-0.11, 0.21) | 0.1 (0.023, 0.18)* | 0.018 (-0.11, 0.14) |
| 60-69 | 0.14 (-0.017, 0.3) | 0.13 (0.053, 0.21)** | 0.022 (-0.1, 0.15) |
| 70-79 | 0.24 (0.093, 0.39)** | 0.046 (-0.03, 0.12) | -0.078 (-0.2, 0.042) |
| Year (per one more recent year since 1985)^#^ | 0.0095 (0.0045, 0.014)*** | 0.0043 (0.0019, 0.0068)*** | 0.0067 (0.0028, 0.011)*** |
| Region^#^ |  |  |  |
| Central and Eastern Europe | Reference | Reference | Reference |
| Central Asia, the Middle East and North Africa | 0.3 (0.19, 0.41)*** | 0.16 (0.099, 0.23)*** | 0.22 (0.12, 0.33)*** |
| East and Southeast Asia | 0.14 (0.011, 0.27)* | 0.25 (0.17, 0.32)*** | 0.31 (0.18, 0.44)*** |
| High-income Asia Pacific | 0.25 (0.056, 0.45)* | 0.11 (-0.027, 0.25) | -0.15 (-0.4, 0.094) |
| High-income western | 0.14 (0.039, 0.24)** | 0.12 (0.059, 0.17)*** | 0.27 (0.17, 0.37)*** |
| Latin America and the Caribbean | 0.11 (0.00037, 0.22)* | 0.1 (0.041, 0.17)** | 0.15 (0.048, 0.26)** |
| Oceania | 0.12 (-0.073, 0.31) | 0.14 (0.047, 0.23)** | 0.18 (0.033, 0.32)* |
| South Asia | 0.34 (0.19, 0.49)*** | 0.23 (0.13, 0.32)*** | 0.35 (0.19, 0.5)*** |
| Sub-Saharan Africa | 0.3 (0.16, 0.44)*** | 0.13 (0.049, 0.21)** | 0.35 (0.21, 0.48)*** |
| Region × mean BMI (per one more unit kg/m^2^) |  |  |  |
| Central and Eastern Europe | Reference | Reference | Reference |
| Central Asia, the Middle East and North Africa | -0.03 (-0.069, 0.0099) | -0.012 (-0.03, 0.0066) | 0.033 (0.0055, 0.061)* |
| East and Southeast Asia | -0.13 (-0.17, -0.093)*** | 0.028 (0.009, 0.046)** | 0.017 (-0.014, 0.047) |
| High-income Asia Pacific | -0.066 (-0.13, 0.00039) | -0.028 (-0.068, 0.013) | -0.08 (-0.15, -0.013)* |
| High-income western | 0.087 (0.047, 0.13)*** | 0.032 (0.014, 0.051)*** | 0.083 (0.055, 0.11)*** |
| Latin America and the Caribbean | -0.053 (-0.093, -0.013)** | -0.027 (-0.046, -0.0087)** | -0.013 (-0.042, 0.016) |
| Oceania | 0.069 (0.027, 0.11)** | -0.046 (-0.065, -0.028)*** | -0.012 (-0.04, 0.016) |
| South Asia | -0.13 (-0.17, -0.089)*** | 0.0095 (-0.01, 0.029) | 0.011 (-0.02, 0.043) |
| Sub-Saharan Africa | -0.029 (-0.069, 0.01) | 0.02 (0.00087, 0.039)* | 0.031 (0.0021, 0.06)* |
| Region × age group (years) |  |  |  |
| Central and Eastern Europe | Reference | Reference | Reference |
| Central Asia, the Middle East and North Africa |  |  |  |
| 20-29 | Reference | Reference | Reference |
| 30-39 | -0.028 (-0.17, 0.11) | -0.073 (-0.15, 0.003) | -0.14 (-0.25, -0.018)* |
| 40-49 | 0.0013 (-0.16, 0.16) | -0.1 (-0.18, -0.02)* | -0.28 (-0.4, -0.15)*** |
| 50-59 | -0.052 (-0.23, 0.12) | -0.12 (-0.21, -0.034)** | -0.26 (-0.39, -0.12)*** |
| 60-69 | -0.02 (-0.2, 0.16) | -0.14 (-0.23, -0.048)** | -0.3 (-0.44, -0.16)*** |
| 70-79 | -0.22 (-0.39, -0.042)* | -0.099 (-0.19, -0.0093)* | -0.13 (-0.27, 0.0092) |
| East and Southeast Asia |  |  |  |
| 20-29 | Reference | Reference | Reference |
| 30-39 | -0.0069 (-0.16, 0.15) | -0.25 (-0.33, -0.16)*** | -0.23 (-0.36, -0.089)** |
| 40-49 | -0.035 (-0.21, 0.14) | -0.37 (-0.46, -0.28)*** | -0.44 (-0.59, -0.3)*** |
| 50-59 | -0.14 (-0.33, 0.04) | -0.38 (-0.48, -0.28)*** | -0.51 (-0.66, -0.36)*** |
| 60-69 | -0.14 (-0.33, 0.05) | -0.39 (-0.49, -0.29)*** | -0.54 (-0.7, -0.38)*** |
| 70-79 | -0.27 (-0.47, -0.082)** | -0.21 (-0.32, -0.11)*** | -0.37 (-0.54, -0.2)*** |
| High-income Asia Pacific |  |  |  |
| 20-29 | Reference | Reference | Reference |
| 30-39 | -0.075 (-0.24, 0.091) | -0.23 (-0.32, -0.13)*** | -0.16 (-0.31, -0.013)* |
| 40-49 | -0.19 (-0.38, -0.0074)* | -0.45 (-0.55, -0.35)*** | -0.41 (-0.57, -0.25)*** |
| 50-59 | -0.34 (-0.53, -0.15)*** | -0.63 (-0.73, -0.52)*** | -0.59 (-0.75, -0.42)*** |
| 60-69 | -0.32 (-0.51, -0.13)** | -0.7 (-0.81, -0.6)*** | -0.65 (-0.82, -0.48)*** |
| 70-79 | -0.32 (-0.5, -0.13)*** | -0.63 (-0.73, -0.52)*** | -0.57 (-0.75, -0.39)*** |
| High-income western |  |  |  |
| 20-29 | Reference | Reference | Reference |
| 30-39 | -0.28 (-0.41, -0.14)*** | -0.15 (-0.22, -0.085)*** | -0.25 (-0.36, -0.14)*** |
| 40-49 | -0.36 (-0.52, -0.2)*** | -0.22 (-0.29, -0.14)*** | -0.37 (-0.49, -0.25)*** |
| 50-59 | -0.47 (-0.64, -0.3)*** | -0.26 (-0.34, -0.18)*** | -0.46 (-0.59, -0.33)*** |
| 60-69 | -0.49 (-0.66, -0.32)*** | -0.27 (-0.36, -0.19)*** | -0.52 (-0.65, -0.39)*** |
| 70-79 | -0.48 (-0.65, -0.32)*** | -0.19 (-0.27, -0.11)*** | -0.46 (-0.58, -0.33)*** |
| Latin America and the Caribbean |  |  |  |
| 20-29 | Reference | Reference | Reference |
| 30-39 | 0.068 (-0.077, 0.21) | -0.062 (-0.14, 0.014) | -0.054 (-0.17, 0.064) |
| 40-49 | 0.18 (0.019, 0.35)* | -0.063 (-0.15, 0.02) | -0.1 (-0.23, 0.027) |
| 50-59 | 0.091 (-0.084, 0.27) | -0.087 (-0.18, 0.0017) | -0.18 (-0.32, -0.044)** |
| 60-69 | 0.074 (-0.1, 0.25) | -0.1 (-0.19, -0.013)* | -0.16 (-0.3, -0.022)* |
| 70-79 | -0.00097 (-0.17, 0.17) | -0.032 (-0.12, 0.055) | -0.12 (-0.25, 0.02) |
| Oceania |  |  |  |
| 20-29 | Reference | Reference | Reference |
| 30-39 | 0.021 (-0.21, 0.25) | -0.0016 (-0.11, 0.11) | 0.042 (-0.12, 0.21) |
| 40-49 | 0.17 (-0.069, 0.41) | 0.049 (-0.064, 0.16) | 0.081 (-0.092, 0.25) |
| 50-59 | 0.099 (-0.14, 0.34) | 0.058 (-0.057, 0.17) | 0.11 (-0.072, 0.28) |
| 60-69 | 0.33 (0.034, 0.63)* | 0.068 (-0.074, 0.21) | 0.061 (-0.15, 0.27) |
| 70-79 | 0.25 (-0.17, 0.66) | 0.29 (0.089, 0.49)** | -0.21 (-0.55, 0.14) |
| South Asia |  |  |  |
| 20-29 | Reference | Reference | Reference |
| 30-39 | 0.15 (-0.018, 0.32) | -0.15 (-0.24, -0.055)** | -0.21 (-0.36, -0.053)** |
| 40-49 | 0.17 (-0.018, 0.36) | -0.2 (-0.3, -0.1)*** | -0.24 (-0.4, -0.084)** |
| 50-59 | 0.032 (-0.17, 0.23) | -0.19 (-0.3, -0.09)*** | -0.29 (-0.46, -0.12)*** |
| 60-69 | -0.05 (-0.26, 0.16) | -0.16 (-0.27, -0.046)** | -0.15 (-0.33, 0.038) |
| 70-79 | -0.21 (-0.43, 0.017) | -0.049 (-0.18, 0.079) | 0.047 (-0.17, 0.27) |
| Sub-Saharan Africa |  |  |  |
| 20-29 | Reference | Reference | Reference |
| 30-39 | 0.18 (0.017, 0.34)* | 0.05 (-0.038, 0.14) | -0.01 (-0.15, 0.13) |
| 40-49 | 0.27 (0.097, 0.45)** | 0.032 (-0.062, 0.13) | -0.1 (-0.25, 0.041) |
| 50-59 | 0.21 (0.016, 0.39)* | 0.052 (-0.048, 0.15) | -0.11 (-0.26, 0.047) |
| 60-69 | 0.26 (0.058, 0.46)* | -0.0056 (-0.11, 0.1) | 0.012 (-0.16, 0.18) |
| 70-79 | 0.17 (-0.041, 0.37) | 0.16 (0.049, 0.28)** | 0.16 (-0.024, 0.34) |
| Age group (years) × mean BMI (per one more unit kg/m^2^) |  |  |  |
| 20-29 | Reference | Reference | Reference |
| 30-39 | 0.00034 (-0.022, 0.023) | -0.018 (-0.03, -0.0059)** | -0.014 (-0.032, 0.005) |
| 40-49 | -0.014 (-0.036, 0.0084) | -0.03 (-0.042, -0.018)*** | -0.025 (-0.043, -0.007)** |
| 50-59 | -0.022 (-0.044, -0.00065)* | -0.029 (-0.04, -0.017)*** | -0.039 (-0.057, -0.02)*** |
| 60-69 | -0.025 (-0.048, -0.0014)* | -0.034 (-0.047, -0.021)*** | -0.024 (-0.045, -0.0039)* |
| 70-79 | -0.047 (-0.074, -0.021)*** | -0.024 (-0.039, -0.0094)** | -0.036 (-0.061, -0.012)** |
| Age group (years) × year (per one more recent year since 1985) |  |  |  |
| 20-29 | Reference | Reference | Reference |
| 30-39 | -0.0031 (-0.0066, 0.00048) | 0.0000041 (-0.0019, 0.0019) | 0.00012 (-0.0031, 0.0034) |
| 40-49 | -0.005 (-0.0085, -0.0015)** | -0.0012 (-0.0031, 0.00064) | -0.0013 (-0.0045, 0.0018) |
| 50-59 | -0.0066 (-0.01, -0.0032)*** | -0.0026 (-0.0044, -0.00071)** | -0.001 (-0.0042, 0.0021) |
| 60-69 | -0.01 (-0.014, -0.0064)*** | -0.0038 (-0.0059, -0.0016)*** | -0.0032 (-0.0067, 0.00026) |
| 70-79 | -0.0095 (-0.014, -0.0052)*** | -0.0045 (-0.0069, -0.0021)*** | -0.0013 (-0.0054, 0.0029) |
| Region × year (per one more recent year since 1985) |  |  |  |
| Central and Eastern Europe | Reference | Reference | Reference |
| Central Asia, the Middle East and North Africa | 0.0011 (-0.0044, 0.0066) | -0.0017 (-0.0045, 0.001) | -0.00077 (-0.0049, 0.0034) |
| East and Southeast Asia | 0.0057 (0.00072, 0.011)* | 0.0042 (0.0016, 0.0067)** | -0.0022 (-0.0068, 0.0023) |
| High-income Asia Pacific | -0.0046 (-0.01, 0.0013) | 0.0068 (0.0036, 0.0099)*** | 0.0068 (0.0015, 0.012)* |
| High-income western | -0.0067 (-0.011, -0.002)** | -0.00094 (-0.003, 0.0012) | 0.0002 (-0.003, 0.0034) |
| Latin America and the Caribbean | -0.0032 (-0.0087, 0.0023) | 0.00031 (-0.0023, 0.0029) | -0.00036 (-0.0044, 0.0037) |
| Oceania | -0.011 (-0.021, -0.00058)* | -0.0055 (-0.0091, -0.002)** | -0.0021 (-0.0073, 0.0031) |
| South Asia | -0.0072 (-0.013, -0.0015)* | -0.0023 (-0.0053, 0.00075) | -0.013 (-0.018, -0.0076)*** |
| Sub-Saharan Africa | 0.0013 (-0.0043, 0.0069) | 0.00059 (-0.0024, 0.0036) | 0.00096 (-0.0039, 0.0058) |

*p ≤ 0.05; **p ≤ 0.01; ***p ≤ 0.001

^#^ Coefficients are relative to the reference group 20-29, for the variable Age, and Central and Eastern Europe, for the variable Region.

The R^2^ of the regression for men was 0.84 for underweight, 0.94 for obesity and 0.83 for severe obesity.
