# Supplementary material for: Heterogeneous contributions of change in population distribution of body mass index to change in obesity and underweight
Source: eLife. 2021 Mar 9;10:e60060. doi: 10.7554/eLife.60060 (PMC7943191; doi:10.7554/eLife.60060)
Supplement: Supplementary file 3. [file elife-60060-supp3.docx]

**Supplementary file 3.** List of analysis regions and countries in each region.

| **Region**  **(Number of countries)** | **Countries** |
| --- | --- |
| **Central and Eastern Europe (20)** | Albania, Belarus, Bosnia and Herzegovina, Bulgaria, Croatia, Czech Republic, Estonia, Hungary, Latvia, Lithuania, Macedonia (TFYR), Moldova, Montenegro, Poland, Romania, Russian Federation, Serbia, Slovakia, Slovenia, Ukraine |
| **Central Asia, the Middle East and North Africa (28)** | Algeria, Armenia, Azerbaijan, Bahrain, Egypt, Georgia, Iran, Iraq, Jordan, Kazakhstan, Kuwait, Kyrgyzstan, Lebanon, Libya, Mongolia, Morocco, Occupied Palestinian Territory, Oman, Qatar, Saudi Arabia, Syrian Arab Republic, Tajikistan, Tunisia, Turkey, Turkmenistan, United Arab Emirates, Uzbekistan, Yemen |
| **East and Southeast Asia (16)** | Brunei Darussalam, Cambodia, China, China (Hong Kong SAR), Indonesia, Lao PDR, Malaysia, Maldives, Myanmar, North Korea, Philippines, Sri Lanka, Taiwan, Thailand, Timor-Leste, Viet Nam |
| **High-income Asia Pacific (3)** | Japan, Singapore, South Korea |
| **High-income western (27)** | Andorra, Australia, Austria, Belgium, Canada, Cyprus, Denmark, Finland, France, Germany, Greece, Greenland, Iceland, Ireland, Israel, Italy, Luxembourg, Malta, Netherlands, New Zealand, Norway, Portugal, Spain, Sweden, Switzerland, United Kingdom, United States of America |
| **Latin America and the Caribbean (35)** | Antigua and Barbuda, Argentina, Bahamas, Barbados, Belize, Bermuda, Bolivia, Brazil, Chile, Colombia, Costa Rica, Cuba, Dominica, Dominican Republic, Ecuador, El Salvador, Grenada, Guatemala, Guyana, Haiti, Honduras, Jamaica, Mexico, Nicaragua, Panama, Paraguay, Peru, Puerto Rico, Saint Kitts and Nevis, Saint Lucia, Saint Vincent and the Grenadines, Suriname, Trinidad and Tobago, Uruguay, Venezuela |
| **Oceania (17)** | American Samoa, Cook Islands, Fiji, French Polynesia, Kiribati, Marshall Islands, Micronesia (Federated States of), Nauru, Niue, Palau, Papua New Guinea, Samoa, Solomon Islands, Tokelau, Tonga, Tuvalu, Vanuatu |
| **South Asia (6)** | Afghanistan, Bangladesh, Bhutan, India, Nepal, Pakistan |
| **Sub-Saharan Africa (48)** | Angola, Benin, Botswana, Burkina Faso, Burundi, Cabo Verde, Cameroon, Central African Republic, Chad, Comoros, Congo, Cote d'Ivoire, Djibouti, DR Congo, Equatorial Guinea, Eritrea, Ethiopia, Gabon, Gambia, Ghana, Guinea, Guinea Bissau, Kenya, Lesotho, Liberia, Madagascar, Malawi, Mali, Mauritania, Mauritius, Mozambique, Namibia, Niger, Nigeria, Rwanda, Sao Tome and Principe, Senegal, Seychelles, Sierra Leone, Somalia, South Africa, Sudan, Swaziland, Tanzania, Togo, Uganda, Zambia, Zimbabwe |
