# Supplementary material for: Heterogeneous contributions of change in population distribution of body mass index to change in obesity and underweight
Source: eLife. 2021 Mar 9;10:e60060. doi: 10.7554/eLife.60060 (PMC7943191; doi:10.7554/eLife.60060)
Supplement: Supplementary file 4. [file elife-60060-supp4.docx]

**Supplementary file 4.** List of data sources and their characteristics.

|  | **Country** | **Study years** | **Survey/Study name/Citation** | **Level of representativeness** | **Rural, Urban or Both** | **Age range as in NCD-RisC database** | | **Sample size (BMI)** | |
| --- | --- | --- | --- | --- | --- | --- | --- | --- | --- |
|  |  |  |  |  |  | **Male** | **Female** | **Male** | **Female** |
| 1 | Afghanistan | 2013 | National Nutrition Survey | National | Both |  | 10-49 |  | 18,433 |
| 2 | Afghanistan | 2018 | STEPS | National | Both | 18-69 | 18-69 | 1,984 | 1,703 |
| 3 | Albania | 2001 | Shapo et al., Public Health Nutr 6:471-77, 2003 | Community | Urban | 24+ | 24+ | 535 | 585 |
| 4 | Albania | 2008-2009 | DHS | National | Both | 15-49 | 15-49 | 2,978 | 7,386 |
| 5 | Albania | 2017-2018 | DHS | National | Both | 15-59 | 15-59 | 5,953 | 14,447 |
| 6 | Algeria | 2003 | STEPS | Subnational | Both | 25-64 | 25-64 | 1,612 | 2,437 |
| 7 | Algeria | 2005 | Transition and Health Impact in North Africa | National | Both | 35-70 | 35-70 | 2,004 | 2,741 |
| 8 | Algeria | 2007-2009 | The ISOR (InSulino-resistance in ORan) study | Community | Urban | 30-64 | 30-64 | 378 | 409 |
| 9 | Algeria | 2016-2017 | STEPS | National | Both | 18-69 | 18-69 | 2,991 | 3,636 |
| 10 | American Samoa | 1990 | McGarvey, Pac Health Dialog 8(1):157-62, 2001 | National | Both | 25+ | 25+ | 359 | 484 |
| 11 | American Samoa | 1992 | McGarvey, Pac Health Dialog 8(1):157-62, 2001 | National | Both | 27+ | 27+ | 232 | 337 |
| 12 | American Samoa | 1994 | McGarvey, Pac Health Dialog 8(1):157-62, 2001 | National | Both | 29+ | 29+ | 165 | 245 |
| 13 | American Samoa | 2004 | STEPS | National | Both | 25-64 | 25-64 | 949 | 1,060 |
| 14 | Argentina | 1985-1986 | INTERSALT | Community | Urban | 20-59 | 20-59 | 100 | 100 |
| 15 | Argentina | 1995-1998 | de Sereday et al., Diabetes Metab 30:335-9, 2004 | Subnational | Urban | 15-74 | 15-74 | 924 | 1,246 |
| 16 | Argentina | 2003 | CEDES-Programa VIGI+A-Banco Mundial, 2004 | Community | Urban | 15-74 | 15-74 | 151 | 176 |
| 17 | Argentina | 2005 | Encuensta Nacional de Nutrición y Salud 2005 | National | Both |  | 10-49 |  | 6,581 |
| 18 | Argentina | 2004-2005 | CArdiovascular Risk factors Multiple Evaluation in Latin America | Community | Urban | 25-64 | 25-64 | 733 | 742 |
| 19 | Argentina | 2006 | Virasoro Survey | Community | Urban | 15-84 | 15-84 | 261 | 306 |
| 20 | Argentina | 2008-2011 | The VELA Project | Community | Rural | 5+ | 5+ | 380 | 543 |
| 21 | Argentina | 2011 | Primera Encuesta Alimentaria y Nutricional de la Ciudad Autónoma de Buenos Aires - EAN CABA | Community | Urban | 5-18; 60+ | 5-49; 60+ | 1,173 | 2,229 |
| 22 | Argentina | 2011-2012 | CESCAS Study | Community | Urban | 30-79 | 30-79 | 1,584 | 2,378 |
| 23 | Argentina | 2012-2013 | Primer estudio sobre el estado nutricional y los hábitos alimentarios de la población adulta de Rosario | Community | Urban | 18-70 | 18-70 | 371 | 823 |
| 24 | Argentina | 2018 | Encuesta Nacional de Factores de Riesgo 2018 | National | Both | 18+ | 18+ | 6,960 | 9,449 |
| 25 | Armenia | 1998 | The health and nutritional status of children and women in Armenia | National | Both |  | 18-45 |  | 2,420 |
| 26 | Armenia | 2000 | DHS | National | Both |  | 15-49 |  | 5,982 |
| 27 | Armenia | 2005 | DHS | National | Both | 15-49 | 15-49 | 1,220 | 6,232 |
| 28 | Armenia | 2015-2016 | DHS | National | Both |  | 15-49 |  | 5,731 |
| 29 | Armenia | 2016 | STEPS | National | Both | 18-69 | 18-69 | 604 | 1,447 |
| 30 | Australia | 1988-1989 | Dubbo Study of Australian Elderly | Community | Urban | 59+ | 59+ | 877 | 1,219 |
| 31 | Australia | 1989 | Risk Factor Prevalence Study | National | Urban | 20-69 | 20-69 | 4,497 | 4,678 |
| 32 | Australia | 1988-1989 | MONICA, Newcastle | Subnational | Urban | 35-64 | 35-64 | 672 | 671 |
| 33 | Australia | 1988-1989 | MONICA, Newcastle | Community | Urban | 25-34 | 25-34 | 70 | 84 |
| 34 | Australia | 1992-1993 | Australia Longitudinal Study of Ageing | Community | Urban | 65+ | 65+ | 814 | 746 |
| 35 | Australia | 1994 | MONICA, Newcastle | Subnational | Urban | 35-64 | 35-64 | 637 | 688 |
| 36 | Australia | 1994 | MONICA, Perth inner | Community | Urban | 25-64 | 25-64 | 363 | 349 |
| 37 | Australia | 1994 | MONICA, Perth outer | Community | Urban | 25-64 | 25-64 | 373 | 387 |
| 38 | Australia | 1995 | National Nutrition Survey 1995 | National | Both | 5+ | 5+ | 5,983 | 6,390 |
| 39 | Australia | 1996-1998 | Western Australian AAA Screening Program | Community | Urban | 65-84 |  | 12,194 |  |
| 40 | Australia | 1999-2000 | The Australian Diabetes, Obesity and Lifestyle Study 1999-2000 | National | Both | 25+ | 25+ | 4,991 | 6,070 |
| 41 | Australia | 2000 | Perth children | Community | Both | 25 | 25 | 266 | 334 |
| 42 | Australia | 1999-2003 | North West Adelaide Health Study | Community | Urban | 18+ | 18+ | 1,932 | 2,122 |
| 43 | Australia | 2004-2005 | The Australian Diabetes, Obesity and Lifestyle Study 2004-2005 | National | Both | 30+ | 30+ | 2,874 | 3,472 |
| 44 | Australia | 2004-2006 | North West Adelaide Health Study | Community | Urban | 20+ | 20+ | 1,523 | 1,679 |
| 45 | Australia | 2004-2005 | Janus et al., Med J Aust 187:147-52, 2007 | Community | Rural | 25-74 | 25-74 | 383 | 423 |
| 46 | Australia | 2007-2008 | National Health Survey | National | Both | 18+ | 18+ | 5,279 | 5,655 |
| 47 | Australia | 2008-2010 | North West Adelaide Health Study | Community | Urban | 24+ | 24+ | 1,168 | 1,318 |
| 48 | Australia | 2012 | The Australian Diabetes, Obesity and Lifestyle Study 2012 | National | Both | 37+ | 37+ | 2,048 | 2,530 |
| 49 | Australia | 2011-2013 | Australian Health Survey 2011-13 | National | Both | 5+ | 5+ | 12,190 | 13,011 |
| 50 | Australia | 2017-2018 | National Health Survey | National | Both | 18+ | 18+ | 7,576 | 8,729 |
| 51 | Austria | 1986 | CINDI | Community | Both | 25-64 | 25-64 | 657 | 715 |
| 52 | Austria | 1991 | CINDI survey Vorarlberg/Austria | Subnational | Both | 25-64 | 25-64 | 698 | 738 |
| 53 | Austria | 1992 | Vorarlberg Health Monitoring and Promotion Programme | Subnational | Both | 18+ | 18+ | 14,161 | 18,835 |
| 54 | Austria | 1998 | Vorarlberg Health Monitoring and Promotion Programme | Subnational | Both | 18+ | 18+ | 16,153 | 20,915 |
| 55 | Austria | 1998-1999 | CINDI survey Vorarlberg/Austria | Subnational | Both | 25-64 | 25-64 | 409 | 414 |
| 56 | Austria | 2004 | Vorarlberg Health Monitoring and Promotion Programme | Subnational | Both | 18+ | 18+ | 20,160 | 23,893 |
| 57 | Austria | 2010-2012 | Austrian Study on Nutritional Status 2012 | National | Both | 6-80 | 6-80 | 363 | 446 |
| 58 | Azerbaijan | 1996 | Health and Nutrition Survey | National | Both | 19-59 | 19-59 | 121 | 295 |
| 59 | Azerbaijan | 2001 | Reproductive Health Survey (RHS) | National | Both |  | 15-44 |  | 1,726 |
| 60 | Azerbaijan | 2006 | DHS | National | Both | 15-59 | 15-49 | 2,493 | 7,868 |
| 61 | Azerbaijan | 2017 | STEPS | National | Both | 18-69 | 18-69 | 1,117 | 1,577 |
| 62 | Bahamas | 2011-2012 | STEPS | National | Both | 25-64 | 25-64 | 586 | 938 |
| 63 | Bahrain | 1991-1992 | al-Mannai et al., J R Soc Health 116:30-2, 7-40, 1996 | Community | Both | 20+ | 20+ | 137 | 153 |
| 64 | Bahrain | 1995 | Musaiger et al., Ann Hum Biol 28:346-50, 2001 | Community | Both | 30+ | 30+ | 298 | 216 |
| 65 | Bahrain | 1998-1999 | National Nutrition Survey | National | Both | 19+ | 19+ | 1,120 | 1,181 |

| 66 | Bahrain | 2007 | STEPS | National | Both | 20-64 | 20-64 | 854 | 858 |
| --- | --- | --- | --- | --- | --- | --- | --- | --- | --- |
| 67 | Bangladesh | 1992 | Rahman et al., Hypertension 33:74-8, 1999 | Community | Rural | 30+ | 30+ | 965 | 643 |
| 68 | Bangladesh | 1996-1997 | DHS | National | Both |  | 20-49 |  | 3,384 |
| 69 | Bangladesh | 1998 | Zaman et al., J Health Popul Nutr 21:162-63, 2003 | Community | Rural | 20+ | 20+ | 290 | 379 |
| 70 | Bangladesh | 1999-2000 | DHS | National | Both |  | 20-49 |  | 3,887 |
| 71 | Bangladesh | 1999-2000 | Hussain et al., Eur J Public Health, 17:291-96, 2007 | Community | Rural | 20-59 | 20-59 | 2,037 | 2,720 |
| 72 | Bangladesh | 2002 | STEPS | National | Rural | 25-64 | 25-64 | 2,086 | 2,038 |
| 73 | Bangladesh | 2002 | STEPS | National | Urban | 25-64 | 25-64 | 3,533 | 3,737 |
| 74 | Bangladesh | 2000-2004 | Nutritional Surveillance Project | National | Rural |  | 15-45 |  | 224,251 |
| 75 | Bangladesh | 2004 | DHS | National | Both |  | 20-49 |  | 9,165 |
| 76 | Bangladesh | 2006 | Urban Health Survey | Subnational | Urban | 20-59 | 20-59 | 6,109 | 5,898 |
| 77 | Bangladesh | 2007 | DHS | National | Both |  | 20-49 |  | 9,037 |
| 78 | Bangladesh | 2009-2010 | STEPS | National | Both | 25+ | 25+ | 4,310 | 4,849 |
| 79 | Bangladesh | 2011 | DHS | National | Both | 15+ | 20+ | 5,254 | 16,679 |
| 80 | Bangladesh | 2013 | STEPS | National | Both | 25+ | 25+ | 1,812 | 2,261 |
| 81 | Bangladesh | 2014 | DHS | National | Both |  | 20-49 |  | 14,963 |
| 82 | Bangladesh | 2015 | An Assessment of BRAC Health Nutrition and Population Programme and Benchmark Survey of Sustainable Development Goal – 2015 | National | Rural | 35+ | 11+ | 5,432 | 18,378 |
| 83 | Bangladesh | 2018 | STEPS | National | Both | 18-69 | 18-69 | 3,784 | 4,229 |
| 84 | Bangladesh | 2018-2019 | National Nutrition Surveillence | National | Both | 10+ | 10+ | 12,211 | 12,102 |
| 85 | Barbados | 1987-1992 | Barbados Eye Study | National | Both | 40-84 | 40-84 | 1,980 | 2,627 |
| 86 | Barbados | 1991-1994 | Cooper et al., Am J Public Health 87(2):160-68, 1997 | Community | Urban | 25-100 | 25-100 | 329 | 482 |
| 87 | Barbados | 1999-2000 | The Survey on Health, Well-Being, and Aging in Latin America and the Caribbean (SABE) | Community | Urban | 60+ | 60+ | 559 | 866 |
| 88 | Barbados | 1997-2002 | The Barbados Incidence Studies of Eye Diseases II | National | Both | 40-84 | 40-84 | 1,004 | 1,441 |
| 89 | Barbados | 2011-2013 | Health of the Nation (HotN) | National | Both | 25+ | 25+ | 455 | 703 |
| 90 | Belarus | 2016-2017 | STEPS | National | Both | 18-69 | 18-69 | 2,085 | 2,894 |
| 91 | Belgium | 1984-1985 | Belgian Interuniversity Research on Nutrition and Health | National | Both | 25-74 | 25-74 | 5,897 | 5,289 |
| 92 | Belgium | 1985-1987 | INTERSALT, Charleroi | Community | Urban | 20-59 | 20-59 | 82 | 75 |
| 93 | Belgium | 1985-1986 | INTERSALT, Ghent | Community | Urban | 20-59 | 20-59 | 100 | 100 |
| 94 | Belgium | 1985-1987 | MONICA, Charleroi | Community | Urban | 25-64 | 25-64 | 347 | 327 |
| 95 | Belgium | 1985-1987 | MONICA, Ghent | Community | Urban | 25-64 | 25-64 | 549 | 459 |
| 96 | Belgium | 1985-1990 | Flemish Study on Environment, Genes and Health Outcomes | Community | Rural | 20-90 | 20-90 | 656 | 692 |
| 97 | Belgium | 1987-1990 | MONICA, Charleroi | Community | Urban | 25-64 | 25-64 | 325 | 301 |
| 98 | Belgium | 1988-1990 | MONICA, Ghent | Community | Urban | 25-64 | 25-64 | 456 | 449 |
| 99 | Belgium | 1990-1993 | MONICA, Charleroi | Community | Urban | 25-64 | 25-64 | 337 | 332 |
| 100 | Belgium | 1990-1992 | MONICA, Ghent | Community | Urban | 25-64 | 25-64 | 507 | 475 |
| 101 | Belgium | 1991-1994 | Flemish Study on Environment, Genes and Health Outcomes | Community | Rural | 26-88 | 26-88 | 393 | 416 |
| 102 | Belgium | 1992-1995 | Flemish Study on Environment, Genes and Health Outcomes | Community | Rural | 27-89 | 27-89 | 298 | 312 |
| 103 | Belgium | 1994-1996 | BIRNH Elderly: Belgian Interuniversity Research on Nutrition and Health in the Elderly | National | Both | 65-89 | 65-89 | 1,142 | 953 |
| 104 | Belgium | 1996-1998 | Flemish Study on Environment, Genes and Health Outcomes | Community | Rural | 10-84 | 10-84 | 404 | 402 |
| 105 | Belgium | 1998 | Flemish Study on Environment, Genes and Health Outcomes | Community | Rural | 32-86 | 32-86 | 320 | 359 |
| 106 | Belgium | 1998-2000 | Flemish Study on Environment, Genes and Health Outcomes | Community | Rural | 10-80 | 10-80 | 220 | 217 |
| 107 | Belgium | 1999-2001 | Flemish Study on Environment, Genes and Health Outcomes | Community | Rural | 10-81 | 10-81 | 232 | 254 |
| 108 | Belgium | 2001 | Flemish Study on Environment, Genes and Health Outcomes | Community | Rural | 10-78 | 10-78 | 242 | 222 |
| 109 | Belgium | 2002-2003 | Flemish Study on Environment, Genes and Health Outcomes | Community | Rural | 10-81 | 10-81 | 174 | 197 |
| 110 | Belgium | 2003 | The European Male Ageing Study | Community | Both | 40+ |  | 433 |  |
| 111 | Belgium | 2002-2004 | SPAH | Subnational | Both | 18-75 | 18-75 | 2,595 | 2,308 |
| 112 | Belgium | 2002-2005 | Flemish Study on Environment, Genes and Health Outcomes | Community | Rural | 10-88 | 10-88 | 447 | 462 |
| 113 | Belgium | 2005-2008 | Flemish Study on Environment, Genes and Health Outcomes | Community | Rural | 10-89 | 10-89 | 462 | 471 |
| 114 | Belgium | 2008 | The European Male Ageing Study | Community | Both | 40+ |  | 383 |  |
| 115 | Belgium | 2009-2013 | Flemish Study on Environment, Genes and Health Outcomes | Community | Rural | 20-88 | 20-88 | 330 | 335 |
| 116 | Belgium | 2010-2015 | Flemish Study on Environment, Genes and Health Outcomes | Community | Rural | 15-87 | 15-87 | 388 | 410 |
| 117 | Belgium | 2014-2015 | Food Consumption Survey | National | Urban | 5-64 | 5-64 | 1,481 | 1,491 |
| 118 | Belgium | 2018-2019 | European Health Examination Survey | National | Both | 18+ | 18+ | 548 | 604 |
| 119 | Belize | 2004-2005 | CAMDI | National | Both | 20+ | 20+ | 599 | 1,018 |
| 120 | Benin | 1996 | DHS | National | Both |  | 20-49 |  | 2,137 |
| 121 | Benin | 2001 | DHS | National | Both |  | 15-49 |  | 5,449 |
| 122 | Benin | 2006 | DHS | National | Both |  | 15-49 |  | 14,891 |
| 123 | Benin | 2007 | STEPS | Community | Urban | 25-64 | 25-64 | 955 | 1,508 |
| 124 | Benin | 2008 | STEPS | National | Both | 25-64 | 25-64 | 3,430 | 3,365 |
| 125 | Benin | 2011-2012 | DHS | National | Both |  | 15-49 |  | 14,589 |
| 126 | Benin | 2015 | STEPS | National | Both | 18-69 | 18-69 | 2,304 | 2,543 |
| 127 | Benin | 2017-2018 | DHS | National | Both |  | 15-49 |  | 7,180 |
| 128 | Bhutan | 2007 | STEPS | Community | Urban | 25-74 | 25-74 | 1,125 | 1,322 |
| 129 | Bhutan | 2014 | STEPS | National | Both | 18-69 | 18-69 | 1,069 | 1,674 |
| 130 | Bolivia | 1994 | DHS | National | Both |  | 20-49 |  | 2,128 |
| 131 | Bolivia | 1998 | DHS | National | Both |  | 20-49 |  | 3,939 |
| 132 | Bolivia | 2003 | DHS | National | Both |  | 15-49 |  | 16,349 |
| 133 | Bolivia | 2008 | DHS | National | Both |  | 15-49 |  | 15,543 |

| 134 | Bosnia and Herzegovina | 2002 | Non-communicable disease risk factor survey, Federation of B&H | Subnational | Both | 25-64 | 25-64 | 1,118 | 1,613 |
| --- | --- | --- | --- | --- | --- | --- | --- | --- | --- |
| 135 | Bosnia and Herzegovina | 2012 | Non-communicable disease risk factor survey, Federation of B&H | Subnational | Rural | 18+ | 18+ | 1,191 | 1,274 |
| 136 | Bosnia and Herzegovina | 2012 | Non-communicable disease risk factor survey, Federation of B&H | Subnational | Urban | 18+ | 18+ | 591 | 697 |
| 137 | Botswana | 2007 | STEPS | National | Both | 25-64 | 25-64 | 1,243 | 2,577 |
| 138 | Botswana | 2014 | STEPS | National | Both | 15-69 | 15-69 | 1,298 | 2,602 |
| 139 | Brazil | 1989 | Pesquisa Nacional sobre Saude e Nutricao | National | Both | 5+ | 5+ | 26,642 | 27,504 |
| 140 | Brazil | 1990-1991 | Fornes et al., Rev Saude Publica 36:12-8, 2002 | Community | Urban | 20+ | 20+ | 432 | 613 |
| 141 | Brazil | 1991-1993 | EPIDOSO | Community | Urban | 65+ | 65+ | 269 | 473 |
| 142 | Brazil | 1992-1998 | Moraes et al., Int J Cardiol 90:205-11, 2003 | Community | Urban | 18+ | 18+ | 438 | 543 |
| 143 | Brazil | 1995 | Health and Nutrition Survey of Rio de Janeiro | Community | Urban | 60+ | 60+ | 248 | 385 |
| 144 | Brazil | 1996 | DHS | National | Both |  | 20-49 |  | 2,884 |
| 145 | Brazil | 1996-1997 | Pesquisa sobre Padrões de Vida (PPV) | Subnational | Both | 5+ | 5+ | 7,451 | 8,466 |
| 146 | Brazil | 1995-1996 | Cohort study from Porto Alegre | Community | Urban | 18+ | 18+ | 489 | 596 |
| 147 | Brazil | 1996-1997 | The Bambui Cohort Study of Ageing | Community | Urban | 18+ | 18+ | 931 | 1,335 |
| 148 | Brazil | 1997 | PPV | Subnational | Both | 20+ | 20+ | 8,063 | 9,121 |
| 149 | Brazil | 1999-2000 | Prevalence of Risk Factors for Coronary Artery Disease in the State of Rio Grande do Sul | Subnational | Urban | 20+ | 20+ | 504 | 548 |
| 150 | Brazil | 1999-2000 | The Survey on Health, Well-Being, and Aging in Latin America and the Caribbean (SABE) | Community | Urban | 60+ | 60+ | 732 | 1,064 |
| 151 | Brazil | 1999-2000 | Pelotas cross-sectional survey | Community | Urban | 20-69 | 20-69 | 839 | 1,096 |
| 152 | Brazil | 2001 | de Freitas et al., Arq Bras Cardiol 88:191-99 | Community | Urban | 15+ | 15+ | 310 | 331 |
| 153 | Brazil | 2001-2003 | Bustos et al., Nutr Metab Cardiovasc Dis 17:581-89, 2007 | Community | Both | 22-28 | 22-28 | 992 | 1,064 |
| 154 | Brazil | 2002-2003 | Pesquisa de Orcamentos Familiares | National | Both | 5+ | 5+ | 81,152 | 80,163 |
| 155 | Brazil | 2003 | PNAFS | Community | Urban | 20+ | 20+ | 1,155 | 1,941 |
| 156 | Brazil | 2003 | Women Health in Southern Brazil | Community | Urban |  | 20-60 |  | 986 |
| 157 | Brazil | 2004 | Caju & Virgen das Gracas | Community | Rural | 18+ | 18+ | 291 | 286 |
| 158 | Brazil | 2002-2004 | Ribeira Preto Birth Cohort | Community | Urban | 22-25 | 22-25 | 1,012 | 1,082 |
| 159 | Brazil | 2003-2005 | São Paulo Health and Ageing Study | Community | Urban | 65+ | 65+ | 783 | 1,198 |
| 160 | Brazil | 2004-2006 | Hearts of Brazil | National | Urban | 18+ | 18+ | 550 | 626 |
| 161 | Brazil | 2005 | Syndrome of Obesity and Risk Factors for Cardiovascular Disease Study | Community | Urban | 18-100 | 18-100 | 739 | 1,094 |
| 162 | Brazil | 2004-2005 | The 1982 Pelotas (Brazil) Birth Cohort: 23 years follow-up | Community | Urban | 23 | 23 | 2,173 | 1,935 |
| 163 | Brazil | 2006 | ATITUDE | Subnational | Both | 14-21 | 14-21 | 2,406 | 3,484 |
| 164 | Brazil | 2006 | Pesquisa Nacional de Demografia e Saude 2006 | National | Both |  | 15-49 |  | 14,783 |
| 165 | Brazil | 2006 | Krause et al., J Aging Phys Act 17:387-97, 2009 | Community | Urban | 60+ | 60+ | 93 | 1,069 |
| 166 | Brazil | 2008 | The Bambui Cohort Study of Ageing | Community | Urban | 71+ | 71+ | 248 | 456 |
| 167 | Brazil | 2008 | Caju & Virgen das Gracas | Community | Rural | 18+ | 18+ | 273 | 287 |
| 168 | Brazil | 2008-2009 | Pesquisa de Orcamentos Familiares | National | Both | 5+ | 5+ | 85,725 | 88,156 |
| 169 | Brazil | 2009-2010 | EpiFloripa Cohort Study of Ageing - Wave 1 | Community | Urban | 60+ | 60+ | 592 | 1,047 |
| 170 | Brazil | 2009-2010 | EpiFloripa Adults Cohort Study (EpiFloripa) | Community | Urban | 20-59 | 20-59 | 755 | 940 |
| 171 | Brazil | 2010 | San Pedro | Community | Rural | 18+ | 18+ | 153 | 214 |
| 172 | Brazil | 2012 | EpiFloripa Adults Cohort Study (EpiFloripa) | Community | Urban | 22-62 | 22-62 | 486 | 655 |
| 173 | Brazil | 2010-2015 | Baependi Heart Study | Community | Rural | 18+ | 18+ | 1,002 | 1,357 |
| 174 | Brazil | 2013 | Pesquisas Nacional de Saude | National | Both | 18+ | 18+ | 24,918 | 32,351 |
| 175 | Brazil | 2012-2013 | Prevalence of Leptin Polymorphism Gln223Arg | Community | Urban | 18+ | 18+ | 282 | 523 |
| 176 | Brazil | 2011-2014 | Profile of risk factors for coronary arterial disease in rio grande do sul - revaluation after 10 years | Subnational | Urban | 20+ | 20+ | 364 | 466 |
| 177 | Brazil | 2012-2013 | The 1982 Pelotas (Brazil) Birth Cohort: 30 years follow-up | Community | Urban | 30 | 30 | 1,753 | 1,798 |
| 178 | Brazil | 2013-2014 | EpiFloripa Cohort Study of Ageing - Wave 2 | Community | Urban | 63+ | 63+ | 404 | 744 |
| 179 | Brazil | 2014-2015 | EpiFloripa Adults Cohort Study (EpiFloripa) | Community | Urban | 25-65 | 25-65 | 353 | 476 |
| 180 | Brazil | 2015-2016 | Brazilian Longitudinal Study of the Elderly Health and Wellness | National | Both | 50+ | 50+ | 3,937 | 5,064 |
| 181 | Brazil | 2015-2016 | The 1993 Pelotas (Brazil) Birth Cohort: 22 years follow-up | Community | Urban | 21-23 | 21-23 | 1,687 | 1,872 |
| 182 | Brazil | 2017 | HealthRise Evaluation | Subnational | Both | 30+ | 30+ | 599 | 1,169 |
| 183 | Brazil | 2016-2017 | Study in Presidente Prudente | Community | Urban | 18+ | 18+ | 304 | 481 |
| 184 | Brazil | 2017-2018 | EpiFloripa Cohort Study of Ageing - Wave 3 | Community | Urban | 60+ | 60+ | 361 | 635 |
| 185 | Brazil | 2018-2019 | Epidemiology in the health (Santo Anastácio Edition) | Community | Urban | 18+ | 18+ | 105 | 145 |
| 186 | Brunei Darussalam | 2010-2011 | National Health And Nutritional Status Survey (NHANSS) | National | Both | 5-75 | 5-75 | 1,027 | 1,157 |
| 187 | Brunei Darussalam | 2015-2016 | National Non-Communicable Diseases Survey (NNCDS) | National | Both | 18-69 | 18-69 | 814 | 1,075 |
| 188 | Bulgaria | 2004 | National Nutrition Survey | National | Both | 15+ | 15+ | 515 | 515 |
| 189 | Burkina Faso | 1992-1993 | DHS | National | Both |  | 20-49 |  | 3,190 |
| 190 | Burkina Faso | 1998-1999 | DHS | National | Both |  | 20-49 |  | 3,114 |
| 191 | Burkina Faso | 2002 | Vulnérabilité Alimentaire et Sécurité Nutritionnelle dans la Gnagna (VASN-Gnagna) | Subnational | Rural | 5+ | 5+ | 1,471 | 3,522 |
| 192 | Burkina Faso | 2003 | DHS | National | Both |  | 15-49 |  | 11,001 |
| 193 | Burkina Faso | 2004 | Ouedraogo et al., Public Health Nutr 11:1280-87, 2008 | Community | Urban | 35+ | 35+ | 956 | 1,066 |
| 194 | Burkina Faso | 2010 | DHS | National | Both |  | 15-49 |  | 7,755 |
| 195 | Burkina Faso | 2013 | STEPS | National | Both | 25-64 | 25-64 | 2,223 | 2,250 |
| 196 | Burundi | 2010 | DHS | National | Both |  | 15-49 |  | 4,188 |
| 197 | Burundi | 2016-2017 | DHS | National | Both |  | 15-49 |  | 7,909 |
| 198 | Cabo Verde | 2007 | STEPS | National | Both | 25-64 | 25-64 | 658 | 1,066 |
| 199 | Cambodia | 2000 | DHS | National | Both |  | 15-49 |  | 6,915 |
| 200 | Cambodia | 2005 | DHS | National | Both |  | 15-49 |  | 8,130 |
| 201 | Cambodia | 2008 | Anthropometrics Survey | National | Both |  | 15-49 |  | 5,955 |

| 202 | Cambodia | 2010 | DHS | National | Both |  | 15-49 |  | 8,856 |
| --- | --- | --- | --- | --- | --- | --- | --- | --- | --- |
| 203 | Cambodia | 2010 | STEPS | National | Both | 25-64 | 25-64 | 1,881 | 3,344 |
| 204 | Cambodia | 2014 | DHS | National | Both |  | 15-49 |  | 10,821 |
| 205 | Cameroon | 1998 | DHS | National | Both |  | 20-49 |  | 1,429 |
| 206 | Cameroon | 1998-1999 | ENHIP | Community | Rural | 15+ | 15+ | 523 | 738 |
| 207 | Cameroon | 1998-1999 | ENHIP | Community | Urban | 15+ | 15+ | 523 | 640 |
| 208 | Cameroon | 2003 | STEPS | Subnational | Urban | 15+ | 15+ | 3,672 | 5,490 |
| 209 | Cameroon | 2004 | DHS | National | Both |  | 15-49 |  | 4,646 |
| 210 | Cameroon | 2007 | Cameroon Burden of Diabetes - Second Survey | Subnational | Urban | 18+ | 18+ | 3,345 | 4,633 |
| 211 | Cameroon | 2009 | National Survey of Micronutrient Status and Consumption of Fortifiable Foods | National | Both |  | 15-49 |  | 816 |
| 212 | Cameroon | 2011 | DHS | National | Both |  | 15-49 |  | 7,343 |
| 213 | Cameroon | 2009-2012 | Anthropologie nutritionnelle des migrants d'Afrique centrale à la ville et en France | Subnational | Both | 18-76 | 18-76 | 528 | 584 |
| 214 | Cameroon | 2014-2015 | Cardiovascular risk factors screening in urban and rural areas in the Far-North Region Cameroon | Subnational | Both | 20+ | 20+ | 520 | 369 |
| 215 | Cameroon | 2018-2019 | DHS | National | Both |  | 15-64 |  | 6,255 |
| 216 | Canada | 1985-1986 | INTERSAL, StJohns | Community | Urban | 20-59 | 20-59 | 100 | 100 |
| 217 | Canada | 1985-1988 | MONICA, Halifax | Community | Both | 25-64 | 25-64 | 438 | 420 |
| 218 | Canada | 1986-1992 | Canada Heart Health Survey | National | Both | 18-74 | 18-74 | 9,644 | 9,777 |
| 219 | Canada | 1993 | Chen et al., Int J Obes Relat Metab Disord 22:771-77, 1998 | Community | Rural | 18-74 | 18-74 | 803 | 988 |
| 220 | Canada | 1995 | MONICA, Halifax | Community | Both | 25-64 | 25-64 | 274 | 287 |
| 221 | Canada | 1995-1997 | Canadian Multicentre Osteoporosis Study (CaMos) | Subnational | Both | 35+ | 25+ | 2,616 | 6,343 |
| 222 | Canada | 1997 | PEI Nutrition Survey | Subnational | Both | 18-74 | 18-74 | 1,000 | 995 |
| 223 | Canada | 2005 | CCHS | National | Both | 15+ | 15+ | 1,684 | 2,031 |
| 224 | Canada | 2005-2008 | Canadian Multicentre Osteoporosis Study (CaMos) | Subnational | Both | 35+ | 35+ | 1,486 | 3,661 |
| 225 | Canada | 2008 | CCHS | National | Both | 15+ | 15+ | 1,689 | 1,988 |
| 226 | Canada | 2007-2009 | Canadian Health Measures Survey, Cycle 1 | National | Both | 6-79 | 6-79 | 2,703 | 2,864 |
| 227 | Canada | 2009-2011 | Canadian Health Measures Survey, Cycle 2 | National | Both | 5-79 | 5-79 | 2,870 | 3,086 |
| 228 | Canada | 2012-2013 | Canadian Health Measures Survey, Cycle 3 | National | Both | 5-79 | 5-79 | 2,670 | 2,676 |
| 229 | Canada | 2014-2015 | Canadian Health Measures Survey, Cycle 4 | National | Both | 5-79 | 5-79 | 2,697 | 2,674 |
| 230 | Canada | 2016-2017 | Canadian Health Measures Survey, Cycle 5 | National | Both | 6-79 | 6-79 | 2,571 | 2,564 |
| 231 | Central African Republic | 1994-1995 | DHS | National | Both |  | 20-49 |  | 1,760 |
| 232 | Central African Republic | 2010 | STEPS | Subnational | Both | 25-64 | 25-64 | 1,846 | 1,967 |
| 233 | Chad | 1996-1997 | DHS | National | Both |  | 20-49 |  | 3,262 |
| 234 | Chad | 2004 | DHS | National | Both |  | 20-49 |  | 2,618 |
| 235 | Chad | 2008 | STEPS | Community | Urban | 25-64 | 25-64 | 995 | 845 |
| 236 | Chad | 2014-2015 | DHS | National | Both |  | 15-49 |  | 9,733 |
| 237 | Chile | 1989 | INCLEN | Community | Urban | 35-65 |  | 199 |  |
| 238 | Chile | 1992-1993 | Miquel et al., Gastroenterology 115: 937-46, 1998 | Community | Urban | 18+ | 18+ | 657 | 1,031 |
| 239 | Chile | 2000 | Nervi et al., J Hepatol 45: 299 -305, 2006 | Community | Urban | 18+ | 18+ | 335 | 624 |
| 240 | Chile | 1999-2000 | The Survey on Health, Well-Being, and Aging in Latin America and the Caribbean (SABE) | Community | Urban | 60+ | 60+ | 410 | 806 |
| 241 | Chile | 2001-2003 | Bustos et al., Nutr Metab Cardiovasc Dis 17:581-89, 2007 | Community | Both | 22-28 | 22-28 | 436 | 562 |
| 242 | Chile | 2003 | Encuesta Nacional de Salud | National | Both | 17+ | 17+ | 1,557 | 1,867 |
| 243 | Chile | 2005 | Palomo et al., Rev Med Chil 135:904-12, 2007 | Community | Urban | 18-74 | 18-74 | 339 | 668 |
| 244 | Chile | 2004-2005 | CArdiovascular Risk factors Multiple Evaluation in Latin America | Community | Urban | 25-64 | 25-64 | 783 | 865 |
| 245 | Chile | 2009-2010 | Encuesta Nacional de Salud | National | Both | 15+ | 15+ | 1,935 | 2,869 |
| 246 | Chile | 2010-2011 | Encuesta Nacional de Consumo Alimentario | National | Both | 5+ | 5+ | 1,840 | 2,902 |
| 247 | Chile | 2011-2012 | CESCAS Study | Community | Urban | 30-79 | 30-79 | 922 | 1,020 |
| 248 | Chile | 2016-2017 | Encuesta Nacional de Salud | National | Both | 15+ | 15+ | 1,977 | 3,420 |
| 249 | China | 1984-1985 | Sino-MONICA Beijing | Community | Both | 25-64 | 25-64 | 813 | 857 |
| 250 | China | 1986 | INTERSALT, Beijing | Community | Urban | 20-59 | 20-59 | 100 | 100 |
| 251 | China | 1986 | INTERSALT, Nanning | Community | Both | 20-59 | 20-59 | 100 | 100 |
| 252 | China | 1986 | INTERSALT, Tianjin | Community | Urban | 20-59 | 20-59 | 100 | 100 |
| 253 | China | 1987 | INCLEN | Community | Urban | 35-65 |  | 989 |  |
| 254 | China | 1986-1989 | Ewang et al., Zhonghua Liu Xing Bing Xue Za Zhi 26:394-9, 2005 | Community | Both | 45-64 |  | 18,244 |  |
| 255 | China | 1988 | Sino-MONICA Hebei | Community | Both | 25-64 |  | 800 |  |
| 256 | China | 1988 | Sino-MONICA Heilongjiang | Community | Urban | 25-64 | 25-64 | 800 | 800 |
| 257 | China | 1988 | Sino-MONICA Henan | Community | Urban | 25-64 | 25-64 | 345 | 427 |
| 258 | China | 1988 | Sino-MONICA Neimenggu | Community | Urban | 25-64 | 25-64 | 396 | 400 |
| 259 | China | 1988 | Sino-MONICA Sichuan | Community | Both | 25-64 | 25-64 | 312 | 334 |
| 260 | China | 1988 | Sino-MONICA Shandong | Community | Urban | 25-64 | 25-64 | 211 | 225 |
| 261 | China | 1986-1989 | Sino-MONICA Shanghai | Community | Rural | 25-64 | 25-64 | 675 | 753 |
| 262 | China | 1988-1990 | East Beijing Study 2 | Community | Urban | 20-84 | 20-84 | 135 | 148 |
| 263 | China | 1989 | China Health and Nutrition Study | National | Both | 5-45 | 5-45 | 2,554 | 2,717 |
| 264 | China | 1989 | The Tianjin Project | Community | Urban | 15-64 | 15-64 | 3,894 | 3,971 |
| 265 | China | 1988-1989 | Wang et al., Zhonghua Liu Xing Bing Xue Za Zhi 24:272-75, 2003 | Community | Both | 25-64 | 25-64 | 873 | 731 |
| 266 | China | 1988-1989 | Sino-MONICA Beijing | Community | Both | 25-64 | 25-64 | 701 | 862 |
| 267 | China | 1989 | Sino-MONICA Fujian | Community | Urban | 25-64 | 25-64 | 179 | 191 |
| 268 | China | 1988-1989 | Sino-MONICA Jilin | Community | Urban | 25-64 | 25-64 | 380 | 400 |
| 269 | China | 1989 | Sino-MONICA Jiangsu | Community | Rural | 25-64 | 25-64 | 398 | 399 |

| 270 | China | 1988-1989 | Sino-MONICA Jiangxi | Community | Urban | 25-64 | 25-64 | 379 | 386 |
| --- | --- | --- | --- | --- | --- | --- | --- | --- | --- |
| 271 | China | 1988-1989 | Sino-MONICA Liaoning | Community | Both | 25-64 | 25-64 | 728 | 734 |
| 272 | China | 1991 | China Health and Nutrition Study | National | Both | 5+ | 5+ | 5,584 | 5,922 |
| 273 | China | 1990-1991 | China Prospective Study | National | Both | 40-79 |  | 230,676 |  |
| 274 | China | 1991 | China National Hypertension Survey Epidemiology Follow-up Study | National | Both | 40+ | 40+ | 75,696 | 79,040 |
| 275 | China | 1991 | Sino-MONICA Shanghai | Community | Rural | 30-64 | 30-64 | 564 | 624 |
| 276 | China | 1992-1993 | Anzhen 02 Cohort Study | Community | Urban | 34-65 | 34-65 | 2,032 | 2,120 |
| 277 | China | 1991-1992 | Fangshan Cohort Study | Community | Urban | 34-86 | 34-86 | 871 | 1,736 |
| 278 | China | 1992 | Huashan Study | Community | Urban | 35-75 | 35-75 | 892 | 965 |
| 279 | China | 1992 | China National Nutrition Survey | National | Both | 5+ | 5+ | 33,714 | 36,271 |
| 280 | China | 1992 | Sino-MONICA Sichuan | Community | Both | 25-64 | 25-64 | 608 | 526 |
| 281 | China | 1993 | China Health and Nutrition Study | National | Both | 5+ | 5+ | 5,370 | 5,564 |
| 282 | China | 1993 | Wang et al., Zhonghua Liu Xing Bing Xue Za Zhi 24:272-75, 2003 | Community | Both | 25-64 | 25-64 | 822 | 617 |
| 283 | China | 1993 | Sino-MONICA Anhui | Community | Urban | 25-64 | 25-64 | 193 | 195 |
| 284 | China | 1993 | Sino-MONICA Beijing | Community | Both | 25-64 | 25-64 | 613 | 816 |
| 285 | China | 1993 | Sino-MONICA Jiangsu | Community | Urban | 25-64 | 25-64 | 462 | 365 |
| 286 | China | 1993 | Sino-MONICA Liaoning | Community | Both | 25-64 | 25-64 | 493 | 500 |
| 287 | China | 1996 | Wang et al., Zhonghua Liu Xing Bing Xue Za Zhi 24:272-75, 2003 | Community | Both | 25-64 | 25-64 | 735 | 721 |
| 288 | China | 1996 | The Tianjin Project | Community | Urban | 15-64 | 15-64 | 722 | 717 |
| 289 | China | 1997 | China Health and Nutrition Study | National | Both | 5+ | 5+ | 5,551 | 5,647 |
| 290 | China | 1997 | INTERMAP, Beijing | Community | Rural | 40-59 | 40-59 | 133 | 139 |
| 291 | China | 1997 | INTERMAP, Guangxi | Community | Rural | 40-59 | 40-59 | 140 | 138 |
| 292 | China | 1997 | INTERMAP, Shanxi | Community | Rural | 40-59 | 40-59 | 143 | 146 |
| 293 | China | 1998 | Shanghai Diabetes Study | Community | Urban | 25+ | 25+ | 1,264 | 1,768 |
| 294 | China | 1996-2000 | Shanghai Women's Health Study | Community | Urban |  | 40-70 |  | 74,915 |
| 295 | China | 1999 | Chen et al., Zhonghua Yi Xue Za Zhi 85(40):2830-4, 2005 | Subnational | Both | 35-85 | 35-85 | 13,549 | 10,315 |
| 296 | China | 1999 | Wang et al., Zhonghua Liu Xing Bing Xue Za Zhi 24:272-75, 2003 | Community | Both | 25-64 | 25-64 | 818 | 685 |
| 297 | China | 1998-2000 | Jia et al., Obes Rev 3:157-65, 2002 | Community | Urban | 20+ | 20+ | 1,106 | 1,670 |
| 298 | China | 2000 | China Health and Nutrition Study | National | Both | 5+ | 5+ | 5,828 | 6,100 |
| 299 | China | 1996-2003 | Wu et al., Osteoporos Int 15:751-59, 2004 | Community | Urban |  | 18+ |  | 3,418 |
| 300 | China | 1999-2000 | Xu et al., Public Health Nutr 8:47-51, 2005 | Community | Both | 35+ | 35+ | 18,194 | 18,902 |
| 301 | China | 2001 | Shanghai Diabetes Study | Community | Urban | 25+ | 25+ | 1,264 | 1,768 |
| 302 | China | 2000-2001 | The International Collaborative Study of Cardiovascular Disease in Asia | National | Both | 35-74 | 35-74 | 7,512 | 8,006 |
| 303 | China | 2002 | Ma et al., Zhonghua Liu Xing Bing Xue Za Zhi 25:1035-8, 2004 | Subnational | Both | 18+ | 18+ | 7,352 | 7,352 |
| 304 | China | 2002 | China National Nutrition and Health Survey | National | Both | 5-101 | 5-101 | 84,194 | 92,687 |
| 305 | China | 2002-2003 | Fan et al., J Gastroenterol Hepatol 20:1825-32, 2005 | Community | Urban | 15+ | 15-74 | 5,502 | 7,767 |
| 306 | China | 2004 | China Health and Nutrition Study | National | Both | 5+ | 5+ | 5,228 | 5,517 |
| 307 | China | 2004 | Tian et al., Prev Med 48:59-63, 2009 | Community | Rural | 15+ | 15+ | 1,022,669 | 1,163,313 |
| 308 | China | 2002-2006 | Shanghai Men's Health Study | Community | Urban | 40-74 |  | 61,445 |  |
| 309 | China | 2004-2006 | Pang et al., Intern Med 47:893-97, 2008 | Community | Rural | 35+ | 35+ | 22,963 | 22,962 |
| 310 | China | 2005 | Ye et al., J Am Coll Cardiol 49:1798-805, 2007 | Community | Urban | 50-70 | 50-70 | 743 | 906 |
| 311 | China | 2004-2006 | Shanghai Women's Health Study | Community | Urban |  | 45-80 |  | 64,545 |
| 312 | China | 2006 | Beijing Eye Study | Community | Both | 45+ | 45+ | 1,394 | 1,820 |
| 313 | China | 2006 | China Health and Nutrition Study | National | Both | 5+ | 5+ | 4,948 | 5,399 |
| 314 | China | 2004-2008 | China Kadoorie Biobank baseline survey | Subnational | Rural | 35-74 | 35-74 | 115,792 | 162,848 |
| 315 | China | 2004-2008 | China Kadoorie Biobank baseline survey | Subnational | Urban | 35-74 | 35-74 | 89,219 | 132,860 |
| 316 | China | 2005-2006 | Zhou et al., World J Gastroenterol 13:6419-24, 2007 | Community | Urban | 18-79 | 18-79 | 1,101 | 2,063 |
| 317 | China | 2004-2008 | Shanghai Men's Health Study | Community | Urban | 41-80 |  | 54,800 |  |
| 318 | China | 2006-2007 | Handan Eye Study | Community | Rural | 30+ | 30+ | 2,995 | 3,456 |
| 319 | China | 2007-2008 | China National Diabetes & Metabolic Disorders Study | National | Both | 20+ | 20+ | 18,419 | 27,820 |
| 320 | China | 2008 | China Health and Retirement Longitudinal Study (CHARLS), pilot survey | Subnational | Both | 45+ | 45+ | 923 | 950 |
| 321 | China | 2008-2009 | Chinese Longitudinal Healthy Longevity Survey | National | Both | 65+ | 65+ | 6,827 | 8,976 |
| 322 | China | 2009 | China Health and Nutrition Study | National | Both | 5+ | 5+ | 5,176 | 5,489 |
| 323 | China | 2007-2010 | SAGE | National | Both | 50+ | 50+ | 5,759 | 6,616 |
| 324 | China | 2008-2010 | Fangshan Family-based Ischemic Stroke Study in China (FISSIC) program | Community | Rural | 40+ | 40+ | 19,478 | 36,449 |
| 325 | China | 2007-2011 | Shanghai Women's Health Study | Community | Urban |  | 47-83 |  | 52,116 |
| 326 | China | 2009-2010 | China National Survey of Chronic Kidney Disease | National | Both | 18+ | 18+ | 20,003 | 26,854 |
| 327 | China | 2010 | China Noncommunicable Disease Surveillance | National | Both | 18+ | 18+ | 45,066 | 53,452 |
| 328 | China | 2008-2011 | Shanghai Men's Health Study | Community | Urban | 43-84 |  | 51,948 |  |
| 329 | China | 2011 | Beijing Eye Study | Community | Both | 50+ | 50+ | 1,467 | 1,895 |
| 330 | China | 2011-2012 | China Health and Retirement Longitudinal Study (CHARLS), baseline survey | National | Both | 45+ | 45+ | 6,337 | 7,003 |
| 331 | China | 2011 | China Health and Nutrition Study | National | Both | 5+ | 5+ | 6,771 | 7,477 |
| 332 | China | 2012 | China Health and Retirement Longitudinal Study (CHARLS), wave 2 pilot survey | Subnational | Both | 45+ | 45+ | 856 | 934 |
| 333 | China | 2011-2012 | Chinese Longitudinal Healthy Longevity Survey | National | Both | 65+ | 65+ | 4,035 | 4,620 |
| 334 | China | 2010-2014 | National Free Preconception Health Examination Project | National | Rural | 20-64 |  | 16,166,534 |  |
| 335 | China | 2013 | China Health and Retirement Longitudinal Study (CHARLS), wave 2 survey | National | Both | 45+ | 45+ | 5,898 | 6,582 |
| 336 | China | 2013 | Gobi Desert Children Eye Study | Community | Urban | 6-21 | 6-21 | 800 | 761 |
| 337 | China | 2012-2013 | The Kailuan Study | Community | Urban | 18+ | 18+ | 80,921 | 21,385 |

| 338 | China | 2014 | Chinese Longitudinal Healthy Longevity Survey | National | Both | 65+ | 65+ | 2,978 | 3,172 |
| --- | --- | --- | --- | --- | --- | --- | --- | --- | --- |
| 339 | China | 2012-2015 | Shanghai Men's Health Study | Community | Urban | 47-87 |  | 40,921 |  |
| 340 | China | 2012-2015 | Shanghai Women's Health Study | Community | Urban |  | 52-88 |  | 49,592 |
| 341 | China | 2015 | China Health and Retirement Longitudinal Study (CHARLS), wave 4 survey | National | Both | 45+ | 45+ | 7,032 | 7,719 |
| 342 | China | 2015 | China Health and Nutrition Study | National | Both | 5+ | 5+ | 5,419 | 5,765 |
| 343 | China | 2014-2015 | The Kailuan Study | Community | Urban | 18+ | 18+ | 73,161 | 18,280 |
| 344 | China (Hong Kong SAR) | 1985-1986 | Shatin New Town Study | Community | Urban | 70+ | 70+ | 276 | 669 |
| 345 | China (Hong Kong SAR) | 1991 | The Hong Kong study on health, health risk and quality of life in the Chinese elderly cohort | Community | Both | 70+ | 70+ | 943 | 944 |
| 346 | China (Hong Kong SAR) | 1995-1996 | Hong Kong Cardiovascular Risk Factor Prevalence Study 1995-1996 | National | Both | 25-74 | 25-74 | 1,412 | 1,478 |
| 347 | Colombia | 1986 | INTERSALT | Community | Rural | 20-59 | 20-59 | 96 | 95 |
| 348 | Colombia | 1995 | DHS | National | Both |  | 20-49 |  | 3,068 |
| 349 | Colombia | 2000 | DHS | National | Both |  | 20-49 |  | 2,929 |
| 350 | Colombia | 2001 | CINDI/CARMEN - Bucaramaga | Community | Urban | 15-74 | 15-74 | 627 | 1,218 |
| 351 | Colombia | 2002 | Factores de riesgo cardiovascular en la localidad de Santa Fe de la ciudad de Bogotá. Resultados obtenidos en el área demostrativa Carmen | Community | Urban | 15-69 | 15-69 | 394 | 684 |
| 352 | Colombia | 2002 | Factores de riesgo cardiovascular en la localidad de Tunjuelito de la ciudad de Bogotá. Resultados obtenidos en el área demostrativa Carmen | Community | Urban | 15-29 | 15-29 | 208 | 312 |
| 353 | Colombia | 2002 | CINDI/CARMEN - Bogota | Community | Urban | 15-74 | 15-74 | 322 | 570 |
| 354 | Colombia | 2005 | DHS | National | Both | 5-64 | 5-64 | 43,436 | 57,778 |
| 355 | Colombia | 2005 | Encuesta Nacional de Situacion Nutricional | National | Both | 5-12 | 5-49 | 2,644 | 6,088 |
| 356 | Colombia | 2004-2005 | CArdiovascular Risk factors Multiple Evaluation in Latin America | Community | Urban | 25-64 | 25-64 | 738 | 812 |
| 357 | Colombia | 2007 | Encuesta Nacional de Salud | National | Both | 18-69 | 18-69 | 5,462 | 7,686 |
| 358 | Colombia | 2010 | DHS | National | Both | 5-64 | 5-64 | 65,086 | 76,792 |
| 359 | Colombia | 2010 | STEPS | Subnational | Both | 15-64 | 15-64 | 1,034 | 1,356 |
| 360 | Colombia | 2015 | Encuesta Nacional de Situacion Nutricional | National | Both | 5-64 | 5-64 | 4,874 | 5,742 |
| 361 | Colombia | 2015 | STEPS | Subnational | Both | 15-64 | 15-64 | 979 | 1,181 |
| 362 | Comoros | 1996 | DHS | National | Both |  | 20-49 |  | 744 |
| 363 | Comoros | 2011 | STEPS | National | Both | 25-64 | 25-64 | 1,541 | 3,505 |
| 364 | Comoros | 2012 | DHS | National | Both |  | 15-49 |  | 4,845 |
| 365 | Congo | 1986 | Enquête Brazzaville 1986 | Community | Urban | 5-50 | 5-50 | 129 | 1,079 |
| 366 | Congo | 1987 | Enquête Nationale Congo 1987 | National | Rural |  | 13-49 |  | 1,356 |
| 367 | Congo | 1987 | Maire et al., Rev Epidemiol Sante Publique 40:252-58, 1992 | Community | Rural |  | 16-45 |  | 750 |
| 368 | Congo | 1991 | Enquête Brazzaville 1991 | Community | Urban | 5-90 | 5-90 | 2,393 | 3,149 |
| 369 | Congo | 1996 | Enquête Brazzaville 1996 | Community | Urban | 5-90 | 5-90 | 2,496 | 3,073 |
| 370 | Congo | 2004 | STEPS | Community | Urban | 25-64 | 25-64 | 1,013 | 956 |
| 371 | Congo | 2005 | DHS | National | Both |  | 15-49 |  | 6,266 |
| 372 | Congo | 2011-2012 | DHS | National | Both |  | 15-49 |  | 5,060 |
| 373 | Cook Islands | 2003 | STEPS | National | Both | 25-64 | 25-64 | 925 | 958 |
| 374 | Cook Islands | 2013-2015 | STEPS | National | Both | 18-64 | 18-64 | 456 | 469 |
| 375 | Costa Rica | 2004 | CAMDI | Community | Urban | 20+ | 20+ | 304 | 624 |
| 376 | Costa Rica | 2004-2006 | Costa Rican Longevity and Healthy Aging Study Pre-1945 Cohort Wave 1 | National | Both | 60+ | 60+ | 1,163 | 1,346 |
| 377 | Costa Rica | 2006-2008 | Costa Rican Longevity and Healthy Aging Study Pre-1945 Cohort Wave 2 | National | Both | 62+ | 62+ | 944 | 1,102 |
| 378 | Costa Rica | 2009-2010 | Costa Rican Longevity and Healthy Aging Study Pre-1945 Cohort Wave 3 | National | Both | 64+ | 64+ | 737 | 887 |
| 379 | Costa Rica | 2008-2009 | Encuesta Nacional de Nutricion 2008-2009 | National | Both |  | 45-64 |  | 661 |
| 380 | Costa Rica | 2010 | Costa Rican National Cardiovascular Risk Factors Survey, 2010 | National | Both | 20+ | 20+ | 778 | 1,958 |
| 381 | Costa Rica | 2010-2011 | Costa Rican Longevity and Healthy Aging Study 1945-1955 Cohort Wave 1 | National | Both | 54-66 | 54-66 | 1,058 | 1,676 |
| 382 | Costa Rica | 2012-2014 | Costa Rican Longevity and Healthy Aging Study 1945-1955 Cohort Wave 2 | National | Both | 56-68 | 56-68 | 867 | 1,470 |
| 383 | Costa Rica | 2014 | Costa Rican National Cardiovascular Risk Factors Survey, 2014 | National | Both | 20+ | 20+ | 1,003 | 2,196 |
| 384 | Cote d'Ivoire | 1994 | DHS | National | Both |  | 20-49 |  | 2,682 |
| 385 | Cote d'Ivoire | 1998-1999 | DHS | National | Both |  | 15-49 |  | 2,740 |
| 386 | Cote d'Ivoire | 2005 | STEPS | Subnational | Rural | 15-64 | 15-64 | 894 | 1,022 |
| 387 | Cote d'Ivoire | 2005 | STEPS | Subnational | Urban | 15-64 | 15-64 | 1,071 | 1,437 |
| 388 | Cote d'Ivoire | 2011-2012 | DHS | National | Both |  | 15-49 |  | 4,601 |
| 389 | Croatia | 1997-1999 | Budak A et al., Lijec Vjesn 125(1-2):32-5, 2003 | National | Both | 25-100 | 25-100 | 1,763 | 2,684 |
| 390 | Croatia | 2002-2007 | Epidemiology of arterial hypertension in Croatia (EH-UH) | National | Both | 18+ | 18+ | 505 | 705 |
| 391 | Croatia | 2005 | Endemic Nephropathy and Arterial hypertension (ENAH) | Subnational | Rural | 18+ | 18+ | 264 | 367 |
| 392 | Croatia | 2008 | Endemic Nephropathy and Arterial hypertension (ENAH) | Subnational | Rural | 18+ | 18+ | 331 | 527 |
| 393 | Croatia | 2010 | Endemic Nephropathy and Arterial hypertension (ENAH) | Subnational | Rural | 18+ | 18+ | 252 | 393 |
| 394 | Croatia | 2015 | Endemic Nephropathy and Arterial hypertension (ENAH) Follow-up Study | Subnational | Rural | 18+ | 18+ | 224 | 460 |
| 395 | Cuba | 1999-2000 | The Survey on Health, Well-Being, and Aging in Latin America and the Caribbean (SABE) | Community | Urban | 60+ | 60+ | 630 | 1,044 |
| 396 | Cuba | 2001-2002 | National Survey of Risk Factors | National | Both | 20-60 | 20-60 | 11,426 | 11,426 |
| 397 | Cuba | 2010 | National Risk Factor Survey | National | Both | 15+ | 15+ | 3,344 | 3,868 |
| 398 | Cuba | 2011 | Non communicable disease risk factor in Cienfuegos | Community | Urban | 15-80 | 15-80 | 617 | 880 |
| 399 | Cyprus | 1999-2000 | Countrywide Integrated Noncommunicable Diseases Intervention Programme Cyprus | National | Both | 25-65 | 25-65 | 457 | 546 |
| 400 | Czech Republic | 1985 | Czech-MONICA | National | Both | 25-64 | 25-64 | 1,243 | 1,303 |
| 401 | Czech Republic | 1988 | Czech-MONICA | National | Both | 25-64 | 25-64 | 1,357 | 1,408 |
| 402 | Czech Republic | 1992 | Czech-MONICA | National | Both | 25-64 | 25-64 | 1,131 | 1,207 |
| 403 | Czech Republic | 1997-1998 | Czech post-MONICA | National | Both | 25-64 | 25-64 | 1,527 | 1,665 |
| 404 | Czech Republic | 1998-2002 | Health, Lifestyle and the Environment | National | Urban | 45-54 | 45-54 | 1,539 | 2,044 |
| 405 | Czech Republic | 2000-2001 | Czech post-MONICA | National | Both | 25-64 | 25-64 | 1,628 | 1,690 |

| 406 | Czech Republic | 2002-2005 | Health, Alcohol and Psychosocial Factors In Eastern Europe | Subnational | Urban | 44-72 | 44-72 | 3,321 | 3,935 |
| --- | --- | --- | --- | --- | --- | --- | --- | --- | --- |
| 407 | Czech Republic | 2004-2005 | Health, Lifestyle and the Environment | National | Urban | 45-54 | 45-54 | 775 | 1,072 |
| 408 | Czech Republic | 2006-2009 | Czech post-MONICA | National | Both | 25-64 | 25-64 | 1,717 | 1,861 |
| 409 | Czech Republic | 2009 | Health, Lifestyle and the Environment | National | Urban | 45-54 | 45-54 | 307 | 447 |
| 410 | Czech Republic | 2014-2015 | European Heath Examination Survey | National | Both | 25-64 | 25-64 | 480 | 699 |
| 411 | Czech Republic | 2015-2018 | MONICA | National | Both | 25-65 | 25-65 | 1,247 | 1,371 |
| 412 | Denmark | 1985 | INTERSALT | Community | Urban | 20-59 | 20-59 | 99 | 100 |
| 413 | Denmark | 1984-1985 | The Epidemiology of Gallstones in a 70 Year-Old Danish Population | Community | Both | 70 | 70 | 202 | 172 |
| 414 | Denmark | 1986-1987 | MONICA, Glostrup | Community | Urban | 29-61 | 29-61 | 746 | 753 |
| 415 | Denmark | 1987 | Nilsson et al., J Intern Med 237:479-86, 1995 | Community | Urban | 51 |  | 439 |  |
| 416 | Denmark | 1987-1988 | MONICA I, 5-years re-examination (semi-MONICA) | Community | Urban | 35-66 | 35-66 | 1,524 | 1,463 |
| 417 | Denmark | 1991-1992 | MONICA, Glostrup | Community | Urban | 29-61 | 29-61 | 808 | 816 |
| 418 | Denmark | 1991-1994 | Copenhagen City Heart Study | Subnational | Urban | 20+ | 20+ | 4,274 | 5,416 |
| 419 | Denmark | 1993-1994 | MONICA 1 - 10 years examination (semi-MONICA) | Community | Urban | 41-72 | 41-72 | 1,333 | 1,323 |
| 420 | Denmark | 1993-1997 | EPIC Aarhus | Community | Urban | 50-65 | 50-65 | 8,430 | 8,717 |
| 421 | Denmark | 1993-1997 | EPIC Copenhagen | Community | Urban | 50-65 | 50-65 | 18,729 | 21,133 |
| 422 | Denmark | 1996-1997 | Drivsholm et al., Diabet Med 18:126-32, 2001 | Subnational | Urban | 60 | 60 | 325 | 370 |
| 423 | Denmark | 2001-2003 | Copenhagen City Heart Study | Subnational | Urban | 20+ | 20+ | 2,567 | 3,446 |
| 424 | Denmark | 2002-2003 | Odense Androgen Study | Community | Urban | 20-29 |  | 783 |  |
| 425 | Denmark | 2003-2004 | Copenhagen General Population Study 1 | Subnational | Urban | 20+ | 20+ | 4,828 | 5,397 |
| 426 | Denmark | 2005 | Copenhagen General Population Study 1 | Subnational | Urban | 20+ | 20+ | 5,171 | 6,023 |
| 427 | Denmark | 2006 | Danish Conscript Register | National | Both | 17-26 |  | 25,063 |  |
| 428 | Denmark | 2006 | Copenhagen General Population Study 1 | Subnational | Urban | 20+ | 20+ | 5,055 | 4,861 |
| 429 | Denmark | 2007 | Danish Conscript Register | National | Both | 17-26 |  | 27,194 |  |
| 430 | Denmark | 2007 | Copenhagen General Population Study 1 | Subnational | Urban | 20+ | 20+ | 4,027 | 6,913 |
| 431 | Denmark | 2006-2008 | The Health2006 Cohort | Subnational | Urban | 18-71 | 18-71 | 1,553 | 1,916 |
| 432 | Denmark | 2008 | Danish Conscript Register | National | Both | 17-26 |  | 24,538 |  |
| 433 | Denmark | 2007-2008 | The Danish Health Examination Survey 2007-2008 | National | Both | 18+ | 18+ | 7,349 | 10,651 |
| 434 | Denmark | 2008 | Copenhagen General Population Study 1 | Subnational | Urban | 20+ | 20+ | 4,735 | 6,467 |
| 435 | Denmark | 2009 | Danish Conscript Register | National | Both | 17-26 |  | 27,093 |  |
| 436 | Denmark | 2009 | Copenhagen General Population Study 1 | Subnational | Urban | 20+ | 20+ | 4,214 | 5,244 |
| 437 | Denmark | 2010 | Danish Conscript Register | National | Both | 17-26 |  | 30,814 |  |
| 438 | Denmark | 2009-2010 | The European Youth Heart Study | Community | Both | 14-28 | 14-28 | 481 | 553 |
| 439 | Denmark | 2010 | Copenhagen General Population Study 1 | Subnational | Urban | 20+ | 20+ | 3,967 | 4,891 |
| 440 | Denmark | 2011 | Danish Conscript Register | National | Both | 17-26 |  | 30,719 |  |
| 441 | Denmark | 2011 | Copenhagen General Population Study 1 | Subnational | Urban | 20+ | 20+ | 4,935 | 5,824 |
| 442 | Denmark | 2012 | Danish Conscript Register | National | Both | 17-26 |  | 29,651 |  |
| 443 | Denmark | 2012 | Copenhagen General Population Study 1 | Subnational | Urban | 20+ | 20+ | 4,516 | 5,423 |
| 444 | Denmark | 2011-2012 | The Health2006 cohort - 5-year follow-up | Subnational | Urban | 24-76 | 24-76 | 1,057 | 1,249 |
| 445 | Denmark | 2013 | Danish Conscript Register | National | Both | 17-26 |  | 30,565 |  |
| 446 | Denmark | 2013 | Copenhagen General Population Study 1 | Subnational | Urban | 20+ | 20+ | 4,001 | 4,703 |
| 447 | Denmark | 2014 | Danish Conscript Register | National | Both | 17-26 |  | 32,397 |  |
| 448 | Denmark | 2014 | Copenhagen General Population Study 2 | Subnational | Urban | 20+ | 20+ | 1,391 | 1,782 |
| 449 | Denmark | 2012-2015 | The Danish study of Functional Disorders (DanFunD) | Subnational | Urban | 18-72 | 18-72 | 3,451 | 4,034 |
| 450 | Denmark | 2015 | Danish Conscript Register | National | Both | 17-26 |  | 28,907 |  |
| 451 | Denmark | 2014-2015 | Copenhagen General Population Study 1 | Subnational | Urban | 20+ | 20+ | 2,245 | 2,837 |
| 452 | Denmark | 2015 | Copenhagen General Population Study 2 | Subnational | Urban | 20+ | 20+ | 4,103 | 5,107 |
| 453 | Denmark | 2016 | Copenhagen General Population Study 2 | Subnational | Urban | 20+ | 20+ | 4,493 | 5,495 |
| 454 | Denmark | 2016 | Conscript | National | Both | 17-29 |  | 29,057 |  |
| 455 | Denmark | 2017 | Copenhagen General Population Study 2 | Subnational | Urban | 20+ | 20+ | 3,182 | 4,395 |
| 456 | Denmark | 2017 | Conscript | National | Both | 17-29 |  | 31,057 |  |
| 457 | Denmark | 2018 | Conscript | National | Both | 17-29 |  | 27,597 |  |
| 458 | Denmark | 2019 | Conscript | National | Both | 17-29 |  | 25,412 |  |
| 459 | Dominica | 2007 | STEPS | National | Both | 15-64 | 15-64 | 459 | 568 |
| 460 | Dominican Republic | 1991 | DHS | National | Both |  | 20-49 |  | 1,965 |
| 461 | Dominican Republic | 1993 | Aono et al., J Epidemiol 7(4):238-43, 1997 | National | Both | 20-70 | 20-70 | 767 | 1,149 |
| 462 | Dominican Republic | 1996 | DHS | National | Both |  | 15-49 |  | 7,441 |
| 463 | Dominican Republic | 1996-1998 | Estudio factores de riesgo cardiovascular y sindrome metabolico en la Republica Dominicana I (EFRICARD I) | National | Both | 18-75 | 18-75 | 2,087 | 4,095 |
| 464 | Dominican Republic | 2010-2012 | Estudio factores de riesgo cardiovascular y sindrome metabolico en la Republica Dominicana II (EFRICARD II) | National | Both | 18-75 | 18-75 | 1,641 | 3,254 |
| 465 | Dominican Republic | 2013 | DHS | National | Both | 15-59 | 15-49 | 10,433 | 8,960 |
| 466 | DR Congo | 2001 | Multiple Indicator Cluster Survey Round 2 | National | Both |  | 15-49 |  | 5,520 |
| 467 | DR Congo | 2005 | STEPS | Subnational | Urban | 15+ | 15+ | 761 | 1,152 |
| 468 | DR Congo | 2007 | DHS | National | Both |  | 15-49 |  | 4,137 |
| 469 | DR Congo | 2013-2014 | DHS | National | Both |  | 15-49 |  | 8,163 |
| 470 | Ecuador | 2004 | Encuesta Demografica y de Salud Materno e Infantil/Reproductive Health Survey | National | Both |  | 15-49 |  | 3,850 |
| 471 | Ecuador | 2004-2005 | CArdiovascular Risk factors Multiple Evaluation in Latin America | Community | Urban | 25-64 | 25-64 | 813 | 814 |
| 472 | Ecuador | 2009-2010 | National Survey of Health, Wellbeing, and Aging | National | Both | 60+ | 60+ | 2,341 | 2,592 |
| 473 | Ecuador | 2011-2013 | Encuesta Nacional de Salud y Nutrición (ENSANUT) | National | Both | 5-59 | 5-59 | 22,919 | 25,767 |

| 474 | Ecuador | 2018 | Encuesta Nacional de Salud y Nutrición (ENSANUT) | National | Both | 5+ | 5+ | 63,176 | 65,597 |
| --- | --- | --- | --- | --- | --- | --- | --- | --- | --- |
| 475 | Egypt | 1992 | DHS | National | Both |  | 20-49 |  | 4,654 |
| 476 | Egypt | 1995 | DHS | National | Both |  | 20-49 |  | 6,499 |
| 477 | Egypt | 2000 | DHS | National | Both |  | 20-49 |  | 13,602 |
| 478 | Egypt | 2002 | National Survey of Smoking, Obesity, Blood Pressure and Blood Glucose | National | Both | 5+ | 5+ | 4,397 | 5,161 |
| 479 | Egypt | 2003 | DHS | National | Both |  | 20-49 |  | 7,930 |
| 480 | Egypt | 2003-2004 | Marzouk et al., Gut 56(8):1105-10, 2007 | Community | Rural | 25+ | 25+ | 322 | 456 |
| 481 | Egypt | 2005 | DHS | National | Both |  | 20-49 |  | 16,864 |
| 482 | Egypt | 2005 | STEPS | National | Both | 15-65 | 15-65 | 4,757 | 4,428 |
| 483 | Egypt | 2008 | DHS | National | Both | 10-59 | 20-49 | 14,261 | 15,242 |
| 484 | Egypt | 2007-2009 | Mostafa et al., Gut 59(8):1135-40, 2010 | Community | Rural | 35+ | 35+ | 642 | 843 |
| 485 | Egypt | 2011 | STEPS | National | Both | 15-65 | 15-65 | 1,761 | 2,977 |
| 486 | Egypt | 2014 | DHS | National | Both |  | 20-49 |  | 18,891 |
| 487 | Egypt | 2015 | DHS | National | Both | 15-59 | 15-59 | 7,235 | 8,471 |
| 488 | Egypt | 2017 | STEPS | National | Both | 15-69 | 15-69 | 2,273 | 3,692 |
| 489 | El Salvador | 2002-2003 | Ecuesta Nacional de Salud Familiar | National | Both |  | 15-49 |  | 3,885 |
| 490 | El Salvador | 2004 | CAMDI | Community | Urban | 20+ | 20+ | 396 | 811 |
| 491 | El Salvador | 2008 | Ecuesta Nacional de Salud Familiar | National | Both |  | 15-49 |  | 6,808 |
| 492 | El Salvador | 2014-2015 | Encuesta Nacional de Enfermedades Crónicas (ENECA-ELS) | National | Both | 20+ | 20+ | 1,684 | 2,945 |
| 493 | Equatorial Guinea | 2011 | DHS | National | Both |  | 15-49 |  | 1,074 |
| 494 | Eritrea | 1995 | DHS | National | Both |  | 15-49 |  | 1,621 |
| 495 | Eritrea | 2002 | DHS | National | Both |  | 15-49 |  | 3,223 |
| 496 | Eritrea | 2004 | STEPS | National | Both | 15-64 | 15-64 | 1,113 | 1,089 |
| 497 | Eritrea | 2010 | STEPS | National | Both | 25-74 | 25-74 | 1,712 | 4,285 |
| 498 | Estonia | 1984-1986 | Abina et al., Blood Press 12:111-21, 2003 | Community | Urban | 20-54 | 30-54 | 2,477 | 851 |
| 499 | Estonia | 1992-1994 | Abina et al., Blood Press 12:111-21, 2003 | Community | Urban | 20-54 | 20-54 | 921 | 678 |
| 500 | Estonia | 1997 | Pomerleau et al., Public Health Nutrition 3:3-10, 2000 | National | Both | 19-64 | 19-64 | 525 | 629 |
| 501 | Estonia | 1999-2001 | Abina et al., Blood Press 12:111-21, 2003 | Community | Urban | 20-54 | 20-54 | 635 | 692 |
| 502 | Estonia | 2002 | Estonian Biobank | National | Both | 18+ | 18+ | 89 | 217 |
| 503 | Estonia | 2003 | The European Male Ageing Study | Community | Both | 40+ |  | 416 |  |
| 504 | Estonia | 2003 | Estonian Biobank | National | Both | 18+ | 18+ | 2,695 | 5,688 |
| 505 | Estonia | 2004 | Estonian Biobank | National | Both | 18+ | 18+ | 527 | 947 |
| 506 | Estonia | 2007 | Estonian Biobank | National | Both | 18+ | 18+ | 1,000 | 2,187 |
| 507 | Estonia | 2008 | The European Male Ageing Study | Community | Both | 40+ |  | 305 |  |
| 508 | Estonia | 2008 | Estonian Biobank | National | Both | 18+ | 18+ | 5,147 | 10,990 |
| 509 | Estonia | 2009 | Estonian Biobank | National | Both | 18+ | 18+ | 3,963 | 6,493 |
| 510 | Estonia | 2010 | Estonian Biobank | National | Both | 18+ | 18+ | 4,052 | 7,045 |
| 511 | Estonia | 2011 | Estonian Biobank | National | Both | 18+ | 18+ | 111 | 176 |
| 512 | Estonia | 2012 | Estonian Biobank | National | Both | 18+ | 18+ | 85 | 130 |
| 513 | Estonia | 2013 | Estonian Biobank | National | Both | 18+ | 18+ | 106 | 143 |
| 514 | Estonia | 2013-2015 | National Dietary Survey (RTU) 2014 | National | Both | 5-74 | 5-74 | 1,260 | 2,202 |
| 515 | Ethiopia | 2000 | DHS | National | Both |  | 15-49 |  | 13,912 |
| 516 | Ethiopia | 2005 | DHS | National | Both |  | 15-49 |  | 6,133 |
| 517 | Ethiopia | 2006 | STEPS | Subnational | Urban | 25-64 | 25-64 | 1,642 | 2,295 |
| 518 | Ethiopia | 2011 | DHS | National | Both | 15-59 | 15-49 | 14,329 | 15,111 |
| 519 | Ethiopia | 2016 | DHS | National | Both | 15-59 | 15-49 | 12,380 | 14,104 |
| 520 | Fiji | 2002 | STEPS | National | Both | 15-64 | 15-64 | 2,684 | 3,820 |
| 521 | Fiji | 2007-2008 | Pacific Obesity Prevention in Communities – Healthy Youth Health Communities Study | Subnational | Urban | 13-22 | 13-22 | 1,492 | 1,832 |
| 522 | Fiji | 2009 | Fiji Eye Health Survey 2009 | National | Both | 40+ | 40+ | 582 | 776 |
| 523 | Fiji | 2011 | STEPS | National | Both | 25-64 | 25-64 | 1,123 | 1,417 |
| 524 | Finland | 1985-1986 | INTERSALT, Joensuu | Community | Urban | 20-59 | 20-59 | 100 | 100 |
| 525 | Finland | 1985 | INTERSALT, Turku | Community | Urban | 20-59 | 20-59 | 100 | 100 |
| 526 | Finland | 1986 | Young Finns Study 1986 | National | Rural | 9-24 | 9-24 | 594 | 631 |
| 527 | Finland | 1986 | Young Finns Study 1986 | National | Urban | 9-24 | 9-24 | 587 | 666 |
| 528 | Finland | 1987 | MONICA, North Karelia/Kuopio/Turku/Loimaa | Subnational | Both | 25-64 | 25-64 | 2,896 | 3,151 |
| 529 | Finland | 1984-1989 | Kuopio Ischaemic Heart Disease Risk Factor Study | Subnational | Both | 42-61 |  | 2,670 |  |
| 530 | Finland | 1989 | Finnish cohort of the FINE study | Community | Rural | 70-89 |  | 446 |  |
| 531 | Finland | 1990-1992 | Oulu 35 Study | Community | Urban | 56-57 | 56-57 | 231 | 326 |
| 532 | Finland | 1992 | The National FINRISK Study | Subnational | Both | 25-64 | 25-64 | 2,849 | 3,201 |
| 533 | Finland | 1991-1993 | Kuopio Ischaemic Heart Disease Risk Factor Study | Subnational | Both | 46-64 |  | 1,037 |  |
| 534 | Finland | 1994 | Finnish cohort of the FINE study | Community | Rural | 75-94 |  | 266 |  |
| 535 | Finland | 1997 | North Finland Birth Cohort 1966 | Community | Both | 30-31 | 30-31 | 2,770 | 149 |
| 536 | Finland | 1996-1998 | Oulu 35 Study | Community | Urban | 60-63 | 60-63 | 242 | 345 |
| 537 | Finland | 1996-1998 | Savitaipale Study, Baseline | Community | Rural | 40-66 | 40-66 | 574 | 574 |
| 538 | Finland | 1997 | The National FINRISK Study | National | Both | 25-74 | 25-74 | 4,128 | 4,131 |
| 539 | Finland | 1998-2001 | Kuopio Ischaemic Heart Disease Risk Factor Study | Subnational | Both | 53-73 | 53-73 | 854 | 920 |
| 540 | Finland | 2001 | Young Finns Study 2001 | National | Rural | 24-39 | 24-39 | 346 | 393 |
| 541 | Finland | 2001 | Young Finns Study 2001 | National | Urban | 24-39 | 24-39 | 658 | 769 |

| 542 | Finland | 2000-2001 | Health 2000 Survey | National | Both | 30+ | 30+ | 2,656 | 3,213 |
| --- | --- | --- | --- | --- | --- | --- | --- | --- | --- |
| 543 | Finland | 2001-2003 | Oulu 45 Study | Community | Urban | 55-58 | 55-58 | 426 | 550 |
| 544 | Finland | 2002 | The National FINRISK Study | National | Both | 25-74 | 25-74 | 3,299 | 3,826 |
| 545 | Finland | 2001-2004 | Helsinki Birth Cohort Study | Community | Urban | 56-69 | 56-69 | 927 | 1,074 |
| 546 | Finland | 2004-2005 | FIN-D2D | Subnational | Both | 45-74 | 45-74 | 1,364 | 1,461 |
| 547 | Finland | 2005 | Mantyselka et al., Rheumatology (Oxford) 47(8):1235-38, 2008 | Community | Rural | 30-65 | 30-65 | 230 | 241 |
| 548 | Finland | 2007 | Oulu 35 Study | Community | Urban | 71-73 | 71-73 | 182 | 271 |
| 549 | Finland | 2007 | Young Finns Study 2007 | National | Rural | 30-45 | 30-45 | 374 | 431 |
| 550 | Finland | 2007 | Young Finns Study 2007 | National | Urban | 30-45 | 30-45 | 602 | 714 |
| 551 | Finland | 2007 | The National FINRISK Study | National | Both | 25-74 | 25-74 | 2,934 | 3,323 |
| 552 | Finland | 2005-2008 | Kuopio Ischaemic Heart Disease Risk Factor Study | Subnational | Both | 62-82 | 60-82 | 1,241 | 634 |
| 553 | Finland | 2008 | Control group for Finnish male former elite athletes | National | Both | 61+ |  | 206 |  |
| 554 | Finland | 2007-2008 | Savitaipale Study, Follow-up | Community | Rural | 51-75 | 51-75 | 430 | 483 |
| 555 | Finland | 2011 | Young Finns Study 2011 | National | Rural | 34-49 | 34-49 | 364 | 424 |
| 556 | Finland | 2011 | Young Finns Study 2011 | National | Urban | 34-49 | 34-49 | 506 | 636 |
| 557 | Finland | 2012 | North Finland Birth Cohort 1966 | Community | Both | 45-47 | 45-47 | 2,547 | 3,238 |
| 558 | Finland | 2012 | The National FINRISK Study | National | Both | 25-74 | 25-74 | 2,774 | 3,052 |
| 559 | Finland | 2011-2012 | Health 2011 Survey | National | Both | 30+ | 30+ | 2,041 | 2,532 |
| 560 | Finland | 2017 | The FinHealth Survey | National | Both | 18+ | 18+ | 2,699 | 3,168 |
| 561 | France | 1985-1987 | MONICA, Strasbourg | Subnational | Both | 35-64 | 35-64 | 664 | 713 |
| 562 | France | 1985-1987 | MONICA, Strasbourg | Community | Both | 25-34 | 25-34 | 65 | 78 |
| 563 | France | 1985-1987 | MONICA, Toulouse | Subnational | Both | 35-64 | 35-64 | 675 | 644 |
| 564 | France | 1986-1989 | MONICA, Lille | Community | Urban | 25-64 | 25-64 | 878 | 732 |
| 565 | France | 1988-1991 | MONICA, Toulouse | Subnational | Both | 35-64 |  | 586 |  |
| 566 | France | 1994-1996 | MONICA, Toulouse | Subnational | Both | 35-64 | 35-64 | 608 | 566 |
| 567 | France | 1995-1997 | MONICA, Lille | Community | Urban | 36-67 | 36-67 | 598 | 590 |
| 568 | France | 1995-1997 | MONICA, Strasbourg | Subnational | Both | 35-64 | 35-64 | 526 | 523 |
| 569 | France | 1999-2001 | The Three City Study | Community | Urban | 65+ | 65+ | 2,423 | 3,778 |
| 570 | France | 1996-2003 | Jaquet et al., Diabetologia 48(5):849-55, 2005 | Community | Urban | 15-34 | 15-34 | 173 | 164 |
| 571 | France | 2004-2006 | National Monitoring of Arterial Risk in Lille (MONA LISA Lille) | Subnational | Urban | 35-75 | 35-75 | 783 | 795 |
| 572 | France | 2005-2007 | National Monitoring of Arterial Risk in Bas-Rhin (MONA LISA Bas-Rhin) | Subnational | Both | 35-74 | 35-74 | 780 | 787 |
| 573 | France | 2005-2007 | National Monitoring of Arterial Risk in Toulouse (MONA LISA Toulouse) | Subnational | Both | 35-74 | 35-74 | 829 | 796 |
| 574 | France | 2006-2008 | The Three City Study | Community | Urban | 72+ | 72+ | 768 | 1,217 |
| 575 | France | 2006-2007 | Etude Nationale Nutrition Santé | National | Both | 5-74 | 5-74 | 1,582 | 2,223 |
| 576 | France | 2011-2013 | Enquête LIttorale Souffle Air Biologie EnvironnemenT (ELISABET) Dunkerque | Community | Urban | 40-64 | 40-64 | 761 | 812 |
| 577 | France | 2011-2013 | Enquête LIttorale Souffle Air Biologie EnvironnemenT (ELISABET) Lille | Community | Urban | 40-64 | 40-64 | 758 | 857 |
| 578 | France | 2012-2014 | Cohorte des consultants des Centres d’examens de santé (CONSTANCES) | National | Both | 18-69 | 18-69 | 22,367 | 25,127 |
| 579 | France | 2014-2016 | Esteban | National | Both | 6-74 | 6-74 | 1,662 | 1,913 |
| 580 | France | 2015-2017 | Cohorte des consultants des Centres d’examens de santé (CONSTANCES) | National | Both | 18-69 | 18-69 | 43,472 | 48,739 |
| 581 | France | 2018-2019 | Cohorte des consultants des Centres d’examens de santé (CONSTANCES) | National | Both | 18-69 | 18-69 | 19,614 | 22,539 |
| 582 | French Polynesia | 2010 | STEPS | National | Both | 18-64 | 18-64 | 1,458 | 1,916 |
| 583 | Gabon | 2000 | DHS | National | Both |  | 20-49 |  | 2,082 |
| 584 | Gabon | 2009 | STEPS | Subnational | Urban | 15-64 | 15-64 | 1,051 | 1,515 |
| 585 | Gabon | 2012 | DHS | National | Both |  | 15-49 |  | 5,066 |
| 586 | Gambia | 1996-1997 | National Survey of Blindness and Low Vision | National | Both | 16+ | 16+ | 1,733 | 2,071 |
| 587 | Gambia | 2003 | Siervo et al., Eur J Clin Nutr 60(4):455-63, 2006 | Community | Urban | 14-50 | 14-50 | 50 | 50 |
| 588 | Gambia | 2010 | STEPS | National | Both | 25-64 | 25-64 | 1,610 | 1,919 |
| 589 | Gambia | 2013 | DHS | National | Both |  | 15-49 |  | 4,180 |
| 590 | Georgia | 2010 | STEPS | National | Both | 18-64 | 18-64 | 1,842 | 4,460 |
| 591 | Georgia | 2016 | STEPS | National | Both | 18-69 | 18-69 | 1,188 | 2,784 |
| 592 | Germany | 1985-1986 | INTERSALT, Cottbus | Community | Urban | 20-59 | 20-59 | 99 | 99 |
| 593 | Germany | 1985-1986 | INTERSALT, Heidelberg | Community | Urban | 20-59 | 20-59 | 97 | 99 |
| 594 | Germany | 1984-1985 | MONICA, Augsburg | Community | Both | 25-64 | 25-64 | 2,005 | 1,961 |
| 595 | Germany | 1984-1986 | MONICA, Cottbus County | Community | Urban | 25-64 | 25-64 | 657 | 739 |
| 596 | Germany | 1983-1987 | MONICA, Rhein-Neckar Region | Community | Urban | 25-64 | 25-64 | 1,489 | 1,609 |
| 597 | Germany | 1985-1986 | INTERSALT, Bernried | Community | Urban | 20-59 | 20-59 | 99 | 98 |
| 598 | Germany | 1985-1986 | CINDI | Subnational | Both | 25-64 | 25-64 | 1,875 | 1,990 |
| 599 | Germany | 1987-1988 | MONICA, Erfurt | Community | Urban | 25-64 | 25-64 | 871 | 909 |
| 600 | Germany | 1988 | MONICA, Berlin-Lichtenberg | Community | Urban | 25-64 | 25-64 | 690 | 728 |
| 601 | Germany | 1988 | MONICA, Bremen North/West | Community | Urban | 25-69 | 25-69 | 619 | 632 |
| 602 | Germany | 1988 | MONICA, Bremen Center/South/East | Community | Urban | 25-69 | 25-69 | 499 | 582 |
| 603 | Germany | 1988 | MONICA, Chemnitz | Community | Urban | 25-64 | 25-64 | 288 | 382 |
| 604 | Germany | 1988 | MONICA, Zwickau | Community | Urban | 25-64 | 25-64 | 193 | 250 |
| 605 | Germany | 1988 | German Cardiovascular Prevention Study (GCP) - National Health Survey 1988 | Subnational | Both | 25-69 | 25-69 | 2,642 | 2,678 |
| 606 | Germany | 1989-1990 | MONICA, Cottbus County | Community | Urban | 25-64 | 25-64 | 539 | 529 |
| 607 | Germany | 1988-1989 | MONICA, Halle County | Subnational | Urban | 25-64 | 25-64 | 959 | 1,201 |
| 608 | Germany | 1988-1989 | MONICA, Rest of Karl-Marx-Stadt County | Subnational | Urban | 25-64 | 25-64 | 541 | 626 |
| 609 | Germany | 1988-1989 | CINDI | Subnational | Both | 25-64 | 25-64 | 1,361 | 1,435 |

| 610 | Germany | 1989-1990 | MONICA, Augsburg | Community | Both | 25-64 | 25-64 | 1,933 | 1,944 |
| --- | --- | --- | --- | --- | --- | --- | --- | --- | --- |
| 611 | Germany | 1991-1992 | MONICA, Bremen North/West | Community | Urban | 25-69 | 25-69 | 599 | 671 |
| 612 | Germany | 1991-1992 | MONICA, Bremen Center/South/East | Community | Urban | 25-69 | 25-69 | 524 | 546 |
| 613 | Germany | 1990-1992 | European Community Respiratory Health Survey, Hamburg | Community | Urban | 20-47 | 20-47 | 146 | 138 |
| 614 | Germany | 1990-1992 | European Community Respiratory Health Survey, Erfurt | Community | Urban | 20-47 | 20-47 | 146 | 124 |
| 615 | Germany | 1991-1992 | MONICA, Erfurt | Community | Urban | 25-64 | 25-64 | 587 | 572 |
| 616 | Germany | 1991-1992 | CINDI | Subnational | Both | 25-64 | 25-64 | 1,326 | 1,400 |
| 617 | Germany | 1991-1992 | German Cardiovascular Prevention Study (GCP) - National Health Survey 1991 | Subnational | Both | 25-69 | 25-69 | 2,599 | 2,670 |
| 618 | Germany | 1991-1992 | First National Examination of Life Conditions, Environment and Health in East Germany 1991/92 | Subnational | Both | 25-69 | 25-69 | 1,042 | 1,155 |
| 619 | Germany | 1993-1994 | MONICA, Chemnitz | Community | Urban | 25-64 | 25-64 | 408 | 424 |
| 620 | Germany | 1993-1994 | MONICA, Zwickau | Community | Urban | 25-64 | 25-64 | 139 | 186 |
| 621 | Germany | 1994-1995 | MONICA, Augsburg | Community | Both | 25-64 | 25-64 | 1,898 | 1,968 |
| 622 | Germany | 1994-1998 | EPIC Heidelberg | Community | Urban | 40-64 | 35-64 | 11,680 | 13,458 |
| 623 | Germany | 1994-1998 | EPIC Potsdam | Community | Urban | 40-64 | 35-64 | 10,224 | 15,995 |
| 624 | Germany | 1997-1999 | German National Health Interview and Examination Survey (GNHIES98) | National | Both | 18-79 | 18-79 | 3,435 | 3,608 |
| 625 | Germany | 1997-2001 | Study of Health in Pomerania (SHIP-0) baseline study | Subnational | Both | 20-80 | 20-80 | 2,111 | 2,187 |
| 626 | Germany | 1999-2001 | KORA S4 Study: Kooperative Research in the Region of Augsburg Survey 4 | Community | Both | 24-75 | 24-75 | 2,076 | 2,148 |
| 627 | Germany | 2000-2002 | Epidemiological study of the chances of prevention, early recognition and optimal treatment of chronic diseases in an elderly population (ESTHER) | Subnational | Both | 50-75 | 50-75 | 4,344 | 5,334 |
| 628 | Germany | 2000-2001 | European Community Respiratory Health Survey, Hamburg | Community | Urban | 30-57 | 30-57 | 146 | 138 |
| 629 | Germany | 2000-2001 | European Community Respiratory Health Survey, Erfurt | Community | Urban | 30-57 | 30-57 | 146 | 124 |
| 630 | Germany | 2000-2003 | Heinz Nixdorf Recall Study | Subnational | Urban | 45-75 | 45-75 | 2,380 | 2,401 |
| 631 | Germany | 2000-2003 | Heinz Nixdorf Recall Study | Community | Urban | 45-74 | 45-74 | 2,375 | 2,393 |
| 632 | Germany | 2002 | Echinoccoccus Multilocularis and Internal Diseases in Leutkirch | Community | Urban | 12-65 | 12-65 | 1,171 | 1,261 |
| 633 | Germany | 2002-2006 | Study of Health in Pomerania (SHIP-1) 5-year follow-up | Subnational | Both | 25-85 | 25-85 | 1,583 | 1,707 |
| 634 | Germany | 2005-2008 | Heinz Nixdorf Recall Study | Subnational | Both | 50-80 | 50-80 | 2,044 | 2,099 |
| 635 | Germany | 2006-2008 | KORA F4 Study: Kooperative Research in the Region of Augsburg Follow-Up of Survey 4 | Community | Both | 31-81 | 31-81 | 1,480 | 1,583 |
| 636 | Germany | 2008-2011 | Epidemiological study of the chances of prevention, early recognition and optimal treatment of chronic diseases in an elderly population (ESTHER) | Subnational | Both | 58-84 | 58-84 | 1,468 | 1,622 |
| 637 | Germany | 2008-2012 | Study of Health in Pomerania, second cohort (SHIP-TREND) | Subnational | Both | 20-79 | 20-79 | 2,099 | 2,232 |
| 638 | Germany | 2008-2011 | German Health Interview and Examination Survey for adults 2008-11 (DEGS1) | National | Both | 18-79 | 18-79 | 3,389 | 3,650 |
| 639 | Germany | 2011-2014 | Heinz Nixdorf Recall Study | Subnational | Both | 56-85 | 56-85 | 1,493 | 1,560 |
| 640 | Ghana | 1993 | DHS | National | Both |  | 20-49 |  | 1,650 |
| 641 | Ghana | 1997 | Amoah et al., Ethn Dis 13(2 Suppl 2):S97-101, 2003 | Community | Both | 25+ | 25+ | 1,857 | 2,875 |
| 642 | Ghana | 1998 | DHS | National | Both |  | 20-49 |  | 1,979 |
| 643 | Ghana | 2001 | Addo et al., Ethn Dis 16(4):894-99, 2006 | Community | Rural | 15+ | 15+ | 89 | 206 |
| 644 | Ghana | 2002 | Amoah et al., Ethn Dis 13(2 Suppl 2):S97-101, 2003 | Community | Both | 25+ | 25+ | 1,859 | 2,947 |
| 645 | Ghana | 2001-2002 | Cappuccio et al., Hypertension 43(5):1017-22, 2004 | Community | Both | 35-84 | 35-84 | 194 | 338 |
| 646 | Ghana | 2003 | DHS | National | Both |  | 15-49 |  | 4,935 |
| 647 | Ghana | 2003 | Women's Health Study of Accra (WHSA-I) | Community | Urban |  | 18+ |  | 1,184 |
| 648 | Ghana | 2006 | STEPS | Community | Urban | 25+ | 25+ | 841 | 1,635 |
| 649 | Ghana | 2008 | DHS | National | Both |  | 15-49 |  | 4,455 |
| 650 | Ghana | 2007-2008 | SAGE | National | Both | 50+ | 50+ | 2,192 | 1,987 |
| 651 | Ghana | 2008-2010 | Women's Health Study of Accra (WHSA-II) | Community | Urban |  | 18+ |  | 2,677 |
| 652 | Ghana | 2012-2014 | Research on Obesity and Diabetes among African Migrants (RODAM), control group | Subnational | Rural | 25+ | 25+ | 431 | 676 |
| 653 | Ghana | 2012-2014 | Research on Obesity and Diabetes among African Migrants (RODAM), control group | Subnational | Urban | 25+ | 25+ | 418 | 1,034 |
| 654 | Ghana | 2014 | DHS | National | Both | 15-59 | 15-49 | 4,416 | 4,486 |
| 655 | Greece | 1991-1999 | EPIC | National | Both | 19-86 | 19-86 | 11,578 | 16,477 |
| 656 | Greece | 1997 | The Didima Study | Community | Rural | 18+ | 18+ | 265 | 373 |
| 657 | Greece | 2000-2001 | Karalis et al., BMC Public Health7:351, 2007 | Community | Rural | 5+ | 5+ | 73 | 87 |
| 658 | Greece | 2013-2015 | Hellenic National Nutrition and Health Survey (HNNHS) | National | Both | 5+ | 5+ | 1,781 | 2,420 |
| 659 | Greece | 2013-2016 | National Survey of Morbidity and Risk Factors | National | Both | 18+ | 18+ | 2,039 | 2,726 |
| 660 | Greece | 2016 | SKG-Elderly | Community | Urban | 60+ | 60+ | 51 | 63 |
| 661 | Greece | 2018 | STEPS-Thessaloniki | Community | Both | 60+ | 60+ | 353 | 141 |
| 662 | Greenland | 2005-2010 | Population Health Survey in Greenland | National | Both | 18+ | 18+ | 1,336 | 1,714 |
| 663 | Grenada | 2011 | STEPS | National | Both | 25-64 | 25-64 | 438 | 637 |
| 664 | Guatemala | 1995 | DHS | National | Both |  | 20-49 |  | 4,547 |
| 665 | Guatemala | 1998-1999 | DHS | National | Both |  | 20-49 |  | 2,172 |
| 666 | Guatemala | 2001-2002 | CAMDI | Community | Urban | 20+ | 20+ | 293 | 638 |
| 667 | Guatemala | 2002 | Reproducive Health Survey | National | Both | 15-59 | 15-49 | 2,164 | 7,374 |
| 668 | Guatemala | 2003-2005 | The Institute of Nutrition of Central America and Panama Nutrition Supplementation Trial Cohort | Community | Both | 25-41 | 25-41 | 268 | 288 |
| 669 | Guatemala | 2008-2009 | Encuesta Nacional de Salud Materno Infantil | National | Both | 15-59 | 15-49 | 6,636 | 15,271 |
| 670 | Guatemala | 2014-2015 | DHS | National | Both |  | 15-49 |  | 24,195 |
| 671 | Guinea | 1999 | DHS | National | Both |  | 20-49 |  | 2,984 |
| 672 | Guinea | 2005 | DHS | National | Both |  | 15-49 |  | 3,574 |
| 673 | Guinea | 2009 | STEPS | Subnational | Both | 15-64 | 15-64 | 1,124 | 1,232 |
| 674 | Guinea | 2012 | DHS | National | Both |  | 15-49 |  | 4,229 |
| 675 | Guinea | 2018 | DHS | National | Both |  | 15-49 |  | 4,905 |
| 676 | Guinea Bissau | 2010 | Multiple Indicator Cluster Survey | National | Both |  | 15-49 |  | 7,676 |
| 677 | Guyana | 2009 | DHS | National | Both | 15-49 | 15-49 | 3,412 | 4,575 |

| 678 | Guyana | 2016 | STEPS | National | Both | 18-69 | 18-69 | 1,060 | 1,571 |
| --- | --- | --- | --- | --- | --- | --- | --- | --- | --- |
| 679 | Haiti | 1994-1995 | DHS | National | Both |  | 20-49 |  | 1,788 |
| 680 | Haiti | 2000 | DHS | National | Both |  | 15-49 |  | 9,163 |
| 681 | Haiti | 2005-2006 | DHS | National | Both |  | 15-49 |  | 5,011 |
| 682 | Haiti | 2012 | DHS | National | Both |  | 15-49 |  | 8,993 |
| 683 | Haiti | 2015-2016 | Carrefour | Community | Urban | 25-65 | 25-65 | 557 | 835 |
| 684 | Haiti | 2015-2016 | Thomonde | Community | Rural | 25-65 | 25-65 | 254 | 441 |
| 685 | Haiti | 2016-2017 | DHS | National | Both |  | 15-49 |  | 9,049 |
| 686 | Honduras | 1996 | Honduras National Micronutrient Survey | National | Both |  | 20-40 |  | 722 |
| 687 | Honduras | 2003-2004 | CAMDI | Community | Urban | 20+ | 20+ | 428 | 764 |
| 688 | Honduras | 2005-2006 | DHS | National | Both |  | 15-49 |  | 18,125 |
| 689 | Honduras | 2011-2012 | DHS | National | Both |  | 15-49 |  | 21,097 |
| 690 | Hungary | 1985 | INTERSALT | Community | Rural | 20-59 | 20-59 | 100 | 100 |
| 691 | Hungary | 1985-1988 | First Hungarian Representative Nutrition Survey | National | Both | 15+ | 15+ | 3,079 | 8,916 |
| 692 | Hungary | 1987-1988 | MONICA, Budapest | Community | Urban | 25-64 | 25-64 | 1,413 | 1,594 |
| 693 | Hungary | 1987-1988 | MONICA, Pecs | Community | Urban | 25-64 | 25-64 | 1,573 | 1,510 |
| 694 | Hungary | 2003 | The European Male Ageing Study | Community | Both | 40+ |  | 428 |  |
| 695 | Hungary | 2008 | The European Male Ageing Study | Community | Both | 40+ |  | 349 |  |
| 696 | Iceland | 1985-1986 | INTERSALT | Community | Urban | 20-59 | 20-59 | 100 | 100 |
| 697 | Iceland | 1985-1987 | The Reykjavik Study (Men) | Subnational | Urban | 51-79 |  | 2,584 |  |
| 698 | Iceland | 1988-1989 | MONICA, Arnes County | Community | Rural | 25-64 | 25-64 | 385 | 435 |
| 699 | Iceland | 1988-1989 | MONICA, Reykjavik | Subnational | Urban | 25-64 | 25-64 | 414 | 443 |
| 700 | Iceland | 1987-1991 | The Reykjavik Study (Women) | Subnational | Urban |  | 52-82 |  | 2,993 |
| 701 | Iceland | 1993-1994 | MONICA, Arnes County | Community | Rural | 25-64 | 25-64 | 422 | 484 |
| 702 | Iceland | 1993-1994 | MONICA, Reykjavik | Subnational | Urban | 25-64 | 25-64 | 441 | 448 |
| 703 | Iceland | 1991-1994 | The Reykjavik Study (Men) | Subnational | Urban | 70-86 |  | 797 |  |
| 704 | Iceland | 1994-1996 | The Reykjavik Study (Women) | Subnational | Urban |  | 69-88 |  | 1,101 |
| 705 | Iceland | 2001-2003 | The Reykjavik Study for the young | Subnational | Urban | 47-62 | 47-62 | 626 | 705 |
| 706 | Iceland | 2002-2006 | AGES-Reykjavik Study | Subnational | Urban | 66-96 | 66-96 | 2,413 | 3,272 |
| 707 | Iceland | 2005-2011 | Risk Evaluation For INfarct Estimates (REFINE) | Subnational | Urban | 20-73 | 20-73 | 3,402 | 3,525 |
| 708 | Iceland | 2007-2011 | AGES-Reykjavik Study - follow up visit | Subnational | Urban | 71-98 | 71-98 | 1,389 | 1,928 |
| 709 | Iceland | 2010-2012 | Risk Evaluation For INfarct Estimates (REFINE) follow-up visit (REFINELO) | Subnational | Urban | 26-74 | 26-74 | 653 | 667 |
| 710 | Iceland | 2012-2013 | Risk Evaluation For INfarct Estimates (REFINE) - follow-up visit (REFLOCT) | Subnational | Urban | 55-73 | 55-73 | 516 | 561 |
| 711 | India | 1986 | INTERSALT | Community | Urban | 20-59 | 20-59 | 100 | 99 |
| 712 | India | 1988-1989 | Rmachandran et al., Diabetes Res Clin Pract 58(1):55-60, 2002 | Community | Urban | 20-74 | 20-74 | 455 | 437 |
| 713 | India | 1990 | National Nutrition Monitoring Bureau Rural Survey | National | Rural | 5+ | 5+ | 7,607 | 9,649 |
| 714 | India | 1992-1994 | Jaipur Heart Watch 1 | Community | Rural | 20-80 | 20-80 | 1,946 | 1,147 |
| 715 | India | 1992-1994 | Jaipur Heart Watch 1 | Community | Urban | 20-80 | 20-80 | 1,385 | 782 |
| 716 | India | 1991-1995 | Reddy et al., Obes Rev 3(3):197-202, 2002 | Community | Rural | 35-64 | 35-64 | 1,070 | 1,332 |
| 717 | India | 1991-1995 | Reddy et al., Obes Rev 3(3):197-202, 2002 | Community | Urban | 35-64 | 35-64 | 1,456 | 1,594 |
| 718 | India | 1991-1994 | Prabhakaran et al., Chronic Illn 3(1):8-19, 2007 | Community | Rural | 35-64 | 35-64 | 542 | 630 |
| 719 | India | 1991-1994 | Prabhakaran et al., Chronic Illn 3(1):8-19, 2007 | Community | Urban | 35-64 | 35-64 | 1,388 | 1,455 |
| 720 | India | 1993-1994 | Khongsdier, Eur J Clin Nutr 56(6):484-89, 2002 | Community | Both | 18-59 |  | 575 |  |
| 721 | India | 1991-1997 | Mumbai Cohort Study | Community | Urban | 35+ | 35+ | 88,658 | 59,515 |
| 722 | India | 1995 | Shobana et al., Diabetes Res Clin Pract 42(3):181–86, 1998 | Community | Urban | 20-74 | 20-74 | 1,061 | 1,093 |
| 723 | India | 1995-1996 | Kusuma et al., Ann Hum Biol 29(5):502-12, 2002 | Community | Both | 15-84 | 15-84 | 747 | 737 |
| 724 | India | 1995-1997 | Aravind Comprehensive Eye Survey | Community | Rural | 40+ | 40+ | 2,308 | 2,830 |
| 725 | India | 1995-1996 | Epidemiology of blood pressure across cross-cultural populations of Visakhapatnam district, Andhra Pradesh, India | Community | Rural | 19-76 | 19-76 | 209 | 228 |
| 726 | India | 1995-1997 | Kashmiri Adults | Subnational | Both | 40+ | 40+ | 2,496 | 2,587 |
| 727 | India | 1997 | Ramachandran et al., Diabetes Res Clin Pract 44(3):207–13, 1999 | Community | Rural | 20-74 | 20-74 | 738 | 879 |
| 728 | India | 1996-1997 | National Nutrition Monitoring Bureau Rural Survey | National | Rural | 5+ | 5+ | 22,155 | 27,802 |
| 729 | India | 1996-1999 | Chennai Urban Population Study | Community | Urban | 20+ | 20+ | 557 | 705 |
| 730 | India | 1998-1999 | DHS | National | Both |  | 20-49 |  | 72,536 |
| 731 | India | 2000 | Ramachandran et al., Diabet Med, 20(3):220-24, 2003 | Subnational | Urban | 20-75 | 20-75 | 4,640 | 5,257 |
| 732 | India | 1999-2001 | Jaipur Heart Watch 2 | Community | Urban | 20-75 | 20-75 | 534 | 569 |
| 733 | India | 1998-2002 | Vellore Birth Cohort | Subnational | Both | 25-31 | 25-31 | 1,160 | 1,050 |
| 734 | India | 1998-2001 | Chennai Prospective Study | Community | Urban | 35+ | 35+ | 264,848 | 235,968 |
| 735 | India | 1999-2002 | New Delhi Birth Cohort | Community | Urban | 26-33 | 26-33 | 886 | 638 |
| 736 | India | 2000-2001 | National Nutrition Monitoring Bureau Rural Survey | National | Rural | 5+ | 5+ | 18,046 | 24,844 |
| 737 | India | 2001-2004 | Chennai Urban Rural Epidemiology Study | Community | Urban | 20+ | 20+ | 1,094 | 1,254 |
| 738 | India | 2002-2003 | JHW-3 | Community | Urban | 20-59 | 20-59 | 179 | 195 |
| 739 | India | 2002-2003 | Blood pressure epidemiology in tribal, rural and urban communities of Orissa with special reference to physical and social parameters | Community | Rural | 18-80 | 18-80 | 200 | 186 |
| 740 | India | 2003-2005 | India STEPS Ballabgarh | Subnational | Rural | 15-69 | 15-69 | 1,360 | 1,468 |
| 741 | India | 2003-2005 | India STEPS Ballabgarh | Subnational | Urban | 15-69 | 15-69 | 1,263 | 1,294 |
| 742 | India | 2003-2005 | India STEPS Chennai | Subnational | Rural | 15-69 | 15-69 | 1,372 | 1,338 |
| 743 | India | 2003-2005 | India STEPS Chennai | Subnational | Urban | 15-69 | 15-69 | 1,282 | 1,282 |
| 744 | India | 2003-2005 | India STEPS Delhi | Subnational | Urban | 15-69 | 15-69 | 1,250 | 1,265 |
| 745 | India | 2003-2005 | India STEPS Dibrugarh | Subnational | Rural | 15-69 | 15-69 | 1,460 | 1,410 |

| 746 | India | 2003-2005 | India STEPS Dibrugarh | Subnational | Urban | 15-69 | 15-69 | 1,243 | 1,254 |
| --- | --- | --- | --- | --- | --- | --- | --- | --- | --- |
| 747 | India | 2003-2005 | India STEPS Nagpur | Subnational | Rural | 15-69 | 15-69 | 1,252 | 1,256 |
| 748 | India | 2003-2005 | India STEPS Nagpur | Subnational | Urban | 15-69 | 15-69 | 1,252 | 1,261 |
| 749 | India | 2003-2005 | India STEPS Trivandrum | Subnational | Rural | 15-69 | 15-69 | 1,199 | 1,324 |
| 750 | India | 2003-2005 | India STEPS Trivandrum | Subnational | Urban | 15-69 | 15-69 | 1,250 | 1,252 |
| 751 | India | 2004-2005 | JHW-4 | Community | Urban | 20-59 | 20-59 | 413 | 473 |
| 752 | India | 2005-2006 | DHS | National | Both | 15-54 | 15-49 | 71,463 | 115,828 |
| 753 | India | 2006 | Ramachandran et al., Diabetes Care 31(5):893-98, 2008 | Community | Both | 20+ | 20+ | 3,321 | 3,745 |
| 754 | India | 2005-2006 | Risk factor profile for chronic non-communicable diseases: Results of a community-based study in Kerala, India | Community | Both | 15-64 | 15-64 | 2,795 | 2,926 |
| 755 | India | 2005-2006 | National Nutrition Monitoring Bureau Rural Survey | National | Rural | 5+ | 5+ | 20,448 | 25,272 |
| 756 | India | 2005-2007 | Prevalence of cardiovascular risk factors in rural Tamil Nadu | Community | Rural | 25-65 | 25-65 | 4,927 | 5,573 |
| 757 | India | 2006-2008 | Central India Eye and Medical Study | Community | Rural | 30+ | 30+ | 2,190 | 2,518 |
| 758 | India | 2006-2007 | Kusuma et al., Asia Pac J Public Health 21(4):497-507, 2009 | Community | Urban | 15-74 | 15-74 | 182 | 192 |
| 759 | India | 2006-2008 | Kashmiri Young Adults | Subnational | Both | 20-40 | 20-40 | 2,119 | 905 |
| 760 | India | 2005-2011 | Bengali School Children | Community | Urban | 7-21 | 7-21 | 847 | 2,180 |
| 761 | India | 2006-2009 | New Delhi Birth Cohort | Community | Urban | 33-38 | 33-38 | 650 | 445 |
| 762 | India | 2007-2008 | SAGE | National | Both | 50+ | 50+ | 3,213 | 3,147 |
| 763 | India | 2007-2008 | Integrated Disease Surveillance Project Non-communicable Disease Risk Factors Survey Andhra Pradesh | Subnational | Both | 15-64 | 15-64 | 2,674 | 3,390 |
| 764 | India | 2007-2008 | Integrated Disease Surveillance Project Non-communicable Disease Risk Factors Survey Kerala | Subnational | Both | 15-64 | 15-64 | 1,672 | 2,403 |
| 765 | India | 2007-2008 | Integrated Disease Surveillance Project Non-communicable Disease Risk Factors Survey Madhya Pradesh | Subnational | Both | 15-64 | 15-64 | 2,797 | 2,862 |
| 766 | India | 2007-2008 | Integrated Disease Surveillance Project Non-communicable Disease Risk Factors Survey Maharashtra | Subnational | Both | 15-64 | 15-64 | 3,025 | 2,921 |
| 767 | India | 2007-2008 | Integrated Disease Surveillance Project Non-communicable Disease Risk Factors Survey Mizoram | Subnational | Both | 15-64 | 15-64 | 2,232 | 2,108 |
| 768 | India | 2007-2008 | Integrated Disease Surveillance Project Non-communicable Disease Risk Factors Survey Tamil Nadu | Subnational | Both | 15-64 | 15-64 | 2,039 | 2,928 |
| 769 | India | 2007-2008 | Integrated Disease Surveillance Project Non-communicable Disease Risk Factors Survey Uttrakhand | Subnational | Both | 15-64 | 15-64 | 2,094 | 3,110 |
| 770 | India | 2007-2009 | Prevalence of NCD risk factor in people above 15 year in rural area Nagpur using WHO STEP approach | Community | Rural | 15+ | 15+ | 1,984 | 1,828 |
| 771 | India | 2008-2010 | ICMR India Diabetes Study | National | Both | 20+ | 20+ | 6,953 | 6,889 |
| 772 | India | 2010 | Kerala 2010 follow-up | Community | Rural | 21-70 | 21-70 | 214 | 237 |
| 773 | India | 2009-2010 | Baseline Survey for the assessment of prevalence of risk factors of NCDs in Gandhinagar District | Community | Rural | 15-64 | 15-64 | 875 | 774 |
| 774 | India | 2009-2010 | Baseline Survey for the assessment of prevalence of risk factors of NCDs in Gandhinagar District | Community | Urban | 15-64 | 15-64 | 895 | 890 |
| 775 | India | 2010-2011 | Longitudinal Aging Study in India | Subnational | Both | 45+ | 45+ | 630 | 678 |
| 776 | India | 2011-2012 | National Nutrition Monitoring Bureau Rural Survey | National | Rural | 5+ | 5+ | 34,447 | 43,063 |
| 777 | India | 2012-2013 | District Level Household and Facility Survey (DLHS) 4 | National | Both | 5+ | 5+ | 538,808 | 602,926 |
| 778 | India | 2012-2013 | ICMR-India Diabetes (INDIAB) Study, Phase II | Subnational | Both | 20+ | 20+ | 8,165 | 10,755 |
| 779 | India | 2014 | Annual Health Survey-Chemical, Anthropometric | National | Both | 5+ | 5+ | 658,441 | 680,611 |
| 780 | India | 2013-2014 | Vellore Birth Cohort | Subnational | Both | 39-44 | 39-44 | 580 | 499 |
| 781 | India | 2012-2015 | ICMR-India Diabetes (INDIAB) Study, North East Phase | National | Both | 20+ | 20+ | 10,334 | 12,649 |
| 782 | India | 2015-2016 | DHS | National | Both | 15-54 | 15-49 | 108,751 | 655,681 |
| 783 | India | 2015-2016 | Diet and nutritional status of urban population and prevalence of hypertension | National | Urban | 5+ | 5+ | 68,893 | 81,999 |
| 784 | India | 2016-2019 | Vellore Birth Cohort | Subnational | Both | 43-48 | 43-48 | 843 | 758 |
| 785 | Indonesia | 1983-1987 | Strickland et al., Eur J Clin Nutr 48 Suppl 3: S98-108; discussion S-9, 1994 | Community | Both | 18+ | 18+ | 447 | 564 |
| 786 | Indonesia | 1993-1994 | Indonesian Family Life Surveys | National | Both | 5+ | 5+ | 8,889 | 10,205 |
| 787 | Indonesia | 1997-1998 | Indonesian Family Life Surveys | National | Both | 5+ | 5+ | 12,154 | 13,897 |
| 788 | Indonesia | 2000-2001 | Indonesian Family Life Surveys | National | Both | 5+ | 5+ | 15,442 | 16,225 |
| 789 | Indonesia | 2001 | Ng et al., Bull World Health Organ 84(4):305-13, 2006 | Community | Both | 15-74 | 15-74 | 1,261 | 1,234 |
| 790 | Indonesia | 2001 | STEPS/SURKESNAS | National | Both | 15-64 | 15-64 | 4,100 | 4,775 |
| 791 | Indonesia | 2003 | A genetic-ecological study of the risk foctors for lifestyle-related diseases in Oceanian populations, Study A | Community | Rural | 18-79 | 18-79 | 99 | 103 |
| 792 | Indonesia | 2003 | A genetic-ecological study of the risk foctors for lifestyle-related diseases in Oceanian populations, Study B | Community | Rural | 18-79 | 18-79 | 100 | 140 |
| 793 | Indonesia | 2006 | Jakarta Non Communicable Disease Risk Factor Surveillance | Community | Urban | 25-64 | 25-64 | 641 | 950 |
| 794 | Indonesia | 2007-2008 | Indonesian Family Life Surveys | National | Both | 5+ | 5+ | 17,869 | 19,157 |
| 795 | Indonesia | 2013 | Population Health Basic Health Research 2013 (Riskesdas 2013) | National | Both | 20+ | 20+ | 299,439 | 328,085 |
| 796 | Indonesia | 2014-2015 | Indonesian Family Life Surveys | National | Both | 5+ | 5+ | 20,328 | 21,540 |
| 797 | Iran | 1997-1998 | Khadivzadeh, East Mediterr Health J 8(4-5):612-18, 2002 | Community | Urban |  | 15-49 |  | 1,513 |
| 798 | Iran | 2000 | Asadabadi Study | Community | Urban | 18+ | 18+ | 132 | 168 |
| 799 | Iran | 1999-2000 | National Health Survey II | National | Both | 5+ | 5+ | 23,727 | 26,636 |
| 800 | Iran | 1999-2001 | Tehran Lipid and Glucose Study | Community | Urban | 5+ | 5+ | 6,339 | 7,988 |
| 801 | Iran | 2001 | Isfahan Healthy Heart Program, Arak rural | Community | Rural | 19+ | 19+ | 1,023 | 1,080 |
| 802 | Iran | 2001 | Isfahan Healthy Heart Program, Arak urban | Community | Urban | 19+ | 19+ | 2,084 | 2,124 |
| 803 | Iran | 2001 | Isfahan Healthy Heart Program, Isfahan rural | Community | Rural | 19+ | 19+ | 232 | 233 |
| 804 | Iran | 2001 | Isfahan Healthy Heart Program, Isfahan urban | Community | Urban | 19+ | 19+ | 1,760 | 1,912 |
| 805 | Iran | 2001 | Isfahan Healthy Heart Program, Najaf Abad rural | Community | Rural | 19+ | 19+ | 405 | 416 |
| 806 | Iran | 2001 | Isfahan Healthy Heart Program, Najaf Abad urban | Community | Urban | 19+ | 19+ | 573 | 571 |
| 807 | Iran | 2003-2004 | The Persian Gulf Healthy Heart Study | Subnational | Urban | 25-75 | 25-75 | 1,736 | 1,973 |
| 808 | Iran | 2004 | Hajian-Tilaki et al., Obes Rev 8(1):3-10, 2007 | Community | Urban | 20-70 | 20-70 | 1,800 | 1,800 |
| 809 | Iran | 2002-2005 | Tehran Lipid and Glucose Study | Community | Urban | 5+ | 5+ | 2,736 | 3,388 |
| 810 | Iran | 2005 | Dastgiri et al., J Public Health Nutr 2006; 9: 996-1000 | Subnational | Urban | 15-70 | 15-70 | 130 | 167 |
| 811 | Iran | 2005 | STEPS | National | Both | 15-64 | 15-64 | 40,722 | 39,748 |
| 812 | Iran | 2004-2008 | Golestan Cohort Study Main Phase | Subnational | Rural | 40-75 | 40-75 | 17,298 | 22,708 |
| 813 | Iran | 2004-2008 | Golestan Cohort Study Main Phase | Community | Urban | 40-75 | 40-75 | 3,931 | 6,100 |

| 814 | Iran | 2006 | STEPS | National | Both | 16-65 | 16-65 | 14,885 | 14,617 |
| --- | --- | --- | --- | --- | --- | --- | --- | --- | --- |
| 815 | Iran | 2005-2006 | Rashidy-Pour, Obes Rev (1):2-6, 2009 | Subnational | Both | 30-70 | 30-70 | 1,695 | 2,104 |
| 816 | Iran | 2007 | STEPS | National | Both | 15-64 | 15-64 | 2,372 | 2,312 |
| 817 | Iran | 2007 | STEPS | National | Both | 15-64 | 15-64 | 14,867 | 14,550 |
| 818 | Iran | 2007 | Isfahan Healthy Heart Program, Arak rural | Community | Rural | 19+ | 19+ | 1,028 | 1,024 |
| 819 | Iran | 2007 | Isfahan Healthy Heart Program, Arak urban | Community | Urban | 19+ | 19+ | 1,424 | 1,359 |
| 820 | Iran | 2007 | Isfahan Healthy Heart Program, Isfahan rural | Community | Rural | 19+ | 19+ | 155 | 151 |
| 821 | Iran | 2007 | Isfahan Healthy Heart Program, Isfahan urban | Community | Urban | 19+ | 19+ | 1,309 | 1,302 |
| 822 | Iran | 2007 | Isfahan Healthy Heart Program, Najaf Abad rural | Community | Rural | 19+ | 19+ | 254 | 253 |
| 823 | Iran | 2007 | Isfahan Healthy Heart Program, Najaf Abad urban | Community | Urban | 19+ | 19+ | 494 | 542 |
| 824 | Iran | 2005-2008 | Tehran Lipid and Glucose Study | Community | Urban | 5+ | 5+ | 3,104 | 3,808 |
| 825 | Iran | 2008 | STEPS | National | Both | 15-64 | 15-64 | 14,757 | 14,353 |
| 826 | Iran | 2009 | STEPS | National | Both | 15-64 | 15-64 | 14,834 | 14,495 |
| 827 | Iran | 2009-2010 | The Persian Gulf Healthy Heart Study | Subnational | Urban | 31-79 | 31-79 | 834 | 1,016 |
| 828 | Iran | 2008-2011 | Tehran Lipid and Glucose Study | Community | Urban | 20+ | 20+ | 4,622 | 5,884 |
| 829 | Iran | 2010-2012 | Golestan Cohort Study Second Phase | Subnational | Rural | 43-82 | 43-82 | 4,325 | 4,919 |
| 830 | Iran | 2010-2012 | Golestan Cohort Study Second Phase | Community | Urban | 43-82 | 43-82 | 1,091 | 1,061 |
| 831 | Iran | 2011 | STEPS | National | Both | 6-69 | 6-69 | 4,903 | 6,548 |
| 832 | Iran | 2010-2011 | The Yazd Eye Study | Subnational | Both | 40-80 | 40-80 | 876 | 1,012 |
| 833 | Iran | 2011-2012 | Amol county study | Community | Rural | 10+ | 10+ | 1,862 | 1,098 |
| 834 | Iran | 2011-2012 | Amol county study | Community | Urban | 10+ | 10+ | 1,624 | 1,548 |
| 835 | Iran | 2012 | National Integrated Micronutrient Survey (NIMS) 2012 | National | Both | 6-60 | 6-60 | 10,526 | 11,087 |
| 836 | Iran | 2012-2013 | Tehran City | Community | Urban | 10-90 | 10-90 | 419 | 537 |
| 837 | Iran | 2012-2014 | Pars Cohort Study | Community | Rural | 40-90 | 40-90 | 4,272 | 4,987 |
| 838 | Iran | 2012-2013 | Zahedan City | Community | Urban | 10-90 | 10-90 | 1,377 | 1,205 |
| 839 | Iran | 2013-2014 | Bushehr Elderly Health Program (BEH) | Community | Urban | 60+ | 60+ | 1,437 | 1,514 |
| 840 | Iran | 2013-2014 | Gilan Eye Study | Subnational | Both | 50+ | 50+ | 1,059 | 1,439 |
| 841 | Iran | 2014-2016 | The PERSIAN Fasa Cohort Study | Community | Both | 35-70 | 35-70 | 4,463 | 5,384 |
| 842 | Iran | 2014-2016 | The PERSIAN Guilan Cohort Study | Community | Both | 35-70 | 35-70 | 4,881 | 5,611 |
| 843 | Iran | 2014-2016 | The PERSIAN Kermanshah Cohort Study | Community | Both | 35-70 | 35-70 | 4,746 | 5,167 |
| 844 | Iran | 2014-2016 | The PERSIAN Kharameh Cohort Study | Community | Both | 35-70 | 35-70 | 4,707 | 5,860 |
| 845 | Iran | 2014-2016 | The PERSIAN Tabriz Cohort Study | Community | Both | 35-70 | 35-70 | 6,644 | 8,140 |
| 846 | Iran | 2015-2017 | The PERSIAN Mazandaran Cohort Study | Community | Both | 35-70 | 35-70 | 4,115 | 6,029 |
| 847 | Iran | 2015-2017 | The PERSIAN Rafsanjan Cohort Study | Community | Both | 35-70 | 35-70 | 5,152 | 5,300 |
| 848 | Iran | 2016 | STEPS | National | Both | 18+ | 18+ | 14,080 | 15,036 |
| 849 | Iran | 2015-2017 | The PERSIAN Yazd Cohort Study | Community | Both | 30-70 | 30-70 | 4,947 | 4,819 |
| 850 | Iran | 2016-2018 | The PERSIAN Ahvaz Cohort Study | Community | Both | 35-70 | 35-70 | 3,982 | 5,833 |
| 851 | Iran | 2016-2018 | The PERSIAN BandarKong Cohort Study | Community | Both | 35-70 | 35-70 | 1,702 | 2,260 |
| 852 | Iran | 2016-2018 | The PERSIAN Urmia Cohort Study | Community | Both | 35-70 | 35-70 | 2,161 | 2,796 |
| 853 | Iran | 2015-2018 | The PERSIAN Zahedan Cohort Study | Community | Urban | 35-70 | 35-70 | 3,890 | 6,013 |
| 854 | Iran | 2016-2019 | The PERSIAN Ardabil Cohort Study | Community | Both | 35-70 | 35-70 | 6,658 | 7,931 |
| 855 | Iran | 2018-2019 | Prevalence of risk factors for cardiovascular disease among a rural population in eastern Iran | Subnational | Rural | 18+ | 18+ | 148 | 146 |
| 856 | Iran | 2017-2018 | The PERSIAN Kavar Cohort Study | Community | Urban | 35-70 | 35-70 | 2,417 | 2,539 |
| 857 | Iran | 2016-2019 | The Khuzestan comprehensive health study: A platform for NCDs, blood borne and mental diseases research | Subnational | Both | 20-65 | 20-65 | 10,846 | 19,427 |
| 858 | Iran | 2017-2019 | The PERSIAN Mashhad Cohort Study | Community | Both | 35-70 | 35-70 | 2,276 | 2,619 |
| 859 | Iran | 2017-2018 | PERSIAN elderly component-Iranian Longitudinal Study on Ageing | Subnational | Both | 50-95 | 50-95 | 3,360 | 3,857 |
| 860 | Iran | 2016-2019 | The PERSIAN Shahrekord Cohort Study | Community | Both | 35-70 | 35-70 | 4,222 | 4,464 |
| 861 | Iraq | 2006 | STEPS | National | Both | 25-64 | 25-64 | 2,251 | 2,252 |
| 862 | Iraq | 2015 | STEPS | National | Both | 18+ | 18+ | 1,589 | 2,312 |
| 863 | Ireland | 1998 | Survey of Lifestyle, Attitudes and Nutritional in Ireland 1998 | National | Both | 18+ | 18+ | 123 | 296 |
| 864 | Ireland | 1997-1999 | North/South Ireland Food Consumption Survey | National | Both | 18-64 | 18-64 | 613 | 698 |
| 865 | Ireland | 2002 | Survey of Lifestyle, Attitudes and Nutritional in Ireland 2002 | National | Both | 18+ | 18+ | 164 | 216 |
| 866 | Ireland | 2006-2007 | Survey of Lifestyle, Attitudes and Nutritional in Ireland 2006-2007 | National | Both | 18+ | 18+ | 945 | 1,225 |
| 867 | Ireland | 2008-2010 | National Adult Nutrition Survey | National | Both | 18+ | 18+ | 658 | 696 |
| 868 | Ireland | 2009-2011 | The Irish Longitudinal Study on Ageing | National | Both | 50+ | 50+ | 2,693 | 3,170 |
| 869 | Israel | 1985-1986 | MONICA, Tel Aviv | Community | Urban | 25-64 | 25-64 | 653 | 685 |
| 870 | Israel | 1990-1991 | The Jerusalem Longitudinal Cohort Study | Community | Urban | 69-70 | 69-70 | 245 | 199 |
| 871 | Israel | 1997-1998 | The Jerusalem Longitudinal Cohort Study | Community | Urban | 76-77 | 76-77 | 422 | 429 |
| 872 | Israel | 1999-2001 | Mabat First Israeli National Health and Nutrition Survey | National | Both | 25-64 | 25-64 | 1,371 | 1,410 |
| 873 | Israel | 1999-2005 | The Israel Glucose Intolerance, Obesity and Hypertension Study | National | Urban | 58+ | 58+ | 514 | 527 |
| 874 | Israel | 2002-2007 | Hadera District Study | Subnational | Urban | 25-78 | 25-78 | 548 | 538 |
| 875 | Israel | 2005-2006 | Mabat Zahav First National Health and Nutrition Survey in ages 65 and over | National | Urban | 65+ | 65+ | 743 | 819 |
| 876 | Israel | 2014-2016 | Mabat Second Israeli National Health and Nutrition Survey | National | Both | 18-64 | 18-64 | 1,061 | 1,073 |
| 877 | Israel | 2014-2015 | Mabat Zahav Second National Health and Nutrition Survey ages in 65 and over | National | Both | 65+ | 65+ | 307 | 318 |
| 878 | Italy | 1985 | Finland, Italy, Netherlands, Elderly (Fine-Italy) | Community | Rural | 65-84 |  | 650 |  |
| 879 | Italy | 1985 | INTERSALT, Naples | Community | Urban | 20-59 | 20-59 | 100 | 100 |
| 880 | Italy | 1986 | INTERSALT, Bassiano | Community | Urban | 20-59 | 20-59 | 99 | 100 |
| 881 | Italy | 1986 | INTERSALT, Gubbio | Community | Urban | 20-59 | 20-59 | 99 | 100 |

| 882 | Italy | 1986 | INTERSALT, Mirano | Community | Urban | 20-59 | 20-59 | 100 | 100 |
| --- | --- | --- | --- | --- | --- | --- | --- | --- | --- |
| 883 | Italy | 1986 | MONICA, Friuli | Subnational | Urban | 25-64 | 25-64 | 921 | 918 |
| 884 | Italy | 1986-1987 | Malattie cardiovascolari ATerosclerotiche Istituto Superiore di Sanità (MATISS) | Community | Rural | 19-72 | 19-72 | 1,273 | 1,568 |
| 885 | Italy | 1985-1988 | Pisa Epidemiological Study - first survey | Community | Urban | 5-90 | 5-90 | 1,834 | 2,019 |
| 886 | Italy | 1986-1987 | MONICA, Brianza | Subnational | Urban | 25-64 | 25-64 | 814 | 832 |
| 887 | Italy | 1989 | MONICA, Friuli | Subnational | Urban | 25-64 | 25-64 | 902 | 900 |
| 888 | Italy | 1989 | Ventimiglia Heart Study | Community | Rural | 6+ | 6+ | 602 | 709 |
| 889 | Italy | 1990 | Bruneck Study | Community | Rural | 40-79 | 40-79 | 469 | 450 |
| 890 | Italy | 1988-1991 | Po river delta Epidemiological Study - second survey | Community | Rural | 8-73 | 8-73 | 1,341 | 1,497 |
| 891 | Italy | 1989-1990 | MONICA, Brianza | Subnational | Urban | 25-64 | 25-64 | 787 | 786 |
| 892 | Italy | 1991-1993 | Pisa Epidemiological Study - second survey | Community | Urban | 8-97 | 8-97 | 1,288 | 1,553 |
| 893 | Italy | 1992-1993 | Italian Longitudinal Study on Aging | National | Both | 65-84 | 65-84 | 1,666 | 1,455 |
| 894 | Italy | 1993-1996 | Malattie cardiovascolari ATerosclerotiche Istituto Superiore di Sanità (MATISS) | Community | Rural | 20-77 | 20-77 | 965 | 999 |
| 895 | Italy | 1994 | MONICA, Friuli | Subnational | Urban | 25-64 | 25-64 | 882 | 888 |
| 896 | Italy | 1993-1994 | MONICA, Brianza | Subnational | Urban | 25-64 | 25-64 | 801 | 856 |
| 897 | Italy | 1995 | Bruneck Study | Community | Rural | 45-84 | 45-84 | 411 | 408 |
| 898 | Italy | 1992-1998 | Vobarno Study | Community | Both | 25-64 | 35-64 | 265 | 309 |
| 899 | Italy | 1993-1998 | EPIC Florence | Community | Urban | 24-72 | 24-72 | 3,498 | 9,968 |
| 900 | Italy | 1995-1996 | Friuli Studio Emostatico | Community | Urban | 45-64 | 45-64 | 198 | 198 |
| 901 | Italy | 1995-1996 | Italian Longitudinal Study on Aging | National | Both | 68-90 | 68-90 | 1,011 | 808 |
| 902 | Italy | 1995-1999 | PROgetto Veneto Anziani (PROVA) | Subnational | Both | 65+ | 65+ | 1,187 | 1,722 |
| 903 | Italy | 1997-1999 | Lucca CUORE Study | Community | Urban | 15-84 | 15-84 | 897 | 1,123 |
| 904 | Italy | 1998-1999 | Progetto VIP | Community | Both | 25-74 | 25-74 | 599 | 600 |
| 905 | Italy | 1998-2000 | InCHIANTI study | Community | Both | 15+ | 15+ | 560 | 681 |
| 906 | Italy | 2000 | Bruneck Study | Community | Rural | 50-89 | 50-89 | 331 | 361 |
| 907 | Italy | 1998-2002 | Osservatorio Epidemiologico Cardiovascolare | National | Both | 35-74 | 35-74 | 4,870 | 4,752 |
| 908 | Italy | 2000-2001 | Italian Longitudinal Study on Aging | National | Both | 73-93 | 73-93 | 557 | 473 |
| 909 | Italy | 2000-2003 | PROgetto Veneto Anziani (PROVA) | Subnational | Both | 67+ | 67+ | 795 | 1,331 |
| 910 | Italy | 2001-2003 | The Study of Asti | Community | Both | 45-64 | 45-64 | 780 | 878 |
| 911 | Italy | 2003 | The European Male Ageing Study | Community | Both | 40+ |  | 433 |  |
| 912 | Italy | 2002-2005 | PROgetto Veneto Anziani (PROVA) | Subnational | Both | 68+ | 68+ | 621 | 1,138 |
| 913 | Italy | 2005 | Bruneck Study | Community | Rural | 55-93 | 55-93 | 264 | 307 |
| 914 | Italy | 2004-2005 | Vobarno study | Community | Rural | 55-74 | 55-74 | 99 | 113 |
| 915 | Italy | 2004-2005 | Italian Project on the Epidemiology of Alzheimer's Disease | National | Both | 65-84 | 65-84 | 1,569 | 1,421 |
| 916 | Italy | 2005-2007 | Moli-family Study | Subnational | Both | 14+ | 14+ | 243 | 301 |
| 917 | Italy | 2004-2008 | Cardiolab project | National | Urban | 40+ | 40+ | 19,152 | 14,782 |
| 918 | Italy | 2008 | The European Male Ageing Study | Community | Both | 40+ |  | 346 |  |
| 919 | Italy | 2005-2010 | Moli-sani Study | Subnational | Both | 35+ | 35+ | 11,694 | 12,614 |
| 920 | Italy | 2008-2009 | Progetto VIP | Community | Both | 25-74 | 25-74 | 597 | 598 |
| 921 | Italy | 2010 | Bruneck Study | Community | Rural | 60-98 | 60-98 | 225 | 259 |
| 922 | Italy | 2008-2012 | Osservatorio Epidemiologico Cardiovascolare/Health Examination Survey | National | Both | 35-80 | 35-80 | 4,368 | 4,332 |
| 923 | Italy | 2009-2011 | Pisa Epidemiological Study - third survey (Pisa 3 study) | Community | Urban | 6+ | 6+ | 496 | 574 |
| 924 | Italy | 2009-2010 | Grosso et al., J Epidemiol 24(4):327-33, 2014 | Community | Both | 19+ | 19+ | 760 | 1,129 |
| 925 | Italy | 2010-2012 | CArdiovascular risk MEtabolic syndrome LIver and Autoimmunity diseases (CA.ME.LI.A) | Community | Both | 18-75 | 18-75 | 477 | 515 |
| 926 | Italy | 2011-2012 | Vobarno study | Community | Rural | 49-62 | 49-62 | 107 | 143 |
| 927 | Italy | 2015 | Bruneck Study | Community | Rural | 65-98 | 65-98 | 171 | 169 |
| 928 | Italy | 2014-2016 | Mediterranean healthy Eating, Aging and Lifestyles (MEAL) study | Subnational | Urban | 20+ | 20+ | 762 | 762 |
| 929 | Italy | 2018-2019 | Progetto VIP | Community | Both | 25-74 | 25-74 | 600 | 598 |
| 930 | Jamaica | 1993 | Zohoori et al., West Indian Med J 52(2):111-17, 2003 | Community | Urban | 25-74 | 25-74 | 845 | 1,245 |
| 931 | Jamaica | 1994-1995 | Cooper et al., Am J Public Health 87(2):160-68, 1997 | Community | Urban | 25-100 | 25-100 | 597 | 833 |
| 932 | Jamaica | 1998 | Ragoobirsingh et al., Diabetes Obes Metab 6(1):23–27, 2004 | National | Both | 15+ | 15+ | 552 | 945 |
| 933 | Jamaica | 2000-2001 | Jamaica Health and Lifestyle Survey | National | Both | 15-74 | 15-74 | 653 | 1,281 |
| 934 | Jamaica | 2007-2008 | Jamaica Health and Lifestyle Survey | National | Both | 15-74 | 15-74 | 862 | 1,904 |
| 935 | Jamaica | 2012 | Older Persons in Jamaica 2012 | National | Both | 60+ | 60+ | 158 | 205 |
| 936 | Japan | 1985 | INTERSALT, Osaka | Community | Urban | 20-59 | 20-59 | 100 | 97 |
| 937 | Japan | 1985 | INTERSALT, Tochigi | Community | Urban | 20-59 | 20-59 | 95 | 99 |
| 938 | Japan | 1985 | INTERSALT, Toyama | Community | Urban | 20-59 | 20-59 | 100 | 100 |
| 939 | Japan | 1985 | National Nutrition Survey | National | Both | 5+ | 5+ | 7,461 | 8,865 |
| 940 | Japan | 1985-1986 | Akabane Study | Community | Urban | 40-69 | 40-69 | 812 | 1,022 |
| 941 | Japan | 1986 | National Nutrition Survey | National | Both | 5+ | 5+ | 7,280 | 8,635 |
| 942 | Japan | 1987 | Konan Town Study | Community | Rural | 20-79 | 20-79 | 70 | 88 |
| 943 | Japan | 1987 | National Nutrition Survey | National | Both | 5+ | 5+ | 6,427 | 8,160 |
| 944 | Japan | 1988 | Konan Town Study | Community | Rural | 20-79 | 20-79 | 76 | 85 |
| 945 | Japan | 1988 | National Nutrition Survey | National | Both | 5+ | 5+ | 6,885 | 8,045 |
| 946 | Japan | 1989 | Konan Town Study | Community | Rural | 20-79 | 20-79 | 58 | 63 |
| 947 | Japan | 1989 | Aito Town Study | Community | Rural | 5-74 | 5-84 | 529 | 525 |
| 948 | Japan | 1989 | National Nutrition Survey | National | Both | 5+ | 5+ | 5,767 | 6,882 |
| 949 | Japan | 1990 | Konan Town Study | Community | Rural | 20-79 | 20-79 | 27 | 51 |

| 950 | Japan | 1990 | National Nutrition Survey and National Cardiovascular Survey | National | Both | 5+ | 5+ | 6,080 | 7,291 |
| --- | --- | --- | --- | --- | --- | --- | --- | --- | --- |
| 951 | Japan | 1991 | Konan Town Study | Community | Rural | 20-79 | 20-79 | 93 | 116 |
| 952 | Japan | 1991 | Shigaraki Town Study | Community | Rural | 30-89 | 30-89 | 230 | 319 |
| 953 | Japan | 1991 | National Nutrition Survey | National | Both | 5+ | 5+ | 6,036 | 7,098 |
| 954 | Japan | 1992 | Konan Town Study | Community | Rural | 20-79 | 20-79 | 45 | 47 |
| 955 | Japan | 1992 | Shigaraki Town Study | Community | Rural | 30-89 | 30-89 | 288 | 385 |
| 956 | Japan | 1990-1994 | Japan Public Health Center-based prospective Study (JPHC Study), Cohort I | Subnational | Both | 40-59 | 40-59 | 8,749 | 14,481 |
| 957 | Japan | 1992 | National Nutrition Survey | National | Both | 5+ | 5+ | 5,635 | 6,656 |
| 958 | Japan | 1993 | Konan Town Study | Community | Rural | 20-79 | 20-79 | 54 | 65 |
| 959 | Japan | 1993 | Shigaraki Town Study | Community | Rural | 30-89 | 30-89 | 301 | 452 |
| 960 | Japan | 1993 | National Nutrition Survey | National | Both | 5+ | 5+ | 5,708 | 6,740 |
| 961 | Japan | 1994 | Konan Town Study | Community | Rural | 20-79 | 20-79 | 43 | 59 |
| 962 | Japan | 1994 | Shigaraki Town Study | Community | Rural | 30-89 | 30-89 | 251 | 336 |
| 963 | Japan | 1994 | Japanese Population-Based Osteoporosis Study | Subnational | Both |  | 15-79 |  | 3,222 |
| 964 | Japan | 1993-1994 | Japan Public Health Center-based prospective Study (JPHC Study), Cohort II | Subnational | Both | 40-69 | 40-69 | 8,534 | 16,190 |
| 965 | Japan | 1994 | National Nutrition Survey | National | Both | 5+ | 5+ | 5,439 | 6,386 |
| 966 | Japan | 1995 | Konan Town Study | Community | Rural | 20-79 | 20-79 | 45 | 61 |
| 967 | Japan | 1995 | Shigaraki Town Study | Community | Rural | 30-89 | 30-89 | 300 | 470 |
| 968 | Japan | 1995 | National Nutrition Survey | National | Both | 5+ | 5+ | 5,480 | 6,365 |
| 969 | Japan | 1996 | Shigaraki Town Study | Community | Rural | 30-89 | 30-89 | 86 | 152 |
| 970 | Japan | 1996 | National Nutrition Survey | National | Both | 5+ | 5+ | 5,277 | 6,185 |
| 971 | Japan | 1997 | Shigaraki Town Study | Community | Rural | 30-89 | 30-89 | 61 | 100 |
| 972 | Japan | 1996-1997 | INTERMAP, Aito Town | Community | Rural | 40-59 | 40-59 | 130 | 129 |
| 973 | Japan | 1997 | National Nutrition Survey | National | Both | 5+ | 5+ | 5,104 | 6,068 |
| 974 | Japan | 1997-1998 | INTERMAP, Sapporo | Community | Urban | 40-59 | 40-59 | 149 | 148 |
| 975 | Japan | 1997-1998 | INTERMAP, Toyama | Community | Urban | 40-59 | 40-59 | 149 | 150 |
| 976 | Japan | 1997-1998 | INTERMAP, Wakayama | Community | Urban | 40-59 | 40-59 | 146 | 144 |
| 977 | Japan | 1998 | Niigata Study | Community | Urban | 70 | 70 | 287 | 284 |
| 978 | Japan | 1998 | National Nutrition Survey | National | Both | 5+ | 5+ | 5,381 | 6,249 |
| 979 | Japan | 1999 | Niigata Study | Community | Urban | 71 | 71 | 245 | 216 |
| 980 | Japan | 1997-2000 | Sudo et al., J Orthop Sci 13(5):413-18, 2008 | Community | Rural | 55+ | 45+ | 261 | 785 |
| 981 | Japan | 1999 | National Nutrition Survey | National | Both | 5+ | 5+ | 4,367 | 5,333 |
| 982 | Japan | 2000 | Niigata Study | Community | Urban | 72 | 72 | 233 | 202 |
| 983 | Japan | 2000 | National Nutrition Survey and National Cardiovascular Survey | National | Both | 5+ | 5+ | 4,665 | 5,430 |
| 984 | Japan | 2001 | Niigata Study | Community | Urban | 73 | 73 | 235 | 201 |
| 985 | Japan | 2001 | The Japan Association of Health Service Database | Subnational | Both | 20+ | 20+ | 1,471,868 | 1,231,378 |
| 986 | Japan | 2001 | National Nutrition Survey | National | Both | 5+ | 5+ | 4,527 | 5,448 |
| 987 | Japan | 2002 | Niigata Study | Community | Urban | 74 | 74 | 228 | 202 |
| 988 | Japan | 2002 | National Nutrition Survey | National | Both | 5+ | 5+ | 4,104 | 4,941 |
| 989 | Japan | 2003 | Niigata Study | Community | Urban | 75 | 75 | 215 | 189 |
| 990 | Japan | 2002-2003 | The Hisayama Study | Community | Rural | 40+ | 40+ | 1,414 | 1,884 |
| 991 | Japan | 2003 | National Health and Nutrition Survey | National | Both | 5+ | 5+ | 4,035 | 4,920 |
| 992 | Japan | 2004 | Niigata Study | Community | Urban | 76 | 76 | 215 | 185 |
| 993 | Japan | 2004 | National Health and Nutrition Survey | National | Both | 5+ | 5+ | 3,384 | 3,952 |
| 994 | Japan | 2005 | Niigata Study | Community | Urban | 77 | 77 | 203 | 184 |
| 995 | Japan | 2005 | National Health and Nutrition Survey | National | Both | 5+ | 5+ | 3,154 | 3,802 |
| 996 | Japan | 2006 | Niigata Study | Community | Urban | 78 | 78 | 199 | 194 |
| 997 | Japan | 2006 | National Health and Nutrition Survey | National | Both | 5+ | 5+ | 3,522 | 4,165 |
| 998 | Japan | 2007 | Niigata Study | Community | Urban | 79 | 79 | 183 | 192 |
| 999 | Japan | 2007 | National Health and Nutrition Survey | National | Both | 5+ | 5+ | 3,520 | 4,154 |
| 1000 | Japan | 2008 | Resident in Kanazawa City (age 40+) | Community | Urban | 40+ | 40+ | 6,562 | 11,944 |
| 1001 | Japan | 2008 | National Health and Nutrition Survey | National | Both | 5+ | 5+ | 3,518 | 4,190 |
| 1002 | Japan | 2009 | National Health and Nutrition Survey | National | Both | 5+ | 5+ | 3,486 | 4,197 |
| 1003 | Japan | 2010 | National Health and Nutrition Survey | National | Both | 5+ | 5+ | 3,218 | 3,822 |
| 1004 | Japan | 2011 | National Health and Nutrition Survey | National | Both | 5+ | 5+ | 3,020 | 3,586 |
| 1005 | Japan | 2011 | The Tokyo Health Service Association Database | Community | Urban | 20+ | 20+ | 82,453 | 54,028 |
| 1006 | Japan | 2012 | National Health and Nutrition Survey | National | Both | 5+ | 5+ | 11,298 | 13,674 |
| 1007 | Japan | 2013 | National Health and Nutrition Survey | National | Both | 5+ | 5+ | 3,198 | 3,637 |
| 1008 | Japan | 2014-2015 | Nagaoka Health Screening | Community | Both | 20-89 | 20-89 | 4,938 | 4,298 |
| 1009 | Japan | 2012-2016 | The Nagahama study | Community | Rural | 35-80 | 35-80 | 3,206 | 6,620 |
| 1010 | Japan | 2014 | National Health and Nutrition Survey | National | Both | 5+ | 5+ | 3,208 | 3,657 |
| 1011 | Japan | 2015 | National Health and Nutrition Survey | National | Both | 5+ | 5+ | 2,914 | 3,457 |
| 1012 | Japan | 2016 | National Health and Nutrition Survey | National | Both | 5+ | 5+ | 9,814 | 11,638 |
| 1013 | Japan | 2017 | National Health and Nutrition Survey | National | Both | 5+ | 5+ | 2,662 | 3,057 |
| 1014 | Japan | 2017 | The Tokyo Health Service Association Database | Community | Urban | 20+ | 20+ | 63,713 | 47,577 |
| 1015 | Jordan | 1994-1996 | Ajlouni, Int J Obes Relat Metab Disord 22(7), 1998 | Subnational | Both | 25+ | 25+ | 1,047 | 1,787 |
| 1016 | Jordan | 1997 | DHS | National | Both |  | 20-49 |  | 3,002 |
| 1017 | Jordan | 2002 | DHS | National | Both |  | 20-49 |  | 4,839 |

| 1018 | Jordan | 2004 | Behavioural Risk Factor Surveillence Survey | National | Rural | 18+ | 18+ | 236 | 473 |
| --- | --- | --- | --- | --- | --- | --- | --- | --- | --- |
| 1019 | Jordan | 2004 | Khader et al., Metab Syndr Relat Disord 6(2):113-20, 2008 | Community | Both | 25+ | 25-59 | 394 | 548 |
| 1020 | Jordan | 2007 | DHS | National | Both |  | 20-49 |  | 4,451 |
| 1021 | Jordan | 2007 | Behavioural Risk Factor Surveillence Survey | National | Both | 18+ | 18+ | 332 | 433 |
| 1022 | Jordan | 2009 | DHS | National | Both |  | 20-49 |  | 4,054 |
| 1023 | Jordan | 2009 | Metablic abnomalities and vitamin D study | National | Both | 7+ | 7+ | 1,601 | 3,863 |
| 1024 | Jordan | 2012 | DHS | National | Both |  | 20-49 |  | 6,357 |
| 1025 | Jordan | 2016-2017 | National Cardiovascular Diseases and Diabetes Study (NCDDS) | National | Both | 18+ | 18+ | 1,187 | 2,745 |
| 1026 | Jordan | 2017-2018 | DHS | National | Both |  | 15-49 |  | 6,261 |
| 1027 | Jordan | 2019 | STEPS | National | Both | 18-69 | 18-69 | 2,009 | 3,084 |
| 1028 | Kazakhstan | 1985 | Balakhmetova et l., Ter Arkh 63(1):17-20, 1991 | Community | Urban | 20-54 |  | 2,886 |  |
| 1029 | Kazakhstan | 1995 | DHS | National | Both |  | 15-49 |  | 3,542 |
| 1030 | Kazakhstan | 1999 | DHS | National | Both |  | 15-49 |  | 2,227 |
| 1031 | Kazakhstan | 2015 | Almaty STEPS | Subnational | Both | 18-69 | 18-69 | 385 | 1,145 |
| 1032 | Kazakhstan | 2015 | Shymkent STEPS | Subnational | Both | 18-69 | 18-69 | 400 | 808 |
| 1033 | Kazakhstan | 2015-2016 | Aktobe STEPS | Subnational | Both | 18-69 | 18-69 | 348 | 1,153 |
| 1034 | Kazakhstan | 2019 | A health status assessment of a population of Karaganda urban region | Community | Urban | 18+ | 18+ | 324 | 670 |
| 1035 | Kenya | 1985 | INTERSALT | Community | Rural | 20-59 | 20-59 | 90 | 86 |
| 1036 | Kenya | 1993 | DHS | National | Both |  | 20-49 |  | 3,113 |
| 1037 | Kenya | 1998 | DHS | National | Both |  | 20-49 |  | 3,009 |
| 1038 | Kenya | 2003 | DHS | National | Both |  | 15-49 |  | 7,189 |
| 1039 | Kenya | 2008-2009 | DHS | National | Both |  | 15-49 |  | 7,827 |
| 1040 | Kenya | 2014 | DHS | National | Both |  | 15-49 |  | 13,469 |
| 1041 | Kenya | 2015 | STEPS | National | Both | 18-69 | 18-69 | 1,751 | 2,514 |
| 1042 | Kenya | 2018 | Assessing the gaps in healthcare and determining the feasibility for the setup of a social enterprise - Viwandani Lown Community Health Center, Kenya | Community | Urban | 19-73 | 19-73 | 153 | 143 |
| 1043 | Kiribati | 2004 | STEPS | National | Both | 15-64 | 15-64 | 779 | 939 |
| 1044 | Kiribati | 2015-2016 | STEPS | National | Both | 18-69 | 18-69 | 557 | 694 |
| 1045 | Kuwait | 1993-1994 | al-Isa, Ann Nutr Metab 41(5):307-14, 1997 | Community | Both | 18+ |  | 1,730 |  |
| 1046 | Kuwait | 1995-1996 | Abdella et al., Diabetes Res and Clin Pract 42(3):187–196, 1998 | Subnational | Both | 20-84 | 20-84 | 1,099 | 1,892 |
| 1047 | Kuwait | 1998 | Abiaka et al., , Biol Trace Elem Res 91(1):33-43, 2003 | National | Both | 15-80 | 15-80 | 178 | 233 |
| 1048 | Kuwait | 2006 | STEPS | National | Both | 20-64 | 20-64 | 918 | 1,298 |
| 1049 | Kuwait | 2008-2010 | Gulf Cooperation Council World Health Survey | National | Both | 18+ | 18+ | 1,598 | 1,782 |
| 1050 | Kuwait | 2008-2009 | National Nutrition Program for the State of Kuwait | National | Urban | 5+ | 5+ | 772 | 830 |
| 1051 | Kuwait | 2011-2014 | Kuwait Diabetes Epidemiology Program | National | Urban | 18-82 | 18-82 | 3,007 | 2,242 |
| 1052 | Kuwait | 2014 | STEPS | National | Both | 18-69 | 18-69 | 1,382 | 2,212 |
| 1053 | Kyrgyzstan | 1993 | KyrgyzstanMultipurpose Poverty Surveys | National | Both | 18-60 | 18-60 | 2,457 | 2,457 |
| 1054 | Kyrgyzstan | 1997 | DHS | National | Both |  | 15-49 |  | 3,570 |
| 1055 | Kyrgyzstan | 2012 | DHS | National | Both |  | 15-49 |  | 7,516 |
| 1056 | Kyrgyzstan | 2013 | STEPS | National | Both | 25-64 | 25-64 | 942 | 1,600 |
| 1057 | Lao PDR | 2006 | Multiple Indicator Cluster Survey 3 | National | Both |  | 15-49 |  | 807 |
| 1058 | Lao PDR | 2008 | STEPS | Community | Both | 25-64 | 25-64 | 1,568 | 2,353 |
| 1059 | Lao PDR | 2013 | STEPS | National | Both | 18-64 | 18-64 | 984 | 1,461 |
| 1060 | Latvia | 1997 | Nutrition and lifestyle in the baltic republics, WHO, 1997 | National | Both | 19-50 | 19-50 | 703 | 732 |
| 1061 | Latvia | 2008-2009 | Cardiovascular risk factor study | National | Both | 25-74 | 25-74 | 1,362 | 2,398 |
| 1062 | Lebanon | 1997 | Obesity in Lebanon: National Survey | National | Both | 5+ | 5+ | 871 | 1,164 |
| 1063 | Lebanon | 2008-2009 | STEPS | National | Both | 5+ | 5+ | 1,721 | 1,886 |
| 1064 | Lebanon | 2017 | STEPS | National | Both | 18-69 | 18-69 | 729 | 983 |
| 1065 | Lesotho | 1993 | National survey on iodine, vitamin A and iron status of women and children in Lesotho | National | Both |  | 20-65 |  | 792 |
| 1066 | Lesotho | 2004-2005 | DHS | National | Both |  | 15-49 |  | 3,206 |
| 1067 | Lesotho | 2009-2010 | DHS | National | Both | 15-59 | 15-49 | 3,216 | 3,781 |
| 1068 | Lesotho | 2012 | STEPS | National | Both | 25-64 | 25-64 | 726 | 1,442 |
| 1069 | Lesotho | 2014 | DHS | National | Both | 15-59 | 15-49 | 2,860 | 3,244 |
| 1070 | Liberia | 2006-2007 | DHS | National | Both |  | 15-49 |  | 6,419 |
| 1071 | Liberia | 2011 | STEPS | National | Both | 25-64 | 25-64 | 998 | 1,254 |
| 1072 | Liberia | 2013 | DHS | National | Both | 15-49 | 15-49 | 4,235 | 4,718 |
| 1073 | Libya | 1998-1999 | Kadiki et al., Diabetes Metab 27(6):647-54, 2001 | Community | Both | 15+ | 15+ | 228 | 398 |
| 1074 | Libya | 2009 | STEPS | National | Both | 25-64 | 25-64 | 1,678 | 1,564 |
| 1075 | Lithuania | 1986-1987 | MONICA, Kaunas | Community | Urban | 35-64 | 35-64 | 894 | 868 |
| 1076 | Lithuania | 1987 | Countrywide Integrated Noncommunicable Diseases Intervention Programme | Subnational | Rural | 25-64 | 25-64 | 1,220 | 1,434 |
| 1077 | Lithuania | 1992-1993 | MONICA, Kaunas | Community | Urban | 35-64 | 35-64 | 610 | 621 |
| 1078 | Lithuania | 1992-1993 | Countrywide Integrated Noncommunicable Diseases Intervention Programme | Subnational | Rural | 25-64 | 25-64 | 617 | 798 |
| 1079 | Lithuania | 1997 | Pomerleau, 2000 | National | Both | 19+ | 19+ | 966 | 1,130 |
| 1080 | Lithuania | 1998-1999 | Countrywide Integrated Noncommunicable Diseases Intervention Programme | Subnational | Rural | 25-64 | 25-64 | 816 | 1,021 |
| 1081 | Lithuania | 2002 | Pomerleau et al., Public Health Nutrition 3: 3-10, 2000 | National | Both | 24-70 | 24-70 | 977 | 928 |
| 1082 | Lithuania | 2001-2002 | MONICA4 | Community | Urban | 35-64 | 35-64 | 625 | 776 |
| 1083 | Lithuania | 2006-2007 | Countrywide Integrated Noncommunicable Diseases Intervention Programme | Subnational | Rural | 25-64 | 25-64 | 718 | 972 |
| 1084 | Lithuania | 2006-2008 | Health, Alcohol and Psychosocial Factors In Eastern Europe | Community | Urban | 45-75 | 45-75 | 3,231 | 3,874 |
| 1085 | Luxembourg | 2007-2009 | Observation of cardiovascular risk factors in Luxembourg (ORISCAV-LUX) | National | Both | 18-69 | 18-69 | 696 | 735 |

| 1086 | Luxembourg | 2013-2015 | European Health Examination Survey | National | Both | 25-64 | 25-64 | 721 | 785 |
| --- | --- | --- | --- | --- | --- | --- | --- | --- | --- |
| 1087 | Luxembourg | 2016-2018 | Observation of cardiovascular risk factors in Luxembourg (ORISCAV-LUX2) | National | Both | 25-79 | 25-79 | 670 | 763 |
| 1088 | Macedonia (TFYR) | 1999 | Multiple Indicator Cluster Survey | National | Both |  | 15-45 |  | 1,038 |
| 1089 | Madagascar | 1997 | DHS | National | Both |  | 20-49 |  | 2,253 |
| 1090 | Madagascar | 1997 | Mauny et al., Ann Trop Med Parasitol 97(6):645-54, 2003 | Community | Both | 15+ | 15+ | 248 | 283 |
| 1091 | Madagascar | 2003-2004 | DHS | National | Both |  | 15-49 |  | 7,155 |
| 1092 | Madagascar | 2005 | STEPS | Subnational | Both | 25-64 | 25-64 | 2,596 | 2,494 |
| 1093 | Madagascar | 2008-2009 | DHS | National | Both |  | 15-49 |  | 7,869 |
| 1094 | Malawi | 1992 | DHS | National | Both |  | 20-49 |  | 2,102 |
| 1095 | Malawi | 1996 | Chilima et al., Eur J Clin Nutr 52(9):643-9 | Community | Rural | 55-94 | 55-94 | 86 | 185 |
| 1096 | Malawi | 2000 | DHS | National | Both |  | 15-49 |  | 11,491 |
| 1097 | Malawi | 2004 | DHS | National | Both |  | 15-49 |  | 9,751 |
| 1098 | Malawi | 2009 | STEPS | National | Both | 25-64 | 25-64 | 1,666 | 3,189 |
| 1099 | Malawi | 2010 | DHS | National | Both |  | 15-49 |  | 7,118 |
| 1100 | Malawi | 2013-2017 | NCD Survey Malawi Epidemiology and Intervention Research Unit | Community | Rural | 18+ | 18+ | 5,849 | 7,507 |
| 1101 | Malawi | 2013-2017 | NCD Survey Malawi Epidemiology and Intervention Research Unit | Community | Urban | 18+ | 18+ | 5,802 | 10,291 |
| 1102 | Malawi | 2015-2016 | DHS | National | Both |  | 15-49 |  | 7,415 |
| 1103 | Malawi | 2017 | STEPS | National | Both | 18-69 | 18-69 | 1,478 | 2,534 |
| 1104 | Malaysia | 1996 | National Health and Morbidity Survey (NHMS) | National | Both | 18+ | 18+ | 14,520 | 16,244 |
| 1105 | Malaysia | 2002-2003 | Malaysian Adult Nutrition Survey | National | Both | 18-59 | 18-59 | 1,286 | 966 |
| 1106 | Malaysia | 2004 | Rampal et al., Public Health 2008; 122: 11-8 | National | Both | 15+ | 15+ | 6,834 | 9,293 |
| 1107 | Malaysia | 2005 | STEPS | National | Both | 25-64 | 25-64 | 1,286 | 1,286 |
| 1108 | Malaysia | 2006 | National Health and Morbidity Survey (NHMS) | National | Both | 5+ | 5+ | 22,970 | 25,508 |
| 1109 | Malaysia | 2008 | Metabolic Syndrome Study in Malaysia | National | Rural | 18+ | 18+ | 753 | 1,368 |
| 1110 | Malaysia | 2008 | Metabolic Syndrome Study in Malaysia | National | Urban | 18+ | 18+ | 769 | 1,446 |
| 1111 | Malaysia | 2011 | National Health and Morbidity Survey (NHMS) | National | Both | 5+ | 5+ | 8,033 | 8,780 |
| 1112 | Malaysia | 2014 | Malaysian Adult Nutrition Survey | National | Both | 18-59 | 18-59 | 1,328 | 1,495 |
| 1113 | Malaysia | 2015 | National Health and Morbidity Survey (NHMS) | National | Both | 18+ | 18+ | 8,916 | 9,581 |
| 1114 | Maldives | 2001 | Multiple Indicator Cluster Survey | National | Both |  | 15-50 |  | 1,145 |
| 1115 | Maldives | 2004 | STEPS | Subnational | Urban | 25-64 | 25-64 | 933 | 1,086 |
| 1116 | Maldives | 2009 | DHS | National | Both |  | 20-49 |  | 5,139 |
| 1117 | Maldives | 2011 | STEPS | Subnational | Urban | 15-64 | 15-64 | 660 | 1,060 |
| 1118 | Maldives | 2016-2017 | DHS | National | Both | 15-49 | 15-49 | 3,831 | 6,839 |
| 1119 | Mali | 1995-1996 | DHS | National | Both |  | 20-49 |  | 3,789 |
| 1120 | Mali | 1997 | Programme Intégré de Développement de Bafoulabé | Community | Rural | 15-45 | 15-45 | 425 | 716 |
| 1121 | Mali | 1997 | Torheim et al., Eur J Clin Nutr 58(4):594-604, 2004 | Subnational | Rural | 15-44 | 15-44 | 237 | 337 |
| 1122 | Mali | 1999-2000 | Bafoulabe Iodine Study | Community | Rural |  | 15-45 |  | 365 |
| 1123 | Mali | 1999 | Torheim et al., Public Health Nutr 8(4):387-94, 2005 | Subnational | Rural |  | 15-44 |  | 191 |
| 1124 | Mali | 2001 | DHS | National | Both |  | 15-49 |  | 10,526 |
| 1125 | Mali | 2006 | DHS | National | Both |  | 15-49 |  | 12,512 |
| 1126 | Mali | 2007 | STEPS | Subnational | Both | 15-64 | 15-64 | 1,036 | 1,494 |
| 1127 | Mali | 2012-2013 | DHS | National | Both |  | 15-49 |  | 4,646 |
| 1128 | Mali | 2013 | Santé Nutritionnelle à Assise Comunautaire dans la région de Kayes (SNACK) | Subnational | Rural |  | 20-68 |  | 4,595 |
| 1129 | Mali | 2018 | DHS | National | Both |  | 15-49 |  | 4,576 |
| 1130 | Malta | 1986 | INTERSALT | Community | Rural | 20-59 | 20-59 | 100 | 100 |
| 1131 | Marshall Islands | 2002 | STEPS | National | Both | 15-64 | 15-64 | 772 | 1,195 |
| 1132 | Marshall Islands | 2017-2018 | STEPS | National | Both | 18+ | 18+ | 1,246 | 1,392 |
| 1133 | Mauritania | 2000-2001 | DHS | National | Both |  | 15-49 |  | 2,635 |
| 1134 | Mauritania | 2006 | STEPS | Community | Urban | 15-64 | 15-64 | 1,132 | 1,300 |
| 1135 | Mauritius | 1987 | Mauritius Noncommunicable Disease Survey | National | Both | 25-74 | 25-74 | 2,347 | 2,653 |
| 1136 | Mauritius | 1992 | Mauritius Noncommunicable Disease Survey | National | Both | 25-74 | 25-74 | 2,985 | 3,477 |
| 1137 | Mauritius | 1998 | Mauritius Noncommunicable Disease Survey | National | Both | 25-74 | 25-74 | 2,566 | 3,248 |
| 1138 | Mauritius | 2009 | Mauritius Noncommunicable Disease Survey | National | Both | 19-74 | 19-74 | 2,859 | 3,391 |
| 1139 | Mexico | 1988-1989 | Encuesta Nacional de Nutricion | National | Both |  | 12-49 |  | 16,617 |
| 1140 | Mexico | 1990-1992 | Mexico City Diabetes Study | Community | Urban | 30+ | 30+ | 941 | 1,341 |
| 1141 | Mexico | 1992-1993 | Encuesta Nacional de Enfermedades Cronicas | National | Urban | 20-69 | 20-69 | 6,040 | 8,298 |
| 1142 | Mexico | 1993-1995 | Mexico City Diabetes Study | Community | Urban | 34+ | 34+ | 707 | 1,033 |
| 1143 | Mexico | 1996 | Sanchez-Castillo et al., Eur J Clin Nutr 55(10):833-40, 2001 | Community | Rural | 18+ | 18+ | 104 | 149 |
| 1144 | Mexico | 1998-1999 | Encuesta Nacional de Nutricion | National | Both |  | 12-49 |  | 17,892 |
| 1145 | Mexico | 1997-1999 | Mexico City Diabetes Study | Community | Urban | 37+ | 37+ | 701 | 980 |
| 1146 | Mexico | 2000 | Encuesta Nacional de Salud | National | Both | 10+ | 10+ | 22,554 | 39,204 |
| 1147 | Mexico | 1999-2000 | The Survey on Health, Well-Being, and Aging in Latin America and the Caribbean (SABE) | Community | Urban | 60+ | 60+ | 359 | 548 |
| 1148 | Mexico | 2001 | The Mexican Health and Aging Study | National | Both | 50+ | 50+ | 1,030 | 1,224 |
| 1149 | Mexico | 1998-2004 | Mexico City Prospective Study | Community | Urban | 35-84 | 35-84 | 51,768 | 105,313 |
| 1150 | Mexico | 2002 | Encuesta Nacional Sobre Niveles de vida de los Hogares | National | Both | 5+ | 5+ | 11,606 | 13,614 |
| 1151 | Mexico | 2003 | The Mexican Health and Aging Study | National | Both | 50+ | 50+ | 893 | 1,162 |
| 1152 | Mexico | 2005 | Encuesta Nacional Sobre Niveles de vida de los Hogares | National | Both | 5+ | 5+ | 11,696 | 13,211 |
| 1153 | Mexico | 2004-2005 | CArdiovascular Risk factors Multiple Evaluation in Latin America | Community | Urban | 25-64 | 25-64 | 833 | 894 |

| 1154 | Mexico | 2006 | Encuesta Nacional de Salud y Nutrición | National | Both | 5+ | 5+ | 27,848 | 34,909 |
| --- | --- | --- | --- | --- | --- | --- | --- | --- | --- |
| 1155 | Mexico | 2006 | PREVENIMSS National Coverage Surveys | National | Urban | 20+ | 20+ | 8,715 | 11,315 |
| 1156 | Mexico | 2007-2009 | Mexico City Diabetes Study | Community | Urban | 51+ | 51+ | 460 | 711 |
| 1157 | Mexico | 2009-2010 | SAGE | National | Both | 50+ | 50+ | 796 | 1,236 |
| 1158 | Mexico | 2010 | PREVENIMSS National Coverage Surveys | National | Urban | 20+ | 20+ | 6,238 | 6,003 |
| 1159 | Mexico | 2009-2012 | Encuesta Nacional Sobre Niveles de vida de los Hogares | National | Both | 5+ | 5+ | 4,908 | 4,697 |
| 1160 | Mexico | 2011-2012 | Encuesta Nacional de Salud Y Nutricion | National | Both | 5+ | 5+ | 31,293 | 36,834 |
| 1161 | Mexico | 2012 | The Mexican Health and Aging Study | National | Both | 50+ | 50+ | 786 | 1,106 |
| 1162 | Mexico | 2016 | Encuesta Nacional de Salud Y Nutricion | National | Both | 5+ | 5+ | 5,660 | 8,429 |
| 1163 | Mexico | 2018-2019 | Encuesta Nacional de Salud Y Nutricion | National | Both | 5+ | 5+ | 4,154 | 5,835 |
| 1164 | Micronesia (Federated States of | 2002 | STEPS | Subnational | Both | 25-64 | 25-64 | 591 | 893 |
| 1165 | Micronesia (Federated States of | 2006 | STEPS | Subnational | Both | 15-64 | 15-64 | 918 | 1,553 |
| 1166 | Micronesia (Federated States of | 2008 | STEPS | Subnational | Both | 25-64 | 25-64 | 875 | 1,266 |
| 1167 | Micronesia (Federated States of | 2009 | STEPS, Kosrae | Subnational | Both | 15-64 | 15-64 | 208 | 413 |
| 1168 | Micronesia (Federated States of | 2009 | STEPS, Yap | Subnational | Both | 15-64 | 15-64 | 405 | 521 |
| 1169 | Micronesia (Federated States of | 2016 | STEPS | Subnational | Both | 18-69 | 18-69 | 516 | 818 |
| 1170 | Moldova | 2005 | DHS | National | Both |  | 15-49 |  | 7,076 |
| 1171 | Moldova | 2013 | STEPS | National | Both | 18-69 | 18-69 | 1,712 | 2,777 |
| 1172 | Mongolia | 1999 | National Nutrition Survey | National | Both | 35-65 | 35-65 | 907 | 1,317 |
| 1173 | Mongolia | 2004 | National Nutrition Survey | National | Both | 15-74 | 15-74 | 248 | 360 |
| 1174 | Mongolia | 2005 | STEPS | National | Both | 15-64 | 15-64 | 1,669 | 1,717 |
| 1175 | Mongolia | 2009 | STEPS | National | Both | 15-64 | 15-64 | 2,197 | 3,117 |
| 1176 | Mongolia | 2013 | STEPS | National | Both | 15-64 | 15-64 | 2,698 | 3,167 |
| 1177 | Mongolia | 2019 | STEPS | National | Both | 15-69 | 15-69 | 2,926 | 3,543 |
| 1178 | Montenegro | 1985 | Anthropometric Characteristics of Montenegrin Recruiters from '70 and 80's | National | Both | 17-28 |  | 9,722 |  |
| 1179 | Montenegro | 1986 | Anthropometric Characteristics of Montenegrin Recruiters from '70 and 80's | National | Both | 17-28 |  | 9,961 |  |
| 1180 | Montenegro | 1987 | Anthropometric Characteristics of Montenegrin Recruiters from '70 and 80's | National | Both | 17-28 |  | 10,230 |  |
| 1181 | Montenegro | 2019 | Body Composition of high school students in Montenegro and its relationship with their eating habits | National | Both | 18-20 | 18-20 | 504 | 497 |
| 1182 | Morocco | 1992 | DHS | National | Both |  | 20-49 |  | 2,804 |
| 1183 | Morocco | 2000 | National Survey 2000 | National | Both | 20+ | 20+ | 755 | 1,047 |
| 1184 | Morocco | 2003-2004 | DHS | National | Both |  | 15-49 |  | 15,944 |
| 1185 | Morocco | 2017 | STEPS | National | Both | 18+ | 18+ | 1,871 | 3,390 |
| 1186 | Mozambique | 1997 | DHS | National | Both |  | 20-49 |  | 2,824 |
| 1187 | Mozambique | 2003 | DHS | National | Both |  | 15-49 |  | 10,535 |
| 1188 | Mozambique | 2005 | STEPS | National | Both | 25-64 | 25-64 | 1,276 | 1,689 |
| 1189 | Mozambique | 2011 | DHS | National | Both |  | 15-49 |  | 12,201 |
| 1190 | Mozambique | 2014-2015 | STEPS | National | Both | 15-64 | 15-64 | 1,147 | 1,684 |
| 1191 | Myanmar | 2003-2004 | STEPS | Subnational | Both | 25-74 | 25-74 | 1,990 | 2,449 |
| 1192 | Myanmar | 2009 | STEPS | National | Both | 15-64 | 15-64 | 2,826 | 4,421 |
| 1193 | Myanmar | 2011 | Underweight prevalence among young adults from rural areas, Salin Township, Magwe Region | Community | Rural | 15-35 | 15-35 | 156 | 233 |
| 1194 | Myanmar | 2014 | STEPS | National | Both | 25-64 | 25-64 | 2,947 | 5,444 |
| 1195 | Myanmar | 2013-2014 | STEPS 2013-2014 Yangon | Subnational | Both | 25-74 | 25-74 | 745 | 740 |
| 1196 | Myanmar | 2015-2016 | DHS | National | Both |  | 15-49 |  | 12,163 |
| 1197 | Namibia | 1992 | DHS | National | Both |  | 20-49 |  | 2,062 |
| 1198 | Namibia | 2005 | STEPS | National | Both | 25-64 | 25-64 | 1,390 | 1,778 |
| 1199 | Namibia | 2006-2007 | DHS | National | Both |  | 15-49 |  | 8,968 |
| 1200 | Namibia | 2009 | Okambilimbili Survey | Community | Urban | 5+ | 5+ | 962 | 1,167 |
| 1201 | Namibia | 2013 | DHS | National | Both |  | 15-64 |  | 5,111 |
| 1202 | Nauru | 1987 | Trends in the prevalence and incidence of non-insulin-dependent diabetes mellitus and impaired glucose tolerance | National | Both | 20+ | 20+ | 555 | 667 |
| 1203 | Nauru | 1994 | Trends in the prevalence and incidence of non-insulin-dependent diabetes mellitus and impaired glucose tolerance | National | Both | 25+ | 25+ | 647 | 731 |
| 1204 | Nauru | 2004 | STEPS | National | Both | 15-64 | 15-64 | 1,082 | 1,149 |
| 1205 | Nauru | 2006 | STEPS | National | Both | 16-65 | 16-65 | 255 | 236 |
| 1206 | Nauru | 2015 | STEPS | National | Rural | 18-69 | 18-69 | 505 | 540 |
| 1207 | Nepal | 1996 | DHS | National | Both |  | 20-49 |  | 3,068 |
| 1208 | Nepal | 1997 | Ohno et al., Asia Pac J Public Health 18(3):20-9, 2006 | Community | Rural | 17-75 | 17-75 | 36 | 41 |
| 1209 | Nepal | 2001 | DHS | National | Both |  | 20-49 |  | 7,216 |
| 1210 | Nepal | 2003 | STEPS | Subnational | Both | 25-64 | 25-64 | 1,010 | 996 |
| 1211 | Nepal | 2005 | STEPS | Subnational | Both | 15-64 | 15-64 | 3,634 | 3,998 |
| 1212 | Nepal | 2006 | DHS | National | Both |  | 15-49 |  | 10,117 |
| 1213 | Nepal | 2007-2008 | STEPS | National | Both | 15-64 | 15-64 | 1,889 | 2,348 |
| 1214 | Nepal | 2006-2011 | Early detection and management of Kidney disease, Hypertension, Diabetes and Cardiovascular disease (KHDC Nepal), Tarahara | Community | Rural | 18+ | 18+ | 1,175 | 2,350 |
| 1215 | Nepal | 2006-2011 | Early detection and management of Kidney disease, Hypertension, Diabetes and Cardiovascular disease (KHDC Nepal), Damak | Community | Urban | 18+ | 18+ | 1,095 | 1,576 |
| 1216 | Nepal | 2006-2011 | Early detection and management of Kidney disease, Hypertension, Diabetes and Cardiovascular disease (KHDC Nepal), Dharan | Community | Urban | 18+ | 18+ | 4,130 | 6,126 |
| 1217 | Nepal | 2011 | DHS | National | Both |  | 15-49 |  | 5,848 |
| 1218 | Nepal | 2012-2013 | STEPS | National | Both | 15-69 | 15-69 | 1,326 | 2,763 |
| 1219 | Nepal | 2015 | Community based intervention for prevention and control of non-communicable diseases risk factors (CIPCON) baseline survey, Dhankuta | Subnational | Rural | 15-69 | 15-69 | 555 | 781 |
| 1220 | Nepal | 2015 | Community based intervention for prevention and control of non-communicable diseases risk factors (CIPCON) baseline survey, Ilam | Subnational | Rural | 15-69 | 15-69 | 546 | 721 |
| 1221 | Nepal | 2016 | DHS | National | Both | 15-49 | 15-49 | 4,035 | 6,165 |

| 1222 | Nepal | 2016-2018 | The Population Based Prevalence of Selected Non-Communicable Diseases In Nepal | National | Both | 20+ | 20+ | 4,907 | 7,529 |
| --- | --- | --- | --- | --- | --- | --- | --- | --- | --- |
| 1223 | Netherlands | 1985 | INTERSALT | Community | Urban | 20-59 | 20-59 | 100 | 99 |
| 1224 | Netherlands | 1985 | Zutphen Elderly Study | Community | Urban | 65-85 |  | 886 |  |
| 1225 | Netherlands | 1990 | Zutphen Elderly Study | Community | Urban | 69-90 |  | 552 |  |
| 1226 | Netherlands | 1989-1993 | The Rotterdam Study, first subcohort | Community | Urban | 55+ | 55+ | 2,807 | 4,103 |
| 1227 | Netherlands | 1992-1993 | The Longitudinal Aging Study Amsterdam (LASA) | Subnational | Both | 55-85 | 55-85 | 1,266 | 1,308 |
| 1228 | Netherlands | 1993-1995 | The Rotterdam Study, first subcohort | Community | Urban | 56+ | 56+ | 2,214 | 3,105 |
| 1229 | Netherlands | 1993-1997 | EPIC Bilthoven | Community | Urban | 20-59 | 20-59 | 9,941 | 12,021 |
| 1230 | Netherlands | 1993-1997 | EPIC Utrecht | Community | Both |  | 49-70 |  | 17,335 |
| 1231 | Netherlands | 1995-1996 | The Longitudinal Aging Study Amsterdam (LASA) | Subnational | Both | 65-88 | 65-88 | 714 | 764 |
| 1232 | Netherlands | 1997-1999 | The Rotterdam Study, first subcohort | Community | Urban | 61+ | 61+ | 1,718 | 2,361 |
| 1233 | Netherlands | 1998-1999 | The Longitudinal Aging Study Amsterdam (LASA) | Subnational | Both | 61-91 | 61-91 | 604 | 743 |
| 1234 | Netherlands | 1998-2001 | Regenboog Project | National | Both | 12-89 | 12-89 | 2,714 | 2,643 |
| 1235 | Netherlands | 2000-2001 | The Rotterdam Study, second subcohort | Community | Urban | 55+ | 55+ | 1,210 | 1,468 |
| 1236 | Netherlands | 2001-2002 | The Longitudinal Aging Study Amsterdam (LASA) | Subnational | Both | 64-94 | 64-94 | 577 | 690 |
| 1237 | Netherlands | 2001-2003 | Surinamese in the Netherlands: Study on Ethnicity and Health (SUNSET) | Community | Urban | 35-60 | 35-60 | 251 | 257 |
| 1238 | Netherlands | 2002-2003 | The Longitudinal Aging Study Amsterdam (LASA) | Subnational | Both | 54-65 | 54-65 | 431 | 482 |
| 1239 | Netherlands | 2002-2004 | The Rotterdam Study, first subcohort | Community | Urban | 64+ | 64+ | 1,206 | 1,708 |
| 1240 | Netherlands | 2003-2007 | Doetinchem Cohort Study (4th measurement) | Subnational | Urban | 36-74 | 36-74 | 2,125 | 2,352 |
| 1241 | Netherlands | 2004-2005 | The Rotterdam Study, second subcohort | Community | Urban | 58+ | 58+ | 964 | 1,244 |
| 1242 | Netherlands | 2005-2006 | The Longitudinal Aging Study Amsterdam (LASA) | Subnational | Both | 57-97 | 57-97 | 789 | 958 |
| 1243 | Netherlands | 2006-2008 | The Rotterdam Study, third subcohort | Community | Urban | 45+ | 45+ | 1,547 | 2,029 |
| 1244 | Netherlands | 2008-2009 | The Longitudinal Aging Study Amsterdam (LASA) | Subnational | Both | 60-100 | 60-100 | 642 | 789 |
| 1245 | Netherlands | 2009-2010 | Measuring the Netherlands (NL de Maat) | Subnational | Both | 30-70 | 30-70 | 1,781 | 2,014 |
| 1246 | Netherlands | 2009-2011 | The Rotterdam Study, first subcohort | Community | Urban | 72+ | 72+ | 690 | 1,006 |
| 1247 | Netherlands | 2011-2012 | The Rotterdam Study, second subcohort | Community | Urban | 65+ | 65+ | 735 | 934 |
| 1248 | Netherlands | 2011-2012 | The Longitudinal Aging Study Amsterdam (LASA) | Subnational | Both | 63-104 | 63-104 | 532 | 653 |
| 1249 | Netherlands | 2012-2013 | The Longitudinal Aging Study Amsterdam (LASA) | Subnational | Both | 55-65 | 55-65 | 426 | 448 |
| 1250 | Netherlands | 2011-2015 | Healthy Life in an Urban Setting (HELIUS) | Community | Urban | 18-71 | 18-71 | 2,088 | 2,473 |
| 1251 | Netherlands | 2012-2014 | The Rotterdam Study, third subcohort | Community | Urban | 52+ | 52+ | 1,256 | 1,639 |
| 1252 | Netherlands | 2015-2016 | The Longitudinal Aging Study Amsterdam (LASA) | Subnational | Both | 58+ | 58+ | 759 | 857 |
| 1253 | New Zealand | 1989 | The Life in New Zealand Survey | National | Both | 15+ | 15+ | 1,418 | 1,571 |
| 1254 | New Zealand | 1990-1993 | Williams, N Z Med J 113(1114):308-11, 2000 | Community | Both | 18-21 | 18-21 | 932 | 859 |
| 1255 | New Zealand | 1993-1994 | MONICA, Auckland | Community | Urban | 35-64 | 35-64 | 723 | 674 |
| 1256 | New Zealand | 1996-1997 | National Nutrition Survey | National | Both | 15+ | 15+ | 1,857 | 2,522 |
| 1257 | New Zealand | 2002-2003 | New Zealand Health Survey | National | Both | 15+ | 15+ | 4,594 | 6,729 |
| 1258 | New Zealand | 2006-2007 | New Zealand Health Survey | National | Both | 5+ | 5+ | 6,766 | 8,032 |
| 1259 | New Zealand | 2008-2009 | New Zealand Adult Nutrition Survey | National | Both | 15+ | 15+ | 2,003 | 2,500 |
| 1260 | New Zealand | 2011-2012 | New Zealand Health Survey | National | Both | 5+ | 5+ | 5,783 | 7,220 |
| 1261 | New Zealand | 2012-2013 | New Zealand Health Survey | National | Both | 5+ | 5+ | 6,409 | 7,926 |
| 1262 | New Zealand | 2013-2014 | New Zealand Health Survey | National | Both | 5+ | 5+ | 6,971 | 8,310 |
| 1263 | New Zealand | 2014-2015 | New Zealand Health Survey | National | Both | 5+ | 5+ | 7,128 | 8,437 |
| 1264 | New Zealand | 2015-2016 | New Zealand Health Survey | National | Both | 5+ | 5+ | 7,258 | 8,451 |
| 1265 | Nicaragua | 1997-1998 | DHS | National | Both |  | 15-49 |  | 12,257 |
| 1266 | Nicaragua | 2001 | DHS | National | Both |  | 15-49 |  | 11,940 |
| 1267 | Nicaragua | 2003-2004 | CAMDI | Community | Urban | 20+ | 20+ | 773 | 916 |
| 1268 | Nicaragua | 2003-2005 | Sistema Integrado de Vigilancia de Intervenciones Nutricionales (SIVIN) | National | Both |  | 15-50 |  | 1,115 |
| 1269 | Nicaragua | 2006-2007 | Encuesta Nicaraguense de Demografia y Salud | National | Both |  | 15-49 |  | 13,216 |
| 1270 | Nicaragua | 2011-2012 | Encuesta Nicaraguense de Demografia y Salud | National | Both |  | 15-49 |  | 14,318 |
| 1271 | Niger | 1992 | DHS | National | Both |  | 20-49 |  | 2,993 |
| 1272 | Niger | 1998 | DHS | National | Both |  | 20-49 |  | 2,958 |
| 1273 | Niger | 2006 | DHS | National | Both |  | 15-49 |  | 4,151 |
| 1274 | Niger | 2007 | STEPS | National | Both | 15-64 | 15-64 | 1,430 | 1,215 |
| 1275 | Niger | 2012 | DHS | National | Both |  | 15-49 |  | 4,429 |
| 1276 | Nigeria | 1990 | Non-communicable Diseases National Survey | National | Rural | 15+ | 15+ | 3,619 | 3,682 |
| 1277 | Nigeria | 1990 | Non-communicable Diseases National Survey | National | Urban | 15+ | 15+ | 1,617 | 1,645 |
| 1278 | Nigeria | 1991-1994 | Cooper et al., Am J Public Health 87(2):160-68, 1997 | Community | Both | 20-100 | 20-100 | 910 | 1,080 |
| 1279 | Nigeria | 1999 | DHS | National | Both |  | 20-49 |  | 2,004 |
| 1280 | Nigeria | 2003 | DHS | National | Both |  | 15-49 |  | 6,605 |
| 1281 | Nigeria | 1999-2009 | Prostate cancer dietary risk factors study | Subnational | Both | 35+ |  | 627 |  |
| 1282 | Nigeria | 2007 | Ibadan Study of Ageing | Subnational | Both | 60+ | 60+ | 642 | 914 |
| 1283 | Nigeria | 2008 | DHS | National | Both |  | 15-49 |  | 28,973 |
| 1284 | Nigeria | 2008 | Ibadan Study of Ageing | Subnational | Both | 61+ | 61+ | 453 | 656 |
| 1285 | Nigeria | 2009 | Ibadan Study of Ageing | Subnational | Both | 62+ | 62+ | 420 | 619 |
| 1286 | Nigeria | 2009 | Community Health Plan - Kwara Central Survey | Community | Rural | 5+ | 5+ | 2,264 | 2,354 |
| 1287 | Nigeria | 2011 | Community Health Plan - Kwara Central Survey | Community | Rural | 5+ | 5+ | 791 | 853 |
| 1288 | Nigeria | 2013 | DHS | National | Both |  | 15-49 |  | 33,943 |
| 1289 | Nigeria | 2013 | Community Health Plan - Kwara Central Survey | Community | Rural | 5+ | 5+ | 714 | 754 |

| 1290 | Nigeria | 2018 | Hypertension Prevalence, Awareness, Treatment and Control in Rural Area, Nigeria | Community | Rural | 18+ | 18+ | 189 | 202 |
| --- | --- | --- | --- | --- | --- | --- | --- | --- | --- |
| 1291 | Niue | 2011 | STEPS | National | Both | 15+ | 15+ | 407 | 478 |
| 1292 | Norway | 1984-1986 | HUNT1 Study | Subnational | Rural | 20+ | 20+ | 36,517 | 37,811 |
| 1293 | Norway | 1986-1987 | The Tromsø Study: Tromsø 3 | Community | Both | 20-61 | 20-56 | 10,374 | 9,804 |
| 1294 | Norway | 1992-1993 | The Hordaland Health Study (HUSK) 1925-1927 birth cohort | Community | Urban | 65-67 | 65-67 | 2,123 | 2,630 |
| 1295 | Norway | 1992-1993 | The Hordaland Health Study (HUSK) 1928-1949 birth cohort | Community | Urban | 43-64 | 43-64 | 335 | 348 |
| 1296 | Norway | 1992-1993 | The Hordaland Health Study (HUSK) 1950-1952 birth cohort | Subnational | Both | 40-42 | 40-42 | 6,103 | 6,475 |
| 1297 | Norway | 1994-1995 | The Tromsø Study: Tromsø 4 | Community | Both | 25+ | 25+ | 12,782 | 13,836 |
| 1298 | Norway | 1995-1997 | HUNT2 study | Subnational | Rural | 20+ | 20+ | 30,285 | 33,599 |
| 1299 | Norway | 1995-1997 | Young-HUNT1 Study | Subnational | Rural | 12-21 | 12-21 | 4,203 | 4,253 |
| 1300 | Norway | 1997-1999 | The Hordaland Health Study (HUSK) 1925-27 birth cohort | Community | Urban | 70-74 | 70-74 | 1,465 | 1,839 |
| 1301 | Norway | 1997-1999 | The Hordaland Health Study (HUSK) 1950-1957 birth cohort | Subnational | Both | 40-47 | 40-47 | 10,180 | 11,928 |
| 1302 | Norway | 2000-2001 | Young-HUNT2 Study | Subnational | Rural | 16-21 | 16-21 | 764 | 901 |
| 1303 | Norway | 2001-2002 | The Tromsø Study: Tromsø 5, Tromsø Study Panel | Community | Both | 30-89 | 30-89 | 2,525 | 3,579 |
| 1304 | Norway | 2000-2003 | The Oslo cohort (HUBRO), the Oppland and Hedmark cohort (OPPHED), and the Troms and Finnmark cohort (TROFINN ) of Cohort Norway | Subnational | Both | 30-76 | 30-76 | 16,825 | 20,592 |
| 1305 | Norway | 2006-2008 | HUNT3 Study | Subnational | Rural | 20+ | 20+ | 22,860 | 27,553 |
| 1306 | Norway | 2006-2008 | Young-HUNT3 Study | Subnational | Rural | 12-21 | 12-21 | 3,807 | 3,792 |
| 1307 | Norway | 2007-2008 | The Tromsø Study: Tromsø 6 | Community | Both | 30-87 | 30-87 | 6,048 | 6,889 |
| 1308 | Occupied Palestinian Territory | 1996 | Stene et al., Eur J Clin Nutr 55(9):805-11, 2001 | Community | Rural | 30-65 | 30-65 | 208 | 269 |
| 1309 | Occupied Palestinian Territory | 1996-1998 | Kobar, rural | Community | Rural | 15-64 | 15-64 | 206 | 482 |
| 1310 | Occupied Palestinian Territory | 1996-1998 | Old Ramallah, urban | Community | Urban | 15-64 | 15-64 | 182 | 493 |
| 1311 | Occupied Palestinian Territory | 1999-2000 | The First National Health and Nutrition Survey | National | Both | 18-64 | 18-64 | 1,736 | 1,869 |
| 1312 | Occupied Palestinian Territory | 2010 | STEPS | National | Both | 15-64 | 15-64 | 2,578 | 4,052 |
| 1313 | Oman | 1991 | Oman National Health Survey | National | Both | 20+ | 20+ | 2,128 | 2,958 |
| 1314 | Oman | 2000 | Oman National Health Survey | National | Both | 20+ | 20+ | 3,069 | 3,331 |
| 1315 | Oman | 2001 | Al-Lawati et al., Diabetes Care 26(6):1781-85, 2003 | Community | Urban | 20+ | 20+ | 755 | 756 |
| 1316 | Oman | 2006 | STEPS | Community | Urban | 20-59 | 20-59 | 540 | 732 |
| 1317 | Oman | 2008 | Gulf Cooperation Council World Health Survey | National | Both | 18+ | 18+ | 2,389 | 2,112 |
| 1318 | Oman | 2017 | STEPS | National | Both | 15+ | 15+ | 3,405 | 3,034 |
| 1319 | Pakistan | 1990-1994 | National Health Survey Of Pakistan 1990-1994 | National | Both | 5+ | 5+ | 7,110 | 7,405 |
| 1320 | Pakistan | 1990-1994 | MHS | Community | Urban | 18+ | 18+ | 432 | 478 |
| 1321 | Pakistan | 1996 | Gupta et al., Int J Cardiol 97(2):257-61, 2004 | Community | Urban | 20+ | 20+ | 523 | 559 |
| 1322 | Pakistan | 1999 | Shah et al., Trop Med Int Health 9(4):526-32, 2004 | Community | Both | 18+ | 18+ | 1,391 | 2,754 |
| 1323 | Pakistan | 2005 | STEPS | National | Both | 25-65 | 25-65 | 787 | 1,071 |
| 1324 | Pakistan | 2004-2005 | COBRA-1 | Community | Urban | 40+ | 40+ | 1,500 | 1,635 |
| 1325 | Pakistan | 2011 | National Nutrition Survey | National | Both | 5-49 | 5-49 | 21,461 | 48,503 |
| 1326 | Pakistan | 2012-2013 | DHS | National | Both |  | 20-49 |  | 3,968 |
| 1327 | Pakistan | 2014 | STEPS | Subnational | Both | 18-69 | 18-69 | 2,964 | 3,674 |
| 1328 | Pakistan | 2016-2017 | National Diabetes Survey of Pakistan | National | Both | 20+ | 20+ | 3,771 | 4,647 |
| 1329 | Pakistan | 2017-2018 | DHS | National | Both |  | 15-49 |  | 4,493 |
| 1330 | Palau | 2011-2013 | STEPS | National | Both | 25-64 | 25-64 | 1,031 | 1,124 |
| 1331 | Palau | 2016 | STEPS | National | Both | 18+ | 18+ | 713 | 711 |
| 1332 | Panama | 2003 | Encuesta de Niveles de Vida | National | Both | 5+ | 5+ | 10,808 | 11,133 |
| 1333 | Panama | 2003 | Second Living Standards Survey | National | Both | 18-75 | 18-75 | 6,844 | 7,100 |
| 1334 | Panama | 2010-2011 | Prevalencia de factores de riesgo asociados a enfermedad cardiovascular 2010-2011 | Subnational | Both | 18+ | 18+ | 1,067 | 2,469 |
| 1335 | Papua New Guinea | 1985-1986 | INTERSALT | Community | Rural | 20-59 | 20-59 | 88 | 74 |
| 1336 | Papua New Guinea | 2007 | STEPS | National | Both | 15-64 | 15-64 | 1,401 | 1,440 |
| 1337 | Paraguay | 2011 | Primera Encuesta Nacional de Factores de Riesgo de Enfermedades No Transmisibles en Poblacion General | National | Both | 15-75 | 15-75 | 931 | 1,574 |
| 1338 | Peru | 1991-1992 | DHS | National | Both |  | 15-49 |  | 4,887 |
| 1339 | Peru | 1996 | DHS | National | Both |  | 20-49 |  | 10,125 |
| 1340 | Peru | 2000 | DHS | National | Both |  | 15-49 |  | 25,508 |
| 1341 | Peru | 2003 | Factores de Riesgo de Enfermedades No Transmisibles | Community | Urban | 16+ | 16+ | 327 | 503 |
| 1342 | Peru | 2004 | Factores de Riesgo de Enfermedades No Transmisibles | Community | Urban | 15+ | 15+ | 218 | 445 |
| 1343 | Peru | 2004-2006 | DHS | National | Both |  | 15-49 |  | 5,798 |
| 1344 | Peru | 2004-2005 | Encuesta Nacional de Indicadores Nutricionales, Bioquímicos, Socioeconómicos y Culturales Relacionados con las Enfermedades Crónicas Degenerativ | National | Both | 20+ | 20+ | 2,087 | 2,095 |
| 1345 | Peru | 2005 | Factores de Riesgo de Enfermedades No Transmisibles | Community | Urban | 15+ | 15+ | 209 | 550 |
| 1346 | Peru | 2004-2005 | PREVENCION Study | Community | Urban | 20-80 | 20-80 | 867 | 1,011 |
| 1347 | Peru | 2004-2005 | CArdiovascular Risk factors Multiple Evaluation in Latin America | Community | Urban | 25-64 | 25-64 | 769 | 876 |
| 1348 | Peru | 2006 | Factores de Riesgo de Enfermedades No Transmisibles | Community | Urban | 15+ | 15+ | 662 | 1,101 |
| 1349 | Peru | 2007-2008 | DHS | National | Both |  | 15-49 |  | 20,918 |
| 1350 | Peru | 2007-2008 | Monitoreo de Indicadores Nutricionales en la ENAHO 2007-2008 | National | Both | 5+ | 5+ | 15,041 | 16,282 |
| 1351 | Peru | 2007-2010 | Monitoreo Nacional de Indicadores Nutricionales | National | Both |  | 12-49 |  | 3,874 |
| 1352 | Peru | 2007-2008 | PERU MIGRANT Study | Community | Both | 30+ | 30+ | 464 | 522 |
| 1353 | Peru | 2009 | DHS | National | Both |  | 15-49 |  | 23,034 |
| 1354 | Peru | 2010 | DHS | National | Both |  | 15-49 |  | 22,425 |
| 1355 | Peru | 2009-2011 | Monitoreo de Indicadores Nutricionales en la ENAHO 2009-2010 | National | Both | 5+ | 5+ | 27,753 | 31,269 |
| 1356 | Peru | 2011 | DHS | National | Both |  | 15-49 |  | 22,215 |
| 1357 | Peru | 2009-2012 | CRONICAS Cohort Study | Subnational | Both | 35+ | 35+ | 1,557 | 1,660 |

| 1358 | Peru | 2012 | DHS | National | Both |  | 15-49 |  | 23,724 |
| --- | --- | --- | --- | --- | --- | --- | --- | --- | --- |
| 1359 | Peru | 2011-2012 | Monitoreo de Indicadores Nutricionales en la ENAHO 2011 | National | Both | 5+ | 5+ | 7,424 | 8,424 |
| 1360 | Peru | 2010-2013 | CRONICAS Cohort Study | Subnational | Both | 35+ | 35+ | 1,379 | 1,468 |
| 1361 | Peru | 2013 | DHS | National | Both | 15+ | 15+ | 2,932 | 23,784 |
| 1362 | Peru | 2013 | Clinical functional and sociofamilial profiles of the elderly from a community in a district of Lima, Peru | Community | Urban | 60+ | 60+ | 185 | 309 |
| 1363 | Peru | 2012-2013 | PERU MIGRANT Study | Community | Both | 35+ | 35+ | 339 | 427 |
| 1364 | Peru | 2013-2014 | CRONICAS Cohort Study | Subnational | Both | 36+ | 36+ | 1,292 | 1,361 |
| 1365 | Peru | 2014 | DHS | National | Both | 15+ | 15+ | 12,670 | 28,582 |
| 1366 | Peru | 2014 | Launching a salt substitute to reduce blood pressure at the population level: a cluster randomized stepped wedge trial in Peru | Subnational | Both | 18+ | 18+ | 1,149 | 1,166 |
| 1367 | Peru | 2015 | DHS | National | Both | 15+ | 15+ | 14,744 | 38,415 |
| 1368 | Peru | 2016 | DHS | National | Both | 15+ | 15+ | 14,037 | 35,999 |
| 1369 | Peru | 2015-2016 | PERU MIGRANT Study | Community | Both | 38+ | 38+ | 324 | 414 |
| 1370 | Peru | 2017 | DHS | National | Both | 15+ | 15+ | 14,340 | 37,386 |
| 1371 | Peru | 2016-2017 | Sceening of T2DM | Community | Urban | 30-70 | 30-70 | 798 | 809 |
| 1372 | Peru | 2018 | DHS | National | Both | 15+ | 15+ | 14,600 | 39,575 |
| 1373 | Peru | 2019 | DHS | National | Both | 15+ | 15+ | 14,200 | 37,943 |
| 1374 | Philippines | 1985-1986 | Cebu Longitudinal Health and Nutrition Survey Baseline 20-Month Follow-up | Community | Both |  | 15-50 |  | 2,047 |
| 1375 | Philippines | 1985-1986 | Cebu Longitudinal Health and Nutrition Survey Baseline 22-Month Follow-up | Community | Both |  | 15-50 |  | 2,017 |
| 1376 | Philippines | 1985-1986 | Cebu Longitudinal Health and Nutrition Survey Baseline 24-Month Follow-up | Community | Both |  | 15-50 |  | 2,022 |
| 1377 | Philippines | 1984-1985 | Cebu Longitudinal Health and Nutrition Survey Baseline 16-Month Follow-up | Community | Both |  | 15-50 |  | 2,129 |
| 1378 | Philippines | 1984-1985 | Cebu Longitudinal Health and Nutrition Survey Baseline 18-Month Follow-up | Community | Both |  | 15-50 |  | 2,079 |
| 1379 | Philippines | 1988 | INCLEN | Community | Rural | 35-65 |  | 274 |  |
| 1380 | Philippines | 1991-1992 | Cebu Longitudinal Health and Nutrition Survey 1991 Mother Follow-up | Community | Both |  | 22-55 |  | 2,195 |
| 1381 | Philippines | 1993 | 4th National Nutrition Survey Philippine | National | Both | 20-70 | 20-70 | 4,383 | 4,754 |
| 1382 | Philippines | 1993 | National Safe Motherhood Survey | National | Both |  | 15-49 |  | 7,181 |
| 1383 | Philippines | 1994-1995 | Cebu Longitudinal Health and Nutrition Survey 1994-1995 Mother Follow-up | Community | Both |  | 15-59 |  | 2,692 |
| 1384 | Philippines | 1998 | 5th National Nutrition Survey Philippine | National | Both | 20-60 | 20-60 | 1,323 | 1,340 |
| 1385 | Philippines | 1998-1999 | Cebu Longitudinal Health and Nutrition Survey 1998-1999 Mother Follow-up | Community | Both |  | 15-59 |  | 1,911 |
| 1386 | Philippines | 2002 | Cebu Longitudinal Health and Nutrition Survey 2002 Mother Follow-up | Community | Both |  | 32-66 |  | 2,080 |
| 1387 | Philippines | 2003 | 6th National Nutrition Survey Philippine | National | Both | 5+ | 5+ | 10,686 | 11,131 |
| 1388 | Philippines | 2003-2004 | National Nutrition and Health Survey | National | Both | 15+ | 15+ | 30,231 | 33,295 |
| 1389 | Philippines | 2005 | Cebu Longitudinal Health and Nutrition Survey 2005 Child Follow-up | Community | Both | 20-22 | 20-22 | 1,006 | 831 |
| 1390 | Philippines | 2005 | Cebu Longitudinal Health and Nutrition Survey 2005 Mother Follow-up | Community | Both |  | 35-69 |  | 2,001 |
| 1391 | Philippines | 2007 | Cebu Longitudinal Health and Nutrition Survey 2007 Child Follow-up | Community | Both | 23-24 | 23-24 | 937 | 751 |
| 1392 | Philippines | 2007 | Cebu Longitudinal Health and Nutrition Survey 2007 Mother Follow-up | Community | Both |  | 38-71 |  | 1,925 |
| 1393 | Philippines | 2008 | 7th National Nutrition Survey | National | Both | 5+ | 5+ | 64,001 | 63,616 |
| 1394 | Philippines | 2009 | Cebu Longitudinal Health and Nutrition Survey 2009 Child Follow-up | Community | Both | 24-26 | 24-26 | 864 | 718 |
| 1395 | Philippines | 2011 | 2011 Updating of Nutritional Status of Filipino Children | National | Both | 5+ | 5+ | 63,654 | 66,866 |
| 1396 | Philippines | 2013-2014 | 8th National Nutrition Survey | National | Both | 5+ | 5+ | 57,432 | 61,620 |
| 1397 | Philippines | 2015 | 2015 Updating of Nutritional Status of Filipino Children and Other Population Groups | National | Both | 5+ | 5+ | 69,309 | 73,250 |
| 1398 | Poland | 1986 | INTERSALT, Krakow | Community | Urban | 20-59 | 20-59 | 100 | 100 |
| 1399 | Poland | 1986 | INTERSALT, Warsaw | Community | Urban | 20-59 | 20-59 | 100 | 100 |
| 1400 | Poland | 1987-1988 | MONICA, Tarnobrzeg Voivodship | Community | Rural | 35-64 | 35-64 | 616 | 672 |
| 1401 | Poland | 1988-1989 | MONICA, Warsaw | Community | Urban | 35-64 | 35-64 | 705 | 713 |
| 1402 | Poland | 1989-1990 | Polish Program CINDI (CINDI Lodz 1989-1990) | Community | Urban | 25-64 | 25-64 | 831 | 957 |
| 1403 | Poland | 1992-1993 | MONICA, Tarnobrzeg Voivodship | Community | Rural | 35-64 | 35-64 | 618 | 692 |
| 1404 | Poland | 1993 | MONICA, Warsaw | Community | Urban | 35-64 | 35-64 | 751 | 763 |
| 1405 | Poland | 1995-1996 | Polish Program CINDI (CINDI Lodz 1995) | Community | Urban | 17-64 | 17-64 | 997 | 1,459 |
| 1406 | Poland | 2000 | The health status, risk factors of chronic diseases and health behaviors of residents of Torun (CINDI Torun 2000) | Community | Urban | 16-83 | 16-83 | 989 | 1,054 |
| 1407 | Poland | 2000-2001 | Household Food Consumption and Anthropometric Survey | National | Both | 5+ | 5+ | 1,766 | 2,107 |
| 1408 | Poland | 2001-2002 | The health status, risk factors of chronic diseases and health behaviors of residents of Lodz (CINDI Lodz 2001) | Community | Urban | 18-64 | 18-64 | 1,000 | 840 |
| 1409 | Poland | 2002 | The health status, risk factors of chronic diseases and health behaviors of residents of Lodz - seniors (CINDI Lodz 2002) | Community | Urban | 65+ | 65+ | 285 | 532 |
| 1410 | Poland | 2002 | NATPOL | National | Both | 18+ | 18+ | 1,018 | 1,301 |
| 1411 | Poland | 2003 | The European Male Ageing Study | Community | Both | 40+ |  | 406 |  |
| 1412 | Poland | 2004 | LIPIDOGRAM2004 Study - National epidemiological study of lipid disorders and selected risk factors of cardiovascular disease in primary health care i | National | Both | 30+ | 30+ | 6,673 | 9,920 |
| 1413 | Poland | 2003-2005 | National Multicenter Health Survey in Poland. Project WOBASZ | National | Both | 20-74 | 20-74 | 6,245 | 6,910 |
| 1414 | Poland | 2002-2005 | Health, Alcohol and Psychosocial Factors In Eastern Europe | Community | Urban | 45-70 | 45-70 | 4,502 | 4,752 |
| 1415 | Poland | 2003-2006 | Mogielica Human Ecology Study Site | Community | Rural | 18+ | 18+ | 119 | 321 |
| 1416 | Poland | 2006 | The health, risk factors for chronic diseases, attitudes and behaviors of health residents of Torun (CINDI Torun 2006) | Community | Urban | 15-65 | 15-65 | 790 | 1,147 |
| 1417 | Poland | 2006 | LIPIDOGRAM2006 Study - National epidemiological study of lipid disorders and selected risk factors of cardiovascular disease in primary health care i | National | Both | 32+ | 32+ | 6,441 | 10,640 |
| 1418 | Poland | 2008 | The European Male Ageing Study | Community | Both | 40+ |  | 310 |  |
| 1419 | Poland | 2007-2010 | Mogielica Human Ecology Study Site | Community | Rural | 18+ | 18+ | 133 | 290 |
| 1420 | Poland | 2007-2011 | Medical, psychological and socioeconomic aspects of aging in Poland | National | Both | 55+ | 55+ | 2,750 | 2,582 |
| 1421 | Poland | 2011 | NATPOL | National | Both | 18-79 | 18-79 | 1,158 | 1,235 |
| 1422 | Poland | 2011-2014 | Mogielica Human Ecology Study Site | Community | Rural | 18+ | 18+ | 142 | 418 |
| 1423 | Poland | 2013-2014 | National Multicenter Health Survey in Poland. Project WOBASZ II | National | Both | 20+ | 20+ | 2,626 | 3,198 |
| 1424 | Poland | 2015-2016 | LIPIDOGRAM2015 & LIPIDOGEN2015 Study - National epidemiological study of lipid disorders and selected risk factors of cardiovascular disease in | National | Both | 18+ | 18+ | 5,034 | 8,690 |
| 1425 | Poland | 2018 | Mogielica Human Ecology Study Site | Community | Rural | 18+ | 18+ | 30 | 95 |

| 1426 | Portugal | 1985 | Body Mass Index of Portuguese Conscripts | National | Both | 18-20 |  | 29,420 |  |
| --- | --- | --- | --- | --- | --- | --- | --- | --- | --- |
| 1427 | Portugal | 1986 | Body Mass Index of Portuguese Conscripts | National | Both | 18-20 |  | 70,504 |  |
| 1428 | Portugal | 1986 | INTERSALT | Community | Rural | 20-59 | 20-59 | 99 | 99 |
| 1429 | Portugal | 1987 | Body Mass Index of Portuguese Conscripts | National | Both | 18-20 |  | 68,079 |  |
| 1430 | Portugal | 1988 | Body Mass Index of Portuguese Conscripts | National | Both | 18-20 |  | 67,573 |  |
| 1431 | Portugal | 1989 | Body Mass Index of Portuguese Conscripts | National | Both | 18-20 |  | 68,827 |  |
| 1432 | Portugal | 1990 | Body Mass Index of Portuguese Conscripts | National | Both | 18-20 |  | 44,359 |  |
| 1433 | Portugal | 1991 | Body Mass Index of Portuguese Conscripts | National | Both | 18-20 |  | 19,552 |  |
| 1434 | Portugal | 1992 | Body Mass Index of Portuguese Conscripts | National | Both | 18-20 |  | 52,393 |  |
| 1435 | Portugal | 1993 | Body Mass Index of Portuguese Conscripts | National | Both | 18-20 |  | 59,780 |  |
| 1436 | Portugal | 1994 | Body Mass Index of Portuguese Conscripts | National | Both | 18-20 |  | 55,511 |  |
| 1437 | Portugal | 1995 | Body Mass Index of Portuguese Conscripts | National | Both | 18-20 |  | 68,221 |  |
| 1438 | Portugal | 1996 | Body Mass Index of Portuguese Conscripts | National | Both | 18-21 |  | 106,097 |  |
| 1439 | Portugal | 1997 | Body Mass Index of Portuguese Conscripts | National | Both | 18-21 |  | 61,215 |  |
| 1440 | Portugal | 1998 | Body Mass Index of Portuguese Conscripts | National | Both | 18-21 |  | 41,027 |  |
| 1441 | Portugal | 1999 | Body Mass Index of Portuguese Conscripts | National | Both | 18-21 |  | 54,187 |  |
| 1442 | Portugal | 2000 | Body Mass Index of Portuguese Conscripts | National | Both | 18-21 |  | 53,326 |  |
| 1443 | Portugal | 1999-2003 | EPIPorto Study | Community | Urban | 18+ | 18+ | 932 | 1,507 |
| 1444 | Portugal | 2003-2005 | Estudo de Prevalência da Obesidade e Consumos Alimentares em Portugal | National | Both | 18-64 | 18-64 | 3,796 | 4,320 |
| 1445 | Portugal | 2007-2009 | Portuguese National Survey of Physical Activity and Physical Fitness | National | Both | 9+ | 9+ | 14,914 | 18,025 |
| 1446 | Portugal | 2010-2012 | Exercise for Elderly | Community | Urban | 60-84 | 60-84 | 48 | 104 |
| 1447 | Portugal | 2011-2013 | EPITeen - Epidemiological Health Investigation of Teenagers in Porto | Community | Urban | 20-23 | 20-23 | 854 | 895 |
| 1448 | Puerto Rico | 2002-2003 | Puerto Rican Elderly: Health Conditions | National | Both | 60+ | 60+ | 1,914 | 2,850 |
| 1449 | Puerto Rico | 2005-2007 | Perez et al., Ethn Dis 18(4):434-41, 2008 | Community | Urban | 15-84 | 15-84 | 275 | 529 |
| 1450 | Puerto Rico | 2006-2007 | Puerto Rican Elderly: Health Conditions | National | Both | 60+ | 60+ | 1,056 | 1,669 |
| 1451 | Puerto Rico | 2010-2013 | HPV Infection in a Population-Based Sample of Puerto Rican Women | Subnational | Both |  | 16-64 |  | 563 |
| 1452 | Qatar | 2006 | World Health Survey | National | Both | 18+ | 18+ | 1,859 | 2,018 |
| 1453 | Qatar | 2012 | STEPS | National | Both | 18-64 | 18-64 | 1,034 | 1,353 |
| 1454 | Romania | 1986-1987 | MONICA, Bucharest | Community | Urban | 25-64 | 25-64 | 702 | 873 |
| 1455 | Romania | 1997 | Somatometria | National | Both | 15-75 | 15-75 | 3,142 | 4,063 |
| 1456 | Romania | 2009-2011 | Study on children in Dolj County, South Romania | Subnational | Both | 5-21 | 5-21 | 746 | 672 |
| 1457 | Romania | 2011-2012 | Study for the Evaluation of Prevalence of Hypertension and cArdiovascular Risk among the Adult Population of Romania - SEPHAR II | National | Both | 18-80 | 18-80 | 927 | 1,023 |
| 1458 | Romania | 2015-2016 | Study for the Evaluation of Prevalence of Hypertension and cArdiovascular Risk among the Adult Population of Romania - SEPHAR III | National | Both | 18-80 | 18-80 | 936 | 1,034 |
| 1459 | Russian Federation | 1984-1986 | MONICA, Moscow (control) | Community | Urban | 35-64 | 35-64 | 774 | 642 |
| 1460 | Russian Federation | 1984-1986 | MONICA, Moscow, Leninsky district | Community | Urban | 35-64 | 35-64 | 553 | 622 |
| 1461 | Russian Federation | 1984-1986 | MONICA, Moscow, Cheremushkinsky district | Community | Urban | 35-64 | 35-64 | 580 | 579 |
| 1462 | Russian Federation | 1985 | MONICA, Novosibirsk (intervention) | Community | Urban | 25-64 | 25-64 | 797 | 818 |
| 1463 | Russian Federation | 1986 | INTERSALT | Community | Urban | 20-59 | 20-59 | 97 | 97 |
| 1464 | Russian Federation | 1985-1986 | MONICA, Novosibirsk, Kirowsky district | Community | Urban | 25-64 | 25-64 | 758 | 774 |
| 1465 | Russian Federation | 1985-1986 | MONICA, Novosibirsk, Leninsky district | Community | Urban | 25-64 | 25-64 | 624 | 624 |
| 1466 | Russian Federation | 1988 | MONICA, Novosibirsk (intervention) | Community | Urban | 25-64 | 25-64 | 837 | 852 |
| 1467 | Russian Federation | 1988-1989 | MONICA, Moscow (control) | Community | Urban | 35-64 | 35-64 | 620 | 581 |
| 1468 | Russian Federation | 1988-1989 | MONICA, Moscow, Leninsky district | Community | Urban | 35-64 | 35-64 | 597 | 612 |
| 1469 | Russian Federation | 1988-1989 | MONICA, Novosibirsk, Kirowsky district | Community | Urban | 25-64 | 25-64 | 871 | 705 |
| 1470 | Russian Federation | 1992 | Russian Karelia Survey in Pitkaranta | Community | Both | 25-64 | 25-64 | 380 | 455 |
| 1471 | Russian Federation | 1992 | CINDI | Community | Rural | 25-64 | 25-64 | 377 | 453 |
| 1472 | Russian Federation | 1992-1993 | Russia Longitudinal Monitoring Survey-Higher School of Economics Round II | National | Both | 5+ | 5+ | 4,764 | 6,348 |
| 1473 | Russian Federation | 1993 | Russia Longitudinal Monitoring Survey-Higher School of Economics Round III | National | Both | 5+ | 5+ | 6,009 | 7,685 |
| 1474 | Russian Federation | 1993-1994 | Russia Longitudinal Monitoring Survey-Higher School of Economics Round IV | National | Both | 5+ | 5+ | 5,519 | 7,094 |
| 1475 | Russian Federation | 1992-1995 | MONICA, Moscow (control) | Community | Urban | 35-64 | 35-64 | 556 | 527 |
| 1476 | Russian Federation | 1992-1995 | MONICA, Moscow, Leninsky district | Community | Urban | 35-64 | 35-64 | 538 | 858 |
| 1477 | Russian Federation | 1994 | Russia Longitudinal Monitoring Survey-Higher School of Economics Round V | National | Both | 5+ | 5+ | 4,726 | 5,788 |
| 1478 | Russian Federation | 1994-1995 | MONICA, Novosibirsk (intervention) | Community | Urban | 25-64 | 25-64 | 820 | 860 |
| 1479 | Russian Federation | 1995 | Russia Longitudinal Monitoring Survey-Higher School of Economics Round VI | National | Both | 5+ | 5+ | 4,463 | 5,509 |
| 1480 | Russian Federation | 1995 | MONICA, Novosibirsk, Kirowsky district | Community | Urban | 25-64 | 25-64 | 771 | 787 |
| 1481 | Russian Federation | 1996 | Russia Longitudinal Monitoring Survey-Higher School of Economics Round VII | National | Both | 5+ | 5+ | 4,377 | 5,456 |
| 1482 | Russian Federation | 1997 | Russian Karelia Survey in Pitkaranta | Community | Both | 25-64 | 25-64 | 309 | 440 |
| 1483 | Russian Federation | 1998-1999 | Russia Longitudinal Monitoring Survey-Higher School of Economics Round VIII | National | Both | 5+ | 5+ | 4,497 | 5,590 |
| 1484 | Russian Federation | 2000 | Russia Longitudinal Monitoring Survey-Higher School of Economics Round IX | National | Both | 5+ | 5+ | 4,600 | 5,760 |
| 1485 | Russian Federation | 2001 | Russia Longitudinal Monitoring Survey-Higher School of Economics Round X | National | Both | 5+ | 5+ | 5,023 | 6,440 |
| 1486 | Russian Federation | 2002 | Russian Karelia Survey in Pitkaranta | Community | Both | 25-64 | 25-64 | 251 | 334 |
| 1487 | Russian Federation | 2002 | Russia Longitudinal Monitoring Survey-Higher School of Economics Round XI | National | Both | 5+ | 5+ | 5,199 | 6,604 |
| 1488 | Russian Federation | 2003 | Russia Longitudinal Monitoring Survey-Higher School of Economics Round XII | National | Both | 5+ | 5+ | 5,250 | 6,674 |
| 1489 | Russian Federation | 2004 | Russia Longitudinal Monitoring Survey-Higher School of Economics Round XIII | National | Both | 5+ | 5+ | 5,219 | 6,668 |
| 1490 | Russian Federation | 2002-2005 | Health, Alcohol and Psychosocial Factors In Eastern Europe | Community | Urban | 43-73 | 43-73 | 4,259 | 5,086 |
| 1491 | Russian Federation | 2005 | Russia Longitudinal Monitoring Survey-Higher School of Economics Round XIV | National | Both | 5+ | 5+ | 5,027 | 6,453 |
| 1492 | Russian Federation | 2007 | Russian Karelia Survey in Pitkaranta | Community | Both | 25-64 | 25-64 | 176 | 276 |
| 1493 | Russian Federation | 2007-2010 | SAGE | National | Both | 50+ | 50+ | 1,254 | 2,251 |

| 1494 | Russian Federation | 2015-2017 | Ural Eye and Medical Study (UEMS) | Subnational | Rural | 40+ | 40+ | 1,530 | 1,870 |
| --- | --- | --- | --- | --- | --- | --- | --- | --- | --- |
| 1495 | Russian Federation | 2015-2017 | Ural Eye and Medical Study (UEMS) | Community | Urban | 40+ | 40+ | 1,050 | 1,449 |
| 1496 | Rwanda | 2000 | DHS | National | Both |  | 15-49 |  | 9,175 |
| 1497 | Rwanda | 2005 | DHS | National | Both |  | 15-49 |  | 5,211 |
| 1498 | Rwanda | 2010 | DHS | National | Both | 15-59 | 15-49 | 6,472 | 6,572 |
| 1499 | Rwanda | 2012 | STEPS | National | Both | 15-64 | 15-64 | 2,644 | 4,243 |
| 1500 | Rwanda | 2014-2015 | DHS | National | Both | 15-59 | 15-49 | 6,366 | 6,313 |
| 1501 | Saint Kitts and Nevis | 2007 | STEPS | Subnational | Both | 25-64 | 25-64 | 510 | 852 |
| 1502 | Saint Lucia | 1991-1994 | Cooper et al., Am J Public Health 87(2):160-68, 1997 | Community | Urban | 25-100 | 25-100 | 491 | 593 |
| 1503 | Saint Lucia | 2012 | STEPS | National | Both | 25-64 | 25-64 | 665 | 1,097 |
| 1504 | Saint Vincent and the Grenadine | 2013-2014 | STEPS | National | Both | 18-69 | 18-69 | 1,524 | 1,897 |
| 1505 | Samoa | 1991 | McGarvey, Pac Health Dialog 8(1):157-62, 2001 | National | Both | 25+ | 25+ | 347 | 381 |
| 1506 | Samoa | 1993 | McGarvey, Pac Health Dialog 8(1):157-62, 2002 | National | Both | 27+ | 27+ | 285 | 336 |
| 1507 | Samoa | 1995 | McGarvey, Pac Health Dialog 8(1):157-62, 2001 | National | Both | 29+ | 29+ | 156 | 157 |
| 1508 | Samoa | 2002 | STEPS | National | Both | 25-64 | 25-64 | 1,181 | 1,334 |
| 1509 | Samoa | 2010 | Samoan Genome-Wide Association Study | National | Both | 24-65 | 24-65 | 1,402 | 2,061 |
| 1510 | Samoa | 2013 | STEPS | National | Both | 18-64 | 18-64 | 605 | 918 |
| 1511 | Sao Tome and Principe | 2008-2009 | DHS | National | Both | 15-59 | 15-49 | 2,173 | 2,238 |
| 1512 | Sao Tome and Principe | 2009 | STEPS | National | Both | 25-64 | 25-64 | 998 | 1,286 |
| 1513 | Saudi Arabia | 1985-1988 | National Nutrition Survey | National | Both | 5-75 | 5-75 | 4,356 | 5,944 |
| 1514 | Saudi Arabia | 1990-1993 | National Epidemiological Household Survey | National | Both | 15-60 | 15-60 | 4,882 | 4,509 |
| 1515 | Saudi Arabia | 1989-1994 | National Nutrition Survey | National | Both | 18-40 | 18-40 | 2,481 | 3,294 |
| 1516 | Saudi Arabia | 1990-1993 | Saudi National Survey | National | Both | 30-70 | 30-70 | 1,612 | 1,648 |
| 1517 | Saudi Arabia | 1992-1995 | Saudi Health Information Survey | National | Both | 14-50 | 14-50 | 4,830 | 7,707 |
| 1518 | Saudi Arabia | 1995 | National Household Survey | National | Both | 20-70 | 20-70 | 7,121 | 7,074 |
| 1519 | Saudi Arabia | 1995-2000 | National Epidemiological Health Survey | National | Both | 30-70 | 30-70 | 8,215 | 9,008 |
| 1520 | Saudi Arabia | 2004-2005 | Al-Baghli et al., Saudi Med J 29(9):1319-25, 2008 | Subnational | Both | 30+ | 30+ | 97,254 | 97,254 |
| 1521 | Saudi Arabia | 2005 | STEPS | National | Both | 15-64 | 15-64 | 2,245 | 2,345 |
| 1522 | Saudi Arabia | 2007 | Gulf Cooperation Council World Health Survey | National | Both | 18+ | 18+ | 4,854 | 3,610 |
| 1523 | Saudi Arabia | 2011-2013 | Jeddah City Study | Community | Urban | 5+ | 5+ | 957 | 867 |
| 1524 | Saudi Arabia | 2013 | Saudi Health Information Survey | National | Both | 15+ | 15+ | 5,088 | 5,249 |
| 1525 | Senegal | 1986 | Astagneau et al., J Hypertens 10(9):1095-101, 1992 | Community | Urban | 15+ | 15+ | 651 | 707 |
| 1526 | Senegal | 1986 | Maire et al., Rev Epidemiol Sante Publique 40:252-58, 1992 | Community | Urban |  | 16-45 |  | 616 |
| 1527 | Senegal | 1992-1993 | DHS | National | Both |  | 20-49 |  | 2,713 |
| 1528 | Senegal | 2003 | Perceptions of healthy and desirable body size in urban Senegalese women | Community | Urban |  | 20-50 |  | 287 |
| 1529 | Senegal | 2005 | DHS | National | Both |  | 15-49 |  | 4,166 |
| 1530 | Senegal | 2010-2011 | DHS | National | Both | 15-59 | 15-49 | 4,715 | 5,497 |
| 1531 | Senegal | 2010-2012 | Biocultural determinants of overweight and obesity in the context of nutrition transition in Senegal: a holistic anthropological approach | Subnational | Both | 18+ | 18+ | 280 | 307 |
| 1532 | Senegal | 2015 | Les maladies chroniques au Sénégal: Une écologie de la santé comparative entre Dakar et Widou Thiengoly | Community | Both | 20-100 | 20-100 | 734 | 765 |
| 1533 | Serbia | 1988-1989 | MONICA, Novi Sad | Community | Urban | 25-64 | 25-64 | 778 | 791 |
| 1534 | Serbia | 1994-1995 | MONICA, Novi Sad | Community | Urban | 25-64 | 25-64 | 600 | 670 |
| 1535 | Serbia | 2000 | Health Status, Health Needs and Utilization of Health Care of the Population of Serbia | National | Both | 7+ | 7+ | 5,079 | 6,189 |
| 1536 | Serbia | 2006 | The 2006 National Health Survey for the Population of Serbia | National | Both | 7+ | 7+ | 7,888 | 8,558 |
| 1537 | Serbia | 2013 | The National Health Survey of the Republic of Serbia, 2013 | National | Both | 7+ | 7+ | 7,205 | 8,140 |
| 1538 | Serbia | 2013-2014 | Stay Fit for Lifelong Health; the Prevalence of Lifestyle Health Conditions in Serbian Population | National | Urban | 18-65 |  | 1,366 |  |
| 1539 | Seychelles | 1989 | Seychelles Heart Survey I | National | Both | 25-64 | 25-64 | 513 | 568 |
| 1540 | Seychelles | 1994 | Seychelles Heart Survey II | National | Both | 25-64 | 25-64 | 499 | 563 |
| 1541 | Seychelles | 2004 | Seychelles Heart Survey III | National | Both | 25-64 | 25-64 | 568 | 687 |
| 1542 | Seychelles | 2013-2014 | Seychelles Heart Survey IV | National | Both | 25-64 | 25-64 | 531 | 699 |
| 1543 | Sierra Leone | 2008 | DHS | National | Both |  | 15-49 |  | 3,274 |
| 1544 | Sierra Leone | 2009 | STEPS | National | Both | 25-64 | 25-64 | 2,200 | 2,319 |
| 1545 | Sierra Leone | 2013 | DHS | National | Both | 15-59 | 15-49 | 7,037 | 7,459 |
| 1546 | Singapore | 1992 | National Health Survey 1992 | National | Both | 18-64 | 18-64 | 1,743 | 1,704 |
| 1547 | Singapore | 1993-1995 | NUH Heart Study | National | Both | 26-89 | 26-89 | 498 | 484 |
| 1548 | Singapore | 1998 | National Health Survey 1998 | National | Both | 18-69 | 18-69 | 2,284 | 2,265 |
| 1549 | Singapore | 2004 | National Health Survey 2004 | National | Both | 18-74 | 18-74 | 2,059 | 2,095 |
| 1550 | Singapore | 2004-2007 | Combined follow up of Singapore Cardiovascular Cohort Study and Singapore Prospective Study | National | Both | 24+ | 24+ | 2,471 | 2,686 |
| 1551 | Singapore | 2009 | Social Isolation, Health and Lifestyles Survey (SIHLS) 2009 | National | Both | 60+ | 60+ | 2,038 | 2,382 |
| 1552 | Singapore | 2009-2011 | The Singapore Chinese Eye Study | Community | Both | 40-80 | 40-80 | 1,652 | 1,679 |
| 1553 | Singapore | 2012-2013 | Singapore Health Study 2012 | National | Both | 18-79 | 18-79 | 956 | 1,026 |
| 1554 | Singapore | 2014-2015 | Singapore Health 2 | National | Urban | 18-80 | 18-80 | 781 | 970 |
| 1555 | Singapore | 2016-2017 | Transitions in Health, Employment, Social Engagement and Inter-generational Transfers in Singapore Study | National | Urban | 60+ | 60+ | 1,723 | 2,131 |
| 1556 | Slovakia | 1993 | Countrywide Integrated Noncommunicable Diseases Intervention Programme | National | Both | 15-64 | 15-64 | 876 | 1,293 |
| 1557 | Slovakia | 1998 | Countrywide Integrated Noncommunicable Diseases Intervention Programme | National | Both | 15-64 | 15-64 | 923 | 1,122 |
| 1558 | Slovakia | 2003 | Countrywide Integrated Noncommunicable Diseases Intervention Programme | National | Both | 15-64 | 15-64 | 664 | 905 |
| 1559 | Slovakia | 2008 | Countrywide Integrated Noncommunicable Diseases Intervention Programme | National | Both | 15-64 | 15-64 | 412 | 584 |
| 1560 | Slovakia | 2011-2012 | European Health Examination Survey | National | Both | 18-64 | 18-64 | 884 | 1,080 |
| 1561 | Slovenia | 2014 | the SLOFIT monitoring system | National | Both | 6-21 | 6-21 | 102,790 | 97,859 |

| 1562 | Solomon Islands | 2004 | A genetic-ecological study of the risk factors for lifestyle-related diseases in Oceanian populations | Community | Rural | 18-74 | 18-74 | 106 | 109 |
| --- | --- | --- | --- | --- | --- | --- | --- | --- | --- |
| 1563 | Solomon Islands | 2004 | A genetic-ecological study of the risk factors for lifestyle-related diseases in Oceanian populations | Community | Urban | 18-79 | 18-79 | 91 | 95 |
| 1564 | Solomon Islands | 2006 | STEPS | Subnational | Both | 15-64 | 15-64 | 1,031 | 1,375 |
| 1565 | Solomon Islands | 2009-2010 | Furusawa et al., N Z Med J 124(1333):17-28, 2011 | Subnational | Rural | 5+ | 5+ | 256 | 317 |
| 1566 | Solomon Islands | 2009-2010 | Furusawa et al., N Z Med J 124(1333):17-28, 2011 | Subnational | Urban | 5-70 | 5-70 | 78 | 118 |
| 1567 | Solomon Islands | 2015 | STEPS | National | Both | 18-69 | 18-69 | 816 | 978 |
| 1568 | Somalia | 2016 | The prevalence of selected risk factors for non-communicable diseases in Hargeisa, Somaliland: a cross-sectional study | Community | Urban | 20-69 | 20-69 | 145 | 955 |
| 1569 | South Africa | 1989 | Temple et al., Ethn Dis 11(3):431-7, 2001 | Community | Both | 15+ | 15+ | 457 | 614 |
| 1570 | South Africa | 1990 | Steyn et al., East Afr Med J 75(1):35-40, 1998 | Community | Urban | 15-64 | 15-64 | 292 | 373 |
| 1571 | South Africa | 1996 | Temple et al., Ethn Dis 11(3):431-7, 2001 | Community | Both | 15+ | 15+ | 302 | 406 |
| 1572 | South Africa | 1998 | DHS | National | Both | 15+ | 15+ | 5,645 | 7,757 |
| 1573 | South Africa | 2003 | DHS | National | Both | 15+ | 15+ | 3,200 | 4,497 |
| 1574 | South Africa | 2002-2003 | SASPI | Community | Rural | 35+ | 35+ | 80 | 275 |
| 1575 | South Africa | 2003-2004 | Africa Centre Biomeasure Survey | Community | Rural | 25-49 | 25-49 | 778 | 1,693 |
| 1576 | South Africa | 2004-2006 | Li et al., Curationis 30(4):79-87, 2007 | Community | Both | 18-40 | 18-40 | 334 | 270 |
| 1577 | South Africa | 2008 | National Income Dynamics Study Wave I | National | Both | 5+ | 5+ | 8,131 | 10,624 |
| 1578 | South Africa | 2007-2008 | SAGE | National | Both | 50+ | 50+ | 1,541 | 2,059 |
| 1579 | South Africa | 2008-2009 | Cape Town Bellville South Cohort Study - Baseline evaluation I | Community | Urban | 35-65 | 35-65 | 142 | 499 |
| 1580 | South Africa | 2010 | Africa Centre Biomeasure Survey | Community | Rural | 15+ | 15+ | 2,933 | 6,364 |
| 1581 | South Africa | 2010-2011 | National Income Dynamics Study Wave II | National | Both | 5+ | 5+ | 8,383 | 10,996 |
| 1582 | South Africa | 2012 | National Income Dynamics Study Wave III | National | Both | 5+ | 5+ | 10,956 | 14,118 |
| 1583 | South Africa | 2012 | South African National Health and Nutrition Examination Survey | National | Both | 5+ | 5+ | 2,274 | 4,320 |
| 1584 | South Africa | 2014-2015 | Health and Aging in Africa: A Longitudinal Study of an INDEPTH Community in South Africa (HAALSI) | Community | Rural | 40+ | 40+ | 2,141 | 2,502 |
| 1585 | South Africa | 2014-2015 | National Income Dynamics Study Wave IV | National | Both | 5+ | 5+ | 13,548 | 16,775 |
| 1586 | South Africa | 2016 | DHS | National | Both | 15-59 | 15-49 | 2,807 | 3,263 |
| 1587 | South Africa | 2017 | National Income Dynamics Study Wave V | National | Both | 5+ | 5+ | 13,997 | 17,688 |
| 1588 | South Korea | 1986 | INTERSALT | Community | Urban | 20-59 | 20-59 | 100 | 98 |
| 1589 | South Korea | 1990 | Korean National Blood Pressure Survey | National | Both | 30+ | 30+ | 9,734 | 12,620 |
| 1590 | South Korea | 1992-1993 | Park et al., Diabetes Res Clin Pract 34 Suppl:S65-72, 1996 | Subnational | Both | 30-89 | 30+ | 1,077 | 1,392 |
| 1591 | South Korea | 1998 | Korea National Health and Nutrition Examination Survey | National | Both | 10+ | 10+ | 4,514 | 5,193 |
| 1592 | South Korea | 2001 | Korea National Health and Nutrition Examination Survey | National | Both | 5+ | 5+ | 4,150 | 4,815 |
| 1593 | South Korea | 2001 | Kim et al., Br J Psychiatry 185:102-7, 2004 | Community | Both | 65+ | 65+ | 300 | 432 |
| 1594 | South Korea | 2002-2003 | Korean National Health Insurance | National | Both | 40+ | 40+ | 2,993,634 | 2,483,306 |
| 1595 | South Korea | 2005 | Korea National Health and Nutrition Examination Survey | National | Both | 5+ | 5+ | 3,183 | 3,896 |
| 1596 | South Korea | 2004-2005 | Korean National Health Insurance | National | Both | 40+ | 40+ | 3,604,097 | 3,261,164 |
| 1597 | South Korea | 2007 | Korea National Health and Nutrition Examination Survey | National | Both | 5+ | 5+ | 1,753 | 2,174 |
| 1598 | South Korea | 2006-2007 | Korean National Health Insurance | National | Both | 40+ | 40+ | 4,569,655 | 4,613,826 |
| 1599 | South Korea | 2008 | Korea National Health and Nutrition Examination Survey | National | Both | 5+ | 5+ | 3,849 | 4,824 |
| 1600 | South Korea | 2009 | Korea National Health and Nutrition Examination Survey | National | Both | 5+ | 5+ | 4,288 | 5,182 |
| 1601 | South Korea | 2008-2009 | Korean National Health Insurance | National | Both | 40+ | 40+ | 5,763,909 | 6,089,441 |
| 1602 | South Korea | 2010 | Korea National Health and Nutrition Examination Survey | National | Both | 5+ | 5+ | 3,583 | 4,312 |
| 1603 | South Korea | 2010 | Korea National School Health Examination Survey (KNSHES) | National | Both | 6-20 | 6-20 | 56,933 | 46,295 |
| 1604 | South Korea | 2011 | Korea National Health and Nutrition Examination Survey | National | Both | 5+ | 5+ | 3,363 | 4,193 |
| 1605 | South Korea | 2010-2011 | Korean National Health Insurance | National | Both | 40+ | 40+ | 6,671,572 | 7,127,111 |
| 1606 | South Korea | 2012 | Korea National Health and Nutrition Examination Survey | National | Both | 5+ | 5+ | 3,194 | 4,030 |
| 1607 | South Korea | 2013 | Korea National Health and Nutrition Examination Survey | National | Both | 5+ | 5+ | 3,211 | 3,934 |
| 1608 | South Korea | 2012-2013 | Korean National Health Insurance | National | Both | 40+ | 40+ | 7,256,898 | 7,782,621 |
| 1609 | South Korea | 2014 | Korea National Health and Nutrition Examination Survey | National | Both | 5+ | 5+ | 2,966 | 3,769 |
| 1610 | South Korea | 2015 | Korea National Health and Nutrition Examination Survey | National | Both | 5+ | 5+ | 3,022 | 3,640 |
| 1611 | South Korea | 2014-2015 | Korean National Health Insurance | National | Both | 40+ | 40+ | 7,869,485 | 8,354,998 |
| 1612 | South Korea | 2016 | Korea National Health and Nutrition Examination Survey | National | Both | 5+ | 5+ | 3,288 | 4,042 |
| 1613 | South Korea | 2017 | Korea National Health and Nutrition Examination Survey | National | Both | 5+ | 5+ | 3,341 | 3,986 |
| 1614 | South Korea | 2016-2017 | Korean National Health Insurance | National | Both | 40+ | 40+ | 8,534,031 | 9,071,978 |
| 1615 | Spain | 1985 | INTERSALT, Manresa | Community | Urban | 20-59 | 20-59 | 100 | 100 |
| 1616 | Spain | 1986 | INTERSALT, Torrejo | Community | Urban | 20-59 | 20-59 | 100 | 100 |
| 1617 | Spain | 1986-1988 | MONICA, Catalonia | Subnational | Both | 25-64 | 25-64 | 1,251 | 1,271 |
| 1618 | Spain | 1989 | Cardiovascular Risk Factors Study in Catalonia | Subnational | Both | 15+ | 15+ | 330 | 375 |
| 1619 | Spain | 1990 | Banegas et al., Hypertension 32(6):998-1002, 1998 | National | Both | 35-65 | 35-65 | 810 | 1,203 |
| 1620 | Spain | 1990-1992 | MONICA, Catalonia | Subnational | Both | 25-64 | 25-64 | 1,719 | 1,191 |
| 1621 | Spain | 1991-1993 | Encuesta de Factores de Riesgo Cardiovascular en la Región de Murcia (Cardiovascular Risk Factors Survey) | Subnational | Both | 18-69 | 18-69 | 1,512 | 1,562 |
| 1622 | Spain | 1992 | CINDI | Subnational | Both | 25-64 | 25-64 | 1,194 | 1,454 |
| 1623 | Spain | 1989-1994 | SEEDO | Subnational | Both | 25-60 | 25-60 | 2,533 | 2,855 |
| 1624 | Spain | 1992 | ENCAT | Community | Both | 15-80 | 15-80 | 786 | 952 |
| 1625 | Spain | 1994-1995 | Encuesta de Nutrición y Salud Comunidad Valenciana 1994-95 (ENCV) | Subnational | Urban | 15+ | 15+ | 830 | 959 |
| 1626 | Spain | 1994-1996 | MONICA, Catalonia | Subnational | Both | 25-64 | 25-64 | 1,800 | 1,628 |
| 1627 | Spain | 1990-2000 | SEEDO | Subnational | Both | 25-60 | 25-60 | 4,707 | 5,178 |
| 1628 | Spain | 1996 | Guía Study | Community | Urban | 30+ | 30+ | 305 | 384 |
| 1629 | Spain | 1997 | Soriguer et al., Eur J Epidemiol 19(1):33-40, 2004 | Community | Rural | 18-65 | 18-65 | 613 | 613 |

| 1630 | Spain | 1996-2002 | Castells et al., J Epidemiol Community Health 60(4):316-21, 2006 | Community | Urban |  | 50-69 |  | 26,963 |
| --- | --- | --- | --- | --- | --- | --- | --- | --- | --- |
| 1631 | Spain | 1998-2000 | EnKID study | National | Both | 5-24 | 5-24 | 1,452 | 1,730 |
| 1632 | Spain | 1999-2000 | ENIB | Community | Both | 20-60 | 20-60 | 498 | 702 |
| 1633 | Spain | 1999-2000 | Factores de riesgo en las islas Baleares: Estudio CORSAIB | Subnational | Both | 35-74 | 35-74 | 811 | 864 |
| 1634 | Spain | 2000-2001 | Regidor et al., J Hum Hypertens 20(1):73-82, 2006 | National | Both | 60+ | 60+ | 1,318 | 2,281 |
| 1635 | Spain | 2000-2001 | EUREYE Study | Subnational | Both | 65+ | 65+ | 274 | 324 |
| 1636 | Spain | 2001-2002 | Catalan Health Interview Survey | Subnational | Both | 18-74 | 18-74 | 597 | 745 |
| 1637 | Spain | 2001-2003 | DIabetes, Nutrición y Obesidad en la población adulta de la Región de Murcia (DINO) | Subnational | Both | 20+ | 20+ | 715 | 828 |
| 1638 | Spain | 2000-2005 | CDC of the Canary Islands | Subnational | Both | 18-75 | 18-75 | 2,878 | 3,719 |
| 1639 | Spain | 2003 | The European Male Ageing Study | Community | Both | 40+ |  | 405 |  |
| 1640 | Spain | 2002-2003 | ENCAT | Community | Both | 15-80 | 15-80 | 712 | 813 |
| 1641 | Spain | 2004 | Vioque J et al. Obesity 16:664-70, 2008 | Community | Urban | 24+ | 24+ | 87 | 115 |
| 1642 | Spain | 2004 | Cardiovascular Risk Study in Castilla y León (RECCyL) | Subnational | Both | 15+ | 15+ | 1,903 | 2,077 |
| 1643 | Spain | 2003-2005 | Registre Gironi del Cor (REGICOR) | Subnational | Both | 35-79 | 35-79 | 2,951 | 3,266 |
| 1644 | Spain | 2004-2006 | PREVICTUS | National | Both | 60+ | 60+ | 3,193 | 3,640 |
| 1645 | Spain | 2008 | The European Male Ageing Study | Community | Both | 40+ |  | 272 |  |
| 1646 | Spain | 2007-2009 | Harmonizing Equation of Risk in Mediterraneon countries Extremadura | Subnational | Both | 25-79 | 25-79 | 1,298 | 1,498 |
| 1647 | Spain | 2008-2010 | Study on Nutrition and Cardiovascular Risk in Spain | National | Both | 18+ | 18+ | 5,756 | 6,397 |
| 1648 | Spain | 2009 | Cardiovascular Risk Study in Castilla y León (RECCyL) | Subnational | Both | 20+ | 20+ | 1,315 | 1,590 |
| 1649 | Spain | 2013 | ANIBES Study | National | Both | 9-75 | 9-75 | 1,160 | 1,125 |
| 1650 | Spain | 2014 | Cardiovascular Risk Study in Castilla y León (RECCyL) | Subnational | Both | 20+ | 20+ | 1,215 | 1,475 |
| 1651 | Spain | 2015 | Study on Nutrition and Cardiovascular Risk in Spain (ENRICA) | National | Both | 65+ | 65+ | 711 | 770 |
| 1652 | Sri Lanka | 2003 | Wijewardene et al., Ceylon Med J 50:62-70, 2005 | Subnational | Both | 30-65 | 30-65 | 275 | 296 |
| 1653 | Sri Lanka | 2003 | Wijewardene et al., Ceylon Med J 50:62-70, 2005 | Subnational | Both | 30-65 | 30-65 | 139 | 192 |
| 1654 | Sri Lanka | 2003 | Wijewardene et al., Ceylon Med J 50:62-70, 2005 | Subnational | Both | 30-65 | 30-65 | 1,891 | 2,410 |
| 1655 | Sri Lanka | 2003 | Wijewardene et al., Ceylon Med J 50:62-70, 2005 | Subnational | Both | 30-65 | 30-65 | 387 | 457 |
| 1656 | Sri Lanka | 2006 | STEPS | National | Both | 15-64 | 15-64 | 6,140 | 6,213 |
| 1657 | Sri Lanka | 2006-2007 | DHS | National | Both |  | 15-49 |  | 12,539 |
| 1658 | Sri Lanka | 2014 | STEPS | National | Both | 18-69 | 18-69 | 1,863 | 2,893 |
| 1659 | Sudan | 2005 | STEPS | Subnational | Both | 25-64 | 25-64 | 626 | 881 |
| 1660 | Sudan | 2016 | STEPS | National | Both | 18-69 | 18-69 | 2,661 | 4,544 |
| 1661 | Suriname | 2013-2015 | The Healthy Life in Suriname Study (HELISUR) | Subnational | Urban | 18-70 | 18-70 | 424 | 722 |
| 1662 | Swaziland | 2006-2007 | DHS | National | Both | 15-49 | 15-49 | 4,074 | 4,714 |
| 1663 | Swaziland | 2007 | STEPS | National | Both | 25-64 | 25-64 | 433 | 827 |
| 1664 | Swaziland | 2014 | STEPS | National | Both | 15-69 | 15-69 | 1,102 | 1,976 |
| 1665 | Sweden | 1985 | MONICA Gothenburg | Community | Urban | 25-64 | 25-64 | 666 | 702 |
| 1666 | Sweden | 1986 | MONICA Northern Sweden | Subnational | Both | 25-64 | 25-64 | 822 | 798 |
| 1667 | Sweden | 1985-1989 | Västerbotten Intervention Project | Subnational | Both | 25-64 | 25-64 | 1,676 | 1,554 |
| 1668 | Sweden | 1990 | MONICA Gothenburg | Community | Urban | 25-64 | 25-64 | 775 | 775 |
| 1669 | Sweden | 1990 | MONICA Northern Sweden | Subnational | Both | 25-64 | 25-64 | 773 | 806 |
| 1670 | Sweden | 1990-1992 | Västerbotten Intervention Project | Subnational | Both | 25-64 | 25-64 | 7,263 | 7,804 |
| 1671 | Sweden | 1985-1996 | EPIC Umea | Subnational | Both | 24-72 | 24-72 | 12,359 | 13,217 |
| 1672 | Sweden | 1992-1993 | Population Study of Women in Gothenburg | Community | Urban |  | 62-84 |  | 802 |
| 1673 | Sweden | 1991-1995 | Uppsala Longitudinal Study of Adult Men | Community | Both | 70 |  | 1,215 |  |
| 1674 | Sweden | 1993-1995 | Västerbotten Intervention Project | Subnational | Both | 25-64 | 25-64 | 9,804 | 10,727 |
| 1675 | Sweden | 1994 | Helicobacter Pylori | Community | Urban | 56-65 | 56-65 | 170 | 217 |
| 1676 | Sweden | 1991-1996 | Malmö Diet and Cancer | Community | Urban | 45-73 | 45-73 | 12,096 | 18,293 |
| 1677 | Sweden | 1994 | MONICA Northern Sweden | Subnational | Both | 25-74 | 25-74 | 940 | 961 |
| 1678 | Sweden | 1995 | MONICA Gothenburg | Community | Urban | 25-64 | 25-64 | 745 | 867 |
| 1679 | Sweden | 1996-1998 | Västerbotten Intervention Project | Subnational | Both | 25-64 | 25-64 | 8,327 | 8,893 |
| 1680 | Sweden | 1999 | MONICA Northern Sweden | Subnational | Both | 25-74 | 25-74 | 889 | 920 |
| 1681 | Sweden | 1997-2001 | Uppsala Longitudinal Study of Adult Men | Community | Both | 77 |  | 783 |  |
| 1682 | Sweden | 1998-2001 | The Kalixanda study | Community | Both | 20+ | 20+ | 508 | 483 |
| 1683 | Sweden | 1999-2003 | Västerbotten Intervention Project | Subnational | Both | 25-64 | 25-64 | 6,354 | 6,384 |
| 1684 | Sweden | 2000-2001 | H70 Study | Community | Urban | 70 | 70 | 242 | 270 |
| 1685 | Sweden | 2003 | The European Male Ageing Study | Community | Both | 40+ |  | 396 |  |
| 1686 | Sweden | 2001-2004 | Swedish INTERGENE Cohort Study | Subnational | Both | 24-76 | 24-76 | 1,694 | 1,906 |
| 1687 | Sweden | 2001-2004 | Prospective Investigation of the Vasculature in Uppsala Seniors (PIVUS) | Community | Both | 70 | 70 | 507 | 509 |
| 1688 | Sweden | 2003-2004 | Welin et al., BMC Public Health 8:403, 2008 | Community | Urban | 50 | 50 | 595 | 655 |
| 1689 | Sweden | 2003-2004 | Welin et al., BMC Public Health 8:403, 2008 | Community | Urban | 60 |  | 667 |  |
| 1690 | Sweden | 2004 | MONICA Northern Sweden | Subnational | Both | 26-75 | 26-75 | 926 | 964 |
| 1691 | Sweden | 2004-2005 | European Youth Heart Study (EYHS) II | Subnational | Urban | 15-21 | 15-21 | 196 | 262 |
| 1692 | Sweden | 2004-2005 | Population Study of Women in Gothenburg | Community | Urban |  | 38-50 |  | 494 |
| 1693 | Sweden | 2005-2006 | H70 Study | Community | Urban | 75 | 75 | 320 | 422 |
| 1694 | Sweden | 2008 | The European Male Ageing Study | Community | Both | 40+ |  | 353 |  |
| 1695 | Sweden | 2006-2009 | Prospective Investigation of the Vasculature in Uppsala Seniors (PIVUS) | Community | Both | 75 | 75 | 407 | 419 |
| 1696 | Sweden | 2009 | MONICA Northern Sweden | Subnational | Both | 25-74 | 25-74 | 849 | 857 |
| 1697 | Sweden | 2011-2014 | EpiHealth | National | Both | 45-75 | 45-75 | 4,731 | 6,054 |

| 1698 | Sweden | 2014 | MONICA Northern Sweden | Subnational | Both | 25-74 | 25-74 | 753 | 795 |
| --- | --- | --- | --- | --- | --- | --- | --- | --- | --- |
| 1699 | Sweden | 2014-2016 | Swedish INTERGENE Cohort Study | Subnational | Urban | 37-88 | 37-88 | 602 | 653 |
| 1700 | Sweden | 2016-2017 | Population Study of Women in Gothenburg | Community | Urban |  | 38-50 |  | 570 |
| 1701 | Switzerland | 1984-1986 | The Swiss MONICA Study Wave I | Subnational | Both | 25-74 | 25-74 | 1,744 | 1,689 |
| 1702 | Switzerland | 1988-1989 | The Swiss MONICA Study Wave II | Subnational | Both | 25-74 | 25-74 | 1,778 | 1,684 |
| 1703 | Switzerland | 1992-1993 | The Swiss MONICA Study Wave III | Subnational | Both | 25-74 | 25-74 | 1,577 | 1,672 |
| 1704 | Switzerland | 2004 | The Swiss Conscription Database | National | Both | 18-20 |  | 20,491 |  |
| 1705 | Switzerland | 2003-2006 | Cohorte Lausannoise | Community | Urban | 35-75 | 35-75 | 3,186 | 3,536 |
| 1706 | Switzerland | 2005 | The Swiss Conscription Database | National | Both | 18-20 |  | 32,131 |  |
| 1707 | Switzerland | 2006 | The Swiss Conscription Database | National | Both | 18-20 |  | 34,530 |  |
| 1708 | Switzerland | 2007 | The Swiss Conscription Database | National | Both | 18-20 |  | 36,194 |  |
| 1709 | Switzerland | 2008 | The Swiss Conscription Database | National | Both | 18-20 |  | 34,497 |  |
| 1710 | Switzerland | 2009 | The Swiss Conscription Database | National | Both | 18-20 |  | 34,896 |  |
| 1711 | Switzerland | 2007-2012 | Bus Santé Study | Subnational | Urban | 20-80 | 20-80 | 1,884 | 1,911 |
| 1712 | Switzerland | 2010 | The Swiss Conscription Database | National | Both | 18-20 |  | 37,214 |  |
| 1713 | Switzerland | 2009-2012 | Cohorte Lausannoise | Community | Urban | 40-81 | 40-81 | 2,176 | 2,494 |
| 1714 | Switzerland | 2011 | The Swiss Conscription Database | National | Both | 18-20 |  | 38,108 |  |
| 1715 | Switzerland | 2012 | The Swiss Conscription Database | National | Both | 18-20 |  | 36,938 |  |
| 1716 | Switzerland | 2013 | The Swiss Conscription Database | National | Both | 18-20 |  | 32,890 |  |
| 1717 | Switzerland | 2014 | The Swiss Conscription Database | National | Both | 18-20 |  | 32,691 |  |
| 1718 | Switzerland | 2013-2016 | Bus Santé Study | Subnational | Urban | 20-74 | 20-74 | 2,022 | 2,186 |
| 1719 | Switzerland | 2015 | The Swiss Conscription Database | National | Both | 18-20 |  | 32,616 |  |
| 1720 | Switzerland | 2014-2017 | Cohorte Lausannoise | Community | Urban | 45-87 | 45-87 | 2,008 | 2,473 |
| 1721 | Syrian Arab Republic | 2002 | National survey on non-communicable diseases and factors affecting their development | National | Both | 15-64 | 15-64 | 3,155 | 4,045 |
| 1722 | Taiwan | 1985 | INTERSALT | Community | Rural | 20-59 | 20-59 | 89 | 92 |
| 1723 | Taiwan | 1989-1991 | Chiu et al., J Gerontol A Biol Sci Med Sci 55(11):M684-90, 2000 | Subnational | Both | 65+ | 65+ | 1,322 | 1,308 |
| 1724 | Taiwan | 1993-1994 | The Kinmen Neurological Disorders Survey | Community | Urban | 50+ | 50+ | 672 | 593 |
| 1725 | Taiwan | 1993-1996 | Nutrition and Health Survey in Taiwan 1993-1996 | National | Both | 5+ | 5+ | 2,959 | 3,216 |
| 1726 | Taiwan | 1999-2000 | Nutrition and Health Survey in Taiwan 1999-2000 | National | Both | 65+ | 65+ | 1,271 | 1,202 |
| 1727 | Taiwan | 2000 | Social Environment and Biomarkers of Aging Study | National | Both | 50+ | 50+ | 590 | 433 |
| 1728 | Taiwan | 2004-2005 | TCHS | Community | Urban | 40+ | 40+ | 1,147 | 1,212 |
| 1729 | Taiwan | 2006 | Social Environment and Biomarkers of Aging Study | National | Both | 53+ | 53+ | 548 | 476 |
| 1730 | Taiwan | 2005-2008 | Nutrition and Health Survey in Taiwan 2005-2008 | National | Both | 19+ | 19+ | 1,311 | 1,355 |
| 1731 | Taiwan | 2007 | Taiwanese Survey on Hypertension, Hyperglycemia and Hyperlipidemia | National | Both | 20+ | 20+ | 2,155 | 2,469 |
| 1732 | Taiwan | 2013-2016 | Nutrition and Health Survey in Taiwan | National | Both | 5+ | 5+ | 2,556 | 2,672 |
| 1733 | Tajikistan | 2003 | Micronutrient Status Survey | National | Both |  | 15-49 |  | 2,044 |
| 1734 | Tajikistan | 2012 | DHS | National | Both |  | 15-49 |  | 8,930 |
| 1735 | Tajikistan | 2016 | STEPS | National | Both | 18-69 | 18-69 | 1,091 | 1,553 |
| 1736 | Tajikistan | 2017 | DHS | National | Both |  | 15-49 |  | 9,922 |
| 1737 | Tanzania | 1991-1992 | DHS | National | Both |  | 20-49 |  | 4,039 |
| 1738 | Tanzania | 1996 | DHS | National | Both |  | 20-49 |  | 3,512 |
| 1739 | Tanzania | 1996-1997 | Aspray et al., Trans R Soc Trop Med Hyg 94:637-44, 2000 | Community | Rural | 15+ | 15+ | 251 | 324 |
| 1740 | Tanzania | 1996-1997 | Aspray et al., Trans R Soc Trop Med Hyg 94:637-44, 2000 | Community | Urban | 15+ | 15+ | 117 | 118 |
| 1741 | Tanzania | 1998-1999 | Bovet et al., Int J Epidemiol 31(1):240-7, 2002 | Community | Urban | 25-64 | 25-64 | 3,593 | 5,646 |
| 1742 | Tanzania | 2004-2005 | DHS | National | Both |  | 15-49 |  | 9,160 |
| 1743 | Tanzania | 2010 | DHS | National | Both |  | 15-49 |  | 9,099 |
| 1744 | Tanzania | 2011 | STEPS | Subnational | Both | 25-64 | 25-64 | 1,008 | 1,517 |
| 1745 | Tanzania | 2012 | STEPS | National | Both | 25-64 | 25-64 | 2,581 | 2,827 |
| 1746 | Tanzania | 2014 | Dar es Salaam Urban Cohort Hypertension Study | Community | Urban | 40+ | 40+ | 965 | 1,266 |
| 1747 | Tanzania | 2015-2016 | DHS | National | Both |  | 15-49 |  | 12,036 |
| 1748 | Thailand | 1987 | INCLEN | Community | Rural | 35-65 |  | 244 |  |
| 1749 | Thailand | 1989 | INCLEN | Community | Rural | 35-65 |  | 209 |  |
| 1750 | Thailand | 1989 | INCLEN | Community | Urban | 35-65 |  | 207 |  |
| 1751 | Thailand | 1991 | Thailand National Health Examination Survey I | National | Both | 5+ | 5+ | 8,698 | 11,027 |
| 1752 | Thailand | 1995 | The Fourth National Nutrition Survey of Thailand- 1995 | National | Both | 20-60 | 20-60 | 1,405 | 3,631 |
| 1753 | Thailand | 1997 | Thailand National Health Examination Survey II | National | Both | 5-59 | 5-59 | 4,117 | 4,876 |
| 1754 | Thailand | 2000 | InterASIA | National | Both | 35+ | 35+ | 2,092 | 3,211 |
| 1755 | Thailand | 2004 | Thailand National Health Examination Survey III | National | Both | 15+ | 15+ | 18,819 | 20,143 |
| 1756 | Thailand | 2003-2004 | The Fifth National Nutrition Survey of Thailand | National | Both | 19+ | 19+ | 1,961 | 3,366 |
| 1757 | Thailand | 2009 | Thailand National Health Examination Survey IV | National | Both | 5+ | 5+ | 12,972 | 13,848 |
| 1758 | Timor-Leste | 2009-2010 | DHS | National | Both |  | 15-49 |  | 11,983 |
| 1759 | Timor-Leste | 2009-2010 | Timor-Leste Eye Health Survey | Subnational | Both | 40+ | 40+ | 245 | 247 |
| 1760 | Timor-Leste | 2014 | STEPS | National | Both | 18-69 | 18-69 | 1,048 | 1,437 |
| 1761 | Timor-Leste | 2016 | DHS | National | Both | 15-59 | 15-49 | 4,556 | 11,823 |
| 1762 | Togo | 1998 | DHS | National | Both |  | 20-49 |  | 3,114 |
| 1763 | Togo | 2010 | STEPS | National | Both | 15-64 | 15-64 | 2,063 | 2,095 |
| 1764 | Togo | 2013-2014 | DHS | National | Both |  | 15-49 |  | 4,398 |
| 1765 | Togo | 2014 | Impact evaluation of a cash transfer program in North Togo | Subnational | Rural |  | 20-65 |  | 3,588 |

| 1766 | Tokelau | 2005 | STEPS | National | Both | 15-64 | 15-64 | 270 | 296 |
| --- | --- | --- | --- | --- | --- | --- | --- | --- | --- |
| 1767 | Tokelau | 2014 | STEPS | National | Both | 18-64 | 18-64 | 261 | 276 |
| 1768 | Tonga | 2004 | STEPS | National | Both | 15-64 | 15-64 | 403 | 552 |
| 1769 | Tonga | 2007-2008 | Pacific Obesity Prevention in Communities - Ma’alahi Youth Project | Subnational | Rural | 13-22 | 13-22 | 434 | 579 |
| 1770 | Tonga | 2011 | STEPS | National | Both | 15-64 | 15-64 | 878 | 1,401 |
| 1771 | Trinidad and Tobago | 1985 | INTERSALT | Community | Urban | 20-59 | 20-59 | 84 | 92 |
| 1772 | Trinidad and Tobago | 2001 | Adult Survey | National | Rural | 25+ | 25+ | 198 | 267 |
| 1773 | Tunisia | 1996-1997 | Tunisian National Nutrition Survey 1996-1997 | National | Both | 5+ | 5+ | 2,724 | 4,125 |
| 1774 | Tunisia | 1996-1997 | Ariana Healthy Project 1997 | Community | Both | 35-65 | 35-65 | 2,664 | 2,711 |
| 1775 | Tunisia | 2005 | Tunisian National Survey | National | Both | 35-71 | 35-71 | 3,417 | 4,590 |
| 1776 | Tunisia | 2009-2010 | ObeMaghreb | Subnational | Urban | 5-49 | 5-49 | 1,841 | 1,601 |
| 1777 | Turkey | 1990 | Turkish Adult Risk Factor Study | National | Both | 20+ | 20+ | 1,338 | 1,369 |
| 1778 | Turkey | 1993 | DHS | National | Both |  | 20-49 |  | 2,294 |
| 1779 | Turkey | 1995 | Turkish Adult Risk Factor Study | National | Both | 25+ | 25+ | 855 | 878 |
| 1780 | Turkey | 1998 | DHS | National | Both |  | 20-49 |  | 2,210 |
| 1781 | Turkey | 1998 | Turkish Adult Risk Factor Study | National | Both | 28+ | 28+ | 877 | 909 |
| 1782 | Turkey | 1998-1999 | Erem et al., Diabetes Res Clin Pract 54(3):203-08, 2001 | Community | Urban | 20+ | 20+ | 1,324 | 1,322 |
| 1783 | Turkey | 2000 | Turkish Adult Risk Factor Study | National | Both | 30+ | 30+ | 890 | 938 |
| 1784 | Turkey | 2000 | MDHS | Subnational | Urban |  | 15-49 |  | 1,420 |
| 1785 | Turkey | 2001 | Yumuk et al., Diabetes Res Clin Pract 70(2):151-58, 2005 | Community | Urban | 20+ | 20+ | 1,042 | 1,789 |
| 1786 | Turkey | 2000-2002 | The Healthy Nutrition for Healthy Heart Study | National | Both | 25-84 | 25-84 | 4,718 | 10,631 |
| 1787 | Turkey | 2001-2002 | Turkish Adult Risk Factor Study | National | Both | 32+ | 32+ | 1,098 | 1,209 |
| 1788 | Turkey | 2002 | Onal et al., Blood Press 13(1):31-6, 2004 | Subnational | Urban | 25+ | 25+ | 67 | 355 |
| 1789 | Turkey | 2003 | DHS | National | Both |  | 20-49 |  | 2,934 |
| 1790 | Turkey | 2003 | Prevalence, awareness, treatment and control of hypertension in Turkey in 2003 | National | Both | 18+ | 18+ | 1,988 | 2,847 |
| 1791 | Turkey | 2003-2004 | Turkish Adult Risk Factor Study | National | Both | 34+ | 34+ | 1,097 | 1,130 |
| 1792 | Turkey | 2003-2005 | Prevalence of prehypertension and associated risk factors among Turkish adults: Trabzon Hypertension Study | Subnational | Both | 20+ | 20+ | 2,208 | 2,601 |
| 1793 | Turkey | 2004 | Nationally Representative Cross-sectional Survey | National | Both | 20+ | 20+ | 2,110 | 2,154 |
| 1794 | Turkey | 2005-2006 | Turkish Adult Risk Factor Study | National | Both | 35+ | 35+ | 965 | 1,029 |
| 1795 | Turkey | 2007 | Natinal Household survey | National | Both | 20-85 | 20-85 | 2,263 | 1,842 |
| 1796 | Turkey | 2008 | DHS | National | Both |  | 15-49 |  | 6,167 |
| 1797 | Turkey | 2007-2008 | Turkish Adult Risk Factor Study | National | Both | 37+ | 37+ | 1,048 | 1,070 |
| 1798 | Turkey | 2009-2010 | Turkish Adult Risk Factor Study | National | Both | 39+ | 39+ | 462 | 501 |
| 1799 | Turkey | 2009-2012 | Prevalence of diabetes and associated risk factors among adult population in Trabzon city | Subnational | Both | 20+ | 20+ | 1,570 | 2,124 |
| 1800 | Turkey | 2011 | Chronic Diseases and Risk Factors Survey in Turkey | National | Both | 15+ | 15+ | 8,061 | 8,924 |
| 1801 | Turkey | 2013 | DHS | National | Both |  | 15-49 |  | 8,270 |
| 1802 | Turkey | 2012-2013 | Turkish Adult Risk Factor Study | National | Both | 37+ | 37+ | 1,012 | 1,087 |
| 1803 | Turkey | 2014-2015 | Turkish Adult Risk Factor Study | National | Both | 44+ | 44+ | 437 | 484 |
| 1804 | Turkey | 2017 | STEPS | National | Both | 15+ | 15+ | 2,306 | 3,426 |
| 1805 | Turkmenistan | 2000 | DHS | National | Both |  | 15-49 |  | 2,084 |
| 1806 | Turkmenistan | 2013 | STEPS | National | Both | 18-64 | 18-64 | 1,879 | 2,741 |
| 1807 | Turkmenistan | 2018 | STEPS | National | Both | 18-69 | 18-69 | 1,713 | 2,236 |
| 1808 | Tuvalu | 2015 | STEPS | National | Both | 18-69 | 18-69 | 478 | 550 |
| 1809 | Uganda | 1995 | DHS | National | Both |  | 20-49 |  | 2,831 |
| 1810 | Uganda | 2000-2001 | DHS | National | Both |  | 15-49 |  | 5,829 |
| 1811 | Uganda | 2006 | DHS | National | Both | 15-54 | 15-49 | 2,475 | 2,538 |
| 1812 | Uganda | 2011 | DHS | National | Both | 15-54 | 15-49 | 2,361 | 2,501 |
| 1813 | Uganda | 2011-2013 | Gulu Health and Demographic Surveillance Site (HDSS) | Community | Rural | 5+ | 5+ | 3,938 | 4,820 |
| 1814 | Uganda | 2011-2012 | The Prevalence and Distribution of Non-communicable Diseases and Their Risk Factors in Kasese District, Uganda | Subnational | Both | 25-79 | 25-79 | 277 | 221 |
| 1815 | Uganda | 2014 | STEPS | National | Both | 18-69 | 18-69 | 1,560 | 2,120 |
| 1816 | Uganda | 2014-2015 | Gulu Health and Demographic Surveillance Site (HDSS) | Community | Rural | 15-24 | 15-24 | 671 | 517 |
| 1817 | Uganda | 2016 | DHS | National | Both | 15-54 | 15-54 | 5,191 | 5,415 |
| 1818 | Ukraine | 2002 | National Micronutrient Survey | National | Both |  | 15-50 |  | 816 |
| 1819 | Ukraine | 2019 | STEPS | National | Both | 18-69 | 18-69 | 1,569 | 2,600 |
| 1820 | United Arab Emirates | 1989-1990 | el Mugamer et al., J Trop Med Hyg 98(6):407-15, 1995 | Community | Both | 20+ | 20+ | 123 | 199 |
| 1821 | United Arab Emirates | 1999-2000 | Emirates National Diabetes and Coronary Artery Disease Risk Factor Study | National | Both | 20-80 | 20-80 | 2,822 | 3,743 |
| 1822 | United Arab Emirates | 2000-2001 | Carter et al., J Health Popul Nutr 22(1):75-83, 2004 | Community | Both |  | 20-79 |  | 521 |
| 1823 | United Arab Emirates | 2009 | Gulf Cooperation Council World Health Survey | National | Both | 18+ | 18+ | 605 | 645 |
| 1824 | United Arab Emirates | 2017-2018 | STEPS | National | Both | 18+ | 18+ | 2,148 | 2,324 |
| 1825 | United Kingdom | 1985-1986 | INTERSALT, Belfast | Community | Urban | 20-59 | 20-59 | 99 | 100 |
| 1826 | United Kingdom | 1985 | INTERSALT, Birmingham | Community | Urban | 20-59 | 20-59 | 100 | 100 |
| 1827 | United Kingdom | 1985 | INTERSALT, Wales | Community | Urban | 20-59 | 20-59 | 100 | 99 |
| 1828 | United Kingdom | 1984-1986 | Scottish Heart Health Survey | Subnational | Both | 40-59 | 40-59 | 4,364 | 4,465 |
| 1829 | United Kingdom | 1986-1987 | Dietary and Nutritional Survey of British Adults 1986-1987 | National | Both | 16-64 | 16-64 | 1,158 | 1,161 |
| 1830 | United Kingdom | 1986-1987 | MONICA, Belfast | Subnational | Both | 25-64 | 25-64 | 1,155 | 1,185 |
| 1831 | United Kingdom | 1987-1988 | Edinburgh Artery Study | Community | Urban | 54-75 | 54-75 | 808 | 783 |
| 1832 | United Kingdom | 1989 | MRC National Survey of Health and Development | National | Both | 42-44 | 42-44 | 1,617 | 1,608 |
| 1833 | United Kingdom | 1991 | National Child Development Study (1958 British Cohort Study) | National | Both | 33 | 33 | 5,426 | 5,605 |

| 1834 | United Kingdom | 1991-1992 | Health Survey for England | National | Both | 16+ | 16+ | 3,114 | 3,430 |
| --- | --- | --- | --- | --- | --- | --- | --- | --- | --- |
| 1835 | United Kingdom | 1991-1992 | MONICA, Belfast | Subnational | Both | 25-64 | 25-64 | 998 | 996 |
| 1836 | United Kingdom | 1992 | MONICA, Glasgow | Community | Urban | 25-64 | 25-64 | 696 | 775 |
| 1837 | United Kingdom | 1993 | Health Survey for England | National | Both | 16+ | 16+ | 7,461 | 8,297 |
| 1838 | United Kingdom | 1992-1993 | Whickham Survey | Community | Urban | 35+ | 35+ | 676 | 784 |
| 1839 | United Kingdom | 1992-1994 | Edinburgh Artery Study | Community | Urban | 60-81 | 60-81 | 580 | 582 |
| 1840 | United Kingdom | 1994 | Health Survey for England | National | Both | 16+ | 16+ | 6,825 | 7,939 |
| 1841 | United Kingdom | 1993-1997 | EPIC Norfolk | Subnational | Both | 40-79 | 40-79 | 11,574 | 13,995 |
| 1842 | United Kingdom | 1994-1995 | Hertfordshire Ageing Study | Subnational | Both | 63-73 | 63-73 | 411 | 304 |
| 1843 | United Kingdom | 1995 | Health Survey for England | National | Both | 5+ | 5+ | 8,038 | 9,027 |
| 1844 | United Kingdom | 1994-1995 | National Diet and Nutrition Survey (NDNS) | National | Both | 65+ | 65+ | 701 | 687 |
| 1845 | United Kingdom | 1995 | Scottish Health Survey (SHeS) | Subnational | Both | 16-64 | 16-64 | 3,303 | 4,005 |
| 1846 | United Kingdom | 1995 | MONICA, Glasgow | Community | Urban | 25-64 | 25-64 | 855 | 958 |
| 1847 | United Kingdom | 1996 | British Cohort Study 1970 | National | Both | 26 | 26 | 81 | 78 |
| 1848 | United Kingdom | 1996 | Health Survey for England | National | Both | 5+ | 5+ | 8,469 | 9,461 |
| 1849 | United Kingdom | 1993-2000 | EPIC Oxford | Subnational | Both | 20-98 | 20-98 | 10,851 | 37,605 |
| 1850 | United Kingdom | 1997 | Health Survey for England | National | Both | 5+ | 5+ | 6,285 | 6,841 |
| 1851 | United Kingdom | 1998 | Health Survey for England | National | Both | 5+ | 5+ | 7,980 | 9,047 |
| 1852 | United Kingdom | 1998-1999 | INTERMAP, Belfast | Community | Urban | 40-59 | 40-59 | 125 | 97 |
| 1853 | United Kingdom | 1997-1999 | INTERMAP, WestBromwich | Community | Urban | 40-59 | 40-59 | 141 | 138 |
| 1854 | United Kingdom | 1998 | Scottish Health Survey (SHeS) | Subnational | Both | 5-74 | 5-74 | 5,047 | 5,908 |
| 1855 | United Kingdom | 1998-2000 | The British Regional Heart Study | National | Urban | 60-79 |  | 4,138 |  |
| 1856 | United Kingdom | 1999 | Health Survey for England | National | Both | 5+ | 5+ | 3,880 | 4,304 |
| 1857 | United Kingdom | 1999 | MRC National Survey of Health and Development | National | Both | 53-54 | 53-54 | 1,452 | 1,496 |
| 1858 | United Kingdom | 1999-2001 | British Women's Heart and Health Study | National | Both |  | 60-79 |  | 3,677 |
| 1859 | United Kingdom | 2000 | Health Survey for England | National | Both | 5+ | 5+ | 4,073 | 4,607 |
| 1860 | United Kingdom | 1999-2001 | Edinburgh Artery Study | Community | Urban | 66-87 | 66-87 | 373 | 404 |
| 1861 | United Kingdom | 1999-2004 | Hertfordshire Cohort Study | Subnational | Both | 59-73 | 59-73 | 1,571 | 1,416 |
| 1862 | United Kingdom | 2001 | Health Survey for England | National | Both | 5+ | 5+ | 7,463 | 8,657 |
| 1863 | United Kingdom | 2000-2001 | National Diet and Nutrition Survey 2000-2001 | National | Both | 19-64 | 19-64 | 807 | 973 |
| 1864 | United Kingdom | 2002 | Health Survey for England | National | Both | 5+ | 5+ | 6,797 | 7,578 |
| 1865 | United Kingdom | 2003 | The European Male Ageing Study | Community | Both | 40+ |  | 394 |  |
| 1866 | United Kingdom | 2003 | Health Survey for England | National | Both | 5+ | 5+ | 7,136 | 8,268 |
| 1867 | United Kingdom | 2003 | Scottish Health Survey (SHeS) | Subnational | Both | 5+ | 5+ | 3,988 | 4,687 |
| 1868 | United Kingdom | 2003-2005 | Hertfordshire Ageing Study | Subnational | Both | 72-82 | 72-82 | 171 | 119 |
| 1869 | United Kingdom | 2004 | Health Survey for England | National | Both | 5+ | 5+ | 2,975 | 3,608 |
| 1870 | United Kingdom | 2004-2005 | English Longitudinal Study of Ageing Wave 2 2004-2005 | National | Both | 52+ | 52+ | 3,259 | 3,966 |
| 1871 | United Kingdom | 2005 | Health Survey for England | National | Both | 5+ | 5+ | 4,839 | 5,503 |
| 1872 | United Kingdom | 2006 | Health Survey for England | National | Both | 5+ | 5+ | 8,005 | 8,912 |
| 1873 | United Kingdom | 2007 | Health Survey for England | National | Both | 5+ | 5+ | 5,354 | 5,708 |
| 1874 | United Kingdom | 2008 | The European Male Ageing Study | Community | Both | 40+ |  | 301 |  |
| 1875 | United Kingdom | 2008 | Health Survey for England | National | Both | 5+ | 5+ | 8,317 | 9,472 |
| 1876 | United Kingdom | 2008 | Scottish Health Survey (SHeS) | Subnational | Both | 5+ | 5+ | 2,970 | 3,533 |
| 1877 | United Kingdom | 2008-2009 | English Longitudinal Study of Ageing Wave 4 2008-2009 | National | Both | 50+ | 50+ | 3,540 | 4,296 |
| 1878 | United Kingdom | 2009 | Health Survey for England | National | Both | 5+ | 5+ | 3,242 | 3,384 |
| 1879 | United Kingdom | 2006-2010 | MRC National Survey of Health and Development | National | Both | 60-65 | 60-65 | 1,061 | 1,156 |
| 1880 | United Kingdom | 2009 | Scottish Health Survey (SHeS) | Subnational | Both | 5+ | 5+ | 3,621 | 4,168 |
| 1881 | United Kingdom | 2010 | Health Survey for England | National | Both | 5+ | 5+ | 4,959 | 5,547 |
| 1882 | United Kingdom | 2008-2012 | National Diet and Nutrition Survey (NDNS) | National | Both | 5+ | 5+ | 2,580 | 3,094 |
| 1883 | United Kingdom | 2010 | Scottish Health Survey (SHeS) | Subnational | Both | 5+ | 5+ | 3,206 | 3,767 |
| 1884 | United Kingdom | 2011 | Health Survey for England | National | Both | 5+ | 5+ | 3,680 | 4,404 |
| 1885 | United Kingdom | 2011 | Scottish Health Survey (SHeS) | Subnational | Both | 5+ | 5+ | 3,253 | 3,862 |
| 1886 | United Kingdom | 2012 | Health Survey for England | National | Both | 5+ | 5+ | 3,648 | 4,278 |
| 1887 | United Kingdom | 2012 | Scottish Health Survey (SHeS) | Subnational | Both | 5+ | 5+ | 2,378 | 2,734 |
| 1888 | United Kingdom | 2012-2013 | English Longitudinal Study of Ageing Wave 6 2012-2013 | National | Both | 50+ | 50+ | 3,257 | 4,015 |
| 1889 | United Kingdom | 2013 | Health Survey for England | National | Both | 5+ | 5+ | 3,910 | 4,577 |
| 1890 | United Kingdom | 2013 | Scottish Health Survey (SHeS) | Subnational | Both | 5+ | 5+ | 2,340 | 2,747 |
| 1891 | United Kingdom | 2014 | Health Survey for England | National | Both | 5+ | 5+ | 3,712 | 4,332 |
| 1892 | United Kingdom | 2013-2014 | National Diet and Nutrition Survey (NDNS) | National | Both | 5+ | 5+ | 940 | 1,194 |
| 1893 | United Kingdom | 2014 | Scottish Health Survey (SHeS) | Subnational | Both | 5+ | 5+ | 2,248 | 2,675 |
| 1894 | United Kingdom | 2015 | Health Survey for England | National | Both | 5+ | 5+ | 4,837 | 5,491 |
| 1895 | United Kingdom | 2015 | MRC National Survey of Health and Development | National | Both | 69-70 | 69-70 | 1,040 | 1,082 |
| 1896 | United Kingdom | 2015 | Scottish Health Survey (SHeS) | Subnational | Both | 5+ | 5+ | 2,264 | 2,530 |
| 1897 | United Kingdom | 2016 | Health Survey for England | National | Both | 5+ | 5+ | 3,432 | 4,098 |
| 1898 | United Kingdom | 2015-2016 | National Diet and Nutrition Survey (NDNS) | National | Both | 5+ | 5+ | 1,021 | 1,188 |
| 1899 | United Kingdom | 2016 | Scottish Health Survey (SHeS) | Subnational | Both | 5+ | 5+ | 2,041 | 2,395 |
| 1900 | United Kingdom | 2017 | Health Survey for England | National | Both | 5+ | 5+ | 3,393 | 4,151 |
| 1901 | United Kingdom | 2016-2017 | National Diet and Nutrition Survey (NDNS) | National | Both | 5+ | 5+ | 468 | 545 |

| 1902 | United Kingdom | 2017 | Scottish Health Survey | Subnational | Both | 5+ | 5+ | 1,734 | 2,056 |
| --- | --- | --- | --- | --- | --- | --- | --- | --- | --- |
| 1903 | United States of America | 1985-1986 | Coronary Artery Risk Development in Young Adults (CARDIA) | Subnational | Urban | 18-30 | 18-30 | 2,321 | 2,775 |
| 1904 | United States of America | 1985-1986 | INTERSALT, Chicago | Community | Urban | 20-59 | 20-59 | 97 | 99 |
| 1905 | United States of America | 1986 | INTERSALT, Goodman | Community | Urban | 20-59 | 20-59 | 192 | 192 |
| 1906 | United States of America | 1985-1986 | MONICA, Stanford | Subnational | Urban | 25-64 | 25-64 | 713 | 848 |
| 1907 | United States of America | 1985-1987 | The Minnesota Heart Survey | Community | Both | 25-75 | 25-75 | 5,220 | 2,421 |
| 1908 | United States of America | 1987-1989 | Atherosclerosis Risk in Communities Study | Subnational | Both | 44-66 | 44-66 | 5,041 | 6,213 |
| 1909 | United States of America | 1987-1988 | The Bogalusa Heart Study | Community | Rural | 5-22 | 5-22 | 1,685 | 1,616 |
| 1910 | United States of America | 1987-1988 | Coronary Artery Risk Development in Young Adults (CARDIA) | Subnational | Urban | 20-32 | 20-32 | 2,082 | 2,506 |
| 1911 | United States of America | 1989-1990 | Cardiovascular Health Study | Subnational | Both | 65+ | 65+ | 2,458 | 3,318 |
| 1912 | United States of America | 1989-1990 | MONICA, Stanford | Subnational | Urban | 25-64 | 25-64 | 720 | 842 |
| 1913 | United States of America | 1990-1992 | Atherosclerosis Risk in Communities Study | Subnational | Both | 46-70 | 46-70 | 4,537 | 5,624 |
| 1914 | United States of America | 1990-1991 | Coronary Artery Risk Development in Young Adults (CARDIA) | Subnational | Urban | 23-35 | 23-35 | 1,945 | 2,382 |
| 1915 | United States of America | 1990-1991 | Cardiovascular Health Study | Subnational | Both | 65+ | 65+ | 2,070 | 2,707 |
| 1916 | United States of America | 1988-1994 | US NHANES III | National | Both | 5+ | 5+ | 11,567 | 12,524 |
| 1917 | United States of America | 1991-1992 | Cardiovascular Health Study | Subnational | Both | 65+ | 65+ | 1,919 | 2,563 |
| 1918 | United States of America | 1992-1994 | The Bogalusa Heart Study | Community | Rural | 5-21 | 5-21 | 1,578 | 1,627 |
| 1919 | United States of America | 1992-1993 | Coronary Artery Risk Development in Young Adults (CARDIA) | Subnational | Urban | 25-37 | 25-37 | 1,823 | 2,163 |
| 1920 | United States of America | 1992-1993 | Cardiovascular Health Study | Subnational | Both | 65+ | 65+ | 1,985 | 2,764 |
| 1921 | United States of America | 1993-1995 | Atherosclerosis Risk in Communities Study | Subnational | Both | 48-73 | 48-73 | 4,000 | 5,015 |
| 1922 | United States of America | 1993-1994 | Cardiovascular Health Study | Subnational | Both | 65+ | 65+ | 1,751 | 2,471 |
| 1923 | United States of America | 1994-1995 | Cardiovascular Health Study | Subnational | Both | 66+ | 66+ | 1,617 | 2,354 |
| 1924 | United States of America | 1996 | National Longitudinal Study of Adolescent Health Wave II | National | Both | 11-21 | 11-21 | 2,287 | 2,459 |
| 1925 | United States of America | 1995-1996 | Coronary Artery Risk Development in Young Adults (CARDIA) | Subnational | Urban | 28-40 | 28-40 | 1,739 | 2,145 |
| 1926 | United States of America | 1995-1996 | Cardiovascular Health Study | Subnational | Both | 66+ | 66+ | 1,478 | 2,194 |
| 1927 | United States of America | 1993-1998 | Women's Health Initiative - Observational Study | National | Both |  | 49-81 |  | 92,697 |
| 1928 | United States of America | 1996-1998 | Atherosclerosis Risk in Communities Study | Subnational | Both | 50-75 | 50-75 | 3,550 | 4,485 |
| 1929 | United States of America | 1996-1997 | Cardiovascular Health Study | Subnational | Both | 67+ | 67+ | 1,356 | 2,043 |
| 1930 | United States of America | 1996-1997 | INTERMAP, Baltimore | Community | Urban | 40-59 | 40-59 | 146 | 134 |
| 1931 | United States of America | 1997-1998 | INTERMAP, CC | Community | Urban | 40-59 | 40-59 | 271 | 276 |
| 1932 | United States of America | 1997-1998 | INTERMAP, Chicago | Community | Urban | 40-59 | 40-59 | 156 | 159 |
| 1933 | United States of America | 1996-1997 | INTERMAP, Jackson | Community | Urban | 40-59 | 40-59 | 132 | 134 |
| 1934 | United States of America | 1996-1998 | INTERMAP, Minneapolis | Community | Urban | 40-59 | 40-59 | 130 | 130 |
| 1935 | United States of America | 1996-1997 | INTERMAP, Pittsburgh | Community | Urban | 40-59 | 40-59 | 132 | 128 |
| 1936 | United States of America | 1996-1997 | Study of Women's Health Across the Nation | Subnational | Both |  | 40-55 |  | 3,200 |
| 1937 | United States of America | 1997-1998 | Cardiovascular Health Study | Subnational | Both | 68+ | 68+ | 1,172 | 1,801 |
| 1938 | United States of America | 1997-1999 | Study of Women's Health Across the Nation | Subnational | Both |  | 40-55 |  | 2,761 |
| 1939 | United States of America | 1998-1999 | Cardiovascular Health Study | Subnational | Both | 69+ | 69+ | 1,092 | 1,684 |
| 1940 | United States of America | 1998-2000 | Study of Women's Health Across the Nation | Subnational | Both |  | 40-55 |  | 2,596 |
| 1941 | United States of America | 1999-2000 | US NHANES 1999-2000 | National | Both | 5+ | 5+ | 3,809 | 3,791 |
| 1942 | United States of America | 1999-2001 | Study of Women's Health Across the Nation | Subnational | Both |  | 40-56 |  | 2,507 |
| 1943 | United States of America | 2000-2001 | Coronary Artery Risk Development in Young Adults (CARDIA) | Subnational | Urban | 33-45 | 33-45 | 1,570 | 1,949 |
| 1944 | United States of America | 2000-2002 | Study of Women's Health Across the Nation | Subnational | Both |  | 40-57 |  | 2,441 |
| 1945 | United States of America | 2001-2002 | National Longitudinal Study of Adolescent Health Wave III | National | Both | 18-28 | 18-28 | 2,139 | 2,443 |
| 1946 | United States of America | 2001-2002 | US NHANES 2001-2002 | National | Both | 5+ | 5+ | 4,045 | 4,006 |
| 1947 | United States of America | 2004 | Health and Retirement Study | National | Both | 24+ | 24+ | 241 | 262 |
| 1948 | United States of America | 2003-2004 | US NHANES 2003-2004 | National | Both | 5+ | 5+ | 3,938 | 3,838 |
| 1949 | United States of America | 2005-2006 | Coronary Artery Risk Development in Young Adults (CARDIA) | Subnational | Urban | 38-50 | 38-50 | 1,528 | 2,000 |
| 1950 | United States of America | 2005-2006 | Cardiovascular Health Study | Subnational | Both | 70+ | 70+ | 375 | 684 |
| 1951 | United States of America | 2006 | Health and Retirement Study | National | Both | 53+ | 53+ | 2,834 | 3,822 |
| 1952 | United States of America | 2005-2006 | US NHANES 2005-2006 | National | Both | 5+ | 5+ | 3,984 | 3,835 |
| 1953 | United States of America | 2005-2006 | National Social Life Health and Aging Project | National | Both | 57-85 | 57-85 | 1,355 | 1,435 |
| 1954 | United States of America | 2008 | Health and Retirement Study | National | Both | 55+ | 55+ | 2,494 | 3,493 |
| 1955 | United States of America | 2007-2008 | US NHANES 2007-2008 | National | Both | 5+ | 5+ | 4,086 | 4,038 |
| 1956 | United States of America | 2008-2009 | National Longitudinal Study of Adolescent Health Wave IV | National | Both | 24-34 | 24-34 | 2,317 | 2,725 |
| 1957 | United States of America | 2009-2010 | US NHANES 2009-2010 | National | Both | 5+ | 5+ | 4,291 | 4,332 |
| 1958 | United States of America | 2010-2011 | Coronary Artery Risk Development in Young Adults (CARDIA) | Subnational | Urban | 43-55 | 43-55 | 1,513 | 1,976 |
| 1959 | United States of America | 2010-2011 | Health and Retirement Study | National | Both | 57+ | 57+ | 2,535 | 3,465 |
| 1960 | United States of America | 2010-2011 | National Social Life Health and Aging Project | National | Both | 36-99 | 36-99 | 1,452 | 1,736 |
| 1961 | United States of America | 2011-2013 | Atherosclerosis Risk in Communities Study | Subnational | Both | 67-90 | 67-90 | 1,787 | 2,431 |
| 1962 | United States of America | 2012 | Health and Retirement Study | National | Both | 59+ | 59+ | 2,294 | 3,095 |
| 1963 | United States of America | 2011-2012 | US NHANES 2011-2012 | National | Both | 5+ | 5+ | 3,951 | 3,887 |
| 1964 | United States of America | 2014 | Health and Retirement Study | National | Both | 61+ | 61+ | 2,152 | 2,972 |
| 1965 | United States of America | 2013-2014 | US NHANES 2013-2014 | National | Both | 5+ | 5+ | 4,105 | 4,225 |
| 1966 | United States of America | 2015-2016 | US NHANES 2015-2016 | National | Both | 5+ | 5+ | 3,959 | 4,091 |
| 1967 | United States of America | 2015-2016 | National Social Life Health and Aging Project | Community | Both | 24-99 | 24-99 | 1,995 | 2,490 |
| 1968 | United States of America | 2016-2018 | Health and Retirement Study | National | Both | 63+ | 63+ | 1,638 | 2,321 |
| 1969 | United States of America | 2017-2018 | National Health and Nutrition Examination Survey | National | Both | 5+ | 5+ | 3,642 | 3,784 |

| 1970 | Uruguay | 1999-2000 | The Survey on Health, Well-Being, and Aging in Latin America and the Caribbean (SABE) | Community | Urban | 60+ | 60+ | 492 | 828 |
| --- | --- | --- | --- | --- | --- | --- | --- | --- | --- |
| 1971 | Uruguay | 2006 | STEPS | National | Both | 25-64 | 25-64 | 261 | 641 |
| 1972 | Uruguay | 2011-2012 | CESCAS Study | Community | Urban | 30-79 | 30-79 | 650 | 921 |
| 1973 | Uruguay | 2013 | STEPS | National | Urban | 15-64 | 15-64 | 821 | 1,400 |
| 1974 | Uruguay | 2012-2016 | Genotype, Phenotype and Environment of Hypertension in Uruguay (GEFA-HT-UY) | Community | Urban | 19+ | 19+ | 124 | 189 |
| 1975 | Uzbekistan | 1996 | DHS | National | Both |  | 15-49 |  | 4,082 |
| 1976 | Uzbekistan | 2002 | DHS | National | Both | 15-59 | 15-49 | 2,331 | 5,275 |
| 1977 | Uzbekistan | 2014 | STEPS | National | Both | 18-64 | 18-64 | 1,533 | 2,164 |
| 1978 | Uzbekistan | 2019 | STEPS | National | Both | 18-69 | 18-69 | 1,462 | 2,226 |
| 1979 | Vanuatu | 1996 | Second National Nutrition Survey | National | Both |  | 15-50 |  | 1,353 |
| 1980 | Vanuatu | 1998 | Vanuatu Non-comunicable Disease Survey | National | Both | 20-60 | 20-60 | 533 | 730 |
| 1981 | Vanuatu | 2005 | STEPS | Subnational | Both | 15-60 | 15-60 | 626 | 759 |
| 1982 | Vanuatu | 2011 | STEPS | National | Both | 25-64 | 25-64 | 2,251 | 2,183 |
| 1983 | Venezuela | 1999-2001 | Florez et al., Diabetes Res Clin Pract 69(1):63-77, 2005 | Subnational | Both | 15+ | 15+ | 1,134 | 2,599 |
| 1984 | Venezuela | 2004-2005 | CArdiovascular Risk factors Multiple Evaluation in Latin America | Community | Urban | 25-64 | 25-64 | 713 | 1,123 |
| 1985 | Venezuela | 2005-2006 | Brajkovich et al., Rev Ven Endoc Metab 4(3):31-32, 2006 | Community | Urban | 20-65 | 20-65 | 205 | 439 |
| 1986 | Venezuela | 2007-2008 | Venezuelan Study of Metabolic Syndrome, Obesity and Lifestyle (VEMSOLS) | Community | Urban | 20+ | 20+ | 107 | 230 |
| 1987 | Venezuela | 2008-2009 | Venezuelan Study of Metabolic Syndrome, Obesity and Lifestyle (VEMSOLS) | Community | Rural | 20+ | 20+ | 51 | 89 |
| 1988 | Venezuela | 2010-2011 | Venezuelan Study of Metabolic Syndrome, Obesity and Lifestyle (VEMSOLS) | Community | Urban | 20+ | 20+ | 66 | 193 |
| 1989 | Venezuela | 2015-2017 | Cardio-Metabolic Health Venezuelan Study (EVESCAM) | National | Both | 20+ | 20+ | 1,056 | 2,346 |
| 1990 | Viet Nam | 1987-1989 | General Nutrition Survey | National | Both | 15-70 | 15-70 | 16,012 | 19,574 |
| 1991 | Viet Nam | 1992-1993 | Living Standard Survey | National | Both | 5+ | 5+ | 9,418 | 10,209 |
| 1992 | Viet Nam | 1997-1998 | Living Standard Survey | National | Both | 5+ | 5+ | 12,052 | 13,118 |
| 1993 | Viet Nam | 2000 | National Nutrition Survey | National | Both | 20+ | 20+ | 8,985 | 9,464 |
| 1994 | Viet Nam | 2001-2003 | The National Epidemiological Survey on Hypertension and Its Risk Factors (North) | Subnational | Both | 25-74 | 25-74 | 2,386 | 3,604 |
| 1995 | Viet Nam | 2001-2002 | Viet Nam National Health Survey 2001-2002 | National | Both | 5+ | 5+ | 66,723 | 71,616 |
| 1996 | Viet Nam | 2003-2004 | The Survey on Heart Failure and Its Risk Factors | Subnational | Both | 25-74 | 25-74 | 1,853 | 2,636 |
| 1997 | Viet Nam | 2004 | The Hypertension Management Programme in Rural Communes (Hanoi) | Community | Rural | 25-74 | 25-74 | 855 | 1,288 |
| 1998 | Viet Nam | 2004 | Cuong et al., Eur J Clin Nutr 61(5):673-81, 2007 | Community | Urban | 20-60 | 20-60 | 717 | 771 |
| 1999 | Viet Nam | 2005 | The Survey on Non-Communicable Disease Risk Factors | Subnational | Both | 25-74 | 25-74 | 1,136 | 1,220 |
| 2000 | Viet Nam | 2005 | STEPS Bavi district | Subnational | Rural | 25-64 | 25-64 | 987 | 997 |
| 2001 | Viet Nam | 2005 | National Adult Overweight Survey | National | Both | 25-64 | 25-64 | 8,474 | 8,725 |
| 2002 | Viet Nam | 2005 | Non-communicable disease risk factors in Ho Chi Minh City | Community | Urban | 25-64 | 25-64 | 908 | 1,063 |
| 2003 | Viet Nam | 2006 | The Hypertension Management Programme in Rural Communes (Bavi) | Community | Rural | 25-74 | 25-74 | 395 | 643 |
| 2004 | Viet Nam | 2007 | The Hypertension Management Programme in Rural Communes (Phu Phuong) | Community | Rural | 25-74 | 25-74 | 364 | 616 |
| 2005 | Viet Nam | 2006-2008 | The National Epidemiological Survey on Hypertension and Its Risk Factors (South) | Subnational | Both | 25-74 | 25-74 | 1,310 | 2,078 |
| 2006 | Viet Nam | 2009 | STEPS | National | Both | 25-64 | 25-64 | 6,738 | 7,805 |
| 2007 | Viet Nam | 2008-2009 | The Survey on Diabetes and Its Risk Factors | Subnational | Both | 25+ | 25+ | 830 | 1,446 |
| 2008 | Viet Nam | 2009 | The Hypertension Management Programme in Rural Communes (Phu Cuong) | Community | Rural | 25-74 | 25-74 | 362 | 677 |
| 2009 | Viet Nam | 2009-2010 | Vietnam National Nutrition Survey 2009-2010 | National | Both | 5+ | 5+ | 16,036 | 16,619 |
| 2010 | Viet Nam | 2012 | National Survey of Diabetes in Vietnam | National | Both | 30-69 | 30-69 | 5,319 | 5,855 |
| 2011 | Viet Nam | 2015 | STEPS | National | Both | 18-69 | 18-69 | 1,316 | 1,722 |
| 2012 | Yemen | 1997 | DHS | National | Both |  | 15-49 |  | 5,123 |
| 2013 | Yemen | 2005-2006 | Yemen Household Budget Survey 2005-2006 | National | Both | 5+ | 5+ | 3,290 | 3,307 |
| 2014 | Yemen | 2007-2009 | Hypertension and Diabetes in Yemen (HYDY) | National | Rural | 6-70 | 6-70 | 3,023 | 3,065 |
| 2015 | Yemen | 2007-2009 | Hypertension and Diabetes in Yemen (HYDY) | National | Urban | 6-70 | 6-70 | 2,996 | 3,077 |
| 2016 | Yemen | 2013 | DHS | National | Both |  | 15-49 |  | 22,527 |
| 2017 | Zambia | 1992 | DHS | National | Both |  | 20-49 |  | 2,829 |
| 2018 | Zambia | 1996 | DHS | National | Both |  | 20-49 |  | 3,485 |
| 2019 | Zambia | 2001-2002 | DHS | National | Both |  | 15-49 |  | 6,732 |
| 2020 | Zambia | 2003 | Kelly et al., Am J Clin Nut 88(4):1010-17, 2008 | Community | Urban | 15-74 | 15-84 | 132 | 217 |
| 2021 | Zambia | 2007 | DHS | National | Both |  | 15-49 |  | 6,378 |
| 2022 | Zambia | 2008 | STEPS | Subnational | Urban | 25+ | 25+ | 626 | 1,214 |
| 2023 | Zambia | 2013-2014 | DHS | National | Both |  | 15-49 |  | 14,837 |
| 2024 | Zambia | 2017 | STEPS | National | Both | 18-69 | 18-69 | 1,565 | 2,439 |
| 2025 | Zimbabwe | 1985-1986 | INTERSALT | Community | Urban | 20-59 | 20-59 | 100 | 95 |
| 2026 | Zimbabwe | 1991 | Zinyowera et al., Cent Afr J Med 40(2):33-8, 1994 | Community | Both | 18+ | 18+ | 775 | 734 |
| 2027 | Zimbabwe | 1994 | DHS | National | Both |  | 20-49 |  | 1,776 |
| 2028 | Zimbabwe | 1995 | Mufunda et al., J Hum Hypertens 14(1):65-73, 2000 | Community | Urban | 25+ | 25+ | 384 | 391 |
| 2029 | Zimbabwe | 1999 | DHS | National | Both |  | 15-49 |  | 5,169 |
| 2030 | Zimbabwe | 2005 | STEPS | National | Both | 25+ | 25+ | 569 | 1,808 |
| 2031 | Zimbabwe | 2005-2006 | DHS | National | Both |  | 15-49 |  | 8,186 |
| 2032 | Zimbabwe | 2010-2011 | DHS | National | Both | 15-54 | 15-49 | 7,383 | 8,329 |
| 2033 | Zimbabwe | 2015 | DHS | National | Both | 15-54 | 15-49 | 8,386 | 9,396 |
