# Supplementary material for: Heterogeneous contributions of change in population distribution of body mass index to change in obesity and underweight
Source: eLife. 2021 Mar 9;10:e60060. doi: 10.7554/eLife.60060 (PMC7943191; doi:10.7554/eLife.60060)
Supplement: Supplementary file 5. [file elife-60060-supp5.docx]

**Supplementary file 5.** Mean body mass index estimates (kg/m^2^) in 1985 and 2016 stratified by region, gender and age group.

| **Sex** | **Year** | **Mean BMI (kg/m^2^)** | | | | | |
| --- | --- | --- | --- | --- | --- | --- | --- |
|  |  | **20-29** | **30-39** | **40-49** | **50-59** | **60-69** | **70-79** |
| Central and Eastern Europe | | | | | | | |
| Women | 1985 | 22.7 | 24.8 | 26.8 | 28.1 | 28.5 | 27.9 |
|  | 2016 | 23.3 | 25.6 | 27.7 | 29.1 | 29.5 | 28.7 |
| Men | 1985 | 23.1 | 24.7 | 25.6 | 25.9 | 25.9 | 25.5 |
|  | 2016 | 24.8 | 26.6 | 27.7 | 28.1 | 28.2 | 27.7 |
| Central Asia, the Middle East and North Africa | | | | | | | |
| Women | 1985 | 23.0 | 25.0 | 26.4 | 26.9 | 26.7 | 25.9 |
|  | 2016 | 25.4 | 28.2 | 30.0 | 30.7 | 30.4 | 29.0 |
| Men | 1985 | 22.4 | 24.0 | 24.7 | 24.8 | 24.5 | 24.0 |
|  | 2016 | 24.9 | 26.8 | 27.7 | 27.9 | 27.6 | 26.9 |
| East and Southeast Asia | | | | | | | |
| Women | 1985 | 20.6 | 21.4 | 22.1 | 22.2 | 22.0 | 21.4 |
|  | 2016 | 22.2 | 23.6 | 24.5 | 24.8 | 24.5 | 23.6 |
| Men | 1985 | 20.7 | 21.5 | 21.7 | 21.6 | 21.3 | 20.7 |
|  | 2016 | 22.9 | 24.0 | 24.6 | 24.6 | 24.3 | 23.7 |
| High-income Asia Pacific | | | | | | | |
| Women | 1985 | 20.8 | 21.6 | 22.5 | 23.1 | 23.3 | 23.0 |
|  | 2016 | 21.0 | 21.8 | 22.7 | 23.4 | 23.4 | 23.0 |
| Men | 1985 | 21.8 | 22.6 | 22.8 | 22.7 | 22.3 | 21.8 |
|  | 2016 | 23.2 | 24.2 | 24.5 | 24.5 | 24.0 | 23.3 |
| High-income western | | | | | | | |
| Women | 1985 | 23.2 | 24.3 | 25.4 | 26.4 | 26.8 | 26.6 |
|  | 2016 | 24.8 | 26.4 | 27.8 | 28.9 | 29.2 | 28.6 |
| Men | 1985 | 24.0 | 25.3 | 26.0 | 26.4 | 26.3 | 25.9 |
|  | 2016 | 26.1 | 27.7 | 28.6 | 29.1 | 29.0 | 28.4 |
| Latin America and the Caribbean | | | | | | | |
| Women | 1985 | 22.8 | 24.1 | 25.0 | 25.4 | 25.3 | 24.7 |
|  | 2016 | 25.2 | 27.3 | 28.7 | 29.2 | 29.0 | 28.0 |
| Men | 1985 | 22.8 | 24.1 | 24.7 | 24.7 | 24.3 | 23.6 |
|  | 2016 | 25.2 | 27.0 | 27.7 | 27.8 | 27.4 | 26.6 |
| Oceania | | | | | | | |
| Women | 1985 | 23.7 | 23.9 | 23.8 | 23.4 | 22.3 | 20.9 |
|  | 2016 | 26.2 | 27.4 | 27.9 | 27.8 | 26.6 | 24.8 |
| Men | 1985 | 23.0 | 23.7 | 23.8 | 23.5 | 22.9 | 22.2 |
|  | 2016 | 25.2 | 26.3 | 26.6 | 26.5 | 25.9 | 24.9 |
| South Asia | | | | | | | |
| Women | 1985 | 19.2 | 19.8 | 20.1 | 19.8 | 19.2 | 18.7 |
|  | 2016 | 21.2 | 22.6 | 23.2 | 23.1 | 22.5 | 21.6 |
| Men | 1985 | 19.3 | 20.0 | 20.2 | 19.9 | 19.3 | 18.8 |
|  | 2016 | 21.2 | 22.3 | 22.6 | 22.4 | 21.9 | 21.2 |
| Sub-Saharan Africa | | | | | | | |
| Women | 1985 | 20.9 | 21.3 | 21.6 | 21.5 | 21.1 | 20.6 |
|  | 2016 | 22.8 | 24.2 | 24.9 | 25.1 | 24.7 | 23.8 |
| Men | 1985 | 19.7 | 20.5 | 20.8 | 20.7 | 20.3 | 19.8 |
|  | 2016 | 21.4 | 22.5 | 22.9 | 23.0 | 22.7 | 22.1 |
